# Transcriptomic analyses in the gametophytes of the apomictic fern *Dryopteris affinis*

**DOI:** 10.1007/s00425-024-04540-w

**Published:** 2024-10-02

**Authors:** Sara Ojosnegros, José Manuel Alvarez, Valeria Gagliardini, Luis G. Quintanilla, Ueli Grossniklaus, Helena Fernández

**Affiliations:** 1 Area of Plant Physiology, Department of Organisms and Systems Biology, University of Oviedo, 33071 Oviedo, Spain; 2 Department of Plant and Microbial Biology, Zurich-Basel Plant Science Center, University of Zurich, 8008 Zurich, Switzerland; 3 Global Change Research Institute, University Rey Juan Carlos, 28933 Móstoles, Spain

**Keywords:** Apogamy, Evo-devo, Fern, RNA-Seq, Genomic

## Abstract

**Main conclusion:**

A novel genomic map of the apogamous gametophyte of the fern *Dryopteris affinis* unlocks oldest hindrance with this complex plant group, to gain insight into evo-devo approaches.

**Abstract:**

The gametophyte of the fern *Dryopteris affinis* ssp. *affinis* represents a good model to explore the molecular basis of vegetative and reproductive development, as well as stress responses. Specifically, this fern reproduces asexually by apogamy, a peculiar case of apomixis whereby a sporophyte forms directly from a gametophytic cell without fertilization. Using RNA-sequencing approach, we have previously annotated more than 6000 transcripts. Here, we selected 100 of the inferred proteins homolog to those of *Arabidopsis thaliana*, which were particularly interesting for a detailed study of their potential functions, protein–protein interactions, and distance trees. As expected, a plethora of proteins associated with gametogenesis and embryogenesis in angiosperms, such as FERONIA (FER) and CHROMATING REMODELING 11 (CHR11) were identified, and more than a dozen candidates potentially involved in apomixis, such as ARGONAUTE family (AGO4, AGO9, and AGO 10), BABY BOOM (BBM), FASCIATED STEM4 (FAS4), FERTILIZATION-INDEPENDENT ENDOSPERM (FIE), and MATERNAL EFFECT EMBRYO ARREST29 (MEE29). In addition, proteins involved in the response to biotic and abiotic stresses were widely represented, as shown by the enrichment of heat-shock proteins. Using the String platform, the interactome revealed that most of the protein–protein interactions were predicted based on experimental, database, and text mining datasets, with MULTICOPY SUPPRESSOR OF IRA4 (MSI4) showing the highest number of interactions: 16. Lastly, some proteins were studied through distance trees by comparing alignments with respect to more distantly or closely related plant groups. This analysis identified DCL4 as the most distant protein to the predicted common ancestor. New genomic information in relation to gametophyte development, including apomictic reproduction, could expand our current vision of evo-devo approaches.

**Supplementary Information:**

The online version contains supplementary material available at 10.1007/s00425-024-04540-w.

## Introduction

The most studied aspects in ferns are photomorphogenesis (Wada 2007), spore germination (Suo et al. 2015a, b), cell polarity and wall composition (Salmi and Bushart 2010; Eeckhout et al. 2014), reproduction (López and Renzaglia 2014; Valledor et al. 2014; de Vries et al. 2016; Grossmann et al. 2017; Rivera et al. 2018; Wyder et al. 2020; Fernández et al. 2021; Ojosnegros et al. 2022, 2023), or adaptation to the environment (Sareen et al. 2019), as ferns survive atmospheric conditions with a high carbon dioxide, and resist in situations of salinity, drought, or soil contamination with heavy metals (Wang et al. 2010). They are also efficient at extracting pollutants from the environment (Dhir 2018).

Molecular studies on ferns are scarce. Ferns occupy the last positions in the genome sequencing race due to their high chromosome number and large genome size (Manton 1950; Barker and Wolf 2010; Sessa and Der 2016). To our knowledge, the genomes of only seven fern species are accessible: *Alsophila spinulosa*, *Azolla filiculoides*, *Ceratopteris richardii*, *Salvinia cucullata* (Aragón-Raygoza et al. 2022), *Adiantum capillus-veneris* (Fang et al. 2022), *Adiantum nelumboides* (Zhong et al. 2022), and *Marsilea vestita* (Rahmatpour et al. 2023). Thanks to the advent of next-generation sequencing methodologies, more genomes will soon be deciphered, as predicted by Kinosian and Wolf (2022).

Ferns are a monophyletic group within the vascular lineage (PPG I 2016). Their biological cycle has two independent generations alternate: the sporophyte, which forms spores by meiosis and is typically diploid, and the gametophyte, which forms gametes by mitosis and is usually haploid. In addition to sexual reproduction by gamete fusion, ferns exhibit asexual reproduction by apomixis, which involves two processes: diplospory (generation of unreduced spores by altered meiosis) and apogamy (embryo formation from somatic cells of the gametophyte) (Liu et al. 2012; Grusz et al. 2021). Apomixis may occur when environmental conditions are unfavourable, e.g. lack of light or water (Grusz et al. 2021), or when gametes are nonfunctional. This process is more frequent in ferns than in any other group of land plants (Dyer et al. 2012). In particular, it has been estimated that 10% of fern species have obligate apogamy (Liu et al. 2012). Their introduction into sexual crops has long been one of the main challenges for plant breeders (Grossniklaus et al. 2001; Spillane et al. 2004). The molecular and developmental study of ferns can contribute to this, as they are the lineage most closely related to seed plants.

The gametophyte of the fern *Dryopteris affinis* (Lowe) Fraser-Jenk. ssp. *affinis* has been widely used as a model for understanding apomixis (Cordle et al. 2012; Rivera et al. 2018). This fern produces unreduced spores that give rise to diploid gametophytes that form male gametangia (antheridia), but not female gametangia (archegonia). Sexual reproduction is not feasible, so *D. affinis* is obligately apogamous (Menéndez et al. 2006). Within the family Dryopteridaceae, to which it belongs, 3% of the species show this type of reproduction (Grusz et al. 2021). In this apomixis, there is not change in ploidy level between the gametophyte and sporophyte stages. In addition to this characteristic, the gametophyte has more advantages for the study of asexual reproduction, such as its easy in vitro culture from spores, its small size (approximately 4 × 4 mm) that allows large samples of individuals in a reduced culture space, and direct observation of complete individuals under microscope (Rivera et al. 2018). In Wyder et al. (2020), a RNA analysis using next generation sequencing (RNA-seq) of *D. affinis* gametophytes generated a transcriptome, and showed differential gene expression between the two stages of gametophyte growth: the initial one-dimensional and the subsequent two-dimensional. Here, we perform an in silico study to analyse this transcriptome in detail, specifically, around one hundred proteins homolog to those of *Arabidopsis thaliana* was described by their possible function in the gametophyte, interactions between them, and distance trees. For a better understanding, they are discussed by clustering them into three groups: vegetative development, reproductive development (apomixis and sexual), and response to stress.

## Materials and methods

### Plant material and growing conditions

Fertile fronds from sporophytes of *D. affinis* were collected in Turón forest (Asturias region, Spain, 43°12′10′′N–5°43′43′′W, 477 m a.s.l.) and carried to the lab, where the spores were released from the sporangia, rinsed in water for 2 h and washed for 10 min with a NaCl (0.5%), and Tween 20 (0.1%) solution. Spores were then rinsed three times with distilled water, centrifuged at 1300*g* for 3 min between rinses, and cultured in 500 ml Erlenmeyer flasks containing 100 ml of Murashige and Skoog (MS) medium (Murashige and Skoog 1962) supplemented with 2% sucrose (w/v) at pH 5.7. One-dimensional filamentous gametophytes were obtained by liquid culture of spores at high density on a rotating shaker (75 rpm) for 50 days, and two-dimensional spatulate and heart-shaped gametophytes were obtained by maintaining spores in Petri dishes with 25 ml of MS medium supplemented with 2% sucrose (w/v) and 0.7% agar at pH 5.7 for 65 days. All cultures were grown at 25 °C with a photosynthetically active radiation intensity of 40 μmol m^−2^ s^−1^ and a photoperiod of 16 h light and 8 h dark.

### RNA extraction and sequencing

Methods for RNA extraction and sequencing are summarized in Wyder et al. (2020). Briefly, for RNA extraction, 100 mg of fresh plant material was weighed, frozen in liquid nitrogen and stored at −80 °C until use. Between 3 and 5 biological replicates of gametophytes from each growth stage were used for RNA-Seq. Gametophytes were homogenised with a Silamat S5 shaker (Ivoclar Vivadent, Schaan, Liechtenstein) twice for 10 and 5 s, respectively. Total RNA was isolated using the SpectrumTM Plant Total RNA kit (Sigma-Aldrich, Buchs, Switzerland). After DNA removal with the TURBO DNA-free kit (Life Technologies, Carlsbad, USA), RNA quality was tested with the Bioanalyser Agilent RNA 6000 Pico Kit (Agilent Technologies, Waldbronn, Germany). Sequencing libraries were prepared with the TruSeq RNA Sample Prep Kit v2 and sequenced on the Illumina HiSeq 2000. The Transcriptome Shotgun Assembly project is available at the European Nucleotide Archive (ENA) (http://www.ebi.ac.uk/ena) with the accession number PRJEB18522. The de novo assembly of the transcriptome in fasta format and the transcriptome annotation had been deposited in the Zenodo research data repository (www.zenodo.org) (https://doi.org/10.5281/zenodo.1040330). The highly similar contigs assembled by Trinity were collapsed using Corset 0.93 (Davidson and Oshlack 2014) with a distance threshold of 0.3, which resulted in 166,191 transcript clusters (with a minimum contig size of 201 nucleotides). BUSCO version 2.0.1 with the Embryophyta odb9 dataset to assess transcriptome completeness was used.

### “In silico” analysis and protein analysis using the STRING platform

An in silico analysis of the transcriptome was carried out with the GENEIOUS PRIME software version 2023.2.1, using Araport11 database, to increase the number of homologies or identities, and to obtain a more exhaustive mapping of the genomic background of *D. affinis* gametophyte. As the genome of this species is not yet sequenced, the transcripts were blasted against *A. thaliana*, specifically, a BLASTX was performed. Transcripts annotations with more than 450 bp and an *E* value of less than 10^–20^ were selected. Gene identifiers were used as input for Gene Ontology (GO) pathway enrichment analysis, including the three categories: biological function, molecular function, and cellular components; Kyoto Encyclopedia of Genes and Genomes (KEGG) and SMART protein domains, using ShinyGO version 0.741 platform. Fold enrichment or gene ratio, i.e. the number of genes present over the total number of genes in that category, was also shown. The processes that were selected for their relevance to this study were analysed by STRING platform version 12.0 choosing a high threshold (0.700) to infer the possible function of transcripts and associations between them. In this sense, protein–protein interactions can be of different channels: (a) experiments: proteins that have been shown to have chemical, physical, or genetic interactions in laboratory experiments; (b) data bases: proteins found in the same databases; (c) text mining: proteins mentioned in the same PubMed abstract or articles from an internal selection of the STRING software; (d) co-expression: protein expression patterns are similar; (e) neighbourhood: protein-coding genes are close together in the genome; (f) gene fusion: in at least one organism orthologous protein-coding genes are fused into a single gene; (g) co-occurrence: proteins with a similar phylogenetic distribution; and (h) homology: proteins that have a common ancestor and similar sequences. These interactions are assigned to scores close to 0 if they are weak, or close to 1 if they are strong.

### Distance trees and protein domains analysis

Distance trees analysis was carried out in some protein sequences using NCBI Genome Workbench and BLASTP. For trees construction, the following parameters were selected: expect threshold of 0.05, BLOSUM62 matrix with gap costs of existence of 11 and extension of 1, minimum fast evolutionary tree method, maximum sequence distance of 0.85, and Grishin distance. In addition, only the first 50 sequences with the lowest *E* values were chosen. Amino acid sequence alignments were obtained using the column-based method in the same programmes. Lastly, the sequences of selected *D. affinis* proteins were introduced in SMART software version 9.0 to obtain protein domains and compared with those of *A. thaliana*, obtained using the same software. Its sequences were taken from the TAIR software.

## Results

The blasted (BLASTX) *D. affinis* transcriptome showed 27,037 results corresponding to 6142 protein annotations.

### Protein enrichment classification

**Fig. 1:**
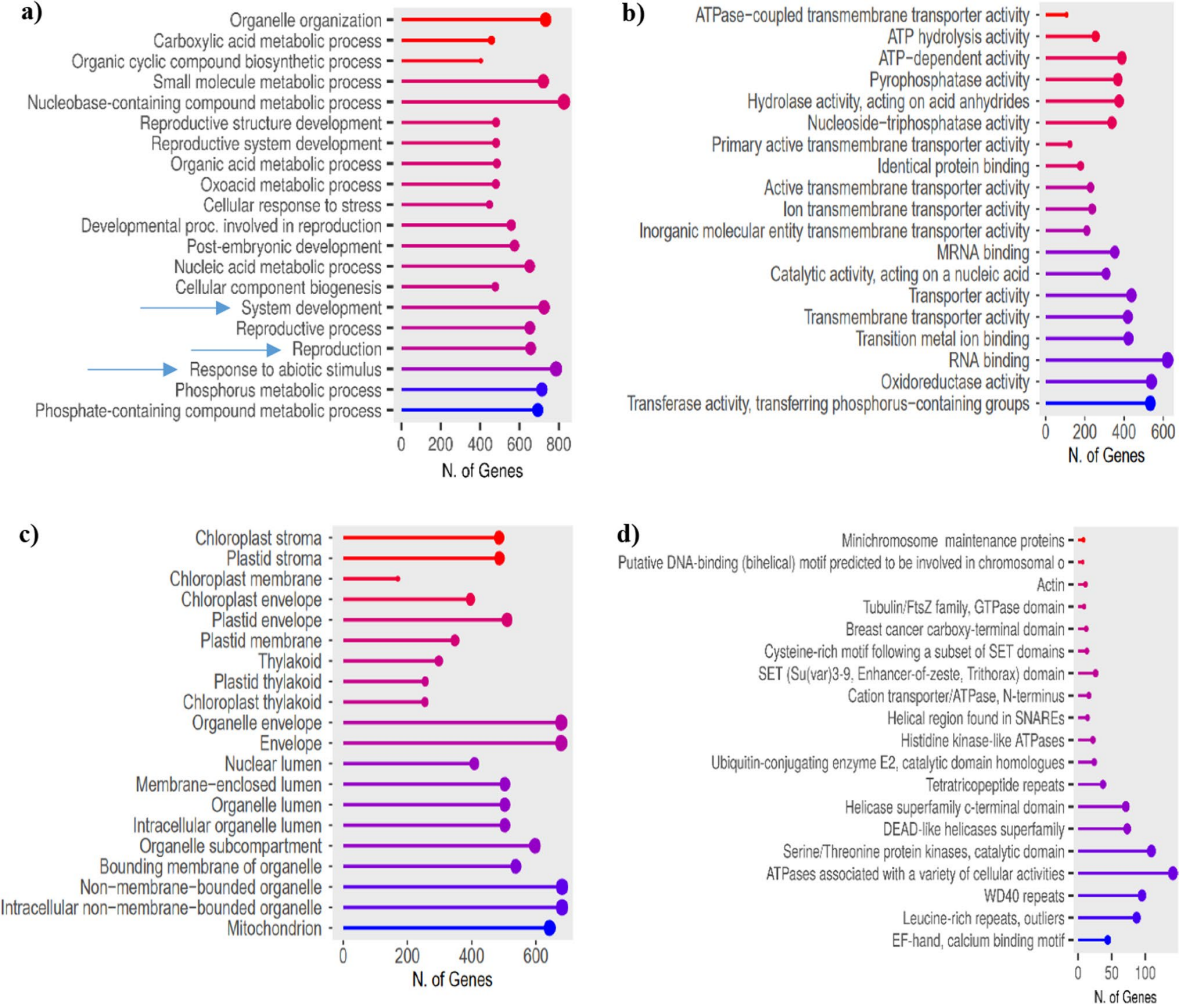
Classifications of proteins extracted from gametophytes of *D. affinis* analysed with ShinyGO program. The size of the balls indicates the number of genes in each category. The fold enrichment is represented by a colour spectrum: blue shades indicate low values, while red shades high values. **a** GO biological function. Blue arrows point processes involving proteins explained in detail in the discussion section. **b** GO molecular function. **c** GO cellular component. **d** SMART protein domains

Based on the GO classification of biological function (Fig. 1a), the categories with the highest number of genes were nucleobase-containing compound metabolic process and response to abiotic stimulus. The fold enrichment was highest in organelle organisation and carboxylic acid metabolic process, and lowest in development and response to abiotic stimulus. Regarding molecular function (Fig. 1b), RNA binding and oxidoreductase activity were the most represented. In this case, the fold enrichment value highest in the ATPase-coupled transmembrane transporter and ATP hydrolysis activities, and lowest in oxidoreductase and transferase categories. In terms of cellular components (Fig. 1c), disregarding the organelle envelope and non-membrane-bounded organelle categories, proteins were mostly located in mitochondrion and chloroplasts. The fold enrichment was highest in different parts of chloroplasts, and lowest in mitochondrion. As for the protein domains obtained from SMART software (Fig. 1d), the highest number corresponded to ATPases associated with a variety of cellular activities, and catalytic domains of serine/threonine protein kinases. Regarding fold enrichment, the highest values were in minichromosome maintenance proteins and actin, while the lowest in EF-hand calcium binding motifs and leucine rich repeats.

**Fig. 2:**
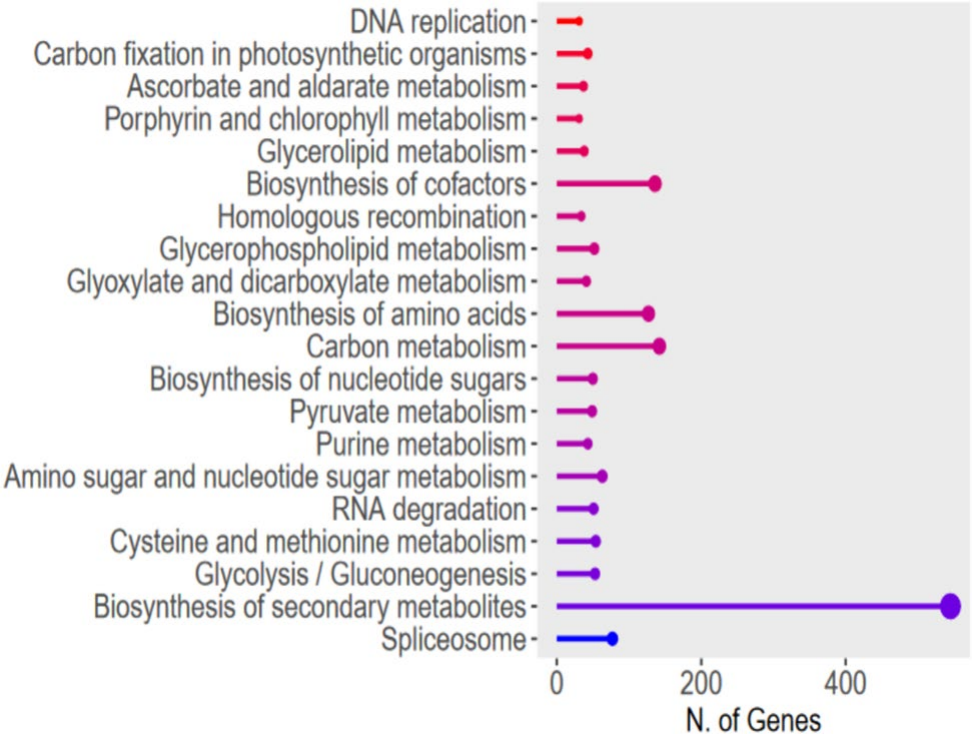
KEGG classification of proteins extracted from gametophytes of *D. affinis* analysed with ShinyGO program. The size of the balls indicates the number of genes in each category. The fold enrichment is represented by a colour spectrum: blue shades indicate low values, while red shades high values

Likewise, KEGG classification (Fig. 2) revealed that the most abundant categories are primary and secondary metabolism, highlighting the biosynthesis of secondary metabolites. The fold enrichment was highest in DNA replication and carbon fixation in photosynthetic organisms, and lowest in the spliceosome and biosynthesis of secondary metabolites.

### Proteins associated with apomixis

Within the total number of proteins obtained, as expected, numerous homologs to *A. thaliana* were linked to reproduction, and specifically to apomixis (Table 1). Among them, there were members of the DEAD box helicase family involved in gametogenesis and embryogenesis, such as FASCIATED STEM 4 (FAS4), MATERNAL EFFECT EMBRYO ARREST 29 (MEE29) and SWITCH1 (SW1). BRI1-ASSOCIATED RECEPTOR KINASE (BAK1), from the SERK family required in somatic embryogenesis, was also found. Regarding the WD-40 repeat family, SLOW WALKER1 (SWA1) and MULTICOPY SUPPRESSOR OF IRA1 (MSI1) were found. Likewise, a member of the WD repeat ESC family associated with parthenogenesis was described: FERTILIZATION INDEPENDENT ENDOSPERM (FIE). BABY BOOM (BBM) was another known protein related to parthenogenesis, somatic embryogenesis and apogamy, belonging to the AP2/ERF family. From the MADS-box family, AGAMOUS-LIKE 62 (AGL62) and 6 (AGL6), required in endosperm development and megasporogenesis, were detected. Several ARGONAUTE family proteins connected to apomixis were identified: AGO4, AGO9 and AGO10, all involved in gene silencing. Finally, SERRATE (SE), from the ARS2 family, also important in gene silencing, was obtained.

**Table 1:** Proteins obtained from the gametophyte of *D. affinis* associated with apomixis

Gene name	Protein name	Description	Function	UniProtKB/Swiss-Prot	*E*-value	MW (kDa)	Amino acids	Number of transcripts	Transcriptome identifier	Authors	Species
*AP2/ERF family*											
*BBM*	BABY BOOM	Transcription factor	Induces parthenogenesis, somatic embryogenesis and apogamy	Q6PQQ4	6 × 10^−117^	65	584	1	369145-36	Boutilier et al. (2002) Schmidt (2020) Bui et al. (2017)	*Brassica napus* *A. thaliana* *C. richardii*
*ARGONAUTE family*											
*AGO4*	ARGONAUTE 4	Transferase	Gene silencing. Loss of function mutants show apospory	Q9ZVD5	1 × 10^−153^	103	924	5	283466-78	Olmedo-Monfil et al. (2010)	*A. thaliana* *Hieracium*
*AGO9*	ARGONAUTE 9			Q84VQ0	0	101	896	1	5517-961	Rabiger et al. (2016)	
*AGO10*	ARGONAUTE 10			Q9XGW1	1 × 10^−39^	111	988	2	89522-265	Grossmann et al. (2017)	
*ARS2 family*											
*SE*	SERRATE	RNA effector	miRNA silencing	Q9ZVD0	1 × 10^−147^	81	720	1	284827-77	Dong et al. (2008)	*A. thaliana*
*DEAD box helicase family*											
*FAS4*	FASCIATED STEM 4	RNA helicase	Crucial for male and female gametogenesis	Q9C813	0	139	1237	1	214887-125	Schmidt (2020)	*Boechera*
*MEE29*	MATERNAL EFFECT EMBRYO ARREST 29		Pre-mRNA splicing in embryogenesis	F4IJV4	0	119	1044	1	77-3381	Barcaccia and Albertini (2013)	*Hypericum perforatum*
*SWI1*	SWITCH1	–	Fertility, chromatid cohesion, and chromosome structure in female gamete meiosis	Q9FGN8	9 × 10^−32^	73	639	1	90,790-263	Siddiqi et al. (2000)	*A. thaliana*
*MADS-box family*											
*AGL6*	AGAMOUS-LIKE 6	Transcription factor	Megasporogenesis	P29386	9 × 10^−33^	29	252	1	398,037-24	Guimaraes et al. 2013	*Brachiaria brizantha*
*AGL62*	AGAMOUS-LIKE 62		Nuclear proliferation in early endosperm development	Q9FKK2	5 × 10^−27^	35	299	3	42,863-389	Kang et al. (2008)	*A. thaliana*
*SERK family*											
*BAK1*	BRI1-ASSOCIATED RECEPTOR KINASE	Kinase	Somatic embryogenesis	Q94F62	0	68	615	1	394,361-25	Albertini et al. (2005)	*Poa pratensis*
*WD-40 repeat family*											
*MSI1*	MULTICOPY SUPPRESSOR OF IRA1	Core histone-binding subunit	Mutants show parthenogenetic development of unfertilized embryo and endosperm	O22467	0	48	424	5	765-1800	Guitton and Berger (2005)	*A. thaliana*
*SWA1*	SLOW WALKER1	RNA helicase	Megagametogenesis	O82266	9 × 10^−98^	59	530	1	20,540-550	Shi et al. (2005)	*A. thaliana*
*WD repeat ESC family*											
*FIE*	FERTILIZATION-INDEPENDENT ENDOSPERM	Polycomb group	Chromatin silencing and genetic imprinting. Mutants show development of egg cells into embryos without fertilization	Q9LT47	1 × 10^−163^	41	369	2	78,705-284	Ohad et al. (1999)	*A. thaliana*

**Fig. 3:**
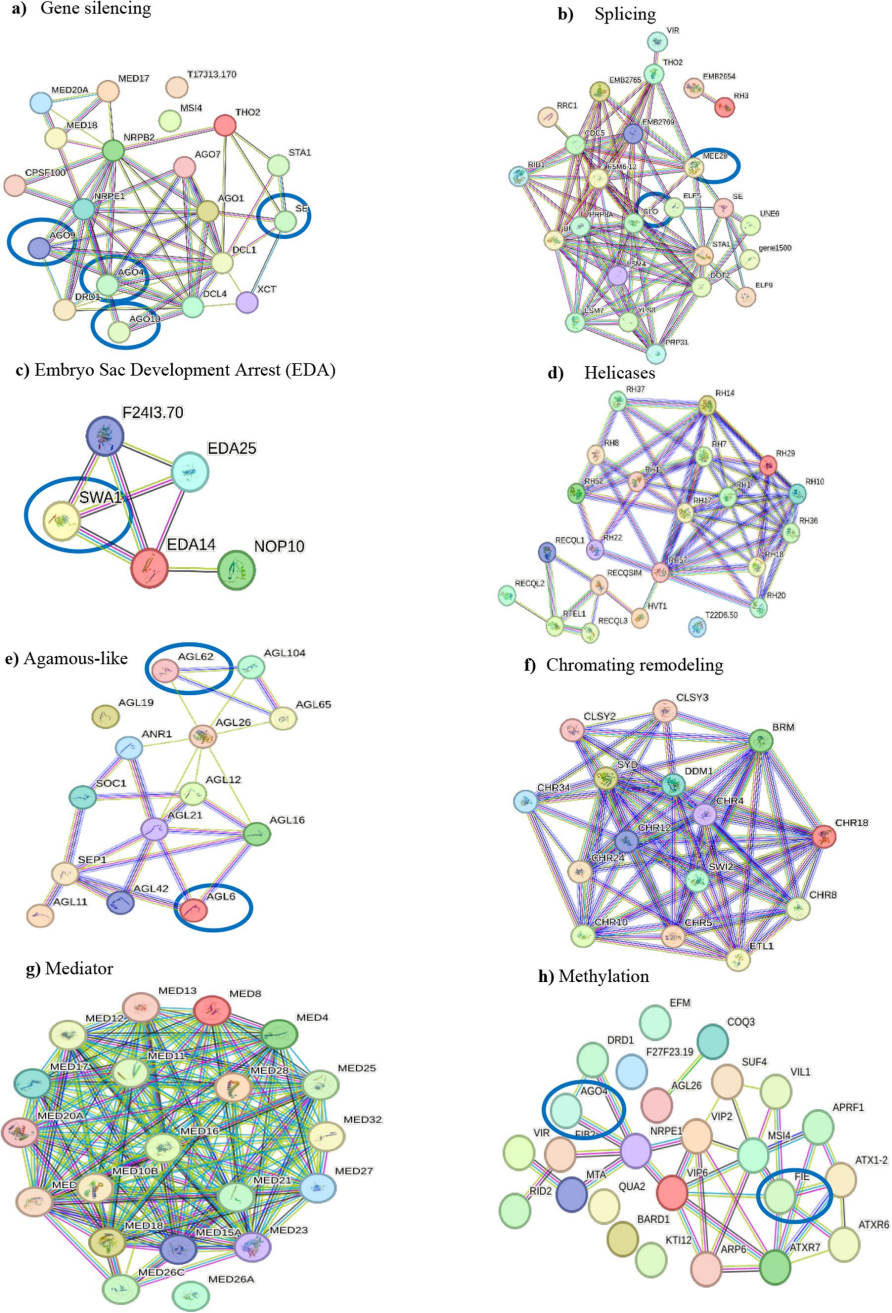
Networks formed by protein clusters interacting with apomictic candidate genes, marked with a blue circle, obtained from gametophytes of *D*. *affinis*, provided by String program. Colour indicates the type of protein–protein interaction: pink lines refer to evidence from experiments, yellow lines from text mining, black lines from co-expression, and blue lines from databases

**Fig. 4:**
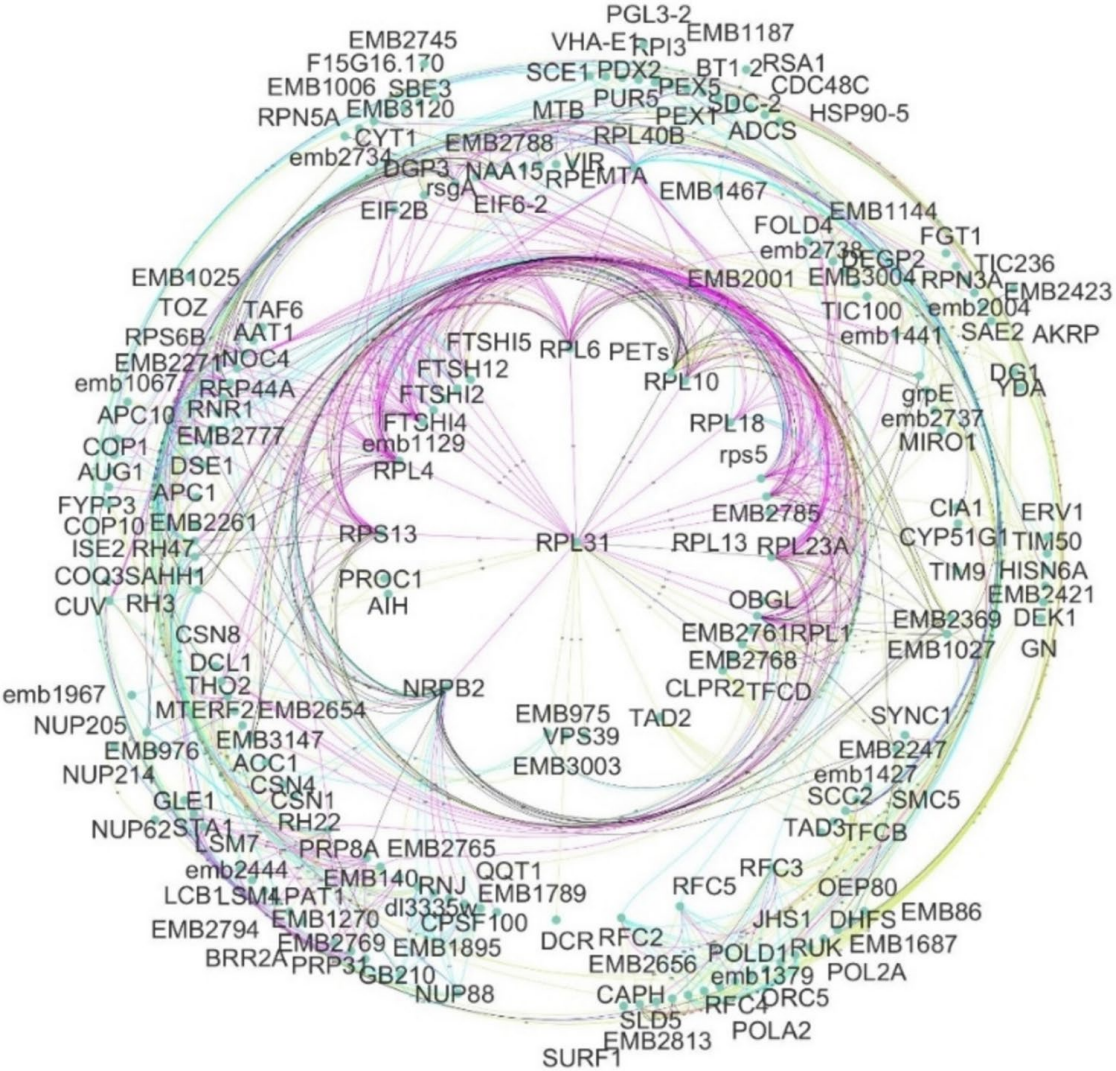
Embryo defective proteins annotated from gametophytes of *D. affinis* provided by String program and edited with Cytoscape software. Colour indicates the type of protein–protein interaction: pink lines refer to evidence from experiments, yellow lines from text mining, black lines from co-expression, and blue lines from databases

Most of the homolog proteins related to apomixis connect to relevant molecular processes operating behind gametogenesis or embryogenesis, such as gene silencing by RNA, splicing, chromatin remodelling, and methylation, among others. The large assortment of proteins related to these processes found in the *D. affinis* transcriptome can be observed in Fig. 3. This is the case of the ARGONAUTE proteins identified: AGO1, AGO4, AGO7, AGO9 and AGO10, associated with gene silencing during transcription and post-transcription, as well as SERRATE (SE), implicated in the control of meristem activity by gene silencing (Fig. 3a). Proteins involved in splicing were found, specifically 33 linked to the maternal effect embryo arrest protein group, such as MATERNAL EFFECT EMBRYO ARREST 29 (MEE29) (Fig. 3b). The embryo sac development arrest (EDA) group was represented by 24 transcripts, such as SLOW WALKER 1 (SWA1), 2 (SWA2) and 3 (SWA3), some of them involved in megagametogenesis (Fig. 3c). The number of helicases (Fig. 3d) and AGAMOUS-LIKE proteins, exemplified by AGAMOUS-LIKE 62 (AGL62) and 6 (AGL6) (Fig. 3e) was striking. Kinases were also very abundant in the transcriptome, with a total of 345 (5.6% of all proteins obtained). Likewise, relevant genomic processes such as chromatin remodelling (Fig. 3f) and transcription regulation, represented by proteins of the mediator complex (Fig. 3g), counted with several proteins. Finally, proteins operating in methylation were copious, such as FERTILIZATION-INDEPENDENT ENDOSPERM (FIE), belonging to the Polycomb group (Fig. 3h). The presence of 250 transcripts corresponding to embryo defective proteins (EMB) was also a remarkable result (Fig. 4).

### Proteins associated with vegetative development, sexual reproductive development, and response to stress

**Fig. 5:**
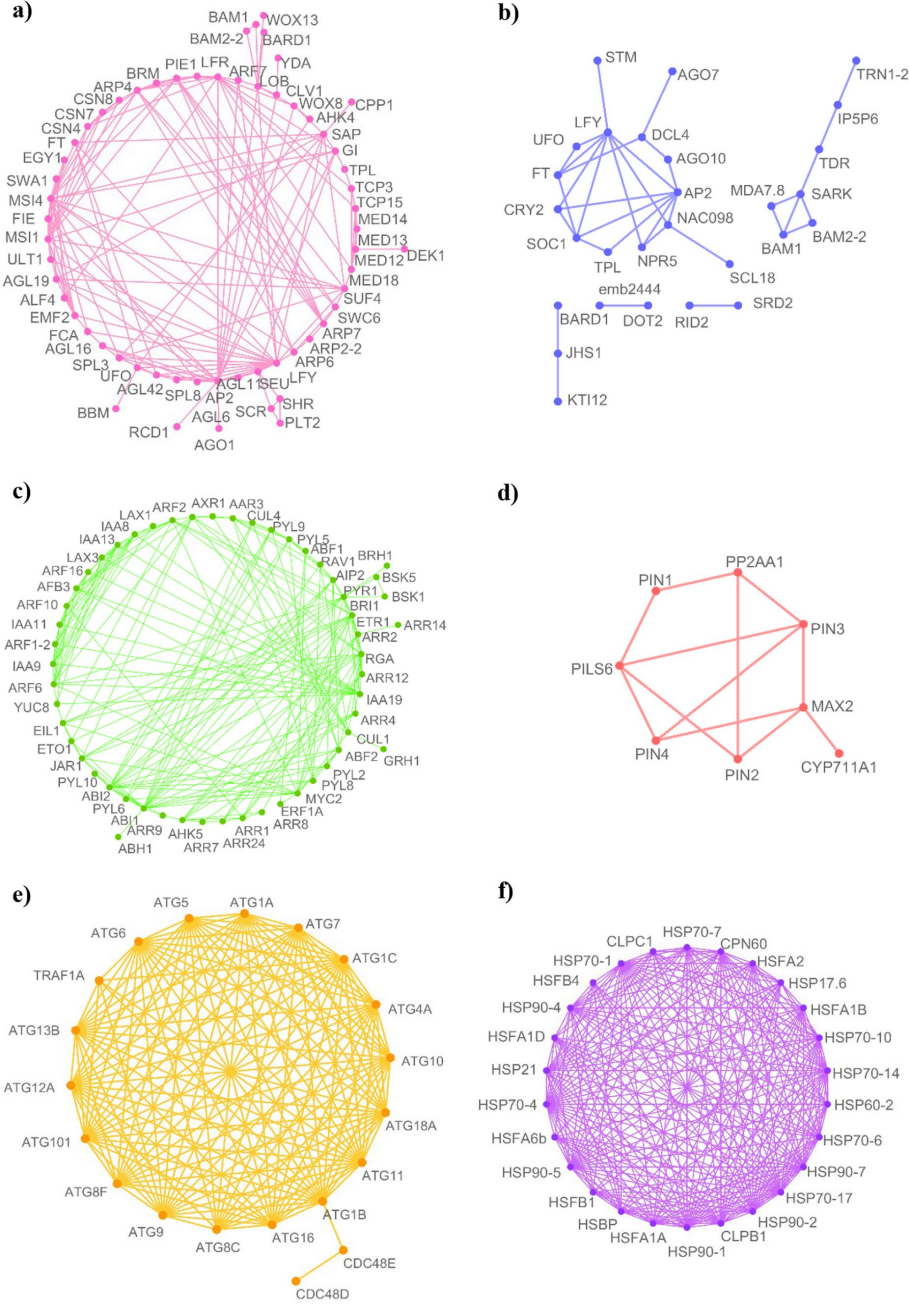
Networks of interactions between proteins obtained from the gametophyte of *D. affinis* related to **a** vegetative and reproductive growth; **b** meristem development; **c** hormone signalling; **d** auxin transport; **e** autophagy, and **f** heat-shock protein family, provided by String program and edited with Cytoscape software

In addition to apomixis proteins, a high number of proteins homolog to those of *A. thaliana* related to vegetative development, sexual reproductive development, and response to stress were identified among the total proteins obtained from the *D. affinis* transcriptome (Fig. 5). Concerning the development of anatomical structures, many orthologs were found, such as HISTONE CHAPERONE HIRA (HIRA) and ABERRANT LATERAL ROOT FORMATION 4 (ALF4), implicated in leaf and root formation (Fig. 5a), or SHOOT MERISTEMLESS (STM), which participates in the formation of the shoot apical meristem during embryogenesis (Fig. 5b). The development of the gametophyte requires a coordinated action of growth regulators, and proteins involved in its metabolism, transport and signalling were found (Fig. 5c). Proteins related to sexual reproduction, such as WUSCHEL RELATED HOMEOBOX 8 (WOX8), were identified. Despite the absence of flowers in fern, homologs of *A. thaliana* proteins essential in the transition to flowering were reported, one of them being FLOWERING PROMOTING FACTOR 1 (FPF1). Likewise, related to the circadian clock is TIMEKEEPER LOCUS 1 (STIPL1). The presence of numerous autophagy-related genes was striking (Fig. 5d). Regarding the response to abiotic stress, proteins coping with cold or heat were identified, such as ABA DEFICIENT 3 (ABA3), as well as heat-shock proteins (Fig. 5e). Some examples of proteins related to biotic stress were DICER-LIKE 4 (DCL4) and PRIORITY IN SWEET LIFE 4 (PSL4), intervening in defence against virus and bacteria.

Around 100 homolog *A. thaliana* proteins of *D. affinis* related to vegetative development, sexual reproductive development and response to stress were studied (Table 2). Their sequences, together with those of the apomixis proteins, are shown in Supplementary Table 1.

**Table 2:** Selected proteins found in gametophytes of *D. affinis* associated to vegetative development, sexual reproductive development and response to stress

Category	UniProtKB/Swiss-Prot	Gene name	Protein name	MW (kDa)	Amino acids	E value	Number of transcripts	Transcriptome identifier
Cell division and differentiation	Q8RWV3	*RCY1*	ARGININE-RICH CYCLIN 1	48	416	1 × 10^–152^	11	10087-752
	Q9C5X3	*KEU*	KEULE	75	666	0	1	155405-187
	Q84XF3	*ADL1B*	DYNAMIN-LIKE 1B	68	610	6 × 10^–6^	1	112514-234
	Q8RXA7	*SCD1*	STOMATAL CYTOKINESIS-DEFECTIVE 1	132	1,187	0	1	280538-80
	B0M1H3	*ATSPO22*	*A. THALIANA* HOMOLOGUE OF YEAST SPO22	104	936	8 × 10^–48^	4	158706-182
Cytoskeleton	Q9LQ81	*ADF11*	ACTIN DEPOLYMERIZING FACTOR 11	16	140	5 × 10^–64^	2	57059-335
Organ development	Q84VX3	*ALF4*	ABERRANT LATERAL ROOT FORMATION 4	66	602	1 × 10^–21^	2	307893-64
	Q8H0U4	*TRN1*	TORNATE 1	100	895	5 × 10^–75^	38	324482-55
	Q9LXN4	*HIRA*	HISTONE CHAPERONE HIRA	116	1058	1 × 10^–6^	1	48519-365
	Q01525	*GRF2*	GROWTH-REGULATING FACTOR 2	29	259	8 × 10^–25^	2	215396-125
	Q9C9Z2	*RER3*	RETICULATA-RELATED 3	35	337	4 × 10^–87^	3	159902-180
	Q94CJ5	*RER4*	RETICULATA-RELATED 4	41	386	1 × 10^–120^	5	127586-218
	Q38874	*STM*	SHOOT MERISTEMLESS	43	382	3 × 10^–67^	2	118041-228
Growth regulators	P33487	*ABP1*	ENDOPLASMIC RETICULUM AUXIN BINDING PROTEIN 1	22	198	6 × 10^–70^	1	255679-96
	Q9S7Z8	*PIN3*	PIN-FORMED 3	69	640	0	3	180789-155
	Q9S814	*EIN2*	ETHYLENE INSENSITIVE 2	141	1,294	2 × 10^–76^	2	412799-19
	O65020	*ETO1*	ETHYLENE OVERPRODUCER 1	107	951	0	1	1753-1462
	F4ITL6	*RTE1*	REVERSION-TO-ETHYLENE SENSITIVITY1	28	250	3 × 10^–84^	5	417835-15
	Q39208	*STE1*	STEROL 1	33	281	5 × 10^–12^	1	8180-819
	Q9XIP7	*GTG1*	GPCR-TYPE G PROTEIN 1	54	468	0	1	24318-509
	Q58G47	*JAZ4*	JASMONATE-ZIM-DOMAIN PROTEIN 4	34	310	5 × 10^–15^	1	169854-167
Photomorphogenesis	P48732	*DET1*	DE-ETIOLATED 1	62	543	0	1	188817-148
	Q8LGH4	*CUL4*	CULLIN 4	91	792	0	2	272777-85
	O24646	*HY5*	ELONGATED HYPOCOTYL 5	18	168	2 × 10^–26^	8	94888-257
Circadian clock	Q8H110	*XCT*	XAP5 CIRCADIAN TIMEKEEPER	39	337	1 × 10^–113^	3	61693-322
	Q9SHG6	*STIPL1*	TIMEKEEPER LOCUS1	97	849	0	2	144128-203
	Q570U6	*ELF4-L4*	ELF4-LIKE 4	13	114	3 × 10^–27^	1	238398-107
Senescence	Q9SIM9	*MAX2*	MORE AXILLARY BRANCHES 2	77	693	3 × 10^–95^	3	263060-91
	Q6USK2	*ACD5*	ACCELERATED CELL DEATH 5	68	608	3 × 10^–51^	6	430042-4
Autophagy	Q9FFI2	*APG5*	AUTOPHAGY 5	38	337	1 × 10^–64^	1	255322-96
	Q9SUG7	*ATG11*	AUTOPHAGY-RELATED 11	129	1148	2 × 10^–12^	3	5559-958
Gametogenesis	Q6TLJ2	*TPD1*	TAPETUM DETERMINANT 1	19	176	2 × 10^–30^	2	399439-23
	Q9LFS2	*GEX3*	GAMETE EXPRESSED 3	73	641	2 × 10^–26^	4	324001-56
	Q9LSZ9	*LCB2*	LONG CHAIN BASE 2	54	489	0	3	135224-211
	Q9SCZ4	*FER*	FERONIA	98	895	0	3	155201-188
Embryogenesis	Q8W104	*FBL17*	F BOX-LIKE17	65	593	4 × 10^–70^	5	162055-177
	O04379	*AGO1*	ARGONAUTE 1	116	1048	0	11	93133-259
	Q9C793	*AGO7*	ARGONAUTE 7	113	990	2 × 10^–75^	1	197199-140
	F4IDC2	*SWA2*	SLOW WALKER 2	117	1043	3 × 10^–168^	2	12016-696
	Q9SA27	*SWA3*	SLOW WALKER 3	55	491	4 × 10^–176^	1	30623-457
	Q8RWY3	*CHR11*	CHROMATIN-REMODELING PROTEIN 11	122	1055	4 × 10^–136^	2	6301-911
	P46667	*HB5*	HOMEOBOX PROTEIN 5	35	312	9 × 10^–30^	3	283256-78
	O80452	*FAC1*	EMBRYONIC FACTOR1	95	839	0	2	172264-164
	F4IVL6	*GRV2*	GRAVITROPISM DEFECTIVE 2	279	2554	0	1	153932-190
	Q94AI7	*TPL*	WUS-INTERACTING PROTEIN 1	124	1131	0	4	308989-63
	Q9SCJ9	*UBP26*	UBIQUITIN-SPECIFIC PROTEASE 26	120	1067	0	7	246668-102
	Q9CAD5	*YDA*	YODA	96	883	1 × 10^–116^	12	58307-332
	Q5YGP7	*PLT2*	PLETHORA 2	62	568	6 × 10^–101^	1	45126-379
	Q6X7J5	*WOX8*	WUSCHEL RELATED HOMEOBOX 8	36	325	3 × 10^–22^	1	63595-317
	Q9LNC5	*MEE5*	MATERNAL EFFECT EMBRYO ARREST 5	110	987	0	1	121247-224
	O23676	*MEE63*	MATERNAL EFFECT EMBRYO ARREST 63	17	150	1 × 10^–91^	1	13540-658
	O23255	*MEE58*	MATERNAL EFFECT EMBRYO ARREST 58	53	485	0	3	250508-99
Flowering	Q0WP44	*HST*	HASTY	133	1202	0	2	367437-36
	Q8W234	*SEU*	SEUSS	96	877	7 × 10^–99^	3	42405-391
	O23624	*FPF1*	FLOWERING PROMOTING FACTOR 1	13	110	9 × 10^–38^	3	394008-25
	Q39110	*GA20OX1*	GIBBERELLIN 20 OXIDASE 1	43	377	2 × 10^–34^	1	35801-423
	P93003	*TFL1*	TERMINAL FLOWER 1	20	177	2 × 10^–12^	3	159790-180
	Q94C32	*MRG1*	MORF RELATED GENE 1	36	320	2 × 10^–65^	1	7219-857
	Q39090	*UFO*	UNUSUAL FLORAL ORGANS	49	442	6 × 10^–107^	2	242870-104
	Q9FIE3	*VIN3*	VERNALIZATION INSENSITIVE 3	69	620	4 × 10^–64^	3	362708-38
	O82166	*AHL21*	AT-HOOK MOTIF NUCLEAR-LOCALIZED PROTEIN 21	29	285	9 × 10^–51^	4	247992-101
	P46639	*KNAT1*	HOMEOBOX PROTEIN KNOTTED-1-LIKE1	46	398	1 × 10^–56^	1	3415-1156
	O22607	*MSI4*	MULTICOPY SUPPRESSOR OF IRA4	56	507	0	1	263958-90
Seed formation	Q9LZV3	*MAN6*	ENDO-BETA-MANNASE 6	51	448	1 × 10^–19^	3	278726-81
	Q67XX3	*JMJ22*	JUMONJI DOMAIN-CONTAINING PROTEIN 22	57	502	0	1	109940-237
Fruit formation	O81788	*WOX13*	WUSCHEL RELATED HOMEOBOX 13	30	268	1 × 10^–48^	4	20354-552
Temperature stress	Q84JU6	*HOS1*	HIGH EXPRESSION OF OSMOTICALLY RESPONSIVE GENES 1	105	927	5 × 10^–174^	5	86304-270
	Q9C5X8	*ABA3*	ABA DEFICIENT 3	92	819	0	3	84009-274
Light stress	Q9FLB1	*PYL5*	PYR1-LIKE 5	23	203	6 × 10^–59^	8	281865-79
	Q9LKI5	*UVH1*	ULTRAVIOLET HYPERSENSITIVE 1	108	956	7 × 10^–158^	3	282136-79
	O22854	*ETFQO*	ELECTRON-TRANSFER FLAVOPROTEIN: UBIQUINONE OXIDOREDUCTASE	70	633	0	3	194802-142
	Q8H2D5	*POLH*	Y-FAMILY DNA POLYMERASE H	65	588	5 × 10^–43^	3	18492-577
	Q6NQ88	*DDB2*	DAMAGED DNA BINDING 2	63	557	0	1	353384-42
Light stress	Q9SJ02	*SEP2*	STRESS ENHANCED PROTEIN 2	22	202	2 × 10^–18^	2	313796-61
Salt stress	Q9LKW9	*SOS1*	SALT OVERLY SENSITIVE 1	127	1146	6 × 10^–18^	1	194815-142
Water Stress	Q9SXL4	*HK1*	HISTIDINE KINASE 1	135	1207	3 × 10^–101^	3	180994-155
Nutrient stress	F4KGN5	*IREG2*	IRON REGULATED 2	57	512	1 × 10^–115^	2	286884-76
	Q8GYE0	*PHF1*	PHOSPHATE TRANSPORTER TRAFFIC FACILITATOR1	44	398	8 × 10^–79^	2	292783-72
	Q1ECJ7	*ARPC3*	ACTIN-RELATED PROTEIN C3	19	174	1 × 10^–40^	9	146923-201
Heavy metal stress	Q9STP8	*ACBP2*	ACYL-COA-BINDING PROTEIN 2	38	354	4 × 10^–61^	1	9531-770
	Q9SZW4	*HMA2*	HEAVY METAL ATPASE 2	102	951	0	9	95501-256
	Q9LQU4	*PCR2*	PLANT CADMIUM RESISTANCE 2	17	152	2 × 10^–25^	3	170997-166
	Q9LUR0	*HEB2*	HYPERSENSITIVE TO EXCESS BORON 2	76	683	1 × 10^–26^	4	355802-41
Oxidative stress	Q8L9Y2	*ELP6*	ELONGATOR PROTEIN 6	29	262	9 × 10^–46^	1	170336-166
	P39207	*NDPK1*	NUCLEOSIDE DIPHOSPHATE KINASE 1	16	149	5 × 10^–81^	3	31293-453
	F4I907	*GLYR2*	GLYOXYLATE REDUCTASE 2	38	358	2 × 10^–129^	2	99357-251
	Q8GXJ4	*GLR3.4*	GLUTAMATE RECEPTOR 3.4	107	959	0	5	113863-232
Defence	Q9SE33	*PBS2*	PPHB SUSCEPTIBLE 2	25	226	1 × 10^–77^	1	165257-173
	P46309	*GSH1*	GLUTAMATE-CYSTEINE LIGASE	59	522	9 × 10^–71^	8	230897-113
	P84634	*DCL4*	DICER-LIKE 4	191	1702	1 × 10^–108^	4	272348-85
	P19172	*CHIA*	CHITINASE A	33	302	2 × 10^–36^	3	23280-519
	Q9SGN6	*NSL1*	NECROTIC SPOTTED LESIONS 1	68	612	6 × 10^–173^	1	283857-78
	Q7XJJ7	*FAAH*	FATTY ACID AMIDE HYDROLASE	66	607	2 × 10^–149^	2	114632-232
	Q9FM96	*PSL4*	PRIORITY IN SWEET LIFE 4	73	647	1 × 10^–64^	1	253585-97
	Q94AQ6	*SRT2*	SIRTUIN 2	42	373	5 × 10^–81^	10	194949-142
	F4JTN2	*LAZ1*	LAZARUS 1	35	304	6 × 10^–150^	10	42999-388
	Q93ZE8	*SDF2*	STROMAL CELL-DERIVED FACTOR 2-LIKE PROTEIN PRECURSOR	24	218	7 × 10^–5^	4	151574-194
	P92948	*CDC5*	CELL DIVISION CYCLE 5	96	844	0	8	32454-444
	Q56XP9	*APD1*	ETHYLENE RESPONSIVE TRANSCRIPTION FACTOR-LIKE PROTEIN	23	196	3 × 10^–37^	4	249981-100

### Protein–protein interactions

The interactome of these 100 proteins of apomixis, vegetative development, sexual reproductive development and response to stress was analysed. They formed a network with 120 nodes and 189 edges with a PPI enrichment *p*-value less than 10^–16^. This indicates that the network has significantly more interactions than expected, i.e. proteins have more interactions with each other than would be expected for a random set of the same size and degree distribution extracted from the genome. The average node degree was 3.15, the average local clustering coefficient of 0.446, and the expected number of edges of 81. The proteins with the highest number of interactions were MULTICOPY SUPPRESSOR OF IRA 4 (MSI4) with 16, and MULTICOPY SUPPRESSOR OF IRA 1 (MSI1) and SLOW WALKER1 (SWA1) with 12 each. Table 3 displays the scores of the different protein–protein interactions showing those with a total score above 0.9.

**Table 3:** Protein–protein interactions of selected proteins from the gametophyte of *D. affinis* with the highest total scores

Protein 1	Protein 2	Neighbourhood interaction	Gene fusion interaction	Co-expression interaction	Experiments interaction	Database interaction	Text mining interaction	Total score
CUL4	DET1	0	0	0.046	0.757	0	0.998	0.999
FIE	MSI1	0	0	0.075	0.939	0.958	0.997	0.999
CUL4	DDB2	0	0	0	0.687	0.841	0.971	0.998
DDB2	DET1	0	0	0	0	0.900	0.913	0.990
AGO1	DCL4	0	0	0.063	0.410	0.313	0.975	0.989
FIE	VIN3	0	0	0	0.050	0.900	0.859	0.985
MSI1	VIN3	0	0	0	0.058	0.900	0.862	0.985
FAS4	RH36	0	0	0.276	0.114	0.932	0.694	0.984
FIE	MSI4	0	0	0.052	0.840	0.809	0.508	0.983
AGO7	DCL4	0	0	0.077	0.410	0.313	0.956	0.981
CUL4	MSI4	0	0	0.048	0.114	0.844	0.865	0.979
MBD9	TAF14B	0	0	0	0	0.900	0.795	0.978
CUL4	MSI1	0	0	0.042	0.345	0.844	0.788	0.976
RH36	SWA1	0	0.005	0.349	0.062	0.808	0.808	0.974
CDC5	CLO	0	0	0.128	0.937	0	0.538	0.972
AGO4	DCL4	0	0	0.058	0.410	0.313	0.934	0.971
AGO10	DCL4	0	0	0.056	0.410	0.313	0.931	0.970
DET1	HY5	0	0	0	0.280	0	0.951	0.963
ALF4	SWA1	0	0	0.500	0.776	0.560	0.224	0.956
FAS4	SWA1	0	0	0.285	0.901	0.350	0.083	0.952
CERK	LCB2a	0.107	0	0	0.062	0	0.931	0.937
HOS1	MSI4	0	0	0.050	0.510	0	0.874	0.936
CDC5	MEE29	0	0.009	0.081	0.889	0.087	0.362	0.932
EDA25	SWA1	0	0	0.388	0.056	0	0.892	0.932
AGO9	DCL4	0	0	0.052	0.410	0.313	0.837	0.929
AGO1	HST1	0	0	0.070	0.047	0.071	0.913	0.918
SE	XCT	0	0	0.069	0	0.911	0	0.913
EDA25	FAS4	0	0	0.911	0.049	0	0	0.912
EIN2	ETO1	0	0	0.065	0	0	0.909	0.911
CUL4	HY5	0	0	0	0	0	0.910	0.910
ALF4	FAS4	0	0	0.526	0.557	0.592	0.065	0.909

According to these scores, few data on neighbourhood, gene fusion, co-occurrence and homology channels were observed. FATTY ACID AMIDE HYDROLASE (FAAH) and PYRIMIDINE 2 (PYD2) highlighted in neighbourhood; CELL DIVISION CYCLE 5 (CDC5) and MATERNAL EFFECT EMBRYO ARREST 29 (MEE29) in gene fusion; and HASTY (HST) and SERRATE (SE) in co-occurrence; while HOMEOBOX PROTEIN KNOTTED-1-LIKE1 and SHOOT MERISTEMLESS (STM) in homology. Co-expression was low, with SLOW WALKER2 (SWA2) and FASCIATED STEM 4 (FAS4) having the strongest interaction. Highest scores came from the experiments, database and the text mining channels: in the first, two, FERTILIZATION-INDEPENDENT ENDOSPERM (FIE) and MULTICOPY SUPPRESSOR OF IRA 1 (MSI1), while in the last, CULLIN 4 (CUL4) and DE-ETIOLATED 1 (DET1).

### Distance trees and protein domain analyses

**Fig. 6:**
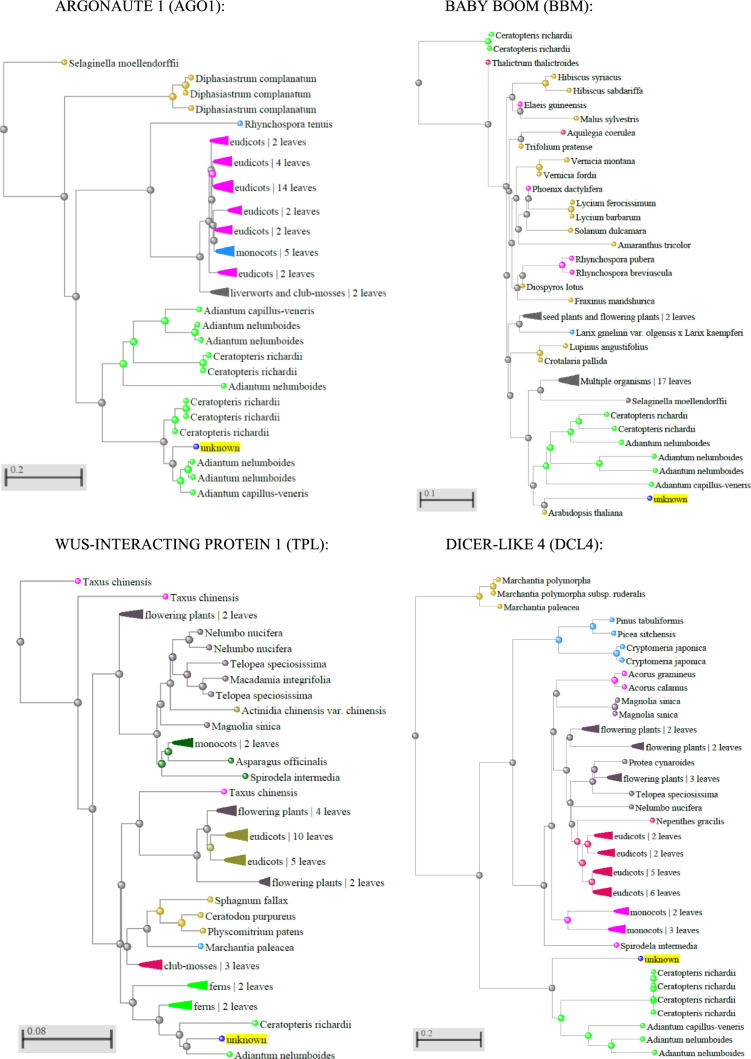
Distance trees of amino acid sequences of selected *D. affinis* gametophyte proteins performed by NCBI Genome Workbench and NCBI BLASTP. *D. affinis* sequence is in yellow. Distance scale represents the number of differences between sequences

To explore the degree of distance existing between homolog *A. thaliana* proteins found in *D. affinis* and proteins from other plants, a distance tree approach is included. Specifically, distance trees of amino acid sequences of the proteins ARGONAUTE 1 (AGO1), WUS-INTERACTING PROTEIN 1 (TPL), BABY BOOM (BBM), and DICER-LIKE 4 (DCL4) are shown in Fig. 6, while those of the other proteins are in Supplementary Fig. 1. It was observed that *D. affinis* AGO1 was similar to those of *A. nelumboides* and *A. capillus-veneris*. In the case of BBM, the *D. affinis* sequence was more similar to that of *A. thaliana*. Continuing with the protein TPL, this showed similarity with that of *A. nelumboides*, and finally, the sequence of DCL4 in *D. affinis* was more similar to those of *C. richardii*, *A. capillus-veneris* and *A. nelumboides*. Comparing distances of the *D. affinis* proteins in all the trees, DCL4 showed the greatest distance from the original node, and CUL4 the shortest distance. A part of the amino acid sequence alignments is shown in Supplementary Fig. 2.

**Fig. 7:**
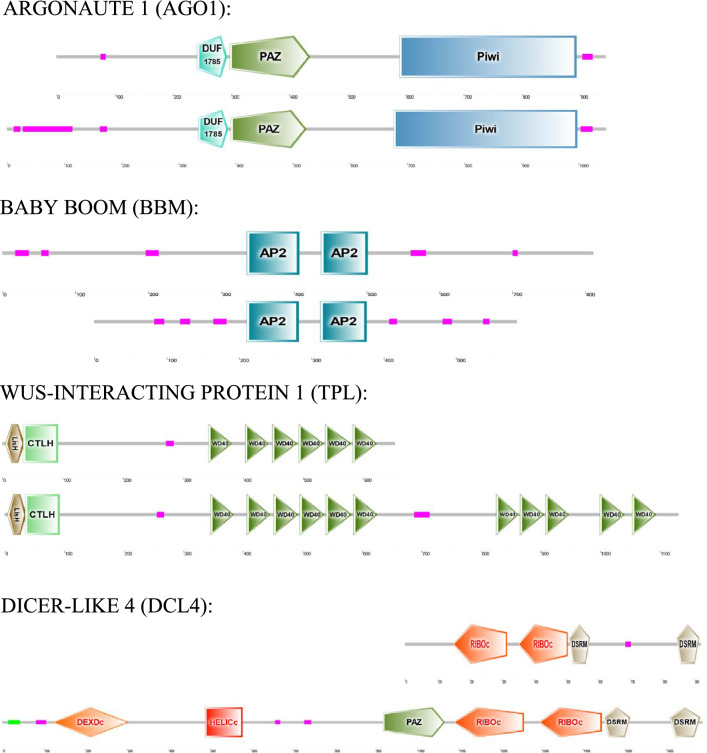
Domains of proteins of *D. affinis* gametophytes above and *A. thaliana* below provided by SMART software

A study of the protein domains was also carried out (Fig. 7 and Supplementary Fig. 3). The proteins AGO1 and BBM in *D. affinis* had the same number and type of domains as in *A. thaliana*. Specifically, AGO1 presented one DUF1785 domain, one Paz domain and one Piwi domain, and BBM showed two AP2 domains. As for the proteins TPL and DCL4, they had in *D. affinis* the same type of domains but in lower amount than in *A. thaliana*. It was noted that TPL had one LisH domain, one CTLH domain and six WD40 domains, which represents five WD40 domains less than in *A. thaliana*. DCL4 showed two RIBOc domains and two DSRM domains, in absence of one DEXDc domain, one HELICc domain and one Paz domain which are also present in *A. thaliana*. The amino acid sequences of these domains are shown in Supplementary Fig. 4.

## Discussion

This work provides detailed information on the transcriptome of *D. affinis* gametophytes contributing to the knowledge acquired on ferns and non-model species, and opening a new avenue to investigate apomixis. As observed in the results, this fern presents numerous homolog proteins related to this type of reproduction. Therefore, *D. affinis* may be a good model to deepen insight in apomixis, providing an opportunity to investigate the molecular mechanisms underlying it and exploiting its potential for applications in plants of economic value.

This research enlarges our previous studies on *D. affinis* and other ferns, such as *Struthiopteris spicant* and *Dryopteris oreades*, focusing on the reproduction that takes place in the gametophyte of this group of plants (Valledor et al. 2014; Grossmann et al. 2017; Wyder et al. 2020; Fernández et al. 2021; Ojosnegros et al. 2022, 2023). According to Wyder et al. (2020), after assembling the RNA-seq reads, a set of 436,707 transcripts was obtained, a higher number than in other fern species, such as *Adiantum flabellulatum*, with 354,228 transcripts (Cai et al. 2022) or *Vandenboschia speciosa*, with 43,139 (Martín-Blázquez et al. 2023). All these studies contribute to enhance the knowledge of the genomes of unsequenced fern species, elusive until recently. Thus, here, the transcripts were blasted against *A. thaliana*. After analysing the resulting total proteins with GENEIOUS, ShinyGO, and STRING platforms, approximately 100 were studied in detail, which represent the core of this discussion. The information was organised around vegetative development, reproductive development and response to abiotic and biotic stress.

### Vegetative development

The development of the gametophyte of *D. affinis* presents different processes in its short life and accordingly, the selected proteins homolog to those of *A. thaliana* were discussed in different processes, such as cell division and differentiation or composition of the cytoskeleton, followed by rudimentary organ development and the necessary growth regulators—whether in their metabolism, transport, or signalling—continuing with whose related to light, such as photomorphogenesis and circadian clock, and ending with senescence and autophagy.

#### Cell division and differentiation, and cytoskeleton

Once spore germination occurs, the initial cell of the gametophyte begins to actively divide and differentiate (Raghavan 1989; Suo et al. 2015a, b). In *D. affinis* some proteins found related to these processes were the homologs of ARGININE-RICH CYCLIN 1 (RCY1), KEULE (KEU), DYNAMIN-LIKE 1B (ADL1B), STOMATAL CYTOKINESIS-DEFECTIVE 1 (SCD1) and *ARABIDOPSIS THALIANA* HOMOLOGUE OF YEAST SPO22 (ATSPO22). Likewise, the cytoskeleton plays an important role in cells by being involved in division, communication, shape determination and extension growth, as well as organelle movement (Goldy and Caillaud 2023). In this regard, the homolog of the *A. thaliana* protein ACTIN DEPOLYMERIZING FACTOR 11 (ADF11) was one example identified.

#### Organ development and growth regulators

**Fig. 8:**
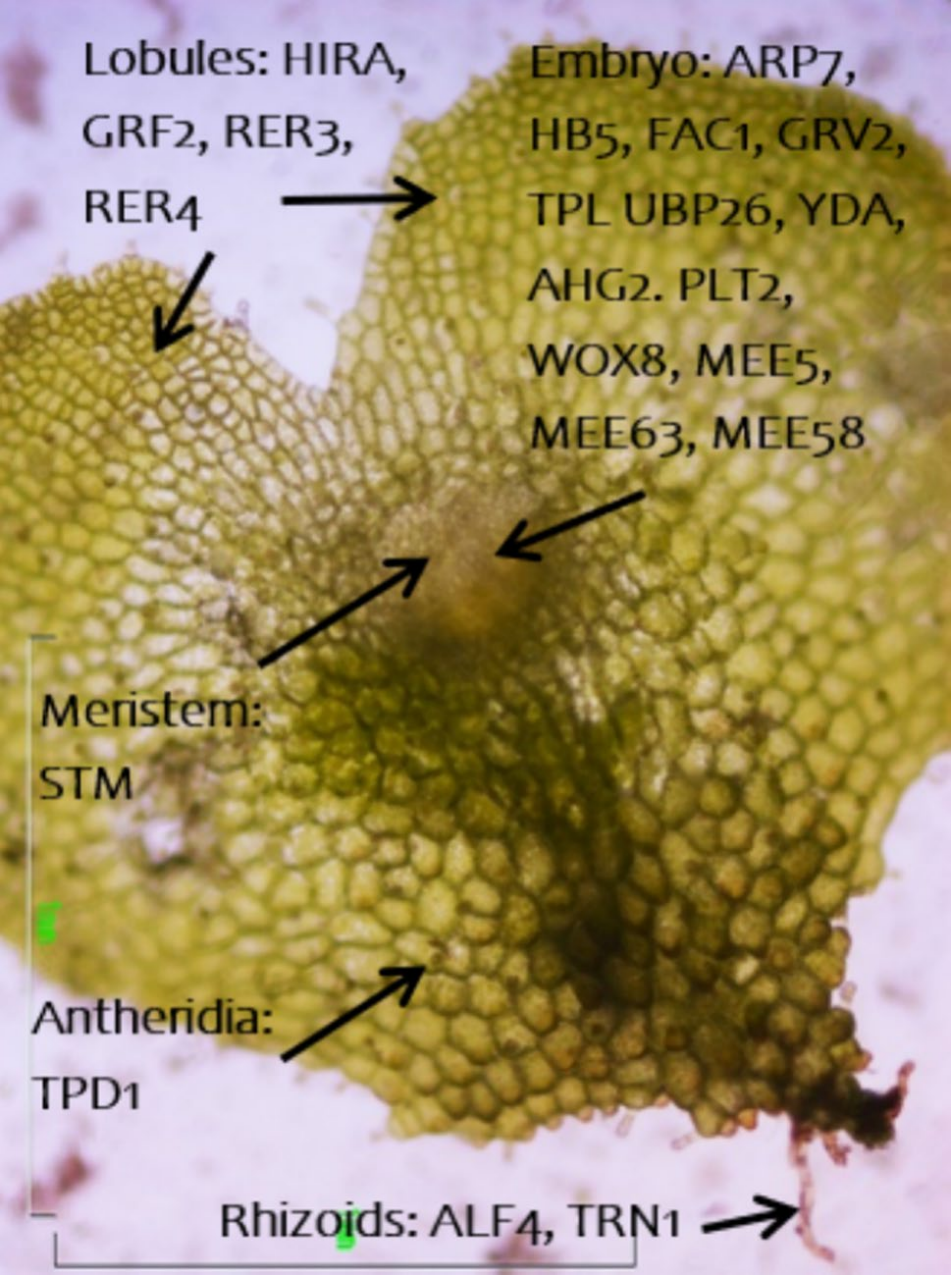
Light microscope image of a two-dimensional gametophyte of *D.* a*ffinis* in which some homolog *A. thaliana* proteins found in the fern transcriptome are hypothetically linked to the corresponding anatomical part

The gametophyte of ferns has rhizoids and photosynthetic flat lobes, instead of roots and leaves found in the sporophyte of ferns and seed plants. However, ferns and seed plants are sister groups (Schneider 2013; PPG I 2016), and thus, they show strong protein homology. This comparison contributes to filling some missing knowledge gaps in plant evolution. In *D. affinis*, the *A. thaliana* homologs of ABERRANT LATERAL ROOT FORMATION 4 (ALF4) and TORNADO 1 (TRN1) were reported, involved in root development in this angiosperm, and HOMOLOG OF HISTONE CHAPERONE HIRA (HIRA), GROWTH-REGULATING FACTOR 2 (GRF2), and RETICULATA-RELATED 3 (RER3) and 4 (RER4) in leaf formation. Meristems are another important structure that in fern gametophyte occurs in three types: apical unicellular, apical multicellular and marginal (Wu et al. 2023), as well as in the sporophyte: root apical, leaf apical and shoot apical (Schneider 2013). In relation to the latter, the homolog of the protein SHOOT MERISTEMLESS (STM), required for the maintenance of undifferentiated cells in shoot and floral meristems in *A. thaliana* (Endrizzi et al. 1996), was identified. Different types of plants have proteins of the KNOX family (STM is member of this family), which participate in the alternation between generations (Pandey et al. 2021). Some proteins found that hypothetically correspond to each anatomical part of a typical two-dimensional gametophyte are depicted in Fig. 8, where we speculate on the existence of common genomic pathways in organ development in both ferns and seed plants, without detracting from the need of further analyses to decipher how genomic fingerprints evolved across taxa and to understand the true functions they have.

The transcriptome of *D. affinis* harboured numerous proteins implicated in the metabolism, transport and signalling of phytohormones, necessary for vegetative and apomictic development, as Menéndez et al. (2006) reflected in their study in in vitro cultures of gametophytes. Related to auxins, the homologs of the *A. thaliana* proteins ENDOPLASMIC RETICULUM AUXIN BINDING PROTEIN 1 (ABP1) and PIN-FORMED 3 (PIN3) were identified. The latter mediates polar transport in *A. thaliana*. It is suggested that auxins, together with cytokinins, are involved in the establishment of polarity (Terceros et al. 2020). In *C. richardii* sporophytes, Aragón-Raygoza et al. (2022) detected PIN3 in the root tip, given the characteristic directional movement of this hormone, and in gametophytes, Withers et al. (2023) demonstrated that proper auxin transport is necessary for meristem positioning and regeneration. As for ethylene, *D. affinis* had several regulators of its signalling pathway: the homolog proteins of ETHYLENE INSENSITIVE 2 (EIN2), ETHYLENE OVERPRODUCER 1 (ETO1), and REVERSION-TO-ETHYLENE SENSITIVITY1 (RTE1). In ferns this hormone is involved in stress defence (Reynolds and John 2004), and exhibits an apogamy-inducing function, as shown by Cordle et al. (2012) in *C. richardii*. Our fern also showed many brassinosteroid-associated protins, such as the homolog of STEROL 1 (STE1). In *C. richardii* Zheng et al. (2022) documented that brassinosteroids hormone promotes almost all growth and developmental processes. Regarding abscisic acid, to which Menéndez et al. (2006) assigned a vegetative growth inhibitory role in gametophytes of *D. affinis*, we found the homologs of GPCR-TYPE G PROTEIN 1 (GTG1) and PYR1-LIKE 5 (PYL5), the latter also detected in gametophytes of *Alsophila spinulosa* (Hong et al. 2022). Finally, on jasmonate, used by ferns to detect and respond to infections caused by bacteria (de Vries et al. 2018), the homolog protein of JASMONATE-ZIM-DOMAIN PROTEIN 4 (JAZ4) was reported. All these hormones were detected in gametophytes of *D. affinis* and its relative *D. oreades* by chromatography and mass spectrometry analyses, performed in our laboratory (Fernández et al. 2021).

#### Photomorphogenesis and circadian clock

Light, through quantity, quality, direction and periodicity, induces morphogenic events in plants throughout their life cycle from germination to development and senescence (Wei et al. 2023). The gametophyte is the first stage to cope with the environment and, although it is able to grow in low light conditions due to efficient morphology and physiology and the presence of unique phytochromes, the way it responds will determine the subsequent growth of the sporophyte (Johnson et al. 2000). Associated with photomorphogenesis in *D. affinis,* the homolog *A. thaliana* proteins of DE-ETIOLATED 1 (DET1), CULLIN 4 (CUL4) and ELONGATED HYPOCOTYL 5 (HY5) were found. Likewise, plant cells contain a circadian clock that interprets environmental signals such as photoperiod to synchronise internal processes with daily and seasonal changes. Related to this, the homologs of XAP5 CIRCADIAN TIMEKEEPER (XCT), TIMEKEEPER LOCUS1 (STIPL1) and ELF4-LIKE 4 (ELF4-L4) were identified in *D. affinis*.

#### Senescence and autophagy

Specifically, as soon as fertilization occurs, the gametophyte is consumed by the new sporophyte, which demands nutrients to sustain its initial development (Haufler et al. 2016). By degrading and reusing cellular components, autophagy helps the plant to respond to environmental and developmental signals while maintaining cellular homeostasis (Cheng et al. 2022). In our fern some several proteins were related to senescence: the homologs of *A. thaliana* MORE AXILLARY BRANCHES 2 (MAX2) and ACCELERATED CELL DEATH 5 (ACD5), or autophagy: AUTOPHAGY 5 (APG5), required in nutrient recycling, proteolysis in chloroplasts and peroxisomes degradation in *A. thaliana* (Yoshimoto et al. 2014), and AUTOPHAGY-RELATED 11 (ATG11), dedicated to the delivery of autophagic vesicles to the vacuole in the same species (Li et al. 2014).

### Reproductive development: sexual and apomixis

The genomic machinery underlying reproductive developmental process remains to be deciphered, especially in species usually ignored as research models, such as most ferns. In this work, several homologs of *A. thaliana* genes involved in sexual and asexual reproduction found in the gametophyte of *D. affinis* were described*.*

#### Gametogenesis and embryogenesis

Gametogenesis is the process of production of male and female gametes (in ferns antherozoids and egg cells) within the sexual organ antheridia and archegonia. Although *D. affinis* is apomictic, male gametogenesis occurs, as antheridia producing antherozoids was reported (Menéndez et al. 2006), therefore, it is not surprising to have found related proteins: the homologs of *A. thaliana* TAPETUM DETERMINANT 1 (TPD1), GAMETE EXPRESSED 3 (GEX3), LONG CHAIN BASE2 (LCB2), F BOX-LIKE17 (FBL17) and FERONIA (FER). TPD1, specifically, is essential in antheridia development in *C. richardii* (Falls 2022). Proteins of female gametogenesis were also annotated, such as the homologs of ARGONAUTE 1 (AGO1) and 7 (AGO7), involved in RNA-mediated post-transcriptional gene silencing in *A. thaliana* (Cuperus et al. 2010), SLOW WALKER 1 (SWA1), 2 (SWA2) and 3 (SWA3), whose mutants result in slow and delayed mitosis progression in the same species (Li et al. 2009), or CHROMATIN-REMODELING PROTEIN 11 (CHR11).

The gametophyte of *D. affinis* is involved in embryo formation, so it was logical to find embryogenesis-associated proteins: the homologs of WUSCHEL RELATED HOMEOBOX 8 (WOX8), whose family controls cell proliferation of several meristematic domains in *C. richardii* (Youngstrom et al. 2022), and is essential for patterning, basal development after fertilisation, cell division and proliferation in *A. thaliana* (Wu et al. 2007), HOMEOBOX PROTEIN 5 (HB5), EMBRYONIC FACTOR 1 (FAC1), GRAVITROPISM DEFECTIVE 2 (GRV2) and WUS-INTERACTING PROTEIN 1 (TPL), a repressor of root promoter genes during this process also in *A. thaliana* (Wei et al. 2015). Others were the homologs of YODA (YDA), which regulates first cell fate decisions in the early embryo (Lukowitz et al. 2004), UBIQUITIN-SPECIFIC PROTEASE 26 (UBP26), a heterochromatin silencer whose loss of function mutants arrests embryogenesis (Sridhar et al. 2007) and PLETHORA 2 (PLT2), involved in stem cell niche establishment (Smith and Long 2010). These results are in agreement with other studies, such as Youngstrom et al. (2019) and Aragón-Raygoza et al. (2022), which detected PLT2 in *C. richardii*. Other important homolog proteins reported were MATERNAL EFFECT EMBRYO ARREST 5 (MEE5), 63 (MEE63) and 58 (MEE58).

#### Flowering, seed and fruit formation

The ferns do not form flowers, but homolog proteins of *A. thaliana* involved in flowering were noted. The fact that *D. affinis* always reproduces asexually might make this finding surprising, but this is not an isolated case in ferns, as Cordle et al. (2012) also reported genes related to flowering in *C. richardii*. Mutants of these genes cannot reproduce correctly by apogamy, as indicated by Marchant et al. (2022). Some of these proteins are involved in *A. thaliana* in the transition from vegetative to reproductive phase, such as HASTY (HST), FLOWERING PROMOTING FACTOR 1 (FPF1), GIBBERELLIN 20 OXIDASE 1 (GA20OX1) or KNOTTED-LIKE FROM *ARABIDOPSIS THALIANA* (KNAT1). This last belongs to the KNOX family of class I, which in the sporophyte of *C. richardii* is involved in the meristem development in a similar manner to seed plants (Sano et al. 2005). In *A. thaliana* KNAT1 keeps the cells in an undifferentiated state until the shoot apex completes the transition (Kim et al. 2003). Also the protein GLYCINE-RICH PROTEIN 2B (GRP2B), which in addition to transition, regulates flower development. Other homolog proteins were MULTICOPY SUPPRESSOR OF IRA 4 (MSI4), which promotes flowering in *A. thaliana* (Chowdhury et al. 2020), SEUSS (SEU), TERMINAL FLOWER 1 (TFL), UNUSUAL FLORAL ORGANS (UFO), involved in histone acetylation of flowering target genes, and VERNALIZATION INSENSITIVE 3 (VIN3), required for vernalization and cold treatment in seed plants. Also worth mentioning is the finding of the homologs of AT-HOOK MOTIF NUCLEAR-LOCALIZED PROTEIN 21 (AHL21), which binds to the promoter of the *FT* gene to regulate flowering in *A. thaliana* (Yun et al. 2012), and MORF RELATED GENE 1 (MRG1), which increases the transcriptional levels of the *FLC* and *FT* genes in the same species (Bu et al. 2014). Marchant et al. (2022) studied homologous *FT* genes in *C. richardii* and suggested that they may be associated with spore formation in ferns, a process that may predate their function of flower regulation in angiosperms. Although *D. affinis* is not a seed and fruit plant, associated homolog proteins were found, such as ENDO-β-MANNASE 6 (MAN6) and JUMONJI DOMAIN-CONTAINING PROTEIN 22 (JMJ22) in seed germination in *A. thaliana*, and WUSCHEL RELATED HOMEOBOX 13 (WOX13), which, although in *A. thaliana* promotes plum development and inhibits fruit dehiscence (Ikeuchi et al. 2022), in *C. richardii* is related to growth in the meristems (Youngstrom et al. 2019), and its function in *D. affinis* is probably close to this.

#### Asexual reproduction

The gametophyte of apomictic ferns can shed some light on the understanding of the molecular basis of apomixis, and open a way to broaden its agricultural applications, long dreamed by researchers (Spillane et al. 2004). In early studies, Nogler (1984) showed that apomixis is genetically controlled. Later, Savidan et al. (2001) pointed out that it can be affected by genetic modifiers or environmental conditions, and in recent years, Grimanelli (2012) considered that epigenetic mechanisms, such as DNA methylation or gene silencing, were involved and inhibited genes for sexual reproduction, a hypothesis that is being increasingly supported, as mutations in some epigenetic regulators promote apomixis in sexual plants (Hernández-Lagana et al. 2016). Likewise, RNA helicases, responsible for unwinding the secondary structure of RNA, and transcription factors, required for gene expression, could also play a possible role. Whatever the mechanism, asexual reproduction has emerged in plants due to mutations in sexual reproduction genes to prevent infertility (Xu et al. 2022).

Regarding methylation, Schmidt (2020) hypothesised that apomixis would have overlapped sexual reproduction through epigenetic control mechanisms. *D. affinis* has several related homolog *A. thaliana* proteins that in the angiosperm, by methylating the genome, prevent the transcription of genes not required at certain stages of reproductive development: FERTILIZATION-INDEPENDENT ENDOSPERM (FIE) and MULTICOPY SUPPRESSOR OF IRA1 (MSI1). They are part of a complex called Repressive Complex 2 (PRC2). Both repress the development of an unfertilised embryo and endosperm in *A. thaliana,* so their mutants can initiate parthenogenesis (Ohad et al. 1999; Guitton and Berger 2005; Weinhofer et al. 2010).

Gene silencing is the next possible mechanism that would explain apomixis in ferns, as suggested by Barcaccia and Albertini (2013). In *D. affinis* some gene silencing proteins found were the homologs of ARGONAUTE 4 (AGO4), AGO9 and AGO10, the first one involved during transcription and the rest afterwards. Further studies pointed in the direction of a possible link between gene silencing and apomixis (Olmedo-Monfil et al. 2010; Rabiger et al. 2016), as *A. thaliana* and *Hieracium* mutants for ARGONAUTE proteins showed apospory. Likewise, other reports have suggested a role of small interfering RNA in apomixis (Grimanelli 2012). On the other hand, AGO10 controls by gene silencing the activity of the shoot apical meristem in *A. thaliana*, as does SERRATE (SE) (Dong et al. 2008), another protein whose homolog is reported in the present study. Both could be candidates for apogamy, as suggested by Grossmann et al. (2017), as they could be involved in the development of the apogamous embryo meristem or in other yet unknown functions in the switch between sexual and asexual reproduction.

Continuing with RNA helicases, the large number of *A. thaliana* homologs found in *D. affinis* is worth mentioning. Some of them are involved in gametogenesis, so it is speculated that they might have a role in apomixis. FASCIATE STEM 4 (FAS4) was one of them. It was found differentially up-regulated in the sexual and apomictic angiosperm *Boechera* (Schmidt 2020). Others were the homologs of SLOW WALKER1 (SWA1), 2 (SWA2) and 3 (SWA3), all required in female gametogenesis. SWA1, in particular, is essential in nuclear division and mitosis organization in *A. thaliana* (Shi et al. 2005), SWA2 in ribosome export (Liu et al. 2010), and SWA3 in ribosomal biogenesis, being, together with RNA processing, essential for cell cycle progression (Liu et al. 2010). It is likely that SWA3 belongs to the same ribosome-associated pathway as FAS4, controlling aspects of mitosis in gametogenesis, as mutants of *SWA3* gene were observed to show the same defects as those of *FAS4* gene (Liu et al. 2010; Schmidt 2020). However, more studies are needed to better understand the possible role of these proteins in apomixis. The last RNA helicase we want to highlight is the homolog of MATERNAL EFFECT EMBRYO ARREST 29 (MEE29), essential in pre-mRNA splicing in embryogenesis of *Hypericum perforatum* (Barcaccia and Albertini 2013).

Transcription factors may also be related to apomixis. One homolog of *A. thaliana* detected in *D. affinis* was BABY BOOM (BBM). The heterologous expression of BBM from *Brassica napus* (BnBBM) in *C. richardii* gametophytes promoted the spontaneous production of apogamous sporophytes (Bui et al. 2017). The same researchers identified a protein in that fern whose overexpression produced the same result: CrANT (the homolog of ANT of *A. thaliana*). These results showed genetic conservation between apogamy in ferns and somatic embryogenesis in angiosperms (Bui et al. 2017), as BBM induces parthenogenesis and somatic embryogenesis in *Brassica napus* and *A. thaliana* (Boutilier et al. 2002; Schmidt 2020). AGAMOUS-LIKE 62 (AGL62) and 6 (AGL6) are other transcription factors found, the first promotes cellularization in early endosperm development in *A. thaliana* (Kang et al. 2008), and the second is involved in megasporogenesis in the apomictic and sexual angiosperm *Brachiaria brizantha* (Guimaraes et al. 2013). A deeper study of how they influence the *D. affinis* genome will provide a better understanding of mechanisms underlying apomixis. Finally, other proteins identified with a possible role in apomixis were the homologs of SWITCH1 (SWI1), required in male and female gamete formation and chromatin remodelling in *A. thaliana* (Siddiqi et al. 2000), and BRI1-ASSOCIATED RECEPTOR KINASE (BAK1)*,* involved in somatic embryogenesis and embryo sac development in *Poa pratensis* (Albertini et al. 2005).

### Response to abiotic and biotic stress

The gametophyte, despite its delicate appearance, can be robust in response to stress (Krieg and Chambers 2022), yet few studies have focused on it. Our transcriptome data revealed the expression in *D. affinis* of a large group of *A. thaliana* homolog genes that allow the gametophyte to cope with environmental threats imposed by variations in temperature, light, salinity, water, nutrients or heavy metals, in addition to biotic attacks, which are discussed below.

#### Temperature and light stress

Temperature variations can stress plants and overcome their ability to recover. In *D. affinis* cold stress-related proteins were found, such as the homologs of HIGH EXPRESSION OF OSMOTICALLY RESPONSIVE GENES 1 (HOS1), and ABA DEFICIENT 3 (ABA3). A large number of heat-shock *A. thaliana* homolog proteins were also identified, revealing the rich machinery of our fern to cope with changing conditions. As for the response to light stress, while sporophyte increases light uptake by producing large fronds and growing higher, the fern gametophyte can only adapt its photosynthetic machinery to achieve a positive carbon balance (Krieg and Chambers 2022). Several proteins found to be involved were the homologs of ELECTRON-TRANSFER FLAVOPROTEIN: UBIQUINONE OXIDOREDUCTASE (ETFQO), ULTRAVIOLET HYPERSENSITIVE 1 (UVH1), Y-FAMILY DNA POLYMERASE H (POLH), DAMAGED DNA BINDING 2 (DDB2), the last three avoiding damage caused by ultraviolet light, and STRESS ENHANCED PROTEIN 2 (SEP2), involved in non-photochemical quenching by minimising the generation of oxidative molecules.

#### Salt, water and nutrient stress

Like other stress factors, salinity affects the growth of gametophyte. A high amount of salinity in the substrate decreases its size and affects gametangia development (Pangua et al. 2009). *D. affinis* contained proteins to overcome salt stress and maintain ionic homeostasis, such as the *A. thaliana* homolog protein SALT OVERLY SENSITIVE 1 (SOS1). In terms of water stress, *D. affinis* was equipped to fight water scarcity and leakage damage with proteins such as the homolog osmo-sensor of water HISTIDINE KINASE 1 (HK1), being striking that the gametophyte, despite having limited water storage capacity and a rudimentary cuticle, has developed some degree of tolerance to desiccation, preventing its survival from being compromised. On the other hand, in Wyder et al. (2020), to obtain firstly only one-dimensional and not two-dimensional gametophytes, *D. affinis* spores were overcrowded causing nutrient stress, consistent with obtaining proteins such as the homologs of IRON REGULATED 2 (IREG2) and PHOSPHATE TRANSPORTER TRAFFIC FACILITATOR1 (PHF1), involved in the response to iron and phosphate shortage respectively. Another annotated stress protein was the homolog of ACTIN-RELATED PROTEIN C3 (ARPC3), required after exposure to extreme changes in the environment, also found in gametophytes of *Diplazium maximum* (Sareen et al. 2019).

#### Heavy metal and oxidative stress

Heavy metals are usually harmful for plants (Pichhode and Nikhil 2016). However, some ferns are able to accumulate them, harbouring proteins that could be good candidates for the design of stress-tolerant crops (Chen 2022). The gametophyte of *D. affinis* showed proteins to respond to plumb, like the *A. thaliana* homolog of ACYL-COA-BINDING PROTEIN 2 (ACBP2), cadmium, such as HEAVY METAL ATPASE 2 (HMA2) and PLANT CADMIUM RESISTANCE 2 (PCR2), and boron, with HYPERSENSITIVE TO EXCESS BORON 2 (HEB2). In relation to oxidative stress, we mention some of them: the homologs of ELONGATOR PROTEIN 6 (ELP6), NUCLEOSIDE DIPHOSPHATE KINASE 1 (NDPK1) and GLYOXYLATE REDUCTASE 2 (GLYR2). Another homolog protein identified was GLUTAMATE RECEPTOR 3.4 (GLR3.4), which is required for the rapid transmission of calcium-based signals.

#### Defence

Regarding defence mechanisms in ferns, in *D. affinis*, a long list of homolog *A. thaliana* proteins related to defence against other organisms was annotated, either intervening in plant innate immunity, induced against pathogen-associated molecular patterns, or in programmed cell death through hypersensitive response: the homologs of PPHB SUSCEPTIBLE 2 (PBS2), GLUTAMATE-CYSTEINE LIGASE (GSH1), DICER-LIKE 4 (DCL4), CHITINASE A (CHIA), NECROTIC SPOTTED LESIONS 1 (NSL1), FATTY ACID AMIDE HYDROLASE (FAAH), PRIORITY IN SWEET LIFE 4 (PSL4), STROMAL CELL-DERIVED FACTOR 2-LIKE PROTEIN PRECURSOR (SDF2), SIRTUIN 2 (SRT2), LAZARUS 1 (LAZ1) and CELL DIVISION CYCLE 5 (CDC5). Likewise, an ethylene-responsive protein required for systemic acquired resistance was found: the homolog of ETHYLENE RESPONSIVE TRANSCRIPTION FACTOR-LIKE PROTEIN (APD1). This type of protein represents a key regulatory centre in plant stress response, as it is involved in hormone signalling and oxidation–reduction reactions (Muller and Munné-Bosch 2015).

### Protein–protein interactions

MSI4, MSI1, and SWA1 were the proteins with the most interactions, probably because they are involved in many processes of vegetative and reproductive development (Guitton and Berger 2005; Shi et al. 2005; Chowdhury et al. 2020; Schmidt 2020). By type of interaction, the strongest in neighbourhood was between FAAH, required in defence, and PYD2, in nitrogen recycling from nucleobases, as their genes are close together in the genome (Khan et al. 2017). In gene fusion, the strongest was between CDC5, essential for innate immunity, and MEE29, in embryogenesis, because at least in the fungus *Pneumocystis jirovecii* orthologous protein-coding genes are fused into an only gene (Barcaccia and Albertini 2013). The highest co-occurrence was between HST, involved in the vegetative to reproductive phase transition, and SE, which regulates leaf polarity, because their genomes have evolved similarly over time (Dong et al. 2008). In terms of homology, AGO1 and AGO7, involved in RNA-mediated post-transcriptional gene silencing, showed the highest values, as they have the same common ancestor and similar sequences (Cuperus et al. 2010). The strongest co-expression was between SWA2 and FAS4, as their expression patterns are similar. Both are required for female gametogenesis and its role in apomixis has been proposed (Li et al. 2009; Schmidt 2020). The next studied evidences were experiments, i.e. proteins that showed chemical, physical or genetic interactions in laboratory experiments; and databases, i.e. proteins found in the same databases. In our work, the strongest interactions in these channels were between FIE and MSI1. Both repress parthenogenesis and are also considered candidates for apomixis (Fei et al. 2019). Finally, in text mining the strongest interaction was between CUL4 and DET1, required in photomorphogenesis, because they are mentioned in the same PubMed abstract or articles from an internal selection of the STRING software (Nassrallah et al. 2018).

### Distance trees and protein domains analyses

Ferns diverged from seed plants about 400 million years ago (Pryer et al. 2001), constituting a clade that can provide much information. To study which species have the most similar sequences to proteins found in *D. affinis* homolog to those of *A. thaliana*, distance trees analyses with amino acid sequences were addressed. In almost all the proteins analysed, concretely AGO1, TPL, and DCL4, the sequence of *D. affinis* was more similar to those of *A. nelumboides*, *A. capillus-veneris* and *C. richardii*. BBM was different, as the sequence in *D. affinis* showed more similarity to that in *A. thaliana*, sharing both the previous node with *A. nelumboides*, *A. capillus-veneris* and *C. richardii*. These last three species, together with *D. affinis,* are ferns and conserved similar sequences in these proteins. As for the comparison of distances in all the trees, DCL4 showed the greatest distance from the original node, and CUL4 the shortest, which means that the first protein diverged more, and the second less.

Regarding the analysis of protein domains, as said in the results section, in AGO1 and BBM the same number and type of domains were conserved in both species studied. The Paz and Piwi domains are found in all ARGONAUTE proteins, with the Piwi domain having a role in epigenetic regulation of the transcriptome (De Storme and Geelen 2013). BBM belongs to the APETALA 2/ETHYLENE-RESPONSIVE ELEMENT BINDING FACTOR (AP2/ERF) family, which has three classes of genes depending on the number of AP2 domains present. BBM always has two AP2 domains (Bui et al. 2017). In contrast, TPL and DCL4 conserved in *D. affinis* the same type of domains but in smaller numbers than in *A. thaliana*. The WD40 domains present in TPL function as a platform for interaction with other proteins or DNA (Xu and Min 2011). As for DCL4, in *D. affinis* it presented two RIBOc and two DSRM domains, in the absence of one DEXDc, one HELICc and one Paz domain that *A. thaliana* has. The DEXDc and HELICc domains are responsible for recognising pathogenic virus RNA in plant infections (Yao et al. 2022).

In conclusion, an exhaustive study of the transcriptome obtained from the apomictic gametophyte of the fern *D. affinis* using RNA-seq has proven to be a very useful methodology to annotate thousands of genes, categorise them and discuss their role in plant development. Although the gametophyte is a simple individual, and ferns lack complex reproductive organs like flowers, the large number of *A. thaliana* homolog genes found that are associated with reproduction, including gametogenesis, embryogenesis, flowering and seed development, is striking, with some of them being possible candidates for apomixis. In addition, homolog proteins associated with vegetative development, as well as with response to biotic and abiotic stress were also pointed to. Protein–protein interactions were provided by String platform and came from experiments, databases and text mining channels, and were led by MSI4 with 16 and MSI1 and SWA1 with 12. Regarding distance trees analysis, DCL4 was the most distant to the common ancestor. All this knowledge provides new information that opens new research lines for understanding the molecular basis of gametophytic development, including apomixis.

## Supplementary Information

Below is the link to the electronic supplementary material.

Supplementary file1 (DOCX 1232 KB)

Supplementary file2 (DOCX 743 KB)

Supplementary file3 (DOCX 410 KB)

Supplementary file4 (DOCX 18 KB)

Supplementary file5 (DOCX 127 KB)

## Data Availability

The de novo assembly of the transcriptome of the *D. affinis* gametophyte in fasta format and the transcriptome annotation are available in the Zenodo research data repository (www.zenodo.org) with the identifier 10.5281/zenodo.1040330. Specifically, the sequences studied in this work are provided in Supplementary Table 1.
