# Supplementary material for: Transcriptomic analyses in the gametophytes of the apomictic fern Dryopteris affinis
Source: Planta. 2024 Oct 2;260(5):111. doi: 10.1007/s00425-024-04540-w (PMC11447071; doi:10.1007/s00425-024-04540-w)
Supplement: Supplementary file 1 — Supplementary file1 (DOCX 1232 KB) [file 425_2024_4540_MOESM1_ESM.docx]

**Article title**: Transcriptomic analyses in the gametophytes of the apomictical fern *Dryopteris affinis*.

**Journal name**: Planta.

**Authors names**: Sara Ojosnegros^1^, José Manuel Alvarez^1^, Valeria Gagliardini^2^, Luis G. Quintanilla^3^, Ueli Grossniklaus^2^, and Helena Fernández^1^

**Affiliations**:

^1^Area of Plant Physiology, Department of Organisms and Systems Biology, University of Oviedo, 33071 Oviedo, Spain; uo286037@uniovi.es (S.O.); alvarezmanuel@uniovi.es (J.M.A.); [fernandezelena@uniovi.es](mailto:fernandezelena@uniovi.es) (H.F.)

^2^Department of Plant and Microbial Biology & Zurich-Basel Plant Science Center, University of Zurich, 8008 Zurich, Switzerland; vgagliar@botinst.uzh.ch (V.G.); grossnik@botinst.uzh.ch (U.G.)

^3^Global Change Research Institute, University Rey Juan Carlos, 28933 Móstoles, Spain; luis.quintanilla@urjc.es

**E-mail address of the corresponding author**: [fernandezelena@uniovi.es](mailto:fernandezelena@uniovi.es)


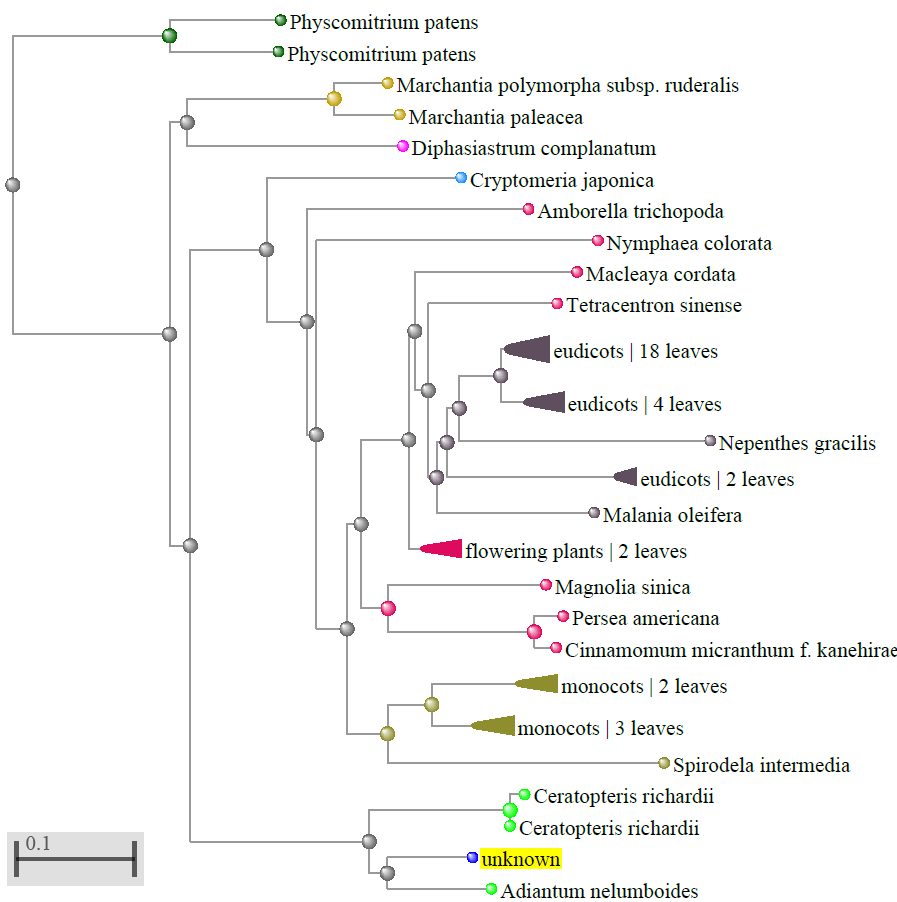

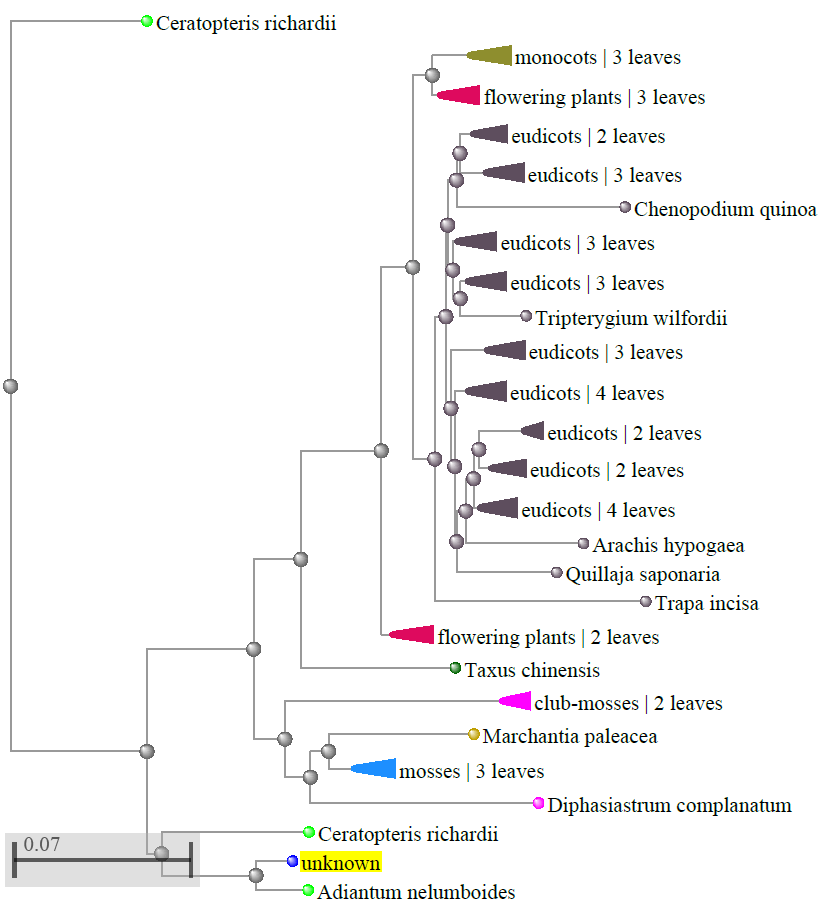
CULLIN 4 (CUL4): TIMEKEEPER LOCUS1 (STIPL1):


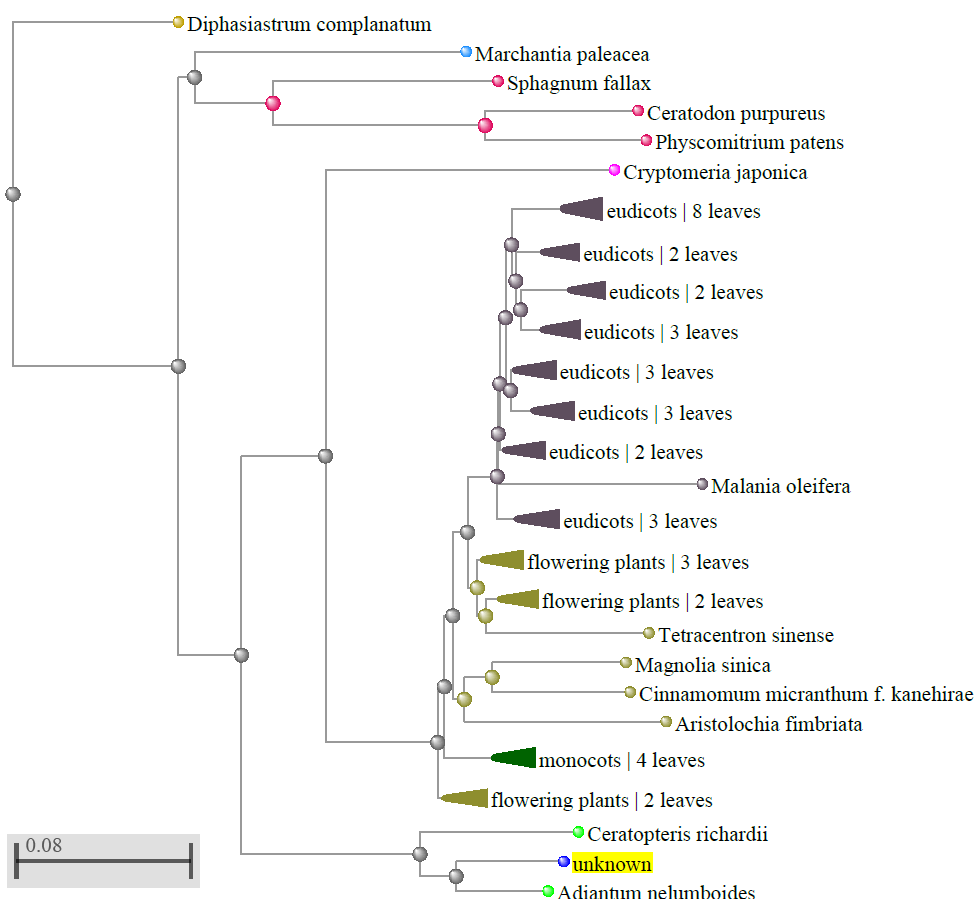

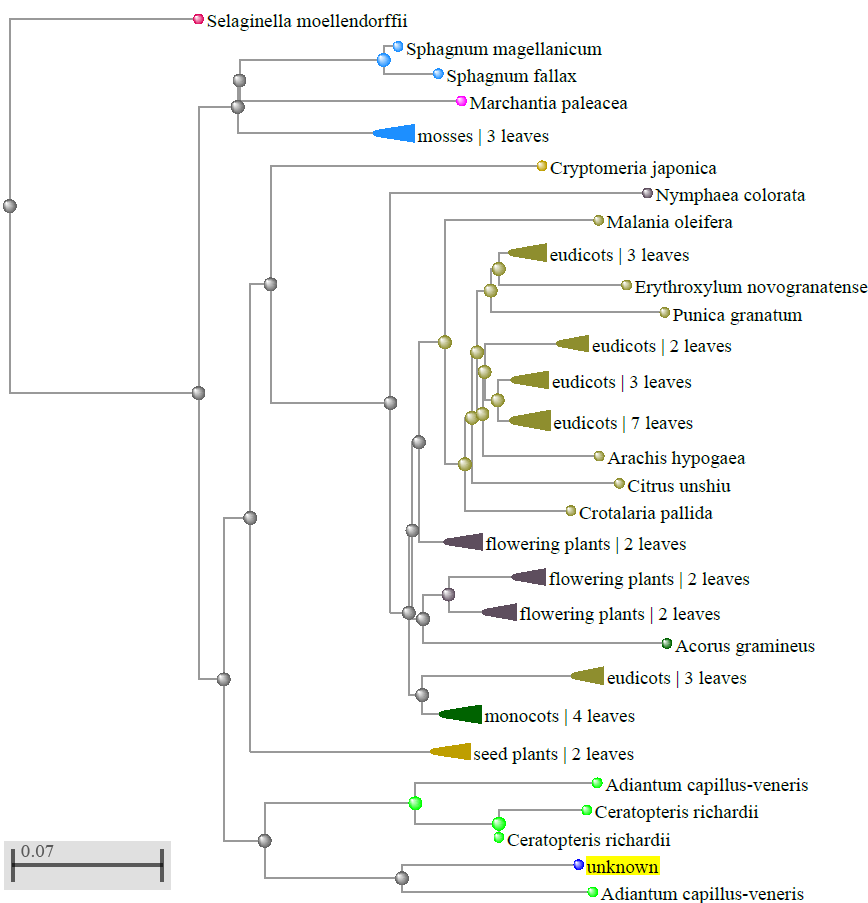
EMBRYONIC FACTOR1 (FAC1): GRAVITROPISM DEFECTIVE 2 (GRV2):


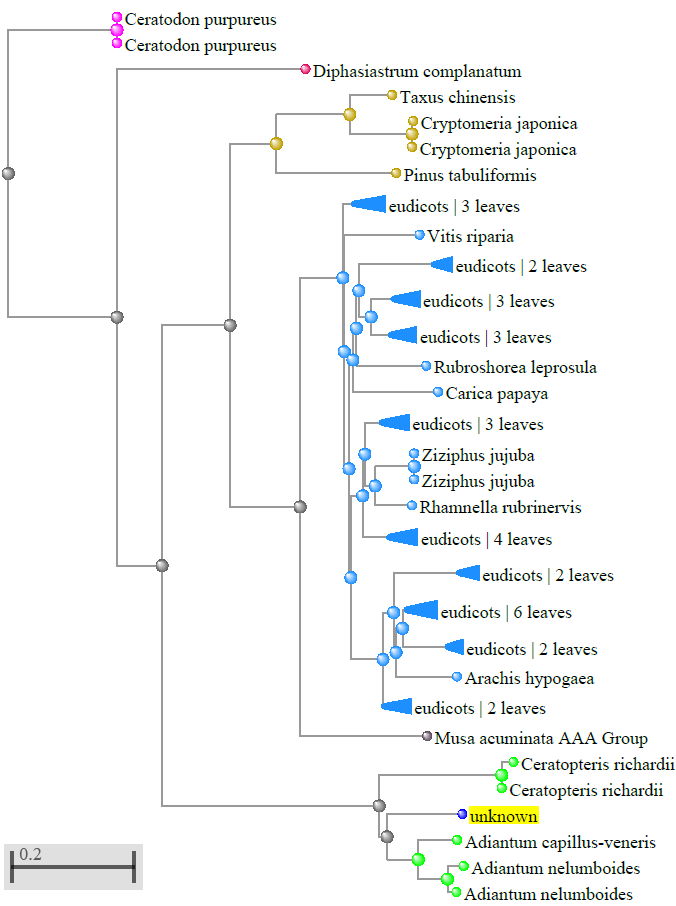

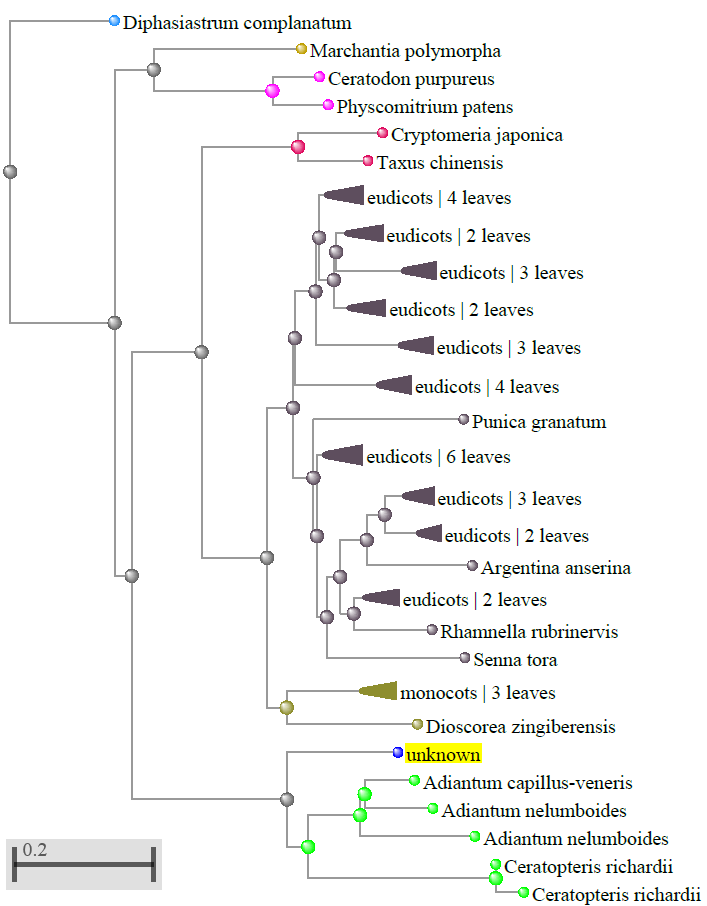
UBIQUITIN-SPECIFIC PROTEASE 26 (UBP26): HASTY (HST):

GLIOMAS 41 (GAS41): SEUSS (SEU):


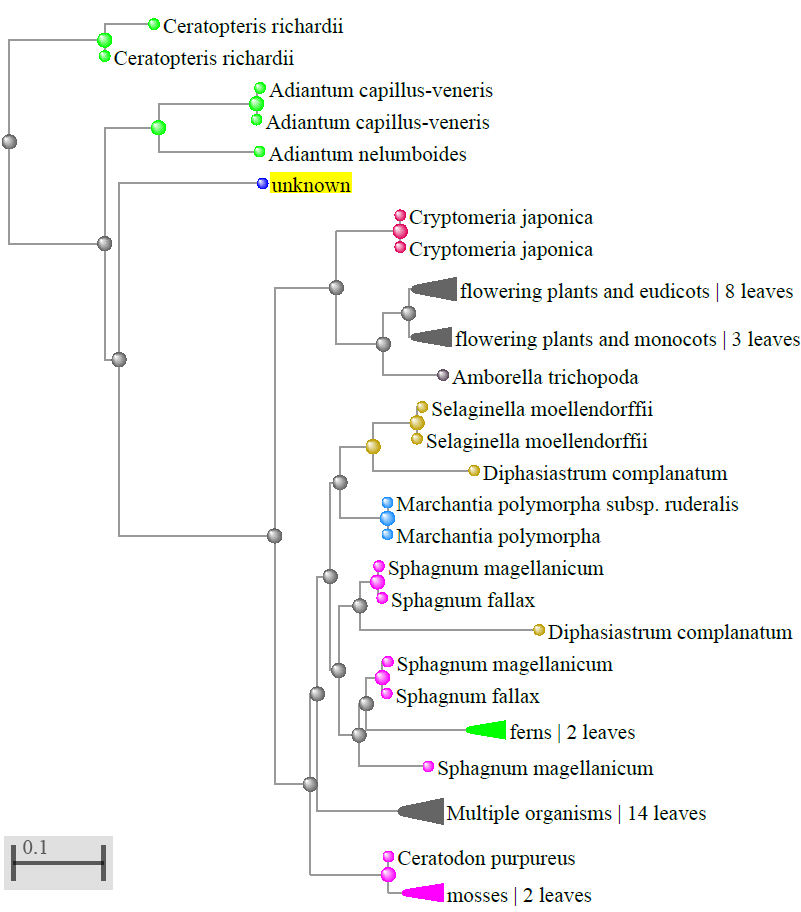

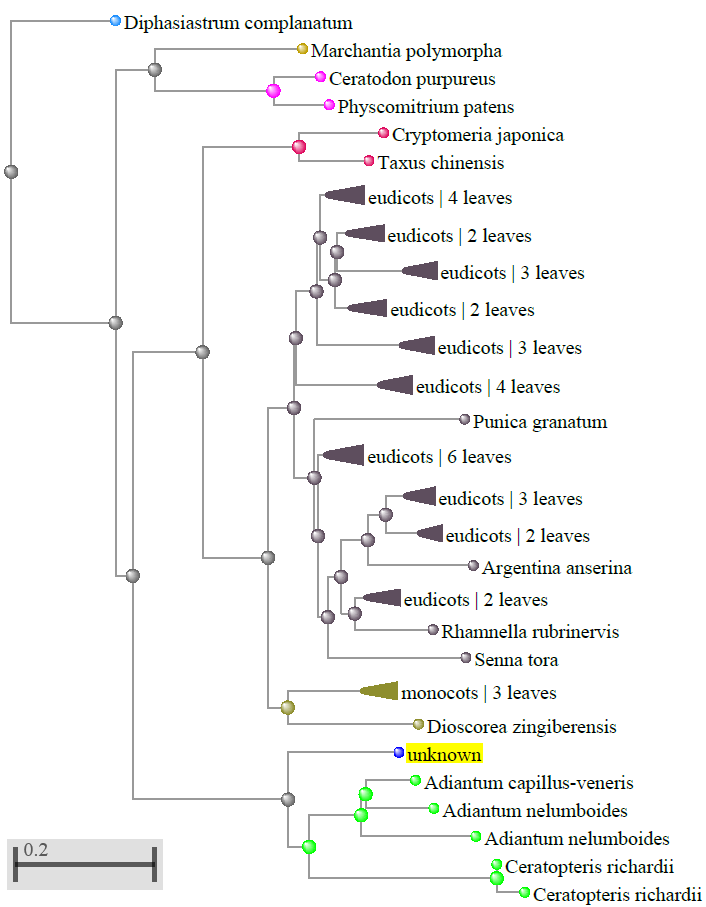


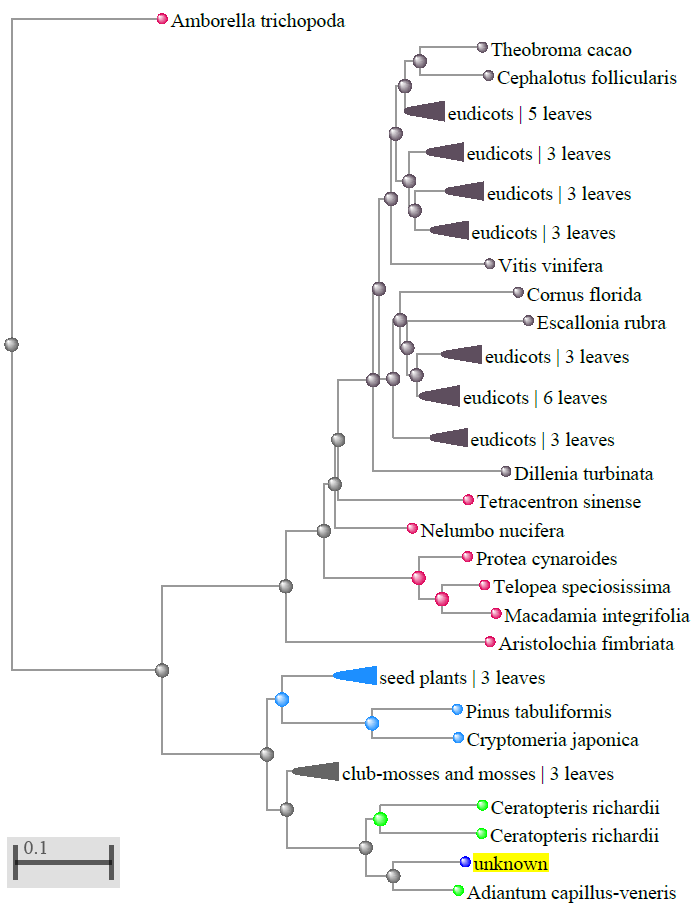

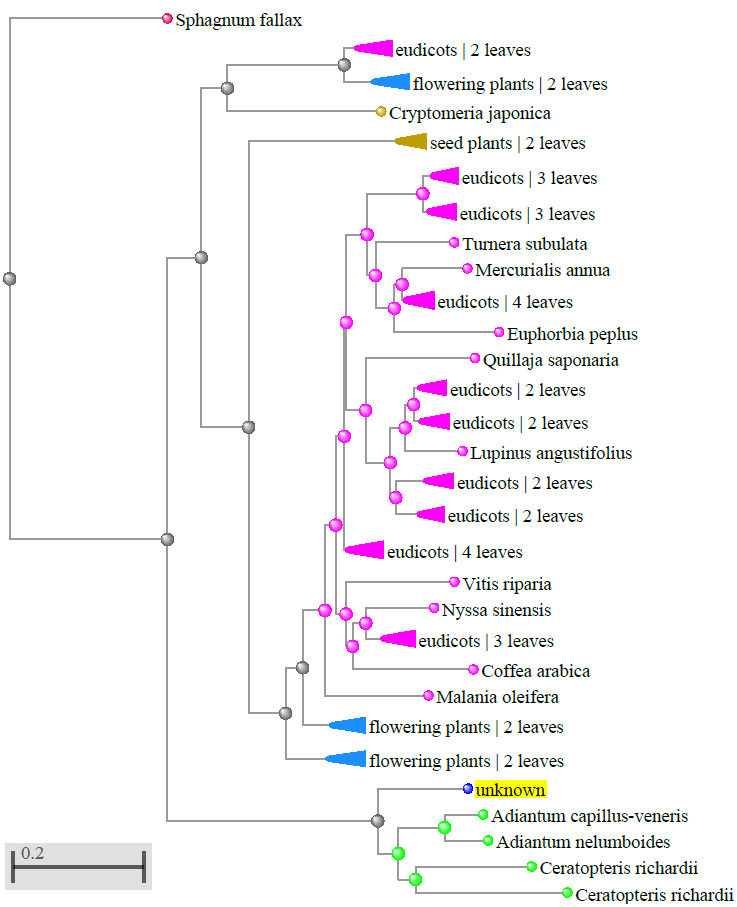
UNUSUAL FLORAL ORGANS (UFO): VERNALIZATION INSENSITIVE 3 (VIN3):

ULTRAVIOLET HYPERSENSITIVE 1 (UVH1):

EXPRESSION OF OSMOTICALLY RESPONSIVE GENES 1 (HOS1):


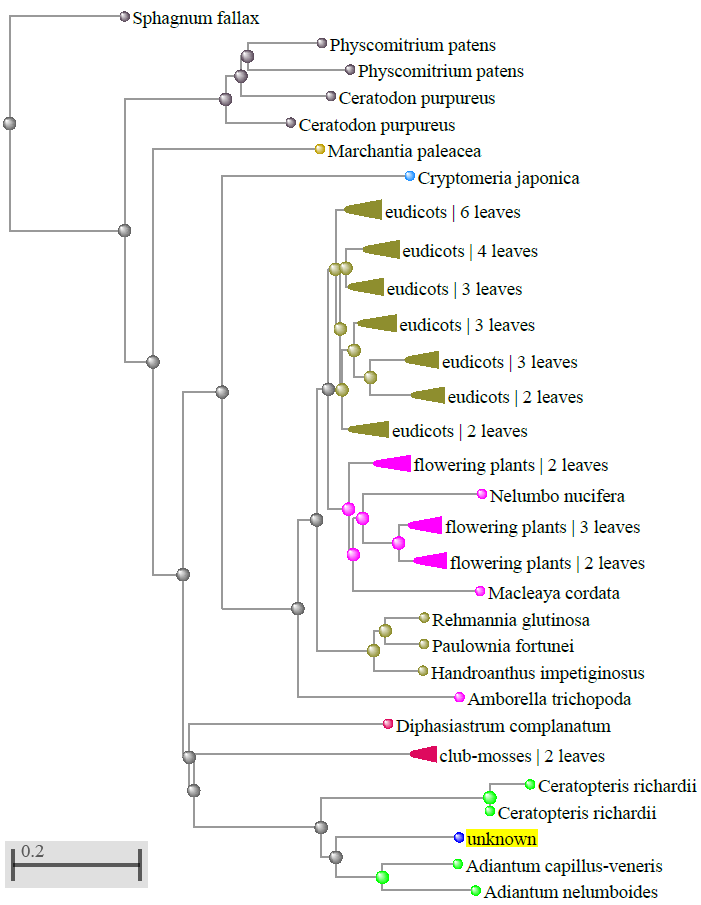


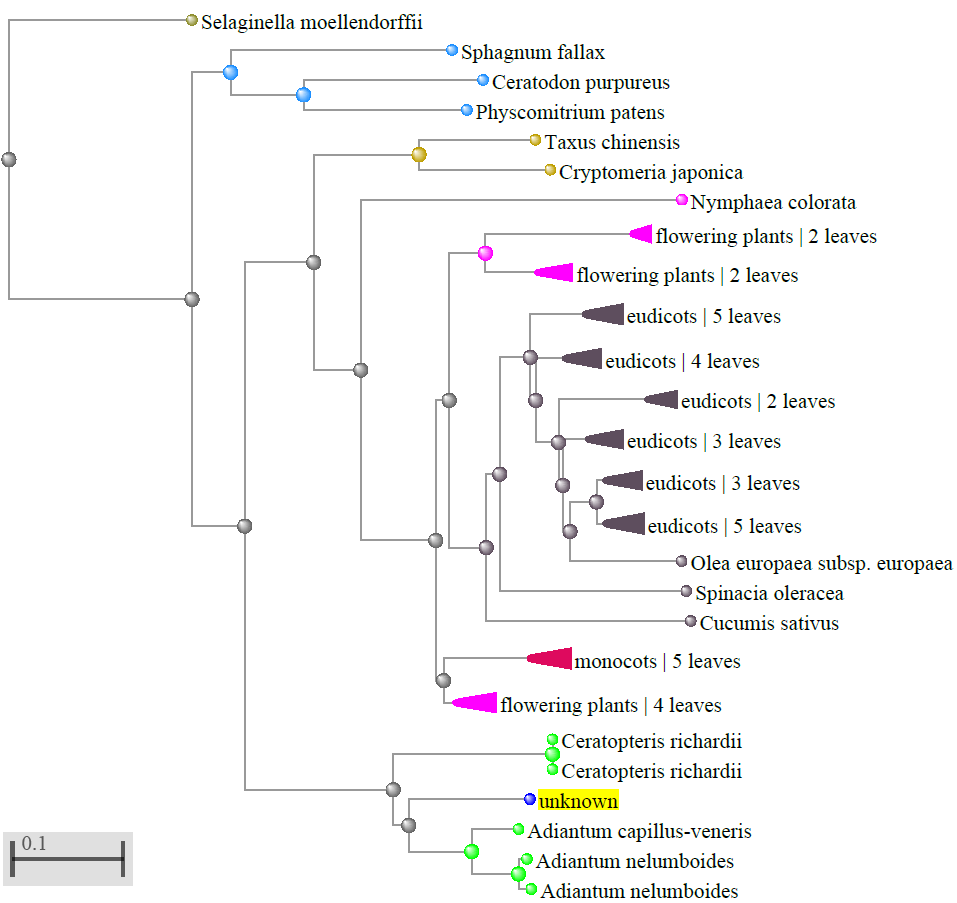


**Supplementary Figure 1**. Phylogenetic trees corresponding to proteins associated with vegetative and reproductive development, as well as with response to stress from the apogamous gametophyte of the fern *D. affinis*. The yellow line indicates our sequence.
