# Supplementary material for: Transcriptomic analyses in the gametophytes of the apomictic fern Dryopteris affinis
Source: Planta. 2024 Oct 2;260(5):111. doi: 10.1007/s00425-024-04540-w (PMC11447071; doi:10.1007/s00425-024-04540-w)
Supplement: Supplementary file 3 — Supplementary file3 (DOCX 410 KB) [file 425_2024_4540_MOESM3_ESM.docx]

**Article title**: Transcriptomic analyses in the gametophytes of the apomictical fern *Dryopteris affinis*.

**Journal name**: Planta.

**Authors names**: Sara Ojosnegros^1^, José Manuel Alvarez^1^, Valeria Gagliardini^2^, Luis G. Quintanilla^3^, Ueli Grossniklaus^2^, and Helena Fernández^1^

**Affiliations**:

^1^Area of Plant Physiology, Department of Organisms and Systems Biology, University of Oviedo, 33071 Oviedo, Spain; uo286037@uniovi.es (S.O.); alvarezmanuel@uniovi.es (J.M.A.); [fernandezelena@uniovi.es](mailto:fernandezelena@uniovi.es) (H.F.)

^2^Department of Plant and Microbial Biology & Zurich-Basel Plant Science Center, University of Zurich, 8008 Zurich, Switzerland; vgagliar@botinst.uzh.ch (V.G.); grossnik@botinst.uzh.ch (U.G.)

^3^Global Change Research Institute, University Rey Juan Carlos, 28933 Móstoles, Spain; luis.quintanilla@urjc.es

**E-mail address of the corresponding author**: [fernandezelena@uniovi.es](mailto:fernandezelena@uniovi.es)

CULLIN 4 (CUL4): TIMEKEEPER LOCUS1 (STIPL1):


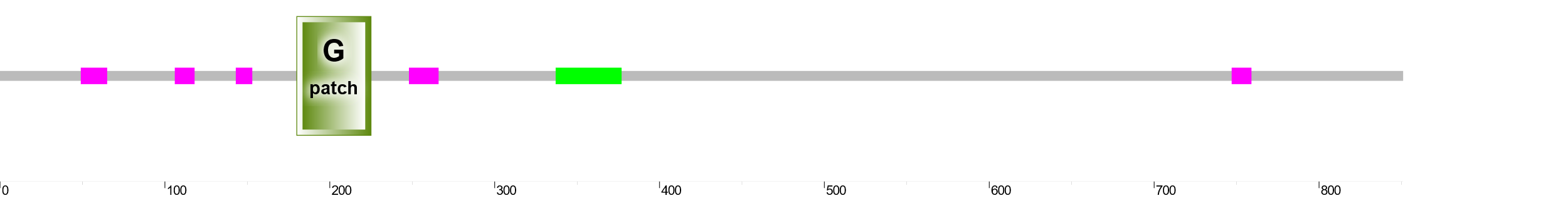

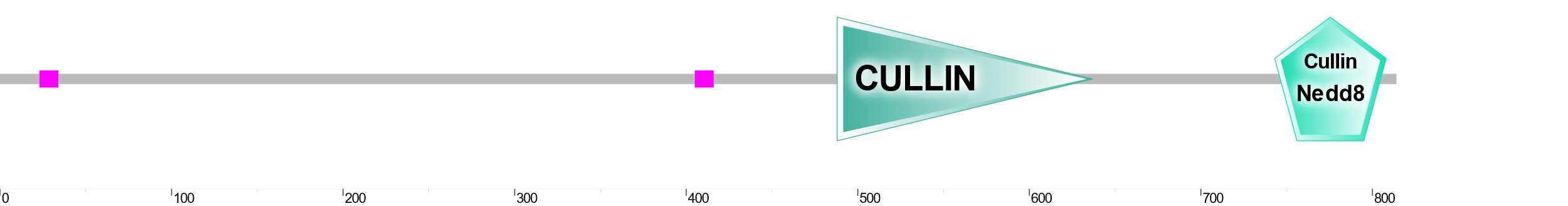


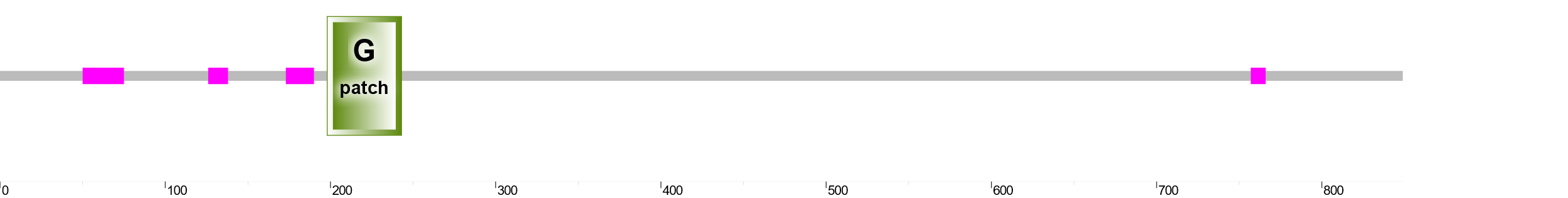


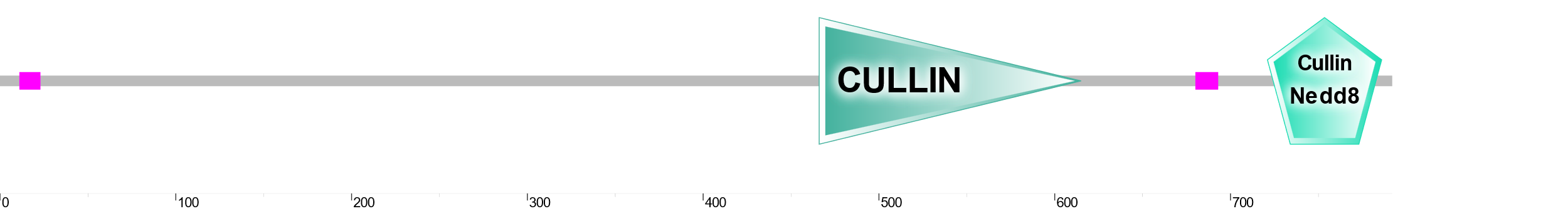


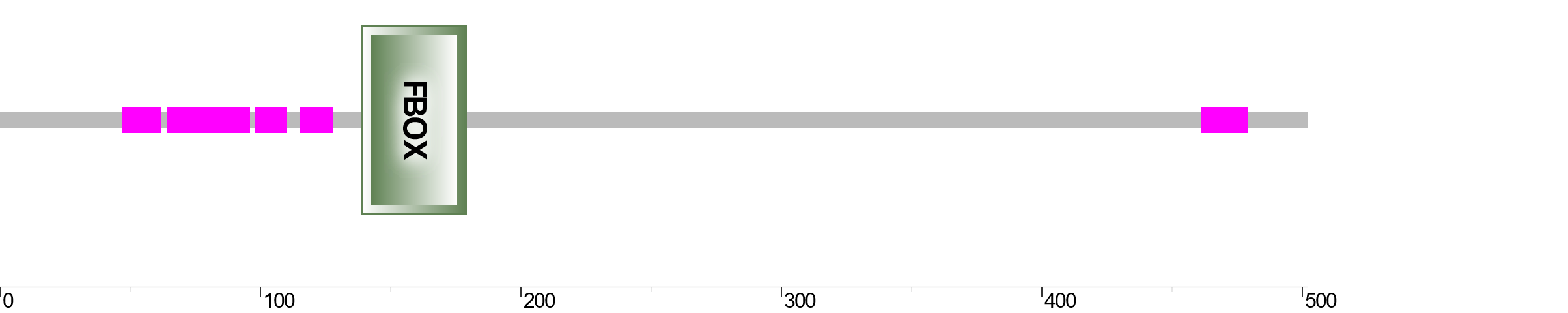

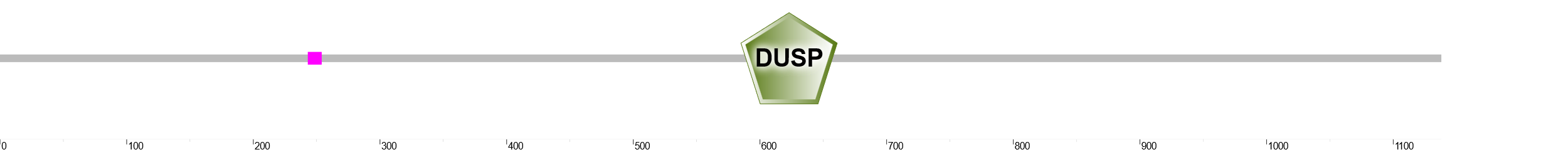
UBIQUITIN-SPECIFIC PROTEASE 26 (UBP26): UNUSUAL FLORAL ORGANS (UFO):


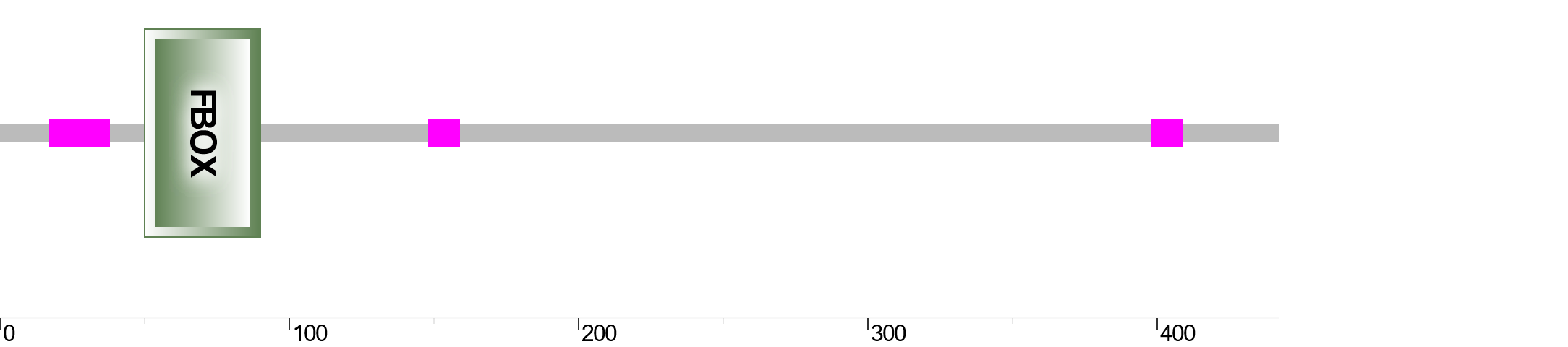

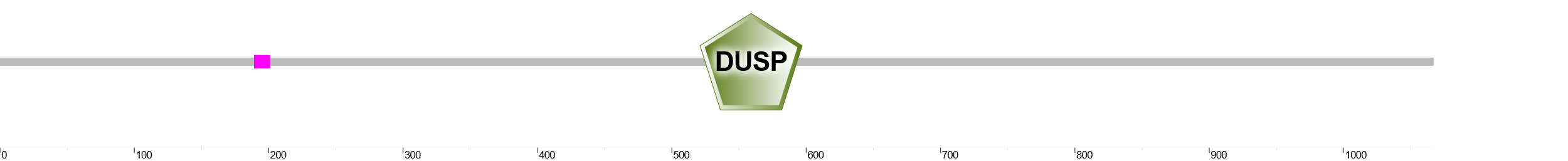


**Supplementary Figure 3**: Schematic representation of domains of proteins from gametophytes of the fern *Dryopteris affinis* above and from *Arabidopsis thaliana* below obtained with SMART version 9.0 software.
