# Supplementary material for: Transcriptomic analyses in the gametophytes of the apomictic fern Dryopteris affinis
Source: Planta. 2024 Oct 2;260(5):111. doi: 10.1007/s00425-024-04540-w (PMC11447071; doi:10.1007/s00425-024-04540-w)
Supplement: Supplementary file 4 — Supplementary file4 (DOCX 18 KB) [file 425_2024_4540_MOESM4_ESM.docx]

**Article title**: Transcriptomic analyses in the gametophytes of the apomictical fern *Dryopteris affinis*.

**Journal name**: Planta.

**Authors names**: Sara Ojosnegros^1^, José Manuel Alvarez^1^, Valeria Gagliardini^2^, Luis G. Quintanilla^3^, Ueli Grossniklaus^2^, and Helena Fernández^1^

**Affiliations**:

^1^Area of Plant Physiology, Department of Organisms and Systems Biology, University of Oviedo, 33071 Oviedo, Spain; uo286037@uniovi.es (S.O.); alvarezmanuel@uniovi.es (J.M.A.); [fernandezelena@uniovi.es](mailto:fernandezelena@uniovi.es) (H.F.)

^2^Department of Plant and Microbial Biology & Zurich-Basel Plant Science Center, University of Zurich, 8008 Zurich, Switzerland; vgagliar@botinst.uzh.ch (V.G.); grossnik@botinst.uzh.ch (U.G.)

^3^Global Change Research Institute, University Rey Juan Carlos, 28933 Móstoles, Spain; luis.quintanilla@urjc.es

**E-mail address of the corresponding author**: [fernandezelena@uniovi.es](mailto:fernandezelena@uniovi.es)

**ARGONAUTE 1 (AGO1**):

- DUF1785 domain:

IAVSRSFFKSELGSAQLEGGLVALNGFYQSLRPTESGLQLNIDLSTTAFHAS

- Paz domain:

DFLRQQLRNFDPRYRLTDVVRVKVKRALARLKVQVIHRQTPRRYRISGLSTSPTKDLKFPIEGGEEMRVVDYFKLTYNYVIEFPELPCLQVQANKPSYLPMEVCVICDGQKYGGKLNDRQTTRLRGLACVLPKVREAKI

-Piwi domain:

LLVCVMADKHPAYGELKRICETQIGIVTQCCLSRHVKQCKSQYLANLALKVNAKAGGRNVTLALELPKMCPVFNRPTIIFGADVTHPSPGDDTGPSIAAVVANIDWPSANRYIARVRAQTHREEIIEYLREMVQELWHEFCEKTKSRPDRVIMFRDGVSEGQFDEVLQREVAALKDAFIEVGGPDYKPLITWAVVQKRHHTRLFPADDKCKDKNNNILPGTVVDSTITHPREFDFFLCSHAGIQGSRPTHYHVLWDENNFKSDDLQGLVYNLCYTYARCTRSVSVVPPAYYAHLAAYRARLYLD

**BABY BOOM (BBM):**

- AP2 domains:

**1)** IYRGVTKHRWTGRFEAHLWDNSCRREGQTRKGRQVYLGGYDKEEKAARAYDLAALKYWGPTTTINFQLDDYEK

**2)** IYRGVTRHHQHGRWQARIGRVAGNKDLYLGTFGTQEEAAEAYDIAAIKFRGINAVTNFDMSRYDI

**WUS-INTERACTING PROTEIN 1 (TPL):**

-LisH domain: LSRELVFLILQFLDEEKFKETVHKLEQESGFFF

-CTLH domain:

FFFNMKYFEDQVQAGEWEEVERYLSGFTKVDDNRYSMKIFFEIRKQKYLEALDKQDRAK

- WD40 domains:

**1)** PKMVARILNQGSCVMSMDFHPIQQTILLVGTNVGDIAIWD

**2)** MPMQAALVKDPAVSVNRCVWSPDGTLLGVAFSKHMVHIYS

**3)** DLRQHLEIDAHIGGVNDLAFSHPNKQLCIITCGDDKTIKVWD

**4)** NGRKQYTFEGHEAPVYSVCPHHKESIQFIFSTAIDGKIKAWL

**5)** LLGSRVDYDAPGQWCTTMAYSADGTRLFSCGTSKEGESYLVEWN

**6)** EGAIKRTYSGFRKRSLGVVQFDTTRNHFLAAGDEFQIKFWD

**DICER-LIKE 4 (DCL4):**

- RIBOc domains:

**1)** KKILEALTTEKCLDSFSLERLELLGDSFLKYAISRRLFLEHEEVDEGFLSLQRNQRICNSSLFTLGSKLGLAGYIRDTLFDPKHWVAPCHPSKAVCGESLLGDLHGDSSESKEWKASVTCNKRHRWMQRKTVADVIEALIGAYLEDGGEQAAVSFMQFIGLEVATDISQ

**2)** GLLIEAFTHASFTNHLGKCYQRLEFLGDSVLDFLITKHLYREFKDSKPGELTDLRSTIVSNESFSRIAIQHKLYAYLIENSTELRKGINEFISYICASSEQELIGDREGDKCSKVLADILESLSGAICVDGEFNLSLVWKVFEPILGRLISSRLV-

- DSRM domain:

PVRELQEVCQKNHLTWTKSSSSLGGRQCQYTYEINVSNNIVKGTSVSKDKKSAKKRAAINALRE

LK

**CULLIN 4 (CUL4):**

- CULLIN domain:

DRVLILFRFIQGKDVFEAFYKKDLAKRLLLGKSASIDAEKSMISKLKTECGSQFTNKLEGMFKDIELSKEINESFKQSSQARTKLPSGIEMSVHVLTTGYWPTYPPMEVRLPHELNVYQDIFKDFYLSKHSGRRLMWQNSLGHCVLKADY

- Cullin Nedd8 domain:

VFQDRQYQIDAAIVRIMKTRKILSHTLLITELFQQLKFPIKPADLKKRIESLIDREYLERDKSNPQ

**TIMEKEEPER LOCUS1 (STIPL1):**

- G patch domain: KHTKGIGMRLLEKMGYKGGGLGKNEQGIAVPIEAKLRPKNMGMGFN

**UBIQUITIN-SPECIFIC PROTEASE 26 (UBP26):**

- DUSP domain:

ALNQDNGYFWISSSWLRSWADELKPFPIDNSELLCEHGKVPPSNVCAMKRISEGAWANLQSQYGGGPELSAYDCCVE

**UNUSUAL FLORAL ORGANS (UFO):**

- FBOX domain: LPEKLVERVVASLPLPSFFRSRLVCKRWYSLLFSDSFLELC

**Supplementary Figure 4**: Amino acid sequences of the domains of selected proteins of *Dryopteris affinis* obtained from SMART program.
