# Supplementary material for: Transcriptomic analyses in the gametophytes of the apomictic fern Dryopteris affinis
Source: Planta. 2024 Oct 2;260(5):111. doi: 10.1007/s00425-024-04540-w (PMC11447071; doi:10.1007/s00425-024-04540-w)
Supplement: Supplementary file 5 — Supplementary file5 (DOCX 127 KB) [file 425_2024_4540_MOESM5_ESM.docx]

**Article title**: Transcriptomic analyses in the gametophytes of the apomictical fern *Dryopteris affinis*.

**Journal name**: Planta.

**Authors names**: Sara Ojosnegros^1^, José Manuel Alvarez^1^, Valeria Gagliardini^2^, Luis G. Quintanilla^3^, Ueli Grossniklaus^2^, and Helena Fernández^1^

**Affiliations**:

^1^Area of Plant Physiology, Department of Organisms and Systems Biology, University of Oviedo, 33071 Oviedo, Spain; uo286037@uniovi.es (S.O.); alvarezmanuel@uniovi.es (J.M.A.); [fernandezelena@uniovi.es](mailto:fernandezelena@uniovi.es) (H.F.)

^2^Department of Plant and Microbial Biology & Zurich-Basel Plant Science Center, University of Zurich, 8008 Zurich, Switzerland; vgagliar@botinst.uzh.ch (V.G.); grossnik@botinst.uzh.ch (U.G.)

^3^Global Change Research Institute, University Rey Juan Carlos, 28933 Móstoles, Spain; luis.quintanilla@urjc.es

**E-mail address of the corresponding author**: [fernandezelena@uniovi.es](mailto:fernandezelena@uniovi.es)

**Supplementary Table 1**. Nucleotide sequences of proteins found in gametophytes of the apogamous fern *Dryopteris affinis*.

| **ARGININE-RICH CYCLIN 1 (RCY1):**  TCTCTCTCTCTCTCTCTCTTCCTTCCAGCCCTAGCTAATGATTTACACCGCCATCGACACCTTCTACTTGTCGCAGGAGCAGCTGCAGAATTCTCCCTCCCGCAAAGATGGCGTTGATGAGGAAACAGAAACTGTGCTTCGGCTCTATGGTTGCGAGCTCGTACAGGAGGGCGGCATACTTCTCAAGCTACCTCAAGCAGTCATGGCGACAGGCCAGGTGCTGCTTCATCGATTTTTCTGCAAGAAGTCATTTGCCAGGTTCAATGTGAAGAGAGTAGCTGCAAGCTGTCTTTGGGTTGCAGCAAAACTTGAGGAGAGCCCAAAGAAGGCCCAGCACGTCTTAAACGTGTTTAATAGAATGGACTGCCGTCGAGAAAACAAGCCTCTTGAACTGCTAGACCCTTTTTCTAAGAGGTACGAAGAATTGAAGATTGATTTGATCAGGACAGAACGCCATCTTTTAAAAGAGATGGGTTTCATTTGTCATGTTGAGCATCCACACAAGTTTATTATCAATTACTTACTACAGCTTGAAACTCCACCAGAGTTGATGCAAGAAGCATGGAACTTAGCTAATGACAGCTTACGTACTACGCTTTCAGTTCGATTCAAAAGTGAAGTGGTGGCTTGTGGCGTTGTCTATGCTGCTGCGCGGAGGTTCAATGTCAGTTTGCCTGAAAGTCCTCCTTGGTGGAAGGTATTTGATGCGGAGAAGGGTGATATTGAGGAAGTATGCAATGTCCTTGCAAACCTCTACAAGAGGCCCAAAGCGAGCTATATTGAAGTGTCAAAAGATTCCAAGTCTTTTGTGTTAAGCAGTAGAGCTTGGGAACCTTCTGCAAGTGTGAAGGATTCTCTCGGAGGTTCTGTAATTGCAAATGGAAGCAAACAGGGCCAGATGACCACAGAAACACGAGAGTTTATGGTTAAAGTAGCCCTAGATAAACTGAAGAACACTTCTGCACAGGATTCAAGGAGACCTTCTGACGAGGCCAGGGCTTCTTCTGCCAATGGGGAGCTTTCAGACGAGTTAGTCAAGGGAGCTGAGGAATTGGACGAGTTTACGCCTGATAGAAACAAGGAGTCTGCAGACACCGAACTCAGGATTAAAGAGAAGGACAAAGACAGGGCAAAGGCTCGCGAAAAGGGAAGAGAAAAAGATAGGCAGCGAGAGTATGAGAAGATTAGGGACAAAGATCGAGGAAAGATTCGAGAAAGTGAGTATGAAAGAGATCGAGATCGAAAAGAGAGGGAGCATCGCAAGGAAAGGACAAAAGAATCAGGTTATGCTGACAAGAGTAGACATCATGGATCAAGCCGAGAAAGGGAGTATCATAGTTCATACAAGCTTTTCCGTGAAAAGGACCGACATCGGCATCATCCTTACTCATGAGAAAGTCTACGAAGATTTAGATTTTACTTTCTTGTAGCTTAAAAGTTATCATCCAGTATGGAAGGCTTTTATTTGATGGTGCTGATCAGTGATTGTTTGGTCAGTAGCTTAAACAACTGTTTCTTACAGTTTCCCTTAGTGGAGACTTCCTTGTAGAGCTGACTTTTGCTCGGTGAGCCGCACTGGATGATAAAAGAATGGAGAGTTGGGAAGGTTGCTGTTCAAGCTGATCTGAGGAGCTGCTTCATATACTTCACGCCCCTCGTAACAACACAGCACACTGTGCTCATGAGGGGACAATGTGGCAGGCCATTCTAGTTCTGATCTGGTGGTAAGTGCGGATGGACCACCTTGTGCTGCAATCGAAGAATGCAGCAGGATGTAAGATCACATCAGACGGGTTTCCAGTAAGTGGAACTGCATGTTTTTATCAAATGCTTTATGGGTTCTGGAGCATGCCTCTATATAAAATAGTAAAGCAAAATGGGTTATTGTATACCTTTTATGATTGTGGGTGTTTTCGCTATGTTTTTCTACTTTGGATGGGGAATACTTGAAGGCTTTATTATTGTTGGAAGTAGCAGGAGCTGCTGGAATTTCATTAATACTGGATGCTCATAGTTGTCCTTGCAACTATTTCAAGCATACCTGCCATGCGAGAGCATGGAGTCTTTCATTTAAAGATCCAAAGCTAGAGCAATGACCACTTCACATAGATCGCAAGGATAGAATTTATAACTTTTGATGCATGAAAGTGGAAACAATATAACTCTGTATTTACCGCTCCATCATCATCTGATTGTATGGTAGGTCAGCAAGCACCTAGCCATTAATGTTATCTAGCAATTTAGCCAATGCAGGCATTCTTGATTTTGGCGACCTTCAGTACACAACTTTTTGTTTGCATGCACAGTCGAGGGAGTTCTTGAGGGTCAAATTATCATCAGACGAATGACTCTCTAGATGGTCTATATTTGGAGTCGCAGAGACGCACATAATTGACTTGGATCTCCTGTTGGGGTTCTGGCATTTTGTTGAGTATAAATTGGTGTACAACCTTAAATATTAGATATTTCTGTCATGGTTTCTTGTTTAAGGGGTACTATTGCTTGTGGTGTTTAAGCGAGTTATCATCAGTGGCTTTGTAGCTTAAATTGGTGCTAGGGGAGGTTGTACAATTTGACAAGAAAGAAAGAAGCCCACAGAAACCTGCCTCCTTGGTTTGCATATTAAGAGTTGAGCAGGGTTGCTCACTATTTAAGTTGAAGAGGTTATCCCTTTTTTTCAAACAACTAAATACCTGGCTAAGAAAAGTTTTCGAGTGTCTTATTGCTTAATGTAACTTCTTATACATATAATGACTCGTAATGTTATCTGTACAATTGTGGGCCATAAGAAAAGCAGCACTTGTGAAGCAAAACGTTTATTGGAGTTGAAAAGTGCCTCCTTGTCAATCAGATCATCTATATTGAATTGATTTATGATTTATCGATTGCTTTTTGGTTTAATGAGATCTCAAATGGGGGATTTGTTGTGCTACCACCAAGGGAGTACCTAGTGATCGTAATGTGTGTAGATTACAAATTAGTAGGTCAAATAATCATTTTTCAGAGAGGAACTGGTCCAGCAGCTCAAAGCATTTCATACAAAGACTTCAGAGTCAGGATTTGGTTATGTGAAATCGGTGAGTGAAATGACTTCATAGTCCTTCCTGTATATACCAACAAAGCTCAGTGCGACATGGCTTGTTTTGAAAAGGCTGCTTGAGTGCCTAATTTTTTTTCTGTTTTAAGGGTTTTGCTTGTAATTATTCCATCCTTGAGGACAGTAGAGGTATTGCTTCACATTACCGGTAAATATTAGCTCTGCCCCTTTTCAGAAGGTTTTTGTTGCACCAAAAAATTTGTGAGATTTCAATGCATAATAAGAATCTCATACCGTTTTTGTAATTATTGTTTTATTTTTCTAATGGAGAGGTTTCACCTTTGAGAATGTTGTATCAAGAGGATTCATGTAATTCAATGTTGTGAGGTGTGAAACAAGTG |
| --- |
| **KEULE (KEU):**  GCGAATTAGTGATCTCACTATTCATCTTGGCCGTGGCAGGCTCCTTGGTTTCATGGTCAATCCGTGAAACCTCCACTCGTCTTCCGCCATTTTCTAAGAAGAGGAGAAGAAGACGTGCCGCCTTGCCCTCCTCTCTCGGAAGCTTCCTCTTCGTCTTCTTCCTCTGCTCGAGCCCTCCTGCATCGCCCCTTTCGGAACTTCATTCTTCAATCCCTAATCCTGCAGAGCTTTGCAATACACAGATATTTCTTAGTTTTCACGCAAAAGGAGATCAAGTTGTCTTCTGTAAGGTTTCCGCAACCCTAATCAACCATAGGAAGGGTCTGGTTCTTCAAACTCTTGATTATCAGGTGCTCCAATCAAACTTTCTACTCTACATTTCATCAAGCAGAGGTTGGATGCAAAACATACTTGTAACTCTGCAGAGCATCCAGAGATCATAAAAACCTAACATTATATTGCTTTTGCACTACGACACGCGGCTGCTTTATCAAAGTGAAGAAGTTGCTACACTTGTTATCTACGAGGACGCCATAGCCATAGCCCTGCACGACATCTTCAACCTTTTGCCTTCACCATGTCAGGATCGGAATCTTCTTTGCATTCTTGGAATTCTGGCAGCTATAAATCATTCCGCCAAGTTACTCGAGAACGTCTTCTTCATGAAATGCTCAGGCTGGCTAAAGGAAAGGATTCTTCGGATTGGAAGGTACTTATCATGGACGAGGTTACAGTGAAAGTGATGTCATATTCTTGCAAGATGGCAGACATAACAGATGAAGGCATTTCATTGGTGGAGGACTTAAATAAGCGGAGACAACCACTTCCAGCATTGGAAGCAGTATACTTCATTCAACCTAAAAGAGAAAGTGTTCAGAAGCTCAAGATGGACTTGTCTGGAAAAACACCTCTTTATAAAAAGGCCCATGTGTTTTTCAGTTCGCCCATAGCTAGAGATCTGGTACAGGCCATCAAAAGTGATGCGCTGGTTTTTTCCCGGCTTGCTACTCTTAGAGAGATGAATTTGGAATATCTCACGATTGATTCTCAGGCATTCTCAACTGATAATAGTAGAGCTTTGGAGCAGTTATTTGGAGAGCATAGTGAAGGTAGTCGTGACTATGATGCTTGTCTGAGCACAATTGCCGCTCGACTCAGCACTGTATTTGCATCTCTCAAGGAATTTCCAATTATCAGGTATCGGGCTGCAAGGTCGGCAGCAGTAGATTCCACTGCTGCAACAACTTTTCGAGATCTTGTACCGACCAAGCTGGCAGCAGCTGTCTGGGACCGTCTTCAAGGATATAGAAAGTTGCCAGGTTTTCCACAAGAAGAAAGTTGTGAACTTTTGATTGTTGATCGATCAATAGACCCGATTGCTCCTATTATACATGAGTGGACCTATGATGCAATGTGCCATGATTTGCTGAATATGGAAGGCAATAAGTATGTGTATGAGATCACAACCAGTGCTGGCAGGCCAGACAGAAAAGAGGTTCTTCTTGAAGAGCATGATCCTGTTTGGGTTGAGTTACGCCATCTACATATTGCTGCAGTTAATAAAAAGCTTGATGAGAAGATGACAAATCTGAAGGAGAAGAACAAGGCTGCTCAAATACGACTTGGCTCCAGGGAAGGGCAGGAACTCACCACACGTGACATGCAAAAGATGGTACAGGCGCTACCTCAGTTTCGCGATCAGATTGAAAAGCTGAGTCTGCACATTGATATAGCAACAAAGTTGAATGCGAAGATTGGAGAGCTTGCCTTACGAGACATCGGCACTTTGGAGCAAGAGTTGGTGTTTGGAGATGCTAGCAGCAAGGAGCTTATAAACATACTAACATCGAATCAGAATCTCACCAAGGAAAACAAGTTGCGCTTGCTTATGATATATGCTGCTTCACACCCAGAAAAGCTTGATGCAGCAAAAAGGCTTCAATGGATGAAGCTCGCAAGGCTTACTGAGGAGGATATGAATCCTGTGGTCAACATGGAGTATATGGGTGTGTCTGTTTCAAAGAAGCAGTCTGGTGGTTTCTCCCTTAAATTTGGTACTCGCAAAAACAAGCGAGGTTTGAGGAAGGAGAAGAACCAAGATGAAGAGATTTATGATTTGTCTCGATTTTGTCCAATGATCCAGGAAATAATAGAGGATTTATCGAAGGGTGAGCTATCCAAAGAAGAATACCCTTATGTGACGGAACCACCAGCTGCAGTTTTGCACTCTATGTCAGGGGACAGTCTCTCCGTAAGGTCAACCACAACAAATTCTAAACCAGCACAGTCCATGAGAACCTCAAAGCCTGGTTCTACTTGGGCCACCAGGTCCAAGCCTTCTGATGATGGACAGTCTAGTGATTCGGCGTTAAGGCATGCATACAGTGATCCCAAGATAACTGGAAAGCGCATTTTTATTTTTGTGGTTGGTGGCGCTACTCGTTCTGAGTTGCGCGCAGTGCACAGACTTACTGGGCAGCTAAGGAGAGAGGTTGTACTGGGAACTACCAGTCTGGATGAACCTCATGAATTTCTAACGAAATTGAAAGTGCTTAGCGTTCTGGACGAGGTTTCGCTCGACGATCTTGAGATATAATATACTATCAACAGTTCTATCTGATACTGGGTTCAGGAAAGCTGCGGCAAGGTGTCTGCATCAGATGGTGTACAAAAGTTTTCTAAATCACAGGAATGCTCACTTTTGCGGTCCTCATCATTTACCAGTTATGCAATGGGCTCACACCAAGGGGAACTGTTACCTAAATGCACTGAACATTCTTAAAAAATCAGCTCGTTGTGGTTGCATGAGCAATTCCCTGGAGACAATTCATAAGTTAGAAGCGTTCGTAACCTAGTTAGCGAGCATATTGGGGCTGTATTTTGTCTTCTGAACACCTAGAATGGTTTGGGAAGTAAAATTGTATTTATTCTTGAGAGAGATGTGTATTGCCATTTCTGTATTACGTGGCTATTTTGAACTGGAGTGATGGTAAGGGCACAATTATTTTGTAAAGCTTAGGGATCATGAGTGCCCCATAGTAATCCTCTCTTGTATTTTTAAATAAATCAAGTTACGTTTCTAACAAGGAATGGGATGGTGATTTTTCTGTTGTGCACCAAAGGCCAACTTGGTGTGTAGGAGATTAGAGATGGTGCACAATTTTGTCA |
| **DYNAMIN-LIKE 1B (ADL1B):**  CCCTTGTCTTGCAACTGCATAAAATTGGTGAGGGATCAGAGGATGCAGAGTTTATGCATCTGCCCAAGCGGAGATTTACATATTTTGGATAAGAAATTGTGGAATTCCTATTTTCATTCCCTTAATTGAGGTGGTTGCAGTTTGTAGCTCAAGTACTGGGAGATTAATAGAAGTTGCAGAGTATCGCTCAGGTGAAGGCGGGGGGTTAAATCAATAGCTTTAGCTGGTACAGGTGGTGGTTCAACAGAAACAGGAGGTCATCAGGTTCAATAAGTTTTGGTGGTTCTGGAAGCTGAGGATGTTGTAAATGCATTATTAGAATGGCTAGAGCTTCGAATTGGGCGTGGAGGCAAGGAATATTTCACGCGCTTTAAACGCCACGAGAGACCCACTTTTTTTATCCACCACTGTAACTTCAACCGAAAGCTCTTTAAGTGGTAACGAAGAGCGATCATACATACAAAGTGTTGACAGATTGCCAAGAAGCTTGAAGCACTCTGGGAGCTTGAGGCAGTCTGGCAGCTTCAGGCAGTCTAGTAGAGATGGTTTTGTTACAAGGAGGAGGAGCGATGCAGATATGTGGCAATGACACATTGATGATGGAGCTTTAGAGCATTTTACAGCCCGAAGGAGTGATG |
| **STOMATAL CYTOKINESIS-DEFECTIVE 1 (SCD1):**  CTGGTTCTCACTTGAGAGAGGAGAGAGAGAGTTACAGAAGTCGATACGGTGGCTGGATCAAGGCAAACAATACAGATGAACACGTCCATAGCTTGTGAGCAGGATCAAGGGATACATCCGATCTTACATTGATCTGTGGGTCGTCTTGCAAATCACAGCAATGGGTTGGGCAGCTCGGGCCATTGGGTACTTCGTAGTCTGTGGCTTGGGCCCACAAGTGAAGACCATGGAGGGCAAGTCTGGCTTCCAAGGATTCGAAGATACTTACATGCCATCTCTTTTGGATCAGTTTCCTCCGACACCTTCCTCCGGCGTATCCGAATTGCCTCCTCAGCTACCTCAGTGTGCATTGCCTACAGGGGTGGAGTTCTACTCATCTGGTCTGACCAGTGGAGATGAAACCAGCCAGCCTCACAGCTATCCCATCATTTTGACAGACGGGGATGGCGCAAAAATTTATGTAAGCTGTGTTGCTTTTCGAGATCCAGTTGATGACGATGTCGCTCAAGCTTATCGAATTCCACCAAATTCTTATGTAGCTAAATGCATCTGTATAGTTTCACATTCACCTTATTTTCATGTCTGCCGAGAGGCAGTAGAAGAGCTACATCGTCTCTGCTTCTCAAGTTCAGGCTGCAGCAAACCTTTGTGGGATATAATTTCGCATATAGTGTTAAATGTACCCCTGCCAAGTCCAGGTGGGGGCATTGTTCTTTTTGCAATGGAAAATCATTTGTTATCGATAGAAGGTCCACCAAAGGATGGTCTTCCACATGCAGACCTCTCATTTCAACCGTTGATCCAGTGCTTGGATGTAGATAACGTAATTCGTTTGTTCACGGCAGTTTTTCTCGAGAGGCACGTTTTGCTACGGGCTTGCAAGTACTCTCTTTTGACAGTTGTGGCTGAAGCTATTTGTCATTTGCTGTATCCAATCAAGTGGCAGCATGTTTTCATTCCAGTCCTCTTCTGTGGAGGAGTGGATTACCTTGACGCTCCAACTCCTTATCTGATGGGCCTTCATTCTAGCATTGACATATCCTACCTTAAGCTTGATACGGTGGCTGTGGTTGATCTTGATCGCAACACTATCACACTTTCAGAAGAGATGCCTTCTATCCCAGAGCCGGAGTTGGGACAACTTAGGGCAGATCTGGTGGAATTTTTATATCCCAATCTTGCTGATATGGATCGAGTGCAAGACGGTGCTGGGGTTCTTAGAGATTCTCGCATTCTAAGAGGAAACAAATCTTGGAGTGCAAGTCATGACTCTGAATTAAGGTTAATTTTTTTGAAATTCTATGCTACAATTCTATCTGGTTACCGTGATTTTGTGGTGTATTGCAACGCTAGCAGCCAGGAGACCACAGAGGAAAACTCTTTCAACAAAGAGGCTTTTTTGAGGAAGCGCTCTAGAATTACCCGCCTTCCTCCGGACCCTACGATGGATCAGTTTTTAAGCTCCATCGGTTTTCTTGAATTTATACAAAAAGGCTACGGATCTTCAAGTACAGGGCAGAATCTTATGGATAAGTTGCAGCAGGCAATGGCTAGAGGTCAGAATCCTTCTGTTGTTCTTCCTGCTCCAGCCGCCGAGCCCAACATACTGACCATTTCAGATCATTCAAGTCAAGAAATAGCATCAAGAGCGAAGTATCGATATGAAAGATTTCCAGCAAATGTCAGAACTCATGAGCAAGAGGAGGAAAGAAAGAGAATTCTAGAGGCTGCAAGTACAGCTTTGGACCATCCAAAGCGACACAGCTCAAGCCCGGGGCTTTTGCAAACAGGGAAAATGGATGGCTTATCTCCACTTGAGAGGGCTGCTGAAAGAGAACGAATGGTTCTTGACATCAAGGTCAAACTACAAGGGCTGTGGCAACGCCTCCTTGTTCTTGGCTCAGCAGAAGACCCGCTTGCTTCTTTTGAATATGGCACAATTCTAGCACTGATCGAGTCAGATGCAGAAGGAATAGGTGGAAGCGGGTTCATTGAGTGCATCGGTGAACACATCAATTCGGGTTGGAGTTGTGGTTTGACTGAAGAGCAGTTTATTGCTGTGAAAGAACTTATTAAAACCACCATTAGCCGTGCGATATCTCGGAATGACATGGTGACTGTGCGTGCAGCACTTGAGTTTTCTGCTGTAGTTTACAGAAAAGATGTGGGCTCTGTCTCTGATTATGTCCAGCGCCACCTTGGTGTTCTTCCTATTTGGGATGAATTCAGGTTTTGGGATGGCTACTTTGATAAACTTTTGGATAGTTTTGCAGACAAGTCTGGAAATTTTGCTACTTTGGTTTCAGAGCAACTTATCATCGTTGCACAGCACATGGCGGGCTTGGGTCTGCCAGATTCTGAAGCATGGTTTATTCTAGAGGCTATTGCAAGGAAGCATAATTTGGGACCAAAGCAGATGATCAAGCTTAGGGGCTTGCTTGCTTACATGCAACAAGTTAGGGTTGGTTACTGGGGTTTTACTCCTATACGATCCCAAACTGCCTATCCTGGTGGAACAAACCAAGCTCAATCTCATGCGCTAACAGATGAAGCACAACAACCAGTTGAAGCTGCAGGCGCGACACGCGGCTGGGTACATTCCATGTTTAGCAGAGATAAAGCTGCATTTGCTGCTGGTAGACGTCGTGGTACATCAGATAGCAGTGTGTTAGCTGCCAATGACAATATTCAGAACATACAACAACCGAAGAATGTGGACGGTTCAAATACTGCCCAAAGAAGAGGCTCTTCTAGTGTTAGGACACTCAGAGGGCATAAGGCTGCAGTAACAGCATTGCATGCTGCAACAAAAGCAGAATGTGGGGACAATGGTGTGGACAGTGATGAAGTCGGCCATTTTATCAGTGGTTCTGCTGATTGCACGGTTAAAGTTTGGAATCCATCTCAGCGAGGATCAGAGCTGCGAGCAACTTTGAAAGGACACACTATGGCTGTTCGTGCGATTGCGTCAGACAGAGTTCGTATTGTTACTGGCTCCGATGACCAACGTGTGCTTGTTTGGGACAAGGCTACAGGAAAACAGCTTGCAGAGTTAAAAGCTCACAATGACAAGATTAGTTGTGTGCGAATGCTATGGGAGCCTTGTGTTTTAACAGCCTCACATGATGGTACGGCCAAGATGTGGGATGTACGAGCTGATACATGTGTCGCAACAGTCGGGCGAAGTGCAAGTGCTATTCTGTGCGCGGACTATGATTATGAAACAAGGGTCTTAGCAGCAGCTGGAATGGATGGGGTCTGCAATATCTGGGATACACAAGCTGCAAAGCAAAGACACAAGTTTCTTGGACACAGCAAATGGATCAGGTGTCTAAGAATGTCAGGAGACACCGTTGTCACAGGAAGTGATGATTGGACTGTCAGAGTATGGTCTGTTTCCAATGGGGCTTGTGATTCAGTTTTGACATGTCATTCTGGGGCAATTACATCAGTTGAGTACTGTGCAGCGGATAAAGGCTTAGTTACAGGCTCGGCTGATGGCATGGTGCGGATGTGGGAGAGGGATGAAGGGAAATTGAGATGCACTAAGAATGTCGGAATCCATTCAGCATCAATTCTTTCTCTGCGGGCGGCTGACAAATGGCTGGCAATTGGGGCGGCAGATAACTCGATGTCCCTGTTTCAGAAGGCAGAACAAAGAGGGGGGACAGCTGGTTGGCAACTATTCAGAACACCGCAGCGAATGGCAGCTGTGGTAAGATGTGTAGCATCGGATCCGGAGAAAGGAAGGATATGTAGTGGAGCTAGAAACGGTTTAATCCGACTCTGGGATGCTGCAATCACTGTTTAGTCTTGAGTGCAGACCTTGAGGTTGGATTCTTTTGTTAGGTCCAGATCGGTTGCACCTGCAAGTGAGAGAAAGAAGCTTAGTACAGATCATTGAATGCAAGAGCATCCACATCCAATTGGAGCTGCTGTGGCCTTGCTAGGGATTCAATCTGTCTTATGTACATTAACTCCTTTGTGAAGCATTTTTGCTACCAAGGCTTTAAAGCAGCTCATGTTTTTATAGTTATTTTCAAACCTCTCTCTCTCTCTCTCTTTTTCTTATCTCTCTTTCTCTCTTCTCTTGTCCATTTGTAATAAAGAAAGTTGGTATAAAAGAGAGCCTGTAGTATAAGTTACTTACTCTCTACTCGCAGCACCATGAGTACTTCCAAAATGGACTGCGAAAATGGTAATGTTTCTGCTTTACTTGAATCTTAAGGCAATTTTTAATTCTTATTCACATTTTTAACAGAAAATAGCTCTCGCGCCACTTCTCAAACTGTTTCTGAGGAGGAAGGAGAGGAAACGCCCCTTGTTGCATCACCAAGTGACCAAAGGCATGTGCAAGAGAGTTTTCCCTCTCCTTCAAAATTTGATGGACAATTTGTTTTAATCTGCAATGAAAATGAAAGTGAGTGTACGAAGCGAAAAGTCAGTCGCGTCAGCTGTTTCCTTTGAAAGGAAATAAAGGTCTGTATATTTGTTAAGTCAGTACTCTGTGTGGTTTTAAATTGTTTCTCTAAACTATGTGCACATTTATTCTTTGTTTTAAAGCTCGATGAGGAAAGTTCTAGCGGACAGAAATCTGTGATACCTTTTGCTACACTGACATCCTCATCTTTGCCTTTGCTCACTATCTCACAAATTTCAAATACCAATCTCCGTTTTGCTTTTGAGGGCAAAATTATTAATGTGTATGGGACTTTTCAAACTGCTAAAGACTGTTCAAAGTATCTCAAAGCTGAACTTGCTGATAGAGAAGGTTGTAGCACTATCACTCTTGTCATAACTGAAGCGTTCATTCAAAAGTATCTTGAGAAAATAAAAGAAAATGAATTCATTAGGGTCGATAAAGGTACATGTGCCTTTGCTCTTAGTGTAGATGCGACTACCTTCATTGTCAAGGCTGAGCCGTTCCATGTATGCTTAATGTTTATGCCTGAGATAAAAATTCGCAACGTCCTTGCCAAAGCAAAAAATGAGCCTTTCTTGAAAGGGACCATTGCTTTTGTTGTCATTCAAGTAGATAAAGTCAAAAAAAGTAAAGGTATTGAAACTTTTGAACAATTGACTGTGGCAGACGGCTCTACTTCAATGGATTTGGCAACGGTATTTTTCTAACTCAACCATTCAAAAAATCTGAAGTGTTTTTTTAGCTCACATTTGTAAACAAGCGCAAGAATATCTATCGTGAGCTCGTAGAACAGTATCAGAAAGGATCTATTGTTATGTATGTTGCCCACAATATTGGTGTATGGAAAGGCCAACCGAACACACTGTCTGTTTTTTATCTTATTTAACTCCTGTGATGGGTTCAAAGTGTAAGGCAGAGCTAGAGACAGCCTTTCGTGTCCAAATTTCTGAAATGGGCTATAGCAAACAGGTGGTCTTAGTTCAAATGCGTTTCTTCTTTTCTCTTATTCTGTTTTTTTTTCTAACTTTTCAATTTATGCAAGTTTTTGGCTCTCTGGAGATTACAATTTTAATGCCGTCTTCATTGTGACAAAGTGTGCTGTCTGCAAACAGCCGAACATCAACAAGCTTCGCGAAGGAGGTTTCTGATTGACTGCGGCAAGCCGGTTGAGCTTTACGAAGTGCCTTTTGAAGCAAGGATTATCGCTCCTTCCGGACAAGCAACCGAAGTTACATTGGTGGGTGACGATGTCGACAAAGTGCTTGGGCTTGAAGATTCACTTGTTAACCTTTACCATAAGAATGGTATTCTCACCCGTGTCTTGCTGTCTGAAATTTCAAAAGTGGGAACCTTTCTGATCGATAACCAGGGTTCTGTTATTGGCTTCAATGCTCCTCCTCCATTACCATAACTATCAACCATGTATATTTTGTGTCATGTCTCCTTAGAAGCCTTGTAGTTATGAAGATGTTTGTCGTACACTATAACCCTTGTATCTATGAAGTCTGTTGTATACTATAATCCTTGTACCTATGAAGATTGCTCTACACTATAATCCTTGGTCTTTTTTGTAACTTTCCCAATGGACATTTTGCAAATATATGATGTTTTGTCCAAAAAAA |
| ***A. THALIANA* HOMOLOGUE OF YEAST SPO22 (ATSPO22):**  TTTTTCGTTAAGCAGGTCACAGTCGCAAATTGCTGATCTTCCAGGTAATCTGCTCATCTATTTCCATCAAAGCAGGAATTTTTCTGCAGTAGATCTCGTCATTTTCCTTTTGTAACCGAGCACTAGGAGCTTCCTGCAGAAGAAAGTGCATTCTATTGAGCTTCTGATCAAACAGCCTCAGGAGCCACGTTTACTTGAATCCATCGTAGCAGCTATTCCAATCTGAGGAACGATGCAAATTTCTGAGGTTTCTAGTGAACAATCTGCAGCCAGTGATATAAGCAATGATCAATCCTGCTTGCCGTTACAAAATTTGGAGAGTCTGGTTGTGGAGGTGGAGCAAATGGCTCCTATGATTAGTGCGGAAACTGTGCAGAAGCTTAGAAACCTCATTCCTACCTTCCCCTCTCGATTGAATGAGGACCAGAAATTTCGGCTATGGAAACTTACCTACCGCATCTGGAACACCTGCATTGAGATCAACAACAGCCTGCAACCAGGCCAAGATGTTGACGAAGAGCACGCTAAGCTTCGCCATGTTGCCAGTGATCTTCTTGTCATTGCAGGTAGTGTCAGTTCCGTACATACAAGCCTTTCAAAGACGGCTATGTTTTTCTTCAAAACAGGAACAATCTGGCACAAGCTTGAGAACCATGCCATGGCTGCTGCTTGCTTTGAGAAAGCTACTGAATTTAGTAACAGAGCCAAAGATCATAAGACAGCTGCTATGTCGCAGGCACAAACAGAAGAGGAAGAAAAGTTCTTGTTCGATTTGTTTGTGGCACGGGCAAAAACAGCATGGGAGCTTCAGCAGAAAGCTTTGGTTCGAAGCTTGTTGAGCAGCGCAAGGGGGATGTTAAAGCTGGCATCTTCTTCTTCATCTTCAACGCTGATGTACGAGGAGCTTGCAGAAGAGTACCTGCACTATGGTAAGGTGCTTCTTGCGAGGCAGGATAAGCCATCACAAGCAGAGTCTATCCAGTTTTTAGAGCTGGCCTTTGAGGTTTGCTCAGATGGAGCTTCGGCTGTTGCTGCTGCTAGCGGGGATGGCGGTGATGATGAAGACATGCTTGGGAAAACAGACAGGGAAACCCTTGCCTTGAGCAATCTGAAGTTCAAGGTTTTACGATACCTTGCTGCTGGCCATCTGCAGAATGACAATTTTGAAAGCGTTCTGAAATGTGTTGATATTCTGAAGGCAGGGCCAGACCATGCAAGCACACCATTCTTAGCCTTAAAGGCCTACGTGGGGCTGAATCGTTTTGAAGGGGCAGAGAAGGAGGCCTACATTCTGGTAGCACAGAGATCTACGCCAGTCGATGTATGTGCGGCTGCAATAGATGTGCTGATTGAGGGCTCATGTAGCTCGGCAACTCAGATTAGTATCAAGATTCTGCAAGATGCTGTGAAGAAGGCCTTTGCCATCGCACATGCCAGGTTTCCATCCAACAAAGAGCTCACTGTAAGGGTGTTGGATAAGCTTTTGACTCAGCCCCGTGACCAGTTCTCCAAAAAGAGGGTTGAAACTGCCCTTGCCATTGCTGTGGACGAAAAAGTCATCAACAGCATAGTTGAAATTTCAAGCAGAAGAAAATCTGCAGCAAACATTATCAACGAGTTGGAGGACGAGTCATCCACTGGAACTGCTCAGCAATGCATCTACACCTTGCTATGGAACAGGTGCAGAGTAATTCTCACTTCCTGTTTCTATCTTTCTTCTCAAATTTATATCCATTCAAATCAATACTGGCTGCAATCAAGATCATTCTGGACTATGAAACAACCTGGCCTCGCTTTGCATGAAGTTTTTCTGCAATTCTCTGCAGTCTCACCACAGAAACCAAATTTAGTTGCTGTGTTTTTTTTTTGAGGCATTTCATGTATCTCCTCCGTCGTCGTGGTCTTAATTACAAGGCCAGAGTTCAAGGAGGTCCGATTTTCTTTTGCAAAATAATGCAGTCTGGCCTTTGCCTTCGTATGTTAGAACTTGGATTGTGAGATGCATGGCTAGGGCTGTAGTTTTTGATAGCTTGACCGGCGTGTTGCGGTAGCCTTCAGGGCCCTGATGTCCACGAGTTACTCTATTCTTTGAACAGCTTGAGAGATAACTGATTTCTAAATTTCAAAACTGCAAGTCTCTAGTACTTGAGCTGGATTTTCAGCATCAACGTAGTTGCATTCTATCAACATTACACTCACGGATCTGTCAGAATCTGCTGCAGTGCACAAGAATTTTTTGTAGCCAAGGAGTATGCAACTTGCATTCAGTTGTTTGAAGCATCTCTTCTGTACCTCTCTCCAAAAGACAACAGTTACACCCAGCACAGAGCCAAGAATCTTCGTATCCTCTGCATTTGCCATCTTGCCCTTGACCAGTATGATCGAGCCTCAGAGTATATTAATGAGGCAAACAAGCTGGAACCTAGCATTGCTGGAGTATTTTTAAAGGTTGAATGTGCTCATCTTCTACATCAATTTCAATCATTCGAGTGTCTTTCTTGAAGCTGCCAAAATTGTGCTGTTCCTTGGAATTTTGACCTTGGGCCTGCTTGAGGCAATGGAGAGCTATCTTGAATGTTGGTAATTAGGATAAGCATTGAGCAAAGTCATTTTAAACGGACCATTTCTTGAATTTTGTTTTCCAGTTCAAAGTGGCTCTTCAGACAAATGATGAAGAAGGGGCAGCCAAGCAGGTTGAAGAAATGTTTCGGTGCCCAGATTTTGAGCCAGATTATCTGACGTTGGCCTCCCATGAGGCCATATCGTGCAAATGTATTGGCGTGGCCATTGCTGCACTGTCACGCTTGCTGGAGCTGTCATGTTCCTCTGCAAGCTCTAATACTGGAGACAATAAAGAGGCAGTATTAATCAGAAACATTATTGCACTCTCTTCTCAAGACTCCAGCAATTCAAGCACTTCTCTTAAATATTTCAAGCTTGCTCGATCTCGTCTGGCTGAGCTGGGACCTGAGGGTTTCTTGGGGTGTGGGAGTATGGAGGAAAAAGAAGCTCAGTGGTATGCAGGCTCTGCATGGAACCAGGGCTTAGATACAGTCAAAACTCAAGAATGGGGCTTGTGTAGAGAATTCTTTCAGTGTGCTGCTGATTTCTACGAAGTTCTCCCTGACACCCTTGAGAACCTTGAGATGAGGAGAGCATCTATGCTGCTCGCTGTAGGTTCAATCCTTGCAGGAAACAGAAAGGATGCAGGTCTGAAAGAGGCTTCCATATATCTTGAAAAAAGCCGTAAGGTAAAGTCTTTGAGTACACTTGTCAACTTGACATGTAAACTACTGTCCATCATTCTTTGAAGAAAATTTGATGGATACAAACCAATAACCTCTAAACCAGGACCTAAATGATCTCTATTAGGAAGGTTAAGCCATTAAAAGAGTCTAAAATTTGGTTACTGAATCTGTTTTGCGCCTCAGATACATGAGATTGTAAGGTCGAAGAGCATTTCAGGTGTTAACAATGATACCACTGAGATTTATCTGAATCTACTCTCCTTCGAAGTGAAAAGGAGGATGAAGGATCATAAAGCTCAGCTGGACATCATTTGCCATTGCAGCACACTGCCAGGTTTTCAGCCAAAGCTCTTTTTCTCAATGGGATCAAGCGGAGGAGAAGGCCAGGATGTTGAGGTCTCCATGGCTGCATTTGAGGCTTGCCTCAACTCAGCCTTGGCTTGTCCAGATCCTGATTATGCTTTGGTTGCAGCTACCATAAGAAAAATGATAACACTTGCAGACACATTGAGACGAGACAGCCCTAAAGCAATGAGCTTGTATAAGCAGGCACACCAGATACTGCTTGGCCTGGGCATCGGAGTGTACCCAAAAGAAGAGGTTCAGTGGTTGGTTTCGACTGCTTGGAACAGAGCATCTCTCCATGTCAAATTTAATAGATACTCAATTGCAGAAAAGTGGATGAACTTGGCCCTGGACATGCTTCAGCATGCACCCTCAATGGAAAATGTTAGGTCTGTCATGCTGGAGAACCTTAGTTCCGTGCTGAAGGGAAAGGCTGATGAGATGGATGAATGAGCTGGACAGAATTAATGCATACGAGTTGAAAAGGATTCCCACAGTTTCTATTTGCGGGTCTCTTTCTAAATTTCTGCAATATCTCTTAAGATCAAAAGGATTCTACAGCTTCTAAGTTCTAATAGTACTGCCTTATAGATATTCTTATGGTGAAGGAAATGAAATATGGGCCACTCTTTGTCTTTTGGTGTCTCTAGCTGATGCCTGCATTGTCTTATCAGATGACAGCTTTTGCTTGAAATGGAAAATAATGTTTGGTCGAAGTTGATTGGACAATTATACCTTCTGTGAAAGAGAAGCTTTGGACCACTGTATTTCTCAGGCTTATGGAAGCATTTTTTTAATACCCACTTTTCATTTTTTGATACGCTCAAATTCGTCAACGATAGGGCTCAAAT |
| **ACTIN DEPOLYMERIZING FACTOR 11 (ADF11):**  GAGAGAGAGAGAGAGAGAGAGAAGTCGGACAGAGAGAGAGAGAGAGAGCTTCCTGAAGTCTGTGCACTACGTCGCGTTGCCATGGCTAACGCTGCTTCTGGGATTGCTGTTGATGATGAGTGCAAGCTCAAGTTTTTGGAGCTTAAAAGCAAGAGAACACACCGCTACATTGTGTACAAGATTGATGACACATTGAATAAGATCGTGGTTGAGAAGCTTGGGGGACCTGAGGAAAGCTACGAGGCCTTTACTGGCTCTCTTCCTGAGGGAGATTGCAGATATGCCATCTACGACTATGACTTTGTTACAGAGGACAACTGCCAGAAGAGCAAGATCTTTTTTGTAGCGTGGTCTCCTGATGTGGCTCGTGTGAGGACTAAGATGCTGTATGCTAGCTCCAAGGATCGTTTCAAGCGGGAGTTGGATGTGACCCATGAAATTCAGGCTACGGATTCCACTGAAATTGACATAACAAATATTCAAGAAAAGGTTAACTAAGGAGCTCTGCAAGTTACACAAGAATTTATAATCCCTATGCAGTGTTCTGGTGTGTTTGTGTATAGTTGTATTTTGCTCGTTGCATCAACTCTTTGCATTAGGTTACTTAATTATGTATTGGGCCATATACTCGGGCTTTCACCTTACATTATTGCCAAGTTTGGACCTCACCAGGGTGATATTTCTTTTCAAGGAGTGGTTTTGATCTTCTACCATTTTGCTGCTCTTGTTCTCTCTTTTGCAAATTTCACGGCCTAATGAGGTGACTAGGCTCTACCCTACAGTTGGTTCAAGGTTGTGCCGTTTATCGGTGATCAGGCTGTACGCGATTGGCGACTGAATTATTAATGTATTTAATACTACTCTCTGTCTCTCTCCCCAATGCATTTTGGTCTTTGTGCTGAGGGGAAAAGTGTTCATCATAGTGGCAACAAATGCCAATGTGTGTAGTTACTGTCTTTGAGGTGGGCTTTCAAAGCGGGGAGAGAAGTGTATTTTGTCTTGATTGTGGTAGCTTGGCTTTCAAGAGATGCAAAGCCTGTAAGATGTGTCTAATGTGGTTT |
| **ABERRANT LATERAL ROOT FORMATION 4 (ALF4):**  AGAGTGAAAACAAAAGAACAAGATGGACAACAAACTCTTAAATTCTAGTCTGTGATTACTTTTGGGGCATGTCAAGATCGCTAACATTCAACTTATTCAACTGTAAGACTGATGATGAGGATTTGGATGTCTTATTTTTTTCGAGATCATTCTATTAAAAAACACCTTAAGGGCAAGGTCCAAAGTGGATGTCTTCTTCACATGGACTTGAAGGAACATTGGCAACGAGGACGCTAAGAATGAGGGCGTAGGGCATGAGCAAATCTTGGGAAGAGGGTCATGCACGTAACTTTTTTCCAACACTTGGGGGAGCCGGCTGCATAAGGTAAAATACGTGCCTTACCAGCTGCTGCCAGTTCAGGAGATTACAGGTCCCAAGCCCCCCCTCCACATAGGCAAGCGAACTTGATGGCCCTGAATTCTAGATAATTATGGCCCACACCCTCTCCAGCAGCTATAGATGCTGCTGTGAAGGACCTATGCGGAGGGGCTCTAGACATGCAAGCTCACCAACCTTGACGTATGACGTGATGTTTACAACATGGCTGGGGCATAACAGTTTAGAAAAAGCTTGAAGTGTTGCCTGACTAGCATTCCTCTGTTTTGGCACATCGTACTCCATTTCAGTGCATCATTTGCCGAGAGGTTAGCACTTGTCGTCAGGTCATGTGATCCATGCCCTTCACCATACCGAGTAATATTCCAAGTTTATGCAGACTGGCAATGACTAGCTGATGTTGGTAGCCTAACATACAGTAAGCTCAAGGAGCTGGTATTAGCCAGAATCTTCTTTCAGTGCTACCTCCGCCAACTCAAGGCATCGGTATAGCACCCCTTCCAGATTTGATATAGATAGAAGAACATTGGCAGTAACTTCAGAATCATCAGCAGAGCAATCCCTTTTAAAGCTTTCAAGAAAAGAACGAAGAGGCTGGAACCATTTTGAACAAGCTTCTTGAAGTCTTGCTTTGAACAAGACCCCTGTATAGTTGGACTTACCTGTACAGAGCAATGACTTACAACTTTATTTTCATGGTTTTTGCAAGTTGCGAAAAAAACAGTGAACAGTAACCTGTTGTTTCCTTGATCAGAAGAAACCGATACAGGTTTAACACTGACAGGACAGCATCTATCTGATTTGGGAGGTCAGGAGGGCCCCCAACAGGTGGATGCAGAACAAAATCAACAAGCTCCAGCACTTGCTCACTTGCGAAAGGTGAATTTAGAAGTGAACTAGTTCCTTGGACTGTGCTTTTAGATGCTAATGCCTTGTCCGCTTCCTCTTTGACAACATAAATAAGAAGAGATACCATTGAAGAATGCTCGCACTCGATGATGAGTTGCTTCAAAGCTTTAAATCTTTCGATTGGAGGAAGTACTTTTCTGACAATAATCACCAAGACACTATATGCACGTTGCCTTGTCCTTGACTGTGGAGAAAAAATCACCACATTTTGAACCACCTGCAGAAGTTCTTTCAGCTTGAGAAACTCGTTGGGCTCCAGAAACTCTAGTTGGATGTGAAGAGGGTTATCTACAGAAGGCGTTAAGCCCCACTCCAGAACGATGGTCAACAAATCAATGCCAATCTCCACTTTTTTCCATGTTTTTTCTTTTGAGGAGAGGAGGACCAAGACATTAGACAATGCACTTACAAATGCATGCCTTGAGCTCCGCACTCCATCAACTACAGATATTACCTCTTGATTATCTATTGCTGTTTCACCAAAACAGAACCTCCACCATACTGCAAGAGCTGCCCCTTGTCTGGTGTCTAAAATGTTACCGCCCTTGTTGACAAGATCTTCACTCATGGAATCTATGAAACTTTCAAAAGGGCTTCCTGCAATCATATCTAAATAAGACAAGCCACAGGTGGGCATTAGCTTAGCAAGTTCCACCAGTACAGATTGTACAGCATCTGAATCATTCAACTGCTCAAATTCAGAGAATCTGGCCAGAAGTAAAAGAATAAATACACCCACTGCTTCATGTAGACTTTGTCTTTGTTCTTTTACCTCTTCTTGTGCACCAATGTCAAGTAAAACCATAATTGTTTGAACCAATTTATCAAGGATCATCCATGGCTCCCAATTGGTATCAATTGCAAGGCCAGCTATTCTCAAGAGGCCTGGCAAAGACTCCTTAAAGTATTCAACCCTTCGTCTTCTTAGGCGAGTGAAAATTTTGGGTAGGCCATCTAGGATGACTGGGCAAAGTGCCAGAGTTTCTGGCTCAGAATACAAATGCAATGCCTGAAAACATGTGAAGCACATTTTCAGAGCAAACAAAAACAGCAAGTGGAACAAAAATGCAAAGCTACCTCCATGTAGGCTGCAAACATGTCGCGTGGGTCACAGCTTTCCAACAAAGTTTTCAGGATCAACTCTGCAATCCCTCCACATGATCCATGTGGAATGATGGCAAACTTGACAAGAATAGATGGCATATCGATTCCAACGATCTCCAGTAAATCCTGACCAGTGGGACTGGATGGGTCGAGATGAGCCTTGACCTCCGCTAACAAGGTCTCCATATCATCTGACGGCTTGCCACCTCGTTCATCTAATCTCTGCGCAGCAACTGACACCCTACGTACGAGTTCCTTGCCCGCTGCTTCCAATTCCATCTCCACCAATTTTTCTTCAAAACATGGCATTATATTTTGCCATTTTC |
| **TORNADO 1 (TRN1):**  AGGAACTCCCTTAAAATACAATGCTGCAATTTTTGCTCAACTTGCAAGGAATTTGAGAAATGCAGAGCTAGCAGAGTTGCTCCAACCTGAACCCAAATATAATATCAAACCCAAACCCCCTCCTAAATGTAGCAAATCCAAACCCATTCTTAAACTCAACCTAAATGTTTTTGGAAGGTTGGACTCAGAGGAAGAAGTTAAACTCAAACCCCCTCCTAAACGTAGCAAACCCAAACCCATTCTTAACCGTGCCGATGACTTTGACATGGTTAAACCAACAGCAGTTAGACTTTTTCTTTGTGGCCTTCCTTATTCAGGAAAAACCACCCTTTCTCAGTCTTTTGTGGCATATAAATCGCAAATGGTTGGTACTTCAGTTCGAAGGTTTCACAGGGCTCATATAAACCGGTTTAGGGTTAAAGGCCGCGTGCCAGAACGGACACGTGGCATAGAAATAACCACTCTGGAGGAGGATGATTTGAAGGTTTCGCTTTGGGATTTGGCCGGTCAGTCAGAGTACTATACCTTTCATGACTACATGTTCCCCAATCTTAGCACTCGGGCAACACCAGCTTTTTTCTTATTTGTTTGGAATCCCATCGAGGTTGATGAGCGAGGTCGTCATAAGACAGATGATAATGGAGCTCTTGTTGAAAAATCTATTGAGAAGTTTGAGAAGGAGTTCAAGTACTGGCTTCGATTCATTGCATCCAAAACTCTAAGGTCAGAAAAGTTCAAGCCTAGGTTGATAATTGTCATAACAAGAAAAGATTGTAATTTGAGCCACATGAAAGGATGGACTCATAGCACCCTTCATAATCTTCAGGATACTTTTGGAGACATCATCCAATTTGACATGAATGGACTTTTTGAAATAGATGCCAGGGATCCTATGGAGCTAAAGGGATTAGCTAAATATTTCTTTGATTGTGCAAGAGATATTCTGGATTCAGCACCAAAGGTGCATGCGGCATGCAAGGACGCAAGGTCAATGATTGCAACACTAGTGGACACTAGAAGCATCGCTCCTTTGATTACAAGAGATAAATTTTTGTCTCTGTGTGAAAAGCATCTTTGTATCAACTCAGAAAAGGTGCAAGAAGCAATCGCTCTCTCTCTCAATGATTCTGGAGATGTAATTTATTTCGCATCACTTCAGTTTGTGGTGGTAGATACACAATGGTTTTGTCGTCAAGTCATGGGTGACCTGATGTCCAAGAGCATTCAGACTCAATCCAATGGCAACAGAAATCCTCAAGGTATCTTTTCGGTAAAGTATCTAGAGGATTTGTTGGAGGCACAAGTGAACTCAGCAGCATGTAGAAAGTCTTCTCGAATCAGGCACGCATTCAAGGCCTATAGAAGAAAACTGGTGACACATGCAAAGGTGGTGGGAGAAGACTTAGTAAAGCTGCTAGTGGAGTTGCAACTAGCTTGTCGTCTGAATGAAGATGAAGGGAACTTGAATGTTTTCATACCTGCAAGTCTCTCTCAAAAGAATTATGCTTATGCACCACACTTTTCCTTAAAGCGAGAAGGCATCAAGGCATTCCTTGGAAGACGGCTAAAATGTAAAGACTCAAAACGCACTTTCCTCACACCCGGTGTCTTCCCGATAATACAGGTTGTTTTTTATAGCACCATCAAAACAGCCAACAAAAGAGAATCTGCTGTTACACTTGAAGAGGATCTCATTTCATATGCTCAAGAAGGTAATGGTGTAATAATAGAACTTTTTAAGCAGCAGGGAGAAGAACATTTCATAGATATTTTAGTCTGCTCTCATCTTGAGCTAGCTCATACAATCAAGTGGGTTCATAAGAACGTAATCACAATCATAGAAAGAGTGTGCGCAGAACCGAAGGGAATCCAAGGTGTGGAGTTAAAACATGAAGTGATTCGACCAGAGTGCGTGGAATACATTGACCGAATAGAGAAAAGAACACAGTCGGTTTCTGTGACAGATTTGAGAGAGAGGTTGGAGAAACATTTGCGAGAAGAAGGATTTGGGGCAGATGGTTTCAATAATGTGCTTCACACTTGGATACCTGGAGGAAATTCACATCGTGTTGATGAATTGTTGGGAGAGGAGGCGACCCTACAGATTGTTGAGACTTATTGGCGAGGTTTAAGTGTGGCTGCTGCTGATGCATTAGGTGTTGTGCCGAAGGACATAGGTACTAGTAAAATAGAGAATAAGATGGATACTAGCAATCTACAGGAGCAGCAGGCAGGCAAAGATAATGTTGGAAAGGGGCACCTTTACGATGGCGAGGCAATTTACAATAAACTGGATGAAGTACTTCTGGAGGTGAAGAAGCTGCGCAAGGAGCAGCAGGCAGGCAATGATGAAGTACTTCTGGAGATGAAGAAGCTGCGCAAGGAAATGCAAAATCATGGAAATAAAATTGACAAGTTGCGAAAGGCTGTGATTCCAAAGCTGGAGGATCTGTTTACATTTTTCAAGGAGGGGGAGGCAAAGCTGCCAAGAATGCTTGTGGTAAGCAAAGGGCAAAGTGGGTTGGTGAGCATGGTGGCAACTGCAATGGTACGTAAGCTTAGTAGCTGGCGTCTAGAGCTTTTGTGCGAGTGTGAGAGTGGACCCCACTCTGTAAAAGGGTACCCTGGAATTGACATTGCAACCCTAGAGGATGGACTTCTCAAGCGTGGCCTTCCTTACATAAACACATTCTTGAAAGTGGCCAATGTGGCTCTCAAAGTGGGCGCTCATGTGACTCTCGGTCTTGGGAACGTGGTACCAGATTTTCGACATGTGCTTGCATTGATAGAAGACACTAATGAATTTCCTGGCTTGCCAACAATTTTCGACCCTGCTGATCCTTTGGGTACCACCTCTTTCGCATCCAGAGAAAATTTTCAAAAAGGCCAACAATGGCTTCTTCAAGTCCTTTCCAATCGAAATTATCTCTCTGATATCCAGATAAAGGAAGTCTTTGGACTTACTCGTATTCGGTACCCTAGCCACAAAATTGCATGGGTGTGCAAGGAACATTCCCATAAAGGTCAACTCTGCCCAGCATGGCTTTGATTTGTCAAGTTGTGGTCCATTTTCTTATCTGCGAAGTCTAGTAACAAAAACTCACTGGGGGCAATAACCTCTGTTTGTAAAAGTTCAGTCACTGGAGGAAATTTGGCTCTTCTGTATTCCAAGCGTGCGAAATTATGCATGATCAATATTTGTAATTTAATAAGTTGTATTTGAATGATCAAACACAACAGTGTACCCAAATGGAAGTAATTTGGCTCCTGTATCCCCAGCGTGCGAATTTATATATG |
| **HOMOLOGUE OF HISTONE CHAPERONE HIRA (HIRA):**  ACCATCATACTTGGCATAGCCCTACTTCCAGCTTTAGTGTATACCTGTAAAGCTCCATCCTGGCAACCTGCAGCAGTTACAAGAAATACCACTGGCCTTTCCCGTCAATTTATCTCGCCACTTGACATCCCCCACCCTGAGATCAGAATCAGTCTTTTGGGGATTCATGCTGGCTGATCCACCACTGAGACTCGAGAGGCCTTGCTTCAAAGCATATTGGCAATCCCTGCTGATCGGCCTCTTGCTTGCTGGTGGAAACAATTATGGAAAGGACACCTTTCCAAATTACCAGCAGCATCAGTCGAGCAACAGAGTTGGTTGCTGTGACAACCTGATGATTGCCAGCTGTGTGGCCACCACCTTGGG |
| **GROWTH REGULATING FACTOR 2 (GRF2):**  CCCACGATGCATGTGCCTCTCACAATACTTCTGATCGGGCACTACTGCCCTCCCGCACCTCCACTTCTTACCATCCGTCCTCCGGCATCTCCCTGGCTGTGGATCAGCCAAACCCAAAAAATAACTACCTGCATTGCTGCCAAAATTAACGGGCACATTCTCCACTTTACAGCCCATGGGTAGCAGCAATGCTGATGGTGGTGTGTTGCATGCAGCCACATGCTTGAGTACGTACGCCTGCTGTCTTAACTCTGCCCATTGATCTGGTGTTAAAAAAGCCTGTGTTGGCCCTAAGTAAGGCAAGCTCCCGTGATGAGATCTTGCAGCAGATGCAGCTTGGGCAGCCGGAATTCTGCAATCTTCAAGCGCAGTAGAAGCATAAGATCTGTTCTGAGAAAGCAAAGCGGAGCTATGGAGACCATCGGCACCACGGATCGCCCCCACCATGTTATGTGGGTACTGCAGAGAGCTTGCACCTAGGGGGTTTGCTATGGCAGATGCGTGATACTGATGATGACGAGGAGGCTCCATGCGCGCAAGCTTGAATGGCCTTGTATCTTCTGTAGTGTCGTGAG |
| **RETICULATA RELATED 3 (RER3):**  GTGGGGTTGGAGGTGGGCGCGATGATAATCATGCAGATAATGAAGGCCAAGGTAATTCTGATGATGAAGGAGGCCTCTTTGGTGCCCTGTTAAAGGGCTGGAATGAAAGGGTAAATGCAGATCCACAGTTTCCGTTCAAGGTTTTGATGGAACAAATTGTGGGTGTGGGTGCGTCTGTCATCGGAGACATGGCTTGCCGTCCCAATTTTGGCTTGAATGAGCTCGATTTTGTGTTCTCCACCCTTGTTGTTGGGTCCATCTTGAATTTTTCCCTCATGTACATGCTCGCCCCTACGTCTCTGGCTGCATCTACAGCAAGCACACTGCCTTTCATCTTCTCCACATGCCCATCGGGGCACATGTTTGAACAAGGGGCATACTCTGTTCTTGACAGGTTTGGTACATTTGTGTATAAAGGAGCTGTTTTTGCTGCTGTTGGCTTTGGGGCAGGTCTGTTCGGGACATTCCTATCAAATTGTCTCATTGGTCTCAGAAAGAAGATGGATCCGAGCTTTGAGCAGCAGAATAAGGCGCCTCCAACTCTCTTGAATGCGTCTACATGGGCTATCCACATGGGGCTGAGTAGTAACCTGAGATATCAAGTTATCAATGGAATGGAATTTGCAATGGAAAACGTCTTATCCCCACCTGTATTTAAGGGGTCGGTTCTCTGTTTACGTGGATTCAACAATGTGTTGGGAGGCTATTCTTTTGTGACATTGGCAAGGTTGACGGGATCTCAAAAGAAAGCAGAGGCAAAAGAAGTTCCTTCTCTTGCTTCTAAAGCACCTACAAATGAATATGAGGAGCCCAAATCGTCTAATACAGATCCCTTATCTATTGAAAAAGTAGTAACCACAGAAGGGAATAGCACTAACATTGGAGAGGGTAGCGCTAAAGTGGGGGATGCGACCTCACAAAAAGGGAATAGCACTAACATTGGAGAGGGTAGCGCTAAAGTGGGGGATGCTACCTCTTAGCGGGGCCCCCAGTTTTGAATTCGCCAATGCAGTATAAAAAAAAAAAATTCCCTCTGTGCAAGCAGATCCCTAACCTTTTGCAGCAATCAGTTAGCTGTTTTGTGCTTCATGCTTGGTCTTTTTGTAGGTGTAGCAGAATGTTGATCAATGTAGTTTCTCTTTTCATTTTCTGTTGTGAGCAACCATGATACACACGTTGAGAGTTTGAGCTGAGAGCTTGAGTAATCTGCTTCTAAGTATCCCTTTGTAAAAGTTTGATGTCTATTAATCATTAATCAGAGGAGTAATGAAAAGTCTCACATTTCTAGCTGTTGACCGTGTCTTTGCTACAATTGTAGTGGATTTCTTATTGGCGTCGAAAGATCGAGTCAAGACTTGAGCCACAGAAGAAGGCTGAGTTGCTACTGCCCGTGCAAGAAGGCGTCTCTGTGTTTACCTTGGCCATCCACGTGGGGCTCGGAAGCAATTGTATTTATCTTGTAATGCAATGCAAGTGACTGAGTTGTTGCTTCTCCTGTTTGCTGGCATGGGTTGGGAAGCAAAGGTGAGGAGTCTAACATGACAATCTAGCAACAGACAGGAGACATTAGCTTTAAATTCAGCCGTTTAACTCATCGAAAAGAGGATCTTTTGAAGGAAGAGCCCATCAGTTTTCATTTTTTAACAATACATCTGTTTTGAGACCAGGTTCCTACCCCCAGAAGCAAAACTCATCGATCTGCTCTTTAGGTCTTCTTCTCAACACTCTAACCGATTCTTTCTTACGAGTTAGCAGTCCAAATTGGCTTGGAATCCTGCATACCAAGTATCATTGGTCAAACCTTCGAGTCAAAGTGATTACCTACAATACGGACCGGGTCCAGGAAACATTCTTTATATGCATGCATTTGCATAGTTCTTGTGTATTGCTTGCAAGCAGTTGGCAAAGGTGCCACATTGAAGCTAGCTATCCTTCAGGTTTGTTTAATTCTGTTGTTGCTACTGTCAATAACATGTTCCTTGGTTGTCTTGGATTGGGTGTTGTATTAAGGTATTTTATATGAGCAGGAGGATTTGCAGCTTTG |
| **RETICULATA RELATED 4 (RER4):**  TACCCAAGCCAAGTTGCTCGTGTTCAGTAAGTATAGATTTCTTGCAAATAGAACATAAACACAATCTTGAATAGAAAGACACTAGATACATCTTTTGGCATGATCAATTTATTCATAAATGAGATCGTAGTCCGATGAAAGACATGTCTAGGCAGAAATCCTCAGCCAAATGCATTGAAGGCACATCCGGAAAGCTTAAAAGGATAAAAAGCAAACACATTGCAAAACAATGCTTCATCGATCCACCATGATGCGGGGGGGGGGAACGTACTGCCTAAGGTCTCATGTATGCTGAAAGCATAAGGTCTCATGTATGATGAAAGCAGACTGCAAGATATGCTGTACATGAAACAGTCTCATAAAGATAGACCCAGTCTGAGCAGACCCAATCTGGCCAAAGAACTGAGGACTCAATTTGAGTGACACCAGTTACAATGCTACAGCACTAAATTGATAAATGGTGTAAACATCCCTGCCGCTAACATTTTGTATGAGTCTGTAATAAATGAAGCTGAAAACTACTTGCTAGAAGAGTTATTCCATCCATGAGAACAAATTGTTTGTGGCTGGCTATCCATGATTTGTATGTAGTAATGGTTGAATCTACAAAAAGCTTTCTCTGAACTAAAAAAGTGTAAGCTAAATGGATGCTTCTTGATAGAGTGGGCATTGATATTTGTCAAACAAATACTAAGCTGTGGTCTCAGAATCTTCTTTGATCTTCTGAACACCCACCCACCGAGCATAGTCCACCCACATCAATGACCCCAAGAAGGTATTGCCAGTTCGAACAATAAAGCATAGAGCACTCAGCGCTAGCCTGTGATTCTCTAGCATAGGTTGAAGAAACCGCTGCTCCACTACACCAGCCAAGAATTGATACCTCAAATTGCTGGATACAGCTGCATAAGCACCATAAGCAACACTCATGGACAAAATAGGCACATCCTCTGTACTGCCAGCGTAGTTCTTGTTGAGAGCTTTCCTAAGATAAATCAATACATTTGTTGCAGAGGTACCCACAAGTGACGCAGAAGTAGCCACACAGAAAAGCTTCCCACCATTTCGTACTATGGCTCCACATCTTTGTAGCAGACTGTAAGAAGTTCCTGCCAAAGCAACCTGGAAAGCATTATCGGGACAACCTCGAAAGAGGTTTGCAAAAAGTCCAGACTGCTTGGAAACTAAAGAGCCGAGAGGGACGGTTGGTGCAGGAAGAAATACCAACATAAAGTCAGCAATAATTGCCATCATCACGTCAGCAATAACAAAATCCAGTTCTTTGGAAAAGTTCTCCCTTCGGCGCTCCAGTTCAGCTGCCGTCTTTGTGATGGTACCAACCCCACATTCAATTGCCACTTTGGTCATGAAGAGGTCATCAGCCAGCAGCCTTTCTTTCATGCCTCCAAATTGAAGCAGCCATCGAAAAAATGGAACCTTCTCGAGCTCGAAATAGCGGCGTACAATAGATCCAGGAATTTTACCCTGTTCTACAGCTCTCGCAAGATCTGCGGGCAGATCACTAAGTGCCTTGCCCAGGCTTGCAAGCACAAGCAATGCTTCTGATTTATTGCTTGAATGTCCTCCCGGCTGCGACTCCCCATCGCCACCACTGCCTCCTCCCCCACTGCTGCCTGCTCCTGCACCATCATCTCCCCCTCCGCCACTTTCAAGTACGGCAGCTTCAAAGGACGCAAGCAAAGGCAGGTTGGGAGTGGACCTGCTGCTAACAAACCCAAAAACTCTTGCAACAGGGAGCAGGGGGGAGAAGTTTGACAAGGAAAATTTCTCTGCAGGTAACTGGTGATGGGCGAAGGAGGCAATGTAGGAGGGCGCATGAGCTTGGAGCCTGCAAGTGGGAAATGCAGAAGGAGCGCTGTGAGAGAAGGAAGAGCTTACAGAAGAATGAAGATTCATCTGTGCGAAGGCCATCTGAGAGGTTTAGGGCTTTCTGCGAGAAGACGCAGGACTCGCAGAAGGGCCTGCAAGAAGAACGCAGCAGATCCTTTCCTTTTCCCCGTTCCAGCAGCCGCTTTTCCTATGAATATTTCCATTATTTACTACCTTTGTCCCTTCGTGTAAGGTGG |
| **SHOOT MERISTEMLESS (STM):**  AATTACCTGATTGCATGTAAACTTGACTCAAAAAAGTTGCCTCCAGAGCTTATTGATCGACCTCAACACACACACACAAAGAAAAAAGCAAAACACATGTATACATAGACGCATACACACACATACTCACTGGCACACAGCGCTCTAAAGTCTCAAAAGTCATACATACACATCCACAGCCATGCACATGCACATGTCTCCAGTAACATTGGTAATGTTTGAGCTGCAGAGATAAGGGTAAGCAAGAAAAAATCTATGTGTGTAGTGACACTATGGACCTGAGCGCAGCTGATCATCAGCTCTTGTACAACAGCGCCATGGCCGGGGCCGACGCAAGTCATCCTTCTTTTGGGTCCATGATGGCTCTCATGACTGCAGAGCAGGATCTTGAAGGAGCTAGAAAGTCTCAGGTTCATCATTCTCCTCATTTTATATACTACAGTGATCCTTACCAAGCTGCAGTTTTTCACACGGACCAATCCGGCATGCCCGATCAGCTCTCGACATACGACCTCATGATTGCTGCAGTGGACGACGCACATGCGGCATCTGTACGACAGCTTGATCATGGAGGAAGAAGAGAAGGAGAAGAAGAAGATCAGAAAGTGCAGTTGCAGCAGAGCGCGGCGACGACGGTGAACAAAATCCACCCCAAATCGGAGGTCGTTCCGTGCGGGAACATATCCGTGTACTCACAAAACTCTTCTCACAGCAATGAAAGGAGAGGTTTTTCTGCAGGTTTGAGCTCTGGGGCATGCAGAAAGGAGCCAGCAGAGCGCGGCGACGACGGTGAACAAAATCCACCCCAAATCGGAGGTCGTTCCGTGCGGGAACATATCCGTGTACTCACAAAACTCTTCTCACAGCAATGAAAGGAGAGGTTTTTCTGCAGGTTTGAGCTCTGGGGCATGCAGAAAGGAGCAGTCGGAGAGTTTATGTAGTACGAGCATTACAGATGATTCAATCAAAGACAAGATAAGAGCTCATCCTGAGTATCACAAGCTGGTGACGGCATACATAAACTGCCGCAAGGTGGGTGCGCCGCCAGATGTCGCGAGGCGGTTGGAGGAGCTGAGCAAAGAGTACGATAACCCGCATCTGCTCAGCGCCATCGTTTGCAAAGCTGCTGCAGCTCCGGATCCTGAGCTCGATCATTTTATGGAGACCTATTGCCATGCTCTAAACAAGTATGAGGAGGAGTTGTCTAAACCCTTCAATGAAGCCATGGCCTTCCTGCAAAAGGTGGAGCTCCAAATAAGCCATGTCAGCAGAGCGGAATTCCGCGTACCTCTGCAAGGTGATGGGTCATCTAGGCTTGGCATGGAGGACACAGAGCAAGACATCGAAGATGAGGAAGAAGAAGATGAAGGTGCCGGAGGCTGTGGCGAGGTCGATTTTGAGATGGATGCTTCCATGGACCCCCACACTGAAGACAAGCAATTGAAAGAGCAGCTTCTTCGAAAGTATAGAGGCTACATCAGTACCCTCAAACACGAATTCATGAAAAAGAAGAAGAAGGGCAAGCTCCCCAAGGATGCTAGACAGCAACTCCTTGACTGGTGGAATGAGCACTACAAGTGGCCCTACCCAACGGAAACGGAGAAGGGAACGCTAGCAGAGACGACGGGGCTCGATCAGAAACAAATTAACAATTGGTTTATAAACCAGCGCAAGAGGCACTGGAAGCCGTCCGATCAAGATATGCGCTACGTGATGGTGAACGGCCAGGATCCTCCTATGCAGCACGACCACCCGCCGGACCAACTCAATTAGACCATGCACGTGCAGCACAACCTTCTCAGCAACTCTCTCATAAAAAGAACCAACGTGTACCTTTTTACTCCTAATTTTGTAAATGTTGAATGAGCTTCAATATATAAATTTTATTTCTTGCAAGCTCCGAATTTAA |
| **ENDOPLASMIC RETICULUM AUXIN BINDING PROTEIN 1 (ABP1):**  GAACACTCTTGCAACAATTTTGCCATTATAAAAAGAAAATAGAAGAGAAAAAAAATCACCCAACTGATATCCACAACATTCTTTCTCAACTAAATTACAATTAGAGCTCCAAAGATTTTTTTCTTGAACGTGTGCAAATCTATAGTGCATGGCTACTTTTACACTTACAAGTATTGGGTGTTTTACATACAGTAAGGACATTAAGAAACAATTTTTGTAGATACAATGGTTTGTTACAATTCATCAAACATATAATCTGACTCAAAATTCTTTATCTCATTTTCCAAACACATGGCATCCCACGCGTATGGAAACTTTAGTACTGCTGCAGTATGTGGAGTAAACCAATCCTTGTAAACAAACGCTTTGATTGGAGGCCGTGATATGATCACAAGGACTTGTAAGTCTTCTTCCACTTGTGTATTTTTTACCTGATGTACATGATTGATAGGAATTGTGAATGTAGTATTTGAAGAAATCATGAATTCGTTAGGTTGTCCCGGAATCTTCTTGCCACTATCAGGTTCCATATATAGAGAGCCTTTTCCCTTCAATGTTACAAAAACTTCTTCACATGAATGTCGGTGGATTGGAGTCCCAGCACCAGGAGCAAATGTTTGAAGCCAGACTTCAACCTCTTGCATGCCATGGTGTGTTGCCCCAGCAATAGTTATATGTGACACACCCGGCCGTCCAAAACTGTCTTCATACATCTCATTTATTTGACTTACAACCGGAAGTTTTGAGTGTGAGCAGTCGAGAGGTGGGCGGCCGCTCATGCTCCGAACAATGAAGCTCAGCAGAAGCCATAGCAGAACCCCTCCCATCGAGAGAGAGAGAGAGAGA |
| **PIN-FORMED 3 (PIN3):**  TTTTTTTAAGTAAAGGTGTTGATCTGCATTTAATGTGGATGCTTGTACATTCAGAAGCATATCATATACAATGAACAATTATGTTTAGATGTCCATGGTTAGTGGCTAACACACACCAAATGCCCCATCATAAAGGATGACTTCAATTTCCCCGACGGGGTAAAGACGTGCTACATGACTCCCAAGGAGCGCAAACTGTCATCCTATTAAGAGTAATTGGTGCAAGCCAAGAGCCACATCATATAATGTTGTCAACACTCTTGAGTTAGTACTTGCTAGCCTCCATTTGCACAAAATCACCCCTACGCAATAACCGGCCGTCTTTACCTTCAAACCCATGGTTGTTTTCAACTGTGACTATTTGGGCCCTTGACAAGCATGGTTGCTTCCAATTGTACCTATTTTTCGTATGAGGCCGAATGTAAGCCCCGCCGCTCCGCTGACACTATCCAAAATTCCTAAGTAGTGGAAGCAAGACCAAGGGTGGGCTCTTTTCCCCAAAAATTAGCTCAGTTGGTTGAACCTAACTTTACATAATCTGATTTTACTACTGATGTTTTTTACTAAAGTCCCAACAGGACATAGTAAATTAGTGTTGTTGGTAACGCAACGAGCATGCCAAATATGACTGCTGTGCTGAGTATATCAGGGTGCAAGTCGTACTCTCTCGCAAAAACGAATGGAACGATCCCTTGAGGTAGTGCTGCCTGAACAATGGCTGCATGCAAATCGACACCTCGCAAGCCAACTACAATTGAGGACGCCGCCATGACAGCGGGCCCCGATAAGAATCGAACTGCCATTGCAAAAGCAGCGAGTGTCGTTCCACACGCTATTAGTCGAGGCTGAAGTGCCATGAACAAACCTAAACTAAACATAGCCATCCCAAGGCCAGCATCTGATAGTAAGCTTATTGAGCCCGCAAGCATTTTTGGCATAGCGACATTCCACTTGAATGCAATTAGAGACCAAATTATGCCAAGTAAACTCGAGTATGTGTTTGGATTTTGAAGAAGCTTCCGCCCAACCATTCGAAGAATGAGCTTAGCCAACACAGGGGCTGGAGGCATTTCTTTGGGCTTACCAAAACCTTGCAGCTTCGGAGAATGTGCTCCTGCTGAGGACTTGGATATGCTTTGCCTATGTTTATCTGCACACCCACCTGTCTCCCCAGGAAATTTAGCTCTGCTACCAAAACTGAAGTCCTCACCTACATATGGAGCAGTTGGAGGGACCGGATTAAGTGGCTCATGAAGATCTCCATTTGGCGCTTGCTCGTTGTGAGGTTGCACATGCATTCGCACTTCTTTGTGGTCATGTGCAGGCTTAGTGAGGTCAGTTGCGTTTACATCGTTGCCTCCAAAGACATGCAAGCCCCCTTCCGACATGGGGGAAGCTGTGGAGCTCCACACAAACATGTGAAGCTCCTTGCCATCATCTTCAAGCCTTGGAGAGCCTCCAGGGTCTAGTACCTTCTTGGAGATCTGTGATGCTTTGGGCGAAAAGCTCGTGGACTTCCCTACTGAGTATCCAGGCCAGCTGCCTGTATTGACGTGCCCAGGACGATTCTGTCCTATCCCATACGGTTCGTTTGGCATGCCATTTGCACCATAGTTTTTGGTGCCATAGCCATGGCTCAGGGTAGAAAAGCGAAGCGGATGCATGCTTCTTGAACCTGGGTGTGCATCCTTATTGCTGTCCTCATGCACATTTGATGCCCTCGGGGTCGGAGCCTGCGAGGACTGCCCAGAATGAACATCAGAAGCCGTGAAAGAGTGCCGCGGACTCACTGGCCTTGTTGACATGACTGAATGCAAATCTGGATTGTTCATTCTATACTCTCGAGGAGTATTGTTTCGAGATGAGTAGACAGAGTACACCTCTACTCCAGTCAAGTTTGAAGGCCTTGGCGTAATGGCCTTTGATGAGGGCATTGATGTCATTCCTATAGACCTTCGAGGGGATGCAAACATGTTCAAAGAAGGCGGGCGGGACGAAGTAGACTTGCGTATAGTCACCCTTATCTTTCCATCCTCCCCTATGTCAGCTTGTGTTTGAAGAGGCTCCCGGCCATCTAGAGAAACTACATCCGACTCCACTTTAAACTCGACAATAGAAGCTCCAACACCGGGAAACTGTTCCATGACTAGATTCGTGGCAGCCCTGTACTCAAACAAAAAAAGTAGGAGAGTGTACCATATGATGCATTGAAGCACAACAAGCTGCACCATAAGACCTCCATAGAACATCCCGTACATGGCTTCCAGTAGGGGAATTCCCATGACTAGAGTGTTTGGGAGAGTAGCGACGGCAAAAAGGGTGATCATCCAATCCAAGCTGCCATTCGGCGAGAACTTGACCCATGCAGCTAGCACAATCAAAACAGCTAACTTCGAAAGCGTGTCTGCTAGAATGAAGGGGCCATCCATCTTGTAAGGATTATTTGTGGAGATGAAATGAAAGGATAGCAAGGGCACAGCAAACACAGAGACAAAGCGGTTGATACCCGAACATTGAACAGGCGTAAAGATTTTCCACCATCTCACTGACCCATATGCTAAAATCATCGCCACGTACAGGGGCACCATCGCACAAAGCACTGCGTAAAGGTCTTTGAGGGAAATCATGTTTGAATGCTCGAAAATTGTGGGCCAAATTTCAGATTTTACAGTGGGGGGGATCAAATCACATATATATATGGATGTCAGACATCGATGAGGAGCCCATAAGGTATGCAGAGATGCTCTTCTTTAGAGCTTATACATGTGTGTAAACGAAAATTACAACAAAGAGGCTGCATCAGCAAGCTAACGCTAAACATGCTGAAATGTAAAGCATGAAAGCATGAAAAAAAAACTGACAAAAATGCTATAAATACTTGTTAGGGAGATGTCCAAAGCTCACATTCAGGGAACTCTTGCACCCAGAAAATGCAAGTAAAACAATTCAAACGATAGAAAATTTAAGCTACCTCACTCAAAAAATAGGCTACCAGTGGCTCAAATCCACCAATAATCTGTGCAGCCAAGCGCAGACAGCGACAAATTCCCTACGCGTGAAGAGACGCAGCACAAACATGGAAACACTCGAAAAGCTTGTTAGCTTAAGCTACACTGCAGAAAAAAATCAGCTGGCCAAATGCTTATTTATCCCGCGAGCAAAAAAATCTGGGAGCTTCGAGCTCTTGTGCAGAAAATAAGAGCATCCTGAGGAGCTACAAGGGCTGAAATCGGTGGAGAACCATTTTCTTCTTACCCTAGAATTGGGCAAAGCAGCCCCAATTGGGAAAGAGGAGGAGGGGAGGAAAGGGGCAGACGGGAGAGAAAAATGAAGAGTATTTGCCCAATGCAGAAAGGGAGGTTGAGGGTCTTTGAAGGAAAGGGAGAAGAAGAGGGAGAAGAAAAGAGGGGCATGCCCATGCCAATGCCATAGCAGCTGTGGTTGCGTGTAGGGGGGCGCCATGTGCAAGGAGGCAGGTTAAAAGTAGAAGCAAAAAAGAGGCGGGGCTAGAAAGGGAGAGTGTGCAGAGTACCTTCGATGCTAGCTCTACCTTAGCTTGCAACTCACAAACACACACACACACACAGCTAACCTGTTACCCAATGGTGGGTTTGAAGTAAAACCCAAGCAAGGAGTAGCTGTGAAGCAAGCAAGGAGGATGACGATGAAGCAGGAGGACGAAAGGGGGTTCATCGCAATCGCTCGTAGAATTAGCCTGCTGCCGCGCCGTGTATTTTATACACGCGAGAGAGTGTCGATGGACCCACAA |
| **ETHYLENE INSENSITIVE 2 (EIN2):**  CACACACACTCACTCACACACACACAAGTACCACACTCACGCACGCACCTACACACGTACTCACACGCAGGTGGGTGGCTTTTTGGAGACACACATGAAGAGATTTACTGGATATGACAGCAGAGAACAAGAAGAAGAGATTATGAAGGAGACTTTGCAAAAGCGGTGACCAGGAGGTTTGCAAATTGCCATGGAAGCTTTGTGGGCACCTGTGAACTCTTTCTGTCTTATCTTAGAAAGGAGGATTTCATGCTCTGTCACTTTGAAGATGCGCCTGCAGGCCATGGGTTTTGGATAAATTCAGCTCTCTTCTGCATTGGCTACCATCTCACGAAACCCTAACCCTAATTCTAGCCCTAGTACAAGCATAGCTAGATAAGCTTTTTTGGGTCCTTTGAAGAATATCTTTCTGGGTCCTTGAACAAATTGGGTCCTCTGAACAAATGTAACAAGGCAGGGAACTTTAATTTGGAATAAAAAAGCCTACTCCTGACCTAAAAGTAGTTTCCCCCACCCCCCCCTCCCTGCTGCTCATTCAATGGCTTGGAGTACCTCTATATCATGCTTGGGTCCTGCTTTTTTAGTGTATGTGGGATGTATGGACCCTGGCAAATGGGCTACTGCTTTAGAGGGCGGCTCTCGGTTCGGATTGGAGCTTGTGTGGGTGATGATTGCTGCCTGTGTTTTTGCTGCTTTTTTACAGTCCCTTTCATCCCGGCTCGGCCTCGCAACTGGACGGAACCTTGCCCAGATTTGCAGTGATGAGTATCCAAGATACCTATCTGCGCTATTGTGGTTTCAGTGTGAAATTTCAGTTCTGATTTTGGACCTTACCATGGTGTTGGGAATTGCGATGACTTTGAATGCGCTGCTTGGCATTTCAATGGTTGCTAGTGTGGTTGTTACAGCAGTTGATGCGATAGTTTTTCTTCTGATTCTTCCTCATGTGGGACTATACAAGGCAGAGATTTTTACTGCAAGCATCATGGGTGTTGTTCTTTTATGTTTTAGTGTGGAGGCTTTATACAGCAATGCAGCCCTCACACCTGGCATTTTCCAAGGATTGATCCCGAAGTTGAAGGGAGACATGCTGTACATAGCTGCTGGCATTTTTGGTGCAAACATTATACCTTGCAATTTTTATCTTCATTCAGCACTCGTGCAAGATGAGAAGCATACAGTGAGCTATGAGCCTGGTGTGTTACTGCATCATAGCATTGTAGATATTTTGACTGGCTCAGGTGTTACCCTGCTCATCAATGTAGCAGTGCTAAGCGCAGCAGCAGCAGCGTTTCACAGCTCTGGTCATGTGGTTATTACCCTTCAAGATGCCCAAGCACTCATGGAACAGATATTAAACAGTTCAGTGGCACCTGCAGCTTTCGGATTTTCCCTGCTTTGTGCTGGTCAGCTTTCAAGTTTTAGTCTGACAAAAGCAGGGCAAGTGGCAACAGAAGGATTACTGGGTTTTAAGTTCCCTCTTTCTTTTCATAGAGTTTTTACCAGACTGGCCGCGGCATTGACAGCTACTTGTCTGCTATGGAGTCTCGGGAGTGAAGGGACATATCAAGCCCTAATTTTTTCTCAGGTTATCCTTTCATTGCAGCTTCCATTTGCCATAGTTCCCCTTCTTAAGGCCACCTCGTCTCAGGCTGTCATGGGCAGTTTGAAAAACTCCTTGCTTGTGGAGTCGCTTTTATGGGTCTGCACAGGATTCTTATATGTAATGAATGTTTGGGTGGCTTTTGACATGTTCTTTGGTGAAAGCGAAGAGTACGTGGGATCAGGAAACTGGAATTTGATAAGGGACTTGGTTGATTCGAGCACAAGCTGGAGAGAAGTGATCCGTATCCTCTGTCTCATTACTGTGGTTGGTGTGACAACAATATCTACATTGCTTATTATGCGGATGATTTCTGCACCATTCAAAGTACAGGACACAAGGCTTCTAGGTGGACCTGTAAGCAGTTCTGTGGAGTCTACTGTCTCAGGACATGCAGAGGGTTCTTACAAAAGTGACACATTAGAAGAGAAACTGACTCTACCTGCATATAGTCCCCTAGATAACTACTCCTTCACCGAAGAAATCGATACTAAATATCATGAGGCTGTTAGGGATAGCGAGTTTTTACTGCCTTGTGAGCTCATCAATGAGCTGCTACCGGAAGGGGGTGCAATTTCTTGTGCCAAAAGTCTAGAGGTGGAACAAGGTGAAATACTTGATGCATCTGTGGCTCAGCAGGTAGTAACGGAAAAGGAGACTCACATGGAAGAAATGCTAGAAGCTACTACAACATTCTCAACAGTGCAGGAAAGCAAGGAGCACTGTCTGGAGCCCCTGCAAAAGGATCCGGATCAACAAAAGCAACCCCAGTCAACTACATCTACTGATAATTCCTCCTTTGTTGAATCGTCCTCAGTAAACTCTTTGTCTCGCGATAGTTTTTTGTCATCATGGGCAGTTTCTCCTCCTGAGTTAACAACAGCAGAGACAAACAATGCACCTGCCACAAAAGTTTCTGGAGGTGGTGAAGCTGAAAATTTGAAAAGGGTTATTGAAGATGTTGATCTTGAGCTACTTGACAAGGATGATTATGATATTGACGGATGGGAAACCTTAGATCAAGATGATGCTCTTCAGGATTCCATATCAGTTTTAGGTGGCAGTGTCAATTCATTAACATTTGAAGAGCCAGAATCGGGGAGAAGCTTTAGTAGGAGTGATGCAAGCGAGGGCAGCTGTGGTGGTAGTGGGAGTGGCAGCTTGTCAAGGTTGTCAGGGTTAGGCAGATCAGCACGGAGGCAATTTGCTGCCTTGCTTGATGAGTTCTGGAAAAAGCTGTATGATTTGCATGGACAACCAGTTTCCCAAACGCATGGAAAATCACGAGGGGCTAGCACCAAGATCTTACAGTCTGATACAGCAGATCACCAGCCAGTATCAGCTTACAAGGAGGCATTTGGTCCAAATTCCACCTGGTATACTTCTAAGCTTGCACAGAAATCTTGGCAGAAGAATGTGAAAGGAGATATGGCAGGTCAAACTGATGCATATCTTTACCCTCCTTATCTAAGCTCCACATCAGACTCTTTTGGTGGCAATGACCCCTTTCGTCAAAAGCATTCTTCACTTGATGTGATTGAAAAGCGATATTCAAGCTTGAGATATCCATCATTTCGGGATGGGCTTGATAACCAGCCTGCTACCATTCATGGGTACAAAATGGCTTCATATGCAGGAAGGAATGGAAGCTTCCCAACAGGTGTGGACACTAGCTCTTTTCTCATAGAAAGGCAGCGTCAGTTGATGCAGCGGTCTTCAGATGAGCAGTCTTTTTCTGCCAGTTCCTTATATGGGCTTCCAGCATCGGCTTGCTCCGAGCTAGATACCAGCATGTCGACCAGTTCTATTCAATCATTCCTGTCATCACCGTACGCGAAGCAGTCATTAGGTAGACAGATTGATAGCTTTTCCCTTCCAAGCAAATCTTATCCCATTCAAGCAGACCGGCCTGACCCATACTCAAGATCTCAATGGACTCCATATACAGGGAGAGATTCTGCAACTTGGGATCCTTTAGTATATAGAGCTACTGGAGAGTTAGACATTCCTCACTATGATCAGATAGCTCTGCAAAGAGACAAAGACTCTTTGAACTCCTCTTTTGGACATCGAACTGGTGAAAGAAATTACTTTGGCACTGGTTCAAACAATAGCAATCATTTTAAGCCTCAGATTGATAGAGGTCCACTACCGTTTGACGAAATTTCTCCATCTCCGTCCCACAAAGATGCCTTTTCTATTCAATCAGCCCCTCAGAACCAATCCCTGTGGGCCACTCAACCATTTGAACAGCTTTTTGGGTCCGTTCGCTCCCCAATAGGGAGTGGCAGGGCAGCCACACGGAGTGCTACAAACAGTTCTTTTGGCCAAAATGGAAGCCATGCATCTGTCAAAAATTTGCAGACAACATTTGCAGGAAATGATCATGAGATTGAGGTCTTGAACATGCTACGCTCGTGTATACGCAAATTGCTGAAGCTTGATGGCTCAGAATGGCTTTTTCGACTTGAGAGTGGCTCAGATGAAGATTTGATTGGTACAGTTGCAACTAGAGAAAAGGTTTTACTCGACGCAGATGCCAGGGAGCTACACAAGCTTCACACAGGCAATGAGCAGATTTGGGGTTCCAGCTCTCAAAGATCAGTTGCAAGTAGTAATAATGACTTGGCCTTCTTAACACTTGCTACCTCTGGTGTACCACATTGTGGTGAGGATTGTGTATGGAATGCAGAACTTTTGATAAGCTTTGGTGTGTGGTGCATTCATAGAGTTTTGGAACTGGCACTGATGGAAAGTAGACCGGAACTTTGGGGAAAATACACTTATGTGCTAAATCGTCTTCAGGGTGTCTTGGATCCGGCATTTTCAAAGCCGCGAATTGTGTCAACGTCATGTATATGTGTGATATCCGAGGCTTCCGATGGGCAAGGAAGAGGTTTAAGTAAGCAAGGAAGTCTCAAGGACGGTGGGATAGGAGACATGCTAATAAGCAGGTCTCTTTCTGGTGGTTTTCCAGGGAATTCTTATCAAGGGTATCCACAGTCTTGGCCTTGGGGACGCAACTCGAGCGCAGGCAAAGGGAAAGGGGCCAGCTCATCAATTTTCTTGGAAATCATTAAGGATGTAGAAACAGCAATAGGTACTCGCAAGGGCAGAACTGGAACTGCTGCAGGTGATGTAGCATTTCCCAAAGGTAAAGAGAACCTAGCTTCTGTTTTGAAGCGTTACAAACGTCGTCTCGGAAACAAAGGCCCTGGAACACCAGCAAATGGCAGCAGTAACAATTCTGGAATTCGAAGGGCACCAGCATCAAGCCCAAATATCTTCATGTAGCATCTGACACCAGTCTCGGGCTATCCATCCCACAGACATCTAATACGCTTGCTGACAACCCTCGCTTTAGCTTGATCATGTCATTATGGGCGTCGTGAAAACTCAACAGAGTTAGAGCAGTTCATGTGTGTCATTATATGGGTGTTTTAAAGGAATGGAATTATAGCAATTCATGTGTGTTCCACCTCTTCATGGATGTTAAGCCCATACAGTTGGGCTTGGTCATACAGGAGGACTCGGTGCAAAAAGGGCACCAGCTTGTTGGCGATGAAGATACACAGCGTTCTCTATCCTCAAACCGAGGATAGTAGTAATTGTTCATATTACCAAAGGAAGCTTGGGGATAATTGGGGCTAGTCTCGATACGCATGCTTTGAAACATCAACGATGGATCACACCAGGGGCTAAAGCTTCAATATGTCAGAACCACTTCGGCCTAAAGAATAAGGCATGAGCAGTGGCATGTTTGACGCAAGTGGGCAGTGCTGTGTTTGTGAGCCAAAATTTTTCTGGATTTCGCTTAGCTGCATCGGTGAAGAAAATTTATCCTGA |
| **ETHYLENE OVERPRODUCER 1 (ETO1):**  TCAAAAGAAAAAAGTCGAATAGATGGGTCAGATAACATGTGATTGTTTTGCTCAATTTTCTTACATGAATGCTAGATTCCAGCAGCTTGAAAAGAAACAACTGTTTGGATGCCACATTCAACATTAAGATCATGTATAGCTAATTCAACAAGGGCCGAGGAGCATGTTTCCTGAGTGTTTCATCGCTTGACCACAAAGACAACATTGTAACAATGCCACCTGGAGATATGGAGGTGGACACGGGAAGATTTTGACAACCTTGATGATTGCAAGACCCTCCCAAATTACATTTCATTACAGAGAAGCAATCTTGATTTCTCCCCCTTAGTGCTGTTAGTTATAGTTACAACATCACGTCAAACTAGCATACAGATGATGGTACACCAGGATTTACTCAAAAGTGGACCACTAATGATCCTTAATCACAGCTTTTGAAGAATTGGCTGAGGAATACAAATTGTCCTCATATGCAATCACAAAGCTGACAGATAAAATCAAGGCTCCCTGCTAGAGACGCGGTTATGAAGCTGCATTGTTTCCACATGGCTTGGATCCACTGACAAAGCTGCTCGACAATCACGTAAAGCACTGCTTGCATCTCCAATGCACTCGTGAAAGGCTGCCCGCAAATGAAGTAAATGCAAGTCAGCTTTAAAGGCTATAGCTTTTGACAATTCATCAATGGCTTCCTTCTCTTTATGACTGTCCATTAGAACTGCAGCCCTGTATCTGTAAGGGTATGTTCGCAAGGGATCCAACTGGGTGACCATGTTCAGATCTGCCAAAGATTTTGTGCGTTCACAGTATTCAGAGCGTTTTTCGAAGGCAGACGCACTACTTTTTGCCTTCTCAATCAGCTTTGTCATCTCTTCATAGGCAGAAGGTCGGTCATTCTTCAAATAATACACTCGAGCAAGGCCCTGGTGTGCCCGTGTATGACGAATCTCAAGAGCACTAAGATAAGAATCAGCTGCAAGGTCTAGCTTGTCACAGTCGACATAAACACTACCTAAGTTATTCAAAGCCTGCCCCTTGCGAAGCCCATCTGATGGGCATTTTAGTGCTTCCTTCAGAAGATCTACCACCTCTTTAGACACATCAGCGTCCCGATTAGTATCTGCTAAAGCATAAGCCTTCAAGAAGAAGGCTTCAAATGAGCGTTGTATATCTATAGACTTCTGTGCTTTGACAATCGCTTCCTTGCAATGCCCTGTATCATAAAGAATCCATCCCTCATATACTAGCCGTTCATGCTCTGTGGGAGCATTCTCTCGAGCAAGTTGAAGACTCTGCATTGCAGACTTCGGACAGTTCAGCCGCAAGAGTAGGAGAGACTGTCTGAAAAAGAGAAGAGCTTTTGAAGGGTCTGACTCAAGCATTTGATGAACAACAGCAAGTGAACCGATGTCATCTACAGAAGACCATCGGTCATACAATTGCATCCAGCAATCTGCAACATTCCACTGCTCCACATGAGGGGATAAAAGATCTAGCAACTTGGAGGCCCTTACCCTACCTGAGTACAGAGTATACTTTGGGTTGAGGGTGAGTAATGCACGGACATCCCTTACAGCCCCTGCATAGTCCTGAGCTGCCAGACAAAAATATACTCTCAATTCGAGACAATCAGTGGTGACCTTGAACCCTAGGATGCGATTAATTTCCAATATAGCCTCATCAACTTTAGAATCATCCATAAGCCTTGCCGCTCTATACTTGTAGGGGTAAGTTAATGTTGGATCAAGTTCTGTTGCCTTGTTCAAGTCATCCATCTTCTCTTCTTCATTACAGTACAGAGACCTCTCAACATACATCCACCCAGTAGTAGGCTTTTGACTGCACAATTTCGTGCTAACTGTGGCAAGAGCAGCATGTCTATGTGCCAGCTCATATTTGGCACGCGCAAGACCAGCCAGAGAGTACCGGTGACCCTCTTCAACAGCTGCTTCAAACAGTTGATGGGCATCCGACAAGGATTTCCTTTCCAGCATTGCACACCCGAGTTGATGCAATGCCAATGCTTTCTGTCTGGAAGTTGTCGCCGATTCTCGTACGTGCTCAAGAAACATGATGCACAAGTCCGATCCTGCATCATCTTCCATAGCTACTTGGCTCAGCAAACTGTAGAAAGCAAATGCGGAATGCGTCCTCATCATAAACATCCTCATATCAGCTCTGCTTAACAAGGATGCCACTTGTGGATTTTGCAGTATGCCTGGAAGACCTTGCAAAAGAACCTCCAAGCATGACGCAACTAGTAATGGTGCACTCTCCTCCAAGCCATACTCCATCAGCATAACTGCATCCTGCCTAGTATGGACTAAATTTGCAAGTGCGCTGTCACAAGCCTCTTTGATGTGCTCGCAACAGAACCTGTTAGCAAATGTAAGCACTTCCAATAGTCCATCAGGCTCCAGGGGAGGCAAGCTACCTGTTTTACTAAACTCGTCGACAGATCGCATACTTTCCACTGAAACCCCACTTTGTGAAAAGTCTATCCGCTTCAATTTAGACTCTGTGAAGCATCCATTTAGCATAGTATCAAAAGGGACAGACAGGGAAGCAATCCTTTGGCGCCCACATATTACTTCTTCACTACCTATGGAAAATGTGATGATTTCATCCAAACTGCCAACACTCAAGGTATCCGCATCCGCGCAATCACTACTATCCCTGCTCAATTCTGAATCTGCAGTACACCCACCCCCATCCGAGTGAGCAGTTCCTGCATGCTTGTCTAATATGGACGACCTGCTACTCAGGTCCTCCCCCTTTTTCTCCAACTGCAACCAAGCTTTGTACACGAGCTTTGCATGAGGCGTACTTGCAAGATTAATGGCTGCTCCCAGACTTCGACGAACCAGTTTATTCTCACCAAGCCCTTTGAAAATGAACGCCTGCAATAAACATAAACTGGCCTTATCGTGTTCTGATGCATTCTCTATCTCCTCGTGCAATTGTGCTAGCGTTTCCACATAGTCTAGAGGCTTGAAATGTGATTCGACTGGGGGATCCGCAAGCTTGGACGCCAAAGAGCTGCGCGGTCGTTCTGACATGCTGTCCCACTGCTTTTTGCATTTCCGTCGCTCCAATTGTACCCAAGAATCTGGTGATTTATCCAAGGGAGATAGCGCATGAACCTGGCTGCCCTTACATGAATCTGCCAGCCAAAGACCTCTCATCTGGCTGAAGAAGGCCAAAGGAAGCTACTTTCACAAAGTTCCAAGAGATTTGCAATTCCGAAACACGTTATCCTCTGCTTCCATGACTTCTGCATCTCTGTGTGTGGATTCAACAAATTGAAGAGAAATCGGCCTCTGATTCATGGCCGCAAGCTCCCTCAATCAACCATGGAGATACATTGAGGGTTTGACGCAGGAGCGAGAGGGTTGCTTGCGACTGCAAGAAGCTTATGCACAGATAGACGAGTGGAAAGACAATGAGCCACTCTCCCTCTCTCTCTAGAACAGGTGGGCAGAGAACGCATTTCCTCTCATCACTCATAAACTCTCTCTCTCTCGCTCATGGGGGGTGGGGTACGCTTCGATGTGTGTTAACCTTTTTAGTCCGTCTGGTAAAG |
| **REVERSION-TO-ETHYLENE SENSITIVITY1 (RTE1):**  AAAGCCCAGACATTCTTTCACTAGGGTTTAAATTGCTCCTGCTTTTGTCTCCAAAGGCCTTGAGGCTTGACCTTTTGTAATGTTGATCCTACTCATGTCGTGCATATGTACCTGCTCAGCTTACATGAGGAATATCATGTGGCATTTATGACCGACCTCGAAGAGGCCGTGCCGACCTTCCCGTTAATCAATGGGCATAGCTGCATAGATGATGTAAAATTTGAGGAGGAGGAGATGTCATTAAAAACGAAAGCAGGGAATGCCGTGGTTGTTGAGGCCGTGCAAAAAGGCGATTGTGTCCGTGTGGGAAGCAAAGCCGCCAAATTAGAGATTGTGCAACCAACAAGGGGTAGAAGCAGCAAGGTCAGCCATCACTCCAATTCTTAGGAAAGCTTTATTGACGAGCTGGAGCCACAAGTTGCAAGACCAAGGGCAAGCCGAGTCATGGCTGAGCTCCATGATGAGGAGGCTTTGATGCATTCAAGTGAATCTTCCAAGCCAGGCATATCAATAGACCCTTCCTGCTCTAGATTCCCCCATTGTATAGTTTGGACTCCATTGCCAATAGTGTCCTGGTTAGCTCCATTTGTTGGCCATGTGGGTGTTTGTCGTGAAGATGGTATAATATTGGATTTTGCGGGCTCTTATCTCGTGAATGTTGACAATCTTGCATTTGGTCCGACAGCAAGATATGCTCACCTCGACGGGCAACAGTGCTGTTTTCCTCCTCATCTGTATGGTCACACATGCGAATCCAGATTTGAGCATGCAGAACTCGGCACAGCAACCTCGTGGGATGATGGACTCCGCAGTTGTATGCAGACTTTCCAGCATAAATGCTTTAACCTGTTTACTTGTAATTGCCACTGCTTTGTCGCCAGTTTTCTGAACAGGATTGCGTACCAAAAGTCAATCAAGTGGAACATGATCGACATTGTATCATTGGTCTTGTTGAGAGGCCAGTGGATAAGCAAATGGTCTGTAGCAAGGTCTTTGGTTCCCTTCACAACAGTGATGTGCCTTGGCTTATTGGTAGCTGGCTGGCCCTTTTTTGCTGGTTGGGCTGTCTTTGACTTTCTCCTCATCGCTTGGTTTTTGTTTGGTACTTACATTAAAAGAGAACTCATTGAGTCCTAGTTTTAGCTTGAAAGGTCATGTACTTTCCAGATTCAAGTGATAGAAGAGCTTTTCTAGGTTCTCTCTCTTACAGTGTACTTGTATGGATGTATACATGTACTGTGTATGTTTATGTTTG |
| **STEROL 1 (STE1):**  CAAGTGATAAAATTTAACTGAATTATCAAACCAAATGTCCTTGGTGATTTTTTTTTCATACAACTCTCTGAACAAGCTGGAAAACATTGAGTAAAATAAACTTGAAGTACTTCAAAGACACTATTAGTCAGCAAGTAAAGTAACACCTTTCGCATCAGTAAAAAATCTTATCTAACATTACAACGCTGTTTCCAGCTAAAATAATTTCACTACAAGCTGCTCGCATGACTGTTTCATCCATCTAGGAGGCCTTCGACTTTTCAGGTTGAAAAGGGTCTCGCAGGGTTCCAAACATCCAATCCATCCAGATTGTGTAGTGTCCATAATTATGTCGGTATGTGGTATGATGGATTGTATGATAACCCGCACCCATTATTGGCCAAACTTTTCCATGAATGCAGTCATGGATATTTGTTGTCCATATGCTTTCCACAAACAATGAAAGTTCATGAGTAAAAAAATGTGTGGGCACAAGAAATATGAAGATGACATGGGGGGATGCCTGTAGTATTCCATCCAATGGGTTGAACGCTAAACCTGCAAATGGTGACAATGTGTTCTCCTTATTGTAGATGTGGTGCGTTGCATGCAACCATTTGTAGAGCGGTTTCACATCGTGTAAACCCCGATGCATCCAGTAAATCCCAAATTCCACAAGAGCAAGGTACAAGAAGGTCAACAATATGTACCTCGGAACTCCAACATCACCTATGTTAAGATAGCATCTCGTCCACCCTCTTTCAATCATGTACTCAGATAGTGTGGGTAAGGAAACATAAATAGGCATGCCTTTCATGCTCACCCAAATCTGTAACAAAATTGGCTCTTTGGACGGTACACCACCTTTGGGAAAATAGACATGTCGTTTCAAATAATAGATGTAGAGGCACCAGAGACCACCGGTGGTGAAGTAAATCAAAACACCTGCAAAATAATTGCGGAGCCATGTTTGCGCAGTATGTGGAATTTTGGATCTAAATTGCGGAGATATAAGGAAACCCAGAACCAGGTCGTTGTACCAAGATGTCTCTTCCACAAAGTATTGTAAGAAGGGGCTTGATGTGGGCATTGAAGAGGATGTCTCTTCAGTAGCATTCACTGCGACGGAGGGGAACGCAAGGGCGACATGGCAAGCTGTGAAGAGGATAAATAAGGCAACAAAATGCAAGTGTGCCATACCCCAAAAGCTTATGCAGCAAGAGCCGTTGTGTGGAGGAGGCACTCTGCAATAACCATAGCCAAAGAGAAGCGCGCCCAAGAACAAAGGGAGCCTTCAACAATCTGTGTGAGGGGTAAAAAGAGCGATTTTGATGTGTGCGCGTGTTGAAAGAGAGAGAAGATGCGAT |
| **GPCR-TYPE G PROTEIN 1 (GTG1):**  AAATTGCCAGAAAGACCAAGACGGACTAATTTGAGCATTTTAAGCCATGATAAAAGATGTGTGTTTGTAGTATTGGTCCTCTTTTAAAGACCAATAAGAAACTACGAACGATTAAATAGAGATAAGATTAGTACAACCCAAGCTCCTCAATCTGAATGATGTTTACAATTCCTAAACACAACTTCATATTGATACATGTCAACTGCACCTTCGATCAATAAGACAGTTGAACAACCCTATCTAAAGGCTTACAGGCACTCACAACTTGCACCTTATTTTCTTTTGATATACATCCAATTCAAAGGGTACTTTATGACAAGTTAAAATTTACATTCGTGATTCTTGAACAGCTTATAGAAAACTACAATGCATCACAGATGATGCTCTTTCACGATCAAAAACGGGATGAAGGTTCCATACAACAAACGCCAATGATACGATACTGATGTGGATGAGCTTGAGATTTGGACAGATGGAAGGGGTATCCATGGATGTATTTTGCAGCTAAACACTAGTACATAACGACTAGTACATAACGACCTGCTCCTTGGAACTTTCTGTACTTTTGAGACAAGTAATTTGCTTCTGCTATTTTATATTACCATTAGTTCATATTCAATAGGATGCTTGAGATTTGAAGGGACAAGAGGGTTGTCCATGATCAACAGTATATGCCAACCTGCTCCTTGAAGCTTTCTGTACAATTAATTTGCATCTTGTGTTTTATATAGCCAAAGGTTCATCAGGCTTCATTATTCAATAGGATGCTTGTCAGACTGACGGGAAGTATACTGCACCGCGATAACTAACAATGAGACAAATGAGCTTGCCACAAAAATAGCATCAAACCACCTATGATAGAAGTTGAACTGTATATCTCCACCTAAAACATCTGTGATGATAAGCCTATACTCATTCGCAAGGCTCTTCCTTATTAGCAGGATTGAGGAGATGAAGTACATCCCCATTACCTCCGACAAAAGCAACACAACATTGGTTGATGAACCACTCCCACCTCCCGATAACACATAGAAGGACTTCATGAGATTCTGGAGAAAGCCTCTGACAGACATTGTGATCAGCATTCCAATGAAAACAAGCGATACATACTGAGACCAAAGAGCAACATTTATGCCAATATGGAAAAATTTCAAGAAGAAGCTTAGTGTCTGCGTAACTGGATCTACTGAATTTGACTCCTTGAATATTACACTTTGCATTGACTTCAACATCTTATAGATGCAATAAATAGAGAAGACGTAGCCGACTAGATTCTTCATGTGTCCTCTCCAGGTTCTTGAATAAGCTGCTGCTTCCTTTGCCTGCCGCAATTCATAAACTTCCAAAAACAACTGCCTAGAGAGCTCTTCCAATCCATTAATTTCTGCTTCCATGCTCTTGATATCTTGTTCCCGTTCATCTTCTTGGACGGTTCGCACCACCGTGCCAATCAATCTCTTGAAAATGGATCTTGCCTCTAGATGCTTTTGCGTACCTTGCAAGCGCTCCATTTCCATGCGAGAGAGAATTATTTTCTTTTTCTTTGCAATGCATATGTCCATGGCTTGCAAAAGTCGCCTTTCAAGGGCAACAACATCCACCTCTTCTATTTCCCTGATAAACAATGACAAATAGCTATATGGAAGATTAACAGCTCCAAATCCAGATAGTATTGCCATGACTGTCACACCAATAACTCCAACTCTGCTAAGCAACTGCGGCATTGTGAAAAAACCTTTGTCTGGCGATGGCATTGGGAAATGGATACCCATTCGCCAAAATGCGTAAAGTAAGGCGATCAAAAAAACAACAGCTCCAGCAGCTGCTCGTTGAAATCCCAATCCTGTGTTACGAAGCGAAAGATAACAGTGGTAGTATGGTAGAACAAAGACGAGCAGTGTGATCAAACAAAAGAGATCCATCTTCCAGTTCATCCACCTTGCACTTTTGGAAAGAAGAGGAATGATTTCAAATAAGACGAGCTGGAGAAGGTTGAAGGAGAAAGCGAAGACAATGCTGAAGAGGATCTGCACAAGAGCGCTTCTCTCCTCGTACTCCTTGTAGAGCTTCCTGTTGAGGAACCACAGGCCGCTCCATGCCAACCCTGCTAGCGATCCTGCAATCACCAACCCCTCCAACGCCACGTACGCCATCGCCATCCTCAGACCCTCATGTGAGAGAGAGAGAGAGAGAG |
| **JASMONATE-ZIM-DOMAIN PROTEIN 4 (JAZ4):**  CTGTACGTACGTAGCCCAAAAGCTTAAAGGGCCTTCAGGAATCACATTCTCGGAGCCTCTCAGATTCTCTCCCTCTTTCCACAGCGCTTAAGCTGCTATCCTTTTGGATCCCTAGCTCTCTGCACAGCATTTGGGCTTTCTTCAGCGCCTGCTCTAGCTCTCATTTGGGTTTTCTTCATCAGCTCTAGCTTTCATTTTCAGGCCTCACTTTTTTGGCGAAGTCTCCATCCTTTCACTGCATGAATTTTGTCTCTCTTGCATTGCCCCCTGCACAATTAGGGTTTTCTTTACAAAACCTGCAGAGTGTTCGCTTTGCTGCTTCCAAGCTGATGAGATGGGCCGATCAGATCAAGAGAATGCCGGGGAAAAAGATTTCATGCGGTTTGGCAGCGACACTGCAAGCTGCGCCGAGGCAAGAAAACATATGTATGCAAGCAGCCACGTAGTACGCTCGGAAAGTAAGCACATGTGCATGAACAACCACATGTATAATGTGGATCCTCATGGGAGCAGCCAGCATTATGCAGACGCTATTGCCGTTGTGAAGTCCAATATTCTGGATGCTGTTCAACGGCCTACCCCTTCCCGGTGGTTGTACAACAACAACCTCACAAACAGCCTGGCAAATGGGAGCTTGGCCTTTGCTGACATGGTGCGCTCTGATTGGAGTAATACTGGTGGCAGGCAACAGTTTTTTTCTTTAAATAGCTCACGAGAGCAAAAAGTAGTGGAGAAGGTCAATGATGCCTACCAAATCATGCATGCCAGGGGCCCTGACAATGCTGTGTTGAATAGCACTGCCTTGAATTGCATTTTGCCAGCCAACTCCTTACCAGGGTTGGGAATCAAGGGGTTCCCTTTTTTAGCTCCGCACCAAATTCCAACGGCAAGTGGTCAAGCACTTACTCCTGCTTTGTTAGTGGGTGGGGGTCCCTCTACAAGAATGCTACCAGCTGACTCTAGCATCAAACCAAGCAGCACACAGCTCACTATATTTTATGCAGGCAAAGTTAATGTCTACAACGATGTGTCTTCAATCAAGGCCCAAGAGATAATGCTGATGGCGGCGGCTGGTAATTCTTTGCCATTAAACATAGATATGTGTTGTGGGACATCTATTAGAATGGTAACACCACCTGTCCCAGGGTCTCTCAAAAATGCAGACCCTCCGGAATTGTGTCAGATAGACCCTTCTACGTGCCAGTCTGATCAGTTGGCAAGGGATCAGGTTGGTATAGGCCCCTTACCTGCCCCTATACTTCAGGCATCTCAAGCAGAAGAAAGTACTCAAAGCACATATCTCGACGATGAGCCTGCCTTTCCACGAGCTTTACCTCATGCAAGAAAGGCATCCTTAGCCCGATTCCTTGAGAGGCGAAAAGAAAGGGTGCTGTCAAAATCTCCTTATCCGGTGGAAAAAACTTACCTTTGAGGAAGTGACCTTAATGAAATCAATTGTCCTTCCCATATCATTGTGGGGATTTATATAGCTCGTGTACAGCAAGCGATTGCAGGAGCATTGTTTTTTAACCACATCATCATGTGGATTTATAGAGCTCTCAGTTCTTGTACATCAAGAGATTGCAGGAGCCATTATTTTTTCCTCTTTTCATTATTTGTCAGTAGACGGGGCTAGTTCTACATAATATGCAGCTCACCCCGAACTGACTTCTATGCATGTTTTATCACAAATCTTAAACAAATATAAAGCCGATGTAGAAAGGCTCCATCCGTTGAGGCCTTACTTTATGTGCTAAGTGATTGTTGATGCGTGACATTGTTATGATGTGGAGTAACAATTTGACTCTTTTGTTTTGCAAATTGATGCCAAG |
| **DE-ETIOLATED 1 (DET1):**  CACAAACTCGTCGAAATTAAGCTGTTAGGCACTGCTCCCTAAAGACTTTTTCCACCACCACCACCAACAAATCTGGCAGTCTAAAGCTTATCCGACTTAACAATTTGTTAATCATGGATCCAAATTACTTCTTACATAAGGCTACTCTAAATCTGTCCTAGTTTACAAATCAAACATTTTGTGTAGTGTTTCATAGGTATACCCCCATGTGCACATGGGTAAAAAAGGGCGGTAACAAACCGAAGGATAAAAACCATTGCTATCTTCGCATGTGAAAATTTACAACAGCGGGCTGCATATATGCTTGCTGGACAGTTATGGCAAAAGGAAATATAGGATGAAAAATAAATGAAGCTACTTTCTTCGCACGCCCATCACCAGTTCCAAGCTCAAGGCCTGGATTAATTTTAAATTTGAGGACATTTGGCTTGCGCCTGGAAATAAACTTTATAGGGTGCTCTGTGCAAGGCTTGTATTGATCGGTTGCAGAAATAAGCTTTTCATCAAAGTGGAACAAAGACTGATCAAAGTATGGTGAAGGACTGAGTGATTGTGCATTGCATGGAAGAGAGGCAAGGGTTCTCTTAATAACCTGGGCAAAGCTATTTGGCTTTGTGTTATTGCAAGCTGACTTCTGCTTCCTGAATTGCTCTCTTGCAAACTTGTTATTTGAGCAGGAAGAGATAAATTGCATGTACAAAGGATAAGATGGTGCAACTCGAAAATGGTCAAAGTATTGCTCCAACAGCAACAAAAACTCTTCAGAAGAGTTCGGAAAAAACCCAAGTATTTTGGTTGATTCAAAGTTGTAAATGACAAAGAAAGCTATTTGATTGGATGCATCTGAGTTTTGTAAAACCACGCCATCCACACTACCAAACTTGATGAGCAAGTGGTACCTGTCCAAAAATTGTACCTTCCACATCACCAAGTCTGCATAATGTTGGAAATGGAAGTAAAATCTTTTGAGATGTTGAGCTTTCGCAACAGGATCAGAATCTTGACTCTCAACGCTTCGGAAAATGTAAGACAGCATCCGTTGCTTCAGGCCACCCAGCATGTGATCACTTTGCATATTGGCACCCATGTCATCAAAAGAGTGAGTTTGTGAAGTGGAGGTCTCTTGACTTGGTCCTGCCTGGGAAGTCATCTCATGATTTGAACCTGTTGAACTTGACCTCGTATTCTCACCGAAAATTGTGTTCTTGTGCCCATCAATATGAGTATCACCACAGTTGCTTCTGCTTGAAGGACCTCGTAATGCCCTGGAACACCCTTGTGCATGGGAGCTAATTATCAGTTCATCATCTTCTCTGCAGAATGGTCCAATAGTTTGAACATCAACAAACATCCCTGCCCTTCGCACTTGCAGTATGTGAATGGACTGATAGCGCACAGATAGAATAGAAAGGAAGTCGTCATACATGAATATGCCAGCATTATGGGCCAAGTGGATGAAATCATCGTAGAACTTCCTTTCATCCATAATCGTACCATCTGCTAGCCTCACAAGAGACAGCGTGATTTTCTTCATGGAAGGGATACCAGGAATAGCTCCAGGCCTTGCAGGAGCATCAGACTCGAGAGCAGTTGCAGTGGCAAATATACCATAACAATTGCAATCTGTTGCCAGGAAAAAATCTTTACATATGAGCTCGTTTCCAGATGCAAGACTTAAAGAATACAACTGGGAGAAATGGCTCTCAAACTTGTTTCCCTTGATGGGGAATTCTTCCTCTATATCACAGTTTACTCCTTTACTGCAGTACGTCAAGCAAATGTGTCGATAAACAACCAGGTTCTGATAATTCCGACTGAAACAGATCAAAAACTGTCCATCATTTGAAAATTTTCGGAAATGATAATCTGGGCACTCCACGTCATACACTGTATAATTTGGAACAATGTTTTCATACAGCACTCTTGCTCTGTGAATGTGAGTATTGGGTGCAGCAGCAGAGATCTGGCGGTCGAAAAGCCGGTGCAAAATGTTCTCGCTTTTCCTCCACATCCTTATACTGTCGTATCTCGTGGCCACAGCTGCTTCTCTGTGACAACACGCTTGTGGACGCCTTCGTCCATTGTTAAACTGCTGAACTTCGAGTGACTCCTAGTTCTCCATAAAACTTATGTAC |
| **CULLIN 4 (CUL4):**  TTTTTACGAAATTTGGATCTAATTTAGCACAAAAATCTTCAGAAAGTAGTCTGGAAGTCCAACTTGCTAAGAAATATGGAAGTAACAGGTGATACAAGCTTTGGAGTCATGCCTTGACTACACTGAAAGAAATCACCGTACACATGGAAGACATTTTCTAGATGATGACGCATTGTGCTTCTGAAGGAGCTTTAGAATTTACACTTTTCAATTCCCTTGCACTTTGCTGATGATTGTGGTTTTCTAGAAGGCTGGCCAAAGATGGATTAATAGATCTCTTTGGCTGTTCCACGCGGTTGGCTAAAAAGCTGTACAATATACAATAGTGCAATGGAGTACTGAAGTTCGTAAAATGCTATGCTCCAGCAAGTCATCATGCTGATTGGTATCATTTACAATTAGAAACACTGCTAAGAAATAAAAGAGTATCTAGATCCCTGTTGAGACATCACATAAATACTTGTTATGTATGAACCCACATTTAGGTATAAAAAGGTCAAAAAAGCTAAGGGCAATTTGTCAGCATCAGAATACTGAGATCATACAAGAGAAAGTGCATTTACAACTCATGGAGCAAATTTCCTTGCAAAGCATTGGCATATACGACGCGTTTGCTCTCTGAGGATAAACATCTGATAATCTGTTATGCCTTGCCAGTAGAAACCTTATAATCCAATGTTCTCCACAATTGAGACTAACCATGATATAGTCAACGGCCTGGTCCCAACAAGCTGTAAGTCAAGCAAGGTAATTGTAGATTTGGGGATTGCTTTTGTCCCTCTCAAGATACTCTCTGTCGATCAAACTTTCTATTCTTTTCTTCAGATCAGCTGGCTTTATGGGAAATTTGAGCTGTTGGAAGAGCTCAGTTATTAACAGTGTATGGCTAAGAATCTTTCTTGTCTTCATGATCCTTACAATAGCAGCATCTATCTGATATTGCCTATCCTGAAAGACTCTTTCAGTTGTGCTGGTATTTTCCTCCACAGTCTCCTTTAACTGAATTGCGTTGACTTTGATCCGGAAGAGAGGGGCAACAAAGTCCACATTGAAAGAAAAGCAGTCATCATCTTCAACATCTCTTCCTTTGGGTTCCTTAGAGAGCACTCTCACTTTTCCACAAGCCAATGACTGAAGAGTTCTACGGAGCTCTTTGTCCTCGATGGCAGTGGTATCCTTGATTTCTTGAAAGGTGAGCCTGTCTGAACTGTTGAAAAGCATCAGCACAACCGTCTGAAATAAGGAAACAGAAAGCTCCTTTCTGCCTTTTGGATAATCGGCTTTCAAAACACAGTGGCCAAGTGAGTTTTGCCACATCAACCTCCGGCCACTATGCTTGCTGAGGTAGAAATCCTTGAAAATGTCTTGATAAACATTCAGCTCATGAGGTAGCCGGACTTCCATAGGCGGGTAAGTAGGCCAATACCCTGTTGTCAATACATGCACACTCATTTCAATGCCTGAAGGCAGCTTCGTTCTGGCCTGTGAAGACTGTTTAAAAGACTCGTTAATCTCCTTAGAAAGCTCTATGTCCTTAAACATGCCTTCCAGTTTATTCGTAAATTGGCTTCCACATTCTGTTTTGAGCTTTGAAATCATTGATTTCTCTGCGTCGATTGATGCACTTTTTCCCAGAAGAAGTCTCTTTGCAAGGTCCTTTTTATAGAAAGCTTCAAAAACATCCTTTCCCTGTATAAACCGAAACAAAATCAGCACTCTGTCCAGAGTGTTTTCCAGCTCCTCTTCAGATGTGCCTTTGTTGCCGGCCCTCAGCTTACCATCTATAAATTTTGCAATTAACTCTGCTGGGCGATTCTGCCGCAAGTTGATAAGGTGCTCAAAAGCATCCTTTATAGCATTTGCAAATAGCTCATTTTCAAAAAAGCTTTTTTCCCACACCACATCAAGTCGAGCTTTGAAGTCCAAAAGCCACAATACCATGTCTTTGTCTTTCTCATCATCCATAACAACATTGTGTCCAGAGGCCTTGATGTAGACACTAAGTGACTGCTTCAGCTGTTCCAATGCGCCCACTTTTGAGAACAAACTGTACATCCTCTGAAGGTCTTTGACACGATTCGCATCCATCAGCATGCTGAAACCCTTGTCTAAAACGGCTGAAGTGTGTCTCTCAAGCAGCTGCTTTTCTGCAGCTGCAACCAAGGGCTTGCGGGTGCTTGCATCCAAGTAAAGAAGGCAGCGATCATGCTCTTCATGTAACCTGACCTCAACATGCTTGAGGTAATCAGGCACATCAGTTTGCTGCATAAATCTAGTGCCTTCTGCCCCATAGAAGTCTGTCGTACAATTAAGGAAAGGCTTTTCAAAACTTTCCACATAAACACCTAAAGCACTGAACATGCGAAGCAGGTGTTTCAGAAGAGTTCGGTCCACTGTCTCTCCCATCCGTTCTTTTTCTATCAACCTCAATAAACCAGTTACAGTTTTATGTTCTACTTCTGGACATGATCCCAAGTGTTTGCGGAATAGCTGCAACCCCATATCCCATAAAGACCTTGCATTTGAGGTCTGTATGACATATGTTCGGTCCAAATACAGCGCTATGCTACGAATCATCAACATCTGGTCACAGTGTTCCTGCCAGCACCTCTCCACCAGTGACAGGAAGACTACTGCATCTGGGCTCTGGCCTACCAGAGACTGCAATTTGGCTGCAATATGCATCTCACATTCTTCTTGTAGACGACTGTAAAGATTGCCTGCCATTTTGTGCAAGCATAAGTCCTCCACAGCCCGATACAATTCCTCAAGGCTGCAGGAAACTGGTTGCTTTAAATGGATAGCAGTGACAGCCTCCTTTATTTTGGCCCAAGTAAGCTCCTCAAAGTTGGTGGGAAGTTTTGGCTTGTCTTTGAAGGGCTTGATGACTAATCGCCTGGGCTGAGGAGGGGTAGCTTTCTTCCGGGACAAGTTTGCCGCAGTACCGGCAGCGATATGGGATCCAGGGAGGCCCTCATCCTTGATATCCATGAGCATATCATCAGGGCCGTGGTCACCTGTGGAGTTGAAGAATCCCAAGCCGTTGGATCTAGGGTCCAGGTTCTCCTGTTTTGGCTTCTTGCTACTAGAGATCATGCCGATGCCCTCACTGCTCCCGTTCACACAGCTGCTACTTGTTTGGCTTGCCGGCCGCTTTGAATGCGACATCAACCGCTCATGCATTGCACCAACACCTGGCCCTAGACTGCATACCCACCGCCGCTCGCTGTTGCATCTCTCTCTTTCTCTCTCTGAGGCGAGCGTACCCTTCTCTGTAAGCGTACGCTCAGTTTAACCCATGGAATTCCGACACCTTAGCAGAATCCAGTAAACTATGTCTGGTAAGGCAAATTGATGGAAACAATAACACAGCTCGACGATGGTGAGACCAAGCTCGCTGCTAAGAAAAACTCAGCAGCAATCCCTCTGTATGTTAAAATG |
| **ELONGATED HYPOCOTYL 5 (HY5):**  TCTCTCTCTCTCTCTCTCTCACATCACTGTTCAGATACGTATAGCAGAAAAGCCCTCGCCTTTGTCAAGGTTTTGCACAGCTTGAACTGTAAGTTTCCATGGCGGCTTCGGAGCTTCGTCATTCCTCTTCTGTCTCGTAGTTTAGGGTTAACTTGTTTCTTTGTCTTCTTCTTTTCTTTTTACGTCACATGATCCGGCAATGTCGGTTAAGAGCTTCGGTTTCCTGTCTCTGGAGTAAAGCTATGGCGAAATTCGAGCCGTCGATAGATCTTTTTTTCTTTAACTCCGTCGTTTGCTGAATCAGTCGTGTTTCGGATATATTGTATTGGTTGCTCTGCTCTGGAGCCGTAGCGTCTTCTGCGTGCACATCCTTGCTCATTTTCTGCAAATCTCCATCGCCGAATTTTCCTGTTTCTCCAGGCTAGCACAAGCGTTCTTTGAAGCAGGCTGTTTCAAATATGTAAGCAGAGCTGCTAGTGATGGTGGACTCAAGCTCTTGGTTTAGGACCATGGCGGATTGAAAGCTCTCACAGGAATAGGTCTCAGGGGTAGTAGAGAGAGTGCAGTGGCCTGTGCAGCTTTTGGAATACGAGAATGCAGGCTTCAACGTCAGCCATGAGCTCCCCCCATGGTAGTGAGGAAAAGCCGGCTCCCTTCGAAATCATGGCAGCTTCCATGGATCAGCTCAGCAAAGACGGTATGCATGCCTTTTATTCTGTAGAATCGATTCTCTGTATGAGAGAGATCTTCTCCTTAGAGTGTGTGTGAAGAGAAAGTGTGTGTGTACACGTATTGTCTCCTGTGTTGTGAAAGATGTGCAATTTTGGCGTTTTCAGCGTAACTTCTTGTTCGGACACGCTCTGTGCGTAAGTAGTTGTTGTTGTGTGAATATTGTCTCTACTCTCTGCATTGTGAGCGTGCACGTGAATGCACACATGTTTGTGTGGTCCACTGTGTGTGCTTTGTGTTCGATTGTGTGTAGTGCATACCTCTTTGTAAAAATCTCAGCGTGAGTGCATCTGTGTATTGCGAATGCTTTTTTGCGTTTGGATTATCACAACCCGTGCAGTCTTTTCCAGTGAGAAGTTATCTTTTGTTTTAGTCATTTCAGTTGTCTGGTGCTGACAAACATGAATCTACACCAATTTGCCAAGGTATTATCTTCCCTTCTCCCGCCAGTTCGTCTCATTGTGATTTGGGAAATATGTGCTGCAGATATCTTTTGAATGTGTGTGTACACGGGCATTTGTCTTTGTTTGCATCAATGGGTACACAGAACCCACTCTGCATATGGGGACGGATGTCTGAGCAAGCATTGGTGTGCACAATTATTGAGTTCACATGTCTAACACAAAGTTAAGTGCTGGTAATGATGTGTATGAACAAGTTAGTGAAGTAATGGGGTAGAAGCTAGTCTTGGAAGCCCAAGTCAAGTGATGGTTTTGATGTTTAAAGTGTGTATACATACAGGTCTAATTCACGTGGTAAACCCTTTTCATAATGTGCTTTGTTGTGTCAGGCGATGATAGTGATTCGGATGTGAGGAAGGTGCCAGAGATTGTTGGGAGGGCTTCAGCATCAGTAGGTGGATCTGGGAAGCATCAAGGAGGCAGGAAGAGGAATGCTTTACCTGCCGAGAAAGAGCACAAGAGGATGAAGAGATTACTGAGGAATAGAGTGTCTGCACAACAAGCAAGAGAGAGGAAGAAAGCCTACATGAGTGACTTGGAAACCAGAACAAAGGAGCTTGAGCAGAGAAATGCTGAGCTGGAGGAGAAGGTGTCTACTTTGCAACAAGAGAACTCCATGCTACGGAAGATAATCAAGAGCACGGCTATCAGAACTGGCTCGGCCGGTGACAAATGAGGAAACAAACAATTTTAACTTAGGACATACTGTTTTATTTAGAGTCCAGTTATACCCTCGTCAAAGACCATAATGTGGCTTATAGCCCATTCCCTCAATGGATAATTGCCTCTTATTTTACCTTTTTTAGGGTGTGATCCCTTAATCTTCAGGCCTTTTTCCAAGGGCCCTAGGACCAAAACATAGTTTCATGGAATATCGGCTTTCCATCCTTACACTTATTTTTGAGTTTCTGGGAAAGCAGACTGGCTAGGCATGTTTCATGGAAAATATTGGCTTACACTTCTTTTGAGTTTCTGGGAAAGCATACTGGCTAGAGATGTGGATAAACTATTTGCACATCCTTGTAAATGTAGTTTCTCAAAATTGCACAAAGCTTATTGCTTCTTTCCTTCTCTGCTTTGGATAAATAGTTTTCTCTCCTTTGTCTTGGGA |
| **XAP5 CIRCADIAN TIMEKEEPER (XCT):**  GGAGGAGCAGAGTGGGCGAGGGCAAGATGTCGGGCATGGGCGATGGATATGTAGGCACAGCGCAGGATGCAGCTCGCATCCGGCGGCTGGAGAAGCAGCGCGAGGTGGAGCGCAAGAGGATCCAGGAGCTCAAGAACAAGTCCTCTGGTGGCCAGACTGGCCTCCTTCAGTTTGGCTCCGGCACCTCTGAGCTTCTAGAGACTGCTTTCAAAAAAGAAACTGTGGGACTCGTCACAAGGGAGCAGTATGTTGAGAAGCGTGTTAACTTGCGGAGCAAAATCGAGGAGGAGGAGAAAGAAAAGCAACAAAAGCTTGCGCAAGAGTGTACAAGCTGCAATTCAATATTTGTAAGTTTGTATGTATTCAATGGCATCTTTTGTGTTTGCACAAGCACAAGCTTACATACACACATGATTATCTTTTGATGAATGGCCTGCTGGACGTACGCCTTGATGGATTTTTGCATATAATAGATGCACCTGAATGCACAGAAAGATACCCATATAGGTAAACATAACTTATGTGCAGGTTGATGCGTAATTTATGTGTGTGGCGAGTGTGCGGCATGCATAGAATGTTTTGTGATGTTGTTGGAGATTACACTCATTTTTATCTTTCAGAAAATGAGGAACCCAAAAGGGGAAAACTCGGTAAGTTTGGTAAGGATCCAGCTGTTGAGACTAGCTTTTTGCCAGACAGGGAGCGCGAAGCAGAAGAGCAAGCTGAGCGTGAACGATTGCGGAAACAATGGCAGAGGGAGCAAGAGCGCATCAAAAATGAGCCACTAGAAATTACTTACAGTTATTGGGATGGAGCTGGTCATCGGCGTGTAATTCAGGTTCGTAAGGGAGATGCAATTGCAGAGTTCCTCCGGGCGGTCCAACAGCAACTAGCCTCAGAGTTCCGGGAAATCAGAACGGCTTCAGTGGAAAATTTATTATATATCAAGGAAGATTTAATAATTCCACATCAACATTCGTTTTATGAGCTGATAATCAATAAAGCAAGGGGCAAGAGTGGGCCGCTCTTTCATTTTGATGTGCATGAAGACATCAGAACAATTGCGGATGCGACTATAGAGAAAGATGAGTCACATGCAGGGAAGGTTGTAGAAAGGCATTGGTACGAGAAGAACAAACATATTTTTCCAGCATCCAGATGGGAGATTTATGATCCCACTAAGAAATGGGAGCGTTACACAATACACGGTGACTGAGGCACAATCAAAGATTAGAAGCACACAAATCAGCAAGATGTTTTAGGGATGACCTCTTTCTAAACTTGTTATGTCAGAGATTTAACATCGTGTTACATGAGAACCTCAGCATTCTAACAAGAAATTTTGTGATGTATGTGGAAACATTTGGGATCATGAATTATTGGCGGTACATCTTGTACAGTTTTAGAAGTAATTTTGTTTTGATTAAAAAAAA |
| **SPLICEOSOMAL TIMEKEEPER LOCUS1 (STIPL1):**  GACACACTCGATATTTGAATATCGCATCACTTCAATGCGATTAAAAAACGATTATAATACTACAGCGTGATATTTGAGTAAGCCTTTGAATCCTACCAAGTGAACAGGTTTTACATCCCACCCGGTTTCCAGTAATAAGTTGCCGACATGTCTAAAATTGCAGAAGCACAGCATCAATACTACAATAACCTCAATTAAGGAACAGTGCAATCCATTACCTACAAAACTATGAGTGGGATTCAACATTTCTCTGCTCAACCTTAGAAGAGATACAAAAGAACACAAGCATCGTCCGTCGCACTTCCAAGGCAGAACCCCCCAAAAACCAAATAAAACAATCAGAACAACAAACCTTTTTAAACCTGCCCTGATTTTGAGCTAATATACCCAAAAATACACATGAAGATGGATCAAAACGCTTCTCTGTTCACCACATTCGATAAGGACTCCGAACACACTCCAAAGTATCCTTTGTTGCTTGTTCAAGAAACGAAACAGCAGTAACATAAGAAAAGGTAACAAAGACAGTCTGAGCATGTCAATTCATAATTGAATTTGACATATGACATTGTATTGCATTCTTTGCAGACCACATCTCAGCATAAATAGGACCACTGTTCCTTGATAATGTACAAGCTTGGCAGCCTACTTCCATCGGCTAGACTGAGCCCGATGCATTTCTAGTAACTGTTCCAGTGAAACTGCCAACCATCTGTCCCCTGATTGCGCATACAACCTTTGCTGTGCATTGTCCATGCAAACATTTGTAGATCCAAAACCATAGACTTGAAAACCTTCATGTAAGCGCCCAATTTTGGGCAGAAATTGCACATCGTTCTGCTCGGCAAATGCCTCTACAACCTCTTTCAGACTCATTTCCACCGAGCCATTATCGCGTGCAACATTACCAAAGACGGAAGAATGCTGCTGCGTGTATGCAGCTGCGGCTGCTGCTTGCTGTTGATTCTCAAACTGTCTTTTTTCTGTGACACGTAGATAGCTTACATTCTCCCTTGCCCCCGGTTGTACAACAGGCATACCCTCCACAGCTTGATCCATCATGTCAAGAGCACCTTGCAGATGCCACCTCACCCTTTCATTTGCCAGCAATTCAGGAGTAAGTAGGCTTTTCCAACCCAGATACCATTGTGTTACTTCTTCAAAATCGGGTGTTGAGCAAAGCCAATTATAAAGGACTTGGCGCCACTTCATAAAGAAACCTGCTTCCAATAAGGTAACCATATGATGTATGGGAACTGCCGCACCCCACGCCATCACCCAGCGAAAAGGCTCAAGCTGCTGTGCTTGAGGGTTTATGATAAGTTCTTGCAAGGCAACCATGAGTTTGGGAAGAATAGACTTCACTAACAGAAGCTCCCAGCTGCCTGGATCAAAAACAGTTTGCCAAGGGGATAATAATGCGTACGCTGAACTATCACTGGCATGCCACGCTCGTAATGCACTCTCCAGCTTGTAGCGAATAGGATGGTAAAGAGGCTCCATTCGTTGCCCCAGTAAAGGCAACCATGGATGAAGCCATGCATGAATAGGAACTGTTTCTTGCCGAGGATCCCATTGATCCACTGCTAGGCTAAGCTTTGGCATTATTAAATGCTCTAGAATGCTGTGAAGCACAGATTTTGGAAGCAAATTCTCCCATATCTCCAGAAATCTTAACATGGGTTCAGGATCACGAGGTTCCCAAATGTTAACCGCTGCAAACCGAAGAGCAGGAAACACAGCCTCCATTACCAACTGGGCATATGGCGAATCCCCATAGCTGTTTGCATCTGGAAAGATTGAATAATCTATGGGATCGTCTCCTTGAAGCAAGCATTTCCAGAGACCTAATATGTCTGTCCCATGAAGAGGTTGAAGCAATGGTTCCCAGCCATGAAACACTTTTACCATTAAAGGGAGCCCGAATGATAAAGCTATGGCTGAAAGATTATGCAGCTTGTATTCTTCTGCATGATCTCTCTGCAAGGCGCTGAACGCACCTGCAACTGCATCTAAATTCATACCTCCACTCGCAACCTTTTGCTGCACATCCCCAATTGCCCTCGTAATAAGTTCCAGGGTACCTATCTGCTTCTTTTGCCTATCAGCTTCACCTTGTAGCCGCACCCGCTCCTTTTCAAGAAGCACCGCTGTGTCTTTCTCCTGCCTTAACTTCTTATCAAACGTCTGTATATCAGCCTCCACAAGATCCACAATCAATCTAACATTATGCTGAAGCTCTGGCATGGGGATATCATCTTCAATATCTGTTTGCTCAGCATTCAAATGCTCGAGATTCGTGAGAACCCGGACCTGCGGCCCTCTCATATCCAAAATGGTTTGTACAGGCTCAATACCCTGCTCACTCTTCAGAACAAGCAAATCATCTGCTGTTTGGAAATCCTTCTTCTTGCCTTTAAATTTTTTGAGCCAGAGTTTCTCTTTAGGCTTAGTTTCCTTGTTTTTAGCTTCTGCTTCTTCAGGTCCTGGTAGAGCAGAGACCGCCGCATGCACCTCTCTGTAGTCATTGAAGCCCATACCCATGTTCTTGGGTCGCAATTTTGCTTCAATTGGGACAGCTATACCCTGCTCATTTTTACCAAGCCCCCCACCTTTGTAGCCCATTTTTTCAAGAAGCCTCATACCTATACCTTTTGTGTGCTTCTCAAACTCAGGCTTATCAGCAGCTGCCGTACTTGCAGAGAGACCCCCCTTGCTCTTCGACGTGGTCGAAAGCCTCACCTTTTCTTTCTCTCTTTCTTTCTCACGCCTCTCTGCGCCTTCCTGGATGCGCTTCCCAAAAGCTGAAGGAAAAAGGGTCTCTTCTTCATCTGCTTGCTCGGGCACAGAACCTGCAAACCCTAATCCAGCAGATCCCAAGCCCGCGTTGGGAGGTTGATCTGCAACATCCTCTTCTACTTTATCTATTTCCTCAGAGGGTTTAACAGTACCGGTAGAGACAAAACTCACAAACTTGGAAAGGTCGCCTTTCTTGATGGTGTCGCCACGGCGCCTGCGTTTGGAGGAGGATTCATCAGCATCAGAGTCGCTTTCGTCGAAGACACCATATAGAGCTTCCTCACGGGTCTGCGAGCGCATCTCCCTGCGCTTCTTGTTGTAGTAGAATTCGCCCCCGATCCATTGCCCACCCGTGTAGTCATTCTCCATGTTGAAACGCTCCATGTGCTGGTACTCATCCATGGCTACGGCACTCTGTGTGCAGCTTTGAGAAAGCTTCTGTGCAGCGTCGGTTTACGTGATTGCTATGTCGGCTGGTTATGGCGAGTGAGCTATCGACTGTGGTGGCATAAAATTCTCTCGCGAGGGAGATGGACAGAATCAAGGTT |
| **ELF4-LIKE 4 (ELF4-L4):**  AACCAATCCAAGTTTACTCAAATCCATTCTGCAAATACATGATGGAAACATTTTTTCAGGTAAAAGAAGGGTCCCACTGTGCAAAACACGGTGCACACGGCCCAAAGACTTGCAAGAGAAACAAGAGAAGATCAGCTGAAGCTCCACAAGCATTCACTCCTCCTCACATGTTTTACTGTATTTATCACATGCATTTGAGGATGAAAACATGACCGCTTTTGGGAGACGCTGTTGCTGTTGCGCACAGATGCAAAGCAAGTGGCATTTTCCTCACTGGACGACAATGATGAAAAGTATGGGATTTGCCTCCCTCTCTCAGTCTCCCTCTAGAGCTGCAAAATGAAGCGCCAGATCCTTGACCTTGCTCAAGAGACACCAAAATTGAGCACATGTAACTGGTCAAGTAAGCCGGGGCTTCTTCTGCGCCTCATGCGAATGACTGTTAAAACCGCCTCTATCCATCTTCACCATGCGATTTGCTTCCCCTTTTCCTGCAGATTCCTCCATTATTGATTGTACAAACTCTGTTGATAGGTTTGCATAGACCTTCACAACACGGGCAATATTAGCATTGAGCTCCCTTATAAGTGAGACATTCCGTGCGAGGTTCTCCGGTACTTTACTTTCTTGATTCTGGTTGATCTCTATGATAAGGATGCGATTCTGATCAAGGATTGTTTGCACCTGGGAGAAGCTCTTCTGAAACATCTCCCAAACCTTGCAATCCATGTTATTCCCATCTTTCGAACATTCTGTATTATTGCCCCCATTCATCAGGGCGCAAAAACAGAAAGCTTCGATAAGTGATATGTATCTGCCTTTGATGAGTGGAAGTTTTCCAAAAAGCAGCTAAGCGCACATTAGCTGCAGCTACCGAGAAGAGGAATGTATGGACTTCATTTGGACGTGTCGTAACAAAAAGCTTTAACAAGCTGTTTGATGTGTTTAAGCTTCACAACTGTGGCTAAACCGACGAACATGTGCGTTGTATGTGTAATTCGCATGTGTGGGGATTACAGAAATTAGCTTTATTACTCTTTGAGGCTTGTCAAAATTGTTGTGTGTCCGCGCACAATCGACAAAAGAGTGTCAAGAGCTATTGTGCAGATACTACTGCCTGTGAGAACTCCCAGGGGCCAAAGTGGGGTACTGCGATGGCAAGAAGCGCTGTCGGTGTAACAGAGAAAACTAAAGAGAGAGGG |
| **MORE AXILLARY BRANCHES 2 (MAX2):**  GAGTGTGTGTATGAGAGACACTATTTGCAGCTATTCATTGAACTTAAACCGATGAACCCAGATCAACAATTGGGCTTCCTGACCATTTCTTACAGCAAGCTCTTCAACAATTGGCATACACCAGCAAATCAACAATTGGGCTTCCCTTCTCCAAGCTCTGTAGAACATGACCATTGCTATTCTGCCTGGAATTGTCCAGGTCAGCGCTCACTCATAGATTGTGGGAGCAAGAGGAAGAACTACAAGGCAGGAAGTCTAAGAGCTTGAAAAGACACTAAATGGTAGCATTTTGCCACAAAGGGTAGGAATGGAGCCTCCGGCTCTTGAAGTCCCTCCTTCTCCCCCCCGATGGTCTCGCATTCCTTTTGACATATTTGAAAATGTGCAAGGAAGAGAGAAGAGGGTTCGAGTTGCGGCACCAATGTCAACGACGTCCATCAAAGATATTCCTGAATCAATCTTGGCCATCATATTTTCTAGCATCTCAGAGACTCGCAGCAGAAACTCTTTTTCTCTGGTTTGCATGGAGTGGTACAGCTTGGAGAGGGCAACAAGGACAAAACTTTGTCTCAGGGGCTGTGTAAGTGATTTGTATCTCATTCCCAGATCATTTCAAGCAGTTGAGTGCCTTGATCTCTCGCATTGCTCTCCGTGGGGATACTCCTTGTTTCAATCCATCCCAGAAAGAGCTGTCCTAGTTGGACAGGTCTTAAAGCAAGCTTTTCCAAGAGTGAAAGACATGACGATTTATGTTCGGGATGTGAGAGATATTGATATGGTGGCCTGTTTTTGGCCAGATTTGCAGTCTTTGAAGCTTGTTAGGTGGCATCAGAGGCCCACTCAACCAGAAGAGGCCAGTGGCCTTGGCATGGAGTTTTGCTTCTTAATGCAGTCATGCAGATTCCTTAAAAACCTTGACTTGTCTGATTTCTACTGCTGGACTGAGGACATACCTCCTGCTTTGATGGTGGAGCCATCTGTGTCAAGCAAGCTTCACAGCCTCAATCTTCTTAAAGTCTCAGTAGAGGGTTTCAAGGCCTCTGAATTGGAAGCCATTTCAACTGCTTGCACAAGCTTGGAGGAACTACGAATAGTCCTTGTGTTTGATCCGCGCTTTTTGGATTTTGTTGATGATGCTGTACTTCTGCACCTTGGAGCTAGCTGTAAGAAATTGAGAGTGCTACATCTCATAGACAATGCCGCATTCAATGATGCGCGTGGTGATCCGGAGGATGCCTTTGATGAGCAGGATGCACAGATTGGAAGGCAGGGCCTTGAAGGTGTTTTTCAAGCTCTTCCTTTGCTTGAAGATCTTGCCATTGTGCTTTCACAAAATGTGAGGGATGCTAGACCCATTTTGGAGGTACTTGCTGGTTCTTGTAAGAGGTTGACATCCCTATGCCTTGGCCATTTTCATGGTTTGTGTAGTGGGCCGCAGCCTAATGGGGTTGCCATGTGTCATGGGCTCACAAACTTAGTCATCAAGAGCTGTGCAGATCTTGACGACATTGGTCTGGTTGCAATAGGATCTGGTTGCACAGGGCTACGAAAACTAGGCCTTAACTCTTGCAGAGAAATATCCAGAGCTGGGTTGCGCAGCTTAACAAGACAACTTAGTCACAGTCTTGTGGATGTTCAGGTTATTTGCTGCTTACAAATGGATGCAGTGGACGCTCTTTGGGCCTTGCAACCAATCCAAGGCACCATTGTCAACTTGCATGTCGACTTTCTGCTAAATCCAGATTTTGTGGCAAGGGAGGCAGAAGATGCTAAAAAAAAGCAGCCATTACCTCCTTCCTCCAGTTGTTTAAGTCTCAAGAAGACATGTAATGAAAGTGTAGGATCTTCTGGCACAAGGAGCAACAGTTCACAGCTGAGCAGCTCTGGCAGAGATCGTATCAGTGATGGACTTCAAAAGAGTCATTCTATTGAGAGTTTGGATCTCAATGTTGCCGTAGTTGGCCAAGAAAGTGACACAGGATCCAACTCTCTTTGGCCCCTAGCAGACGGGTATGGATTTCCTACACAGAGTAGCAGTAGCTCAGGGGGGCTTGCAAGCGATTGCCTGACTGCAGCCGAGCAGGACACCAGGGGTTCCACTTCCTTGTGTGATGCCAGTGTGATTACTGATGACCTCTCAAGCCACTATTCAAAACGCCTCCCAGGACTTGAAGTAAGCTCTGAGTGTGGCGCTTCTGAATTCTCTCTGGCATTTCGTGAAGGAGAATGGAGCAAGCTAAGATCTTTGTCTTTATGGATCCCTATAGGTGAGCTGTTTTCTCCATTACCCATCATGGGTCTGCAGTTTTGTCCATCTTTACAAGAAGCTCGAATCAAGGTTGAAGGTGATTGTCGAGCATGTCCCAAGCCCAAAGTTCGCTTATCTGGTATAAGCACTTTTGCATCGTACCCAGCATTGTCAAAGCTCACGTTAGATTGCAGTGAAATAGTAGGGTATGCTCTCAGTGCTCCCCTAGGTCATATGGAACTCAGTTTATGGGAGAGATGGTATCTGCAGGGTATAGAACACGTGAAGATCTTAGAGCTTGATTACTGGCCGCCTCAGGACAGGGACGCGAATAGGAGAGGGCTCTCTTTACCATCCGCTGGCCTTATATCTCAGTGTCCAACATTGAGGAAGCTTATCCTTCATGGCACAATTAATGAGCATCTGCTGAGGATGTTTCTAAAGGTTCCAAACCTGCGGGATGTGCAGCTGCGGCTTGATTATTATCCAGCTCCTGAGGTGGAATTGAACACAGAGACACGTGCAGATGCTTGTAGAAGGTTTGAAGCGTGTCTCGCTGAACGTGGGTTTCTAGACTAACTATAGGATCTTACAGGAGTCACTAACATCATGACACAACTTATTTTACTAAGGGTAAGGGCCTTCAGCTTACTTCCTTTTCTGATTAATATGATGGCTGTCTGAAGAAAGTTATTGACAACCATTAGGTCTTGCTAGTGAATTTGACTTGGTTGTACAATGTTCATCTTTTGCAGGGACACTTGCAGGTTTATGGTGGGGGGCATCACCATGGACTGTTTCAGCAGATTTTAGGTAGTTTTTTGGATGGGATCTCAAAATATCTTAACGAGTCCCTATTTTGTATTGTGCATGAGATGTACAAATCCACATTTTTGCATCATGGTGGTAACAGCTCTCTTGGAGGTCTAAAGCCATTTTGATGTTGTGACTGTACCAGAGACTAACGAGCTTGGGGTCAACATTTCTGGCTTTTCAAACATGTTCGCAGTATTAGCCATATATA |
| **ACCELERATED CELL DEATH 5 (ACD5):**  AAAAAGGCTGCAGGTGGACTCATTGATAGAAGCAGCTGCACTTGTTGTCTCTGCGGCCTTTTTGTTCAAGAGAGGGCGTCGATCTGCAGAGGCTGGTAACAATGTTACTGCACTATTGCTTACAGATTTTCCATGATTTTGAGGTTTTGGTGGGAGTTTTGCCCTATGACGCCGCAGCAATAGGCCATTTAGGACTTCATTGAAGAGGCCATCACCACCCACAATAATAACTCCATCTAGTCTTGCCAAGTCATCATCACTAGTGCTGTTTATGACCTCAAATGCATGTCTGGTCCTCTGTGTCATTAATACCTTTGTGTTAATTTTTGCCCTTTTGAAGAGAGATGAGACTTCTTTCCAAGTATTTGAAGCATTTTTCTTGCCACTATGTGGATTGATTATTACCATGAGATTTCTGGGTCGTTTGGTGTCTTTGTCTAATAAGGACTGTATTTGATTCATCCATCCCTCGCAAACACCTTGATCAGTATGATAAAAGACAAATGTTTGAGGTATCCATGCTCCCTTCTGGCTGGAAGACTTTTGATAAGCATGCACTACAAAGCGATGCACCTCTTTTAAAATTTCTCCAGAAAATGTCTCTGGTGATTGTCCTCCTACTGTATCAGCAATAATCGGTTCGCTAGATTCAACAGCATATATTTGTGACATTGCCAACTGATTAACCTCATCAATGTTCTTAATGGGGACCCAGCAACATACGGTGTCAATATCCAGCTTATCTTCAAGAGCCTGCCAAGCCAGACAATTGGATGATAGAATAACAGAGACTGGGCCCAGAATGTCCAAATGAAAATTGGCTTCCAGCAGGATATCTTCAGAGTTTGCAGCAAAGTTTGCAGCCCCATCAAAGCGCCTAAACTTTTGAAATTTACCCTGACCATCCCACAAACTAGCACCAAACCTCAATCTCTTTGTACCATCTTCTATGTGCGATGACCCAACCGACACATCCACTTGCTCCCCCATCCAGAAATTTCTCTTTGTGAGAATCTGGACTGCTTGTCTGCTAATGCAAAAGCTGCTGGGAGCAGACACGCGCATGGAAAACCACTTTTACATAAGAGCGAGAGTGCACGCGTGTGTGTGCGCGCGTGTATTGTTTTGGGCGTTTCAACCTTTGAAAATTTCTACATGAACGTTGATTTCGTG |
| **AUTOPHAGY 5 (APG5):**  AAAAAATCTCTCGAACAGCGCAACTCCAAGGTGATCAGCAGAGTCGATGATGCAAATTGTCTCTTATTTGAGCTTTTACTTCAAAACAGTCTTTGCTGCTCTATTCTAAACTACAGGATTGCTGAAATGTGATAAATGAGTTCCATGTAACAATCTGGCGGGACAAAAGATATGGTTATTGAAGAACGCAAGATTGTATGGAAGGGTGGGATACCTTTGCAGCTGCAGCTCCATAACTCAGAGGTTACAACAGTCCCGCCACCACCTCCTTCATTGATACTTGCACCACGTAATGGATATCTACCACTTCTTGTACCAGTTTTGAAGCCACAATTTCAAAGTGCTTTGCCAATTGGACAAGATACAGGCTGGTTTGAGTATGAAGGCCTTCCTTTAAAATGGCATGTTCCTACTGGCGTGCTTTTTGATCTTTTGTGCTTTGAACCAATCAGGCCCTGGAACCTTACGGTGCACTTTAGAGGTTACCCGTCTGAATTGCTCCCTTACGAAGGAGAAGATGTAATGAAATGGAGTTTCATCAATTCCCTAAAAGAGGCATCATATGTGATGTATGGAAGCACAAAGCATGTAATGAATCTATCTCAGCCCGACCAATTGGATCTCTGGCGATCTGTTGTTCAAGGTGACCTTGAAAGTTATGATCGCATCTGTTTCAGATTGGCACCTAGTTTGGGAACGAACTCGAGTGCCGCAAAAAATTTGGGTGATTCAGAAGGTTCCTCTAGTAGACGACAACAAGGTAATCGATCATGTGGAGGGCCAAATCATGGCTGCGGCTTGTATTTGGATGTTCTGAATCAACTTGGGAACTTCTTTGGGCAGCGGCGGAGATGGCGGCTCTCAAAATACCGTTCCGCCTTTATGTAAGAACAATAGAAGTGAGCGATGGGGATTTGCCGGAGGGATCCCCACTAAAGAGCTGGGATGACATTACATACATATCAAGACCAGTAGATGTATGGAGAGAAGATGGTGTAGTACTCACACTTTGGGAAGCCCTGCAAAAAATTACACCACAGCTGTTTGCATATGCATCGGAGCTGCCTAGTAGAGACCAAAGCCTGCCTGCCCCTTTAATCTGGGAAAGTGACTCAATCAGCACTACAGCAAACTCAGAAAATGATTACTCAGGTTATCCAAACATCAAACCAATAAATTCTTCTCGTAGGCGGCCACAAGCTCATTATTTGAACGGAATCATTCGTATACAAGGTATAGAGCCTAGTCCAGATCTGCCTATAGATTGGATAGCTCAGAATCTGAGCGGACCAGATCATTTTGTACATGTTTGTATACTGATAGCAACTAATGAAAACAAAAAGAGTACATACTAAGGAATTAACATACATGAAGCCCATCTTCACTTGGAGAAAGAAGCTTCAAGGTGGAAGAGGCAAAGTTTTGATTGCGGGGTTTAAAGTGCAAGCAAGTATTTAGTACATTTCAGAGTCTGCCTAGAGGGTATGCACAATGGAATTTCTGAAGAATTCCGCATCAATTAAGAAGCTGCATAGAGAATGGGCAAGAGAGGTACGCCTCTAAAATACATTTGCAAGATGCAACATTGGGTTTGCATCTGAGCAAGAACTTAATGTAAAGCTCATAATTAGAAAGCAGCGCACTTTCAATAATAGGTGTTCCTTTTGCTTGTAAACCTTCTTGACCTTGTAACAATGGTAGCTGTGATCAATACAATGAACATGTCTTCTCCAACAAAATGTCATTGCAATTCTACTTTCTGGTAGAAGCGGTGCAAATCGGAGTATCGGCATGGGTTACGACAACTCTGATTTTAGATTTACTACAGTAGCTCCATTGCGCTTAGTAGCTTGCCATTGTTGTAAGTAGCCTAGTAAACAGTTGAATGATAGTCAAGGCTGCAATTATCTTGAAGCTTATAGACCTTTTCTTGGAATTTCAAATCACTCTGAATAAGCCATGTATGAATTGAATCTAGAGAGGTAATGGAAGTAGCAAGCTTTTATCTTAAAA |
| **AUTOPHAGY-RELATED 11 (ATG11):**  GCCAAAACGAGCAGAGGAAGCACGACAGTCCAGAATAGAGTCTCTTAATATCAGAACGCGCTCAGTATCGCAAGATAGAAGCTCAACTCTAATCAAGCGTAAGAGAGGTTTTTTCTGCATGTTCTTTCTTGGTTGCAGTACAATGGGAAATGTAGTACACTAGTTTAAAAGATGAGGTAACATGCTGCACATGTTGGAGAGAGAAGTGGACTTAGTTGAAAATTATGATGTCCTTGTGAAGCTAGTGTGTATAACATCACATCATGGATGGAATTGTAATACACCTTGTTGTCACTTACCCTTTCTCAGTAGTCTTTTTGAGTTTGATTTCTACTAACATCAGAGTTTTTGCGCTGCAGTGAGGATGTGAATACTGTAAAGAAGCTGATTGATGAATGTGTGAGCAATAACCTTGCTTTATCAATGCGACCACATGATGCCGTGTCTGCATTTGGCCCAATGTATCATGTGCATGTAAAT |
| **TAPETUM DETERMINANT 1 (TPD1):**  AGGGGGGGGGGGAGGGGGAGGGGGATAGGCAAATACTGGAGAGGACTAATTGCTGCTAATGGGGCCATGAGTATGTGTTTGTAGTGAAGTGTGAGCTTTGTATCATCTAGGCACCTAGATACAACATCTCAAAAGAACAGAAGGCTAGAACTAGATGCGACATTGGAGAGGCAAATTCGGTGACAAAATTGGACCCAAAAAAAGAACTCGCAGGAAGCAAGCAGTTTTTTGGGAAAGGGGAGGATAAACAGAGTTCGAGGGAGGAAAAGACAAGAAAGATTTCAGCAGAACTTAGCAGATTTAAGCTTCAAGGGGTACATGAAGGAGTTAGCATATTGAAAACGGATGATGTCCCCATATTTGAGGGGCTTTCCATTGTTAACAAGGCAGTCATCAAAGGCAATGCGTCTAAAGAGCGCGGGGTTCACTAAGGGCGCTGAGGCAAACCAGCCACATGAGAGGTGTATTTCTGATGGCGCACATCCTGCTAAGCATGTGTTCACTATCTGAACCACAAACTGTGGGATCCCCTCTGATCCATCGAGCCCCTGCGATATGCTTATATCACTAGCTGAACACCCATTTAATCTTCGCCGCTTAGAGTACTTATGGGGAGGAGTTGTTGCAATGTCGGCATTCAGAGTGTTGTAACTGCGCCGCTGATCACAAGATTCTGTATTTTGGGTAAGTTTCAACTGTGGTGTTTGATCATCTGCACCACTGCTAGAGCTCCAGCCTCTAAGCCTAGACTTTATCATTTTCAAGCTCTCAGAAGGTTTTTGTAAGGGAGCCCCTATGTAATTTCTGCTACAGTTATTGATTGATACCGAAGCTGTCGATGTAGCTGTTAGTGATCCTTGTCGCGAGGAATGCTTATCTGAGGATGCTTGTAAAACGTTCACAGCTGAAAAACCTATAGCCCACGCGAAGCCAGCAAACGGTAAGAGAAGGAAGAGAAACCAACGAATGAGAAGTGAAGGGCGACAACAACGCTGCAGCATATTGATGCTGATGCTGATCACGCCAGAAGCTGCCCAAGAGAGCTAAGCTCATCATCAACGTGCGCTTCTCGCTGGGAGACAACGGGGAGGAAGGAAGAAGAGGAACGAGGAATGCGACATGGAGAGAGAGAGAGAGAGA |
| **GAMETE EXPRESSED 3 (GEX3):**  GGAAACAGATTCTGTGCTTGGAGGGAAAAGCTCTGCTGACTTCTGTTCCAAGCTGACAATAGAATCATTTGATGCACTAGTTGAATACTGATCAATTCCCTCAAAAAATTCATTAACGGGAGAAAATGATACAGTTTTTTTAGTGGAGCTTCGAGCTACGGTTGTGCTTTTATCATACAAGGGGTTACTGTACTTCTCGGTGATACTTTGGCCGAGAACTGCACTGGAGTCTTCCTTGCTGTTTTGATAATGAGAGGCCTGCATTGCACTTGATGATCGCTGCAAGCTTAAACTACGTTCATGGACTCGTGGCGTTATCCATTGAGGAAGAGTCAGTGTGAGAGGAGCTGTGGGGCAATCATCATATAACGAAAGAATGTGCATTTCCTCAGTGAAATCCTCGTCTTCCGGTGCACCGAGTAAAGCGCGGAAAGCAGTATCGAGCAACACAGTCCAAAACCCACCTCGTGAATTGGTAGATGTGAAATCCAGGGAAGCAATTGCATCCTCTACCAGAAGCTGCTCTTGACGAAAATGATCACCAAGCAATGTGTCTGATGGAAATAATGCCAAGTGTCTTTCTATGAGATGAAGCCCTTCAAGGGTGCCCTTTAACTGTTCGAGAATTTTAGAAGATCTAGGTTGAACCTCAAGTTCTCCCTGCAGATGCTTGATCGTTTGTTTGTACATTCTTTGTCTCTTTAAAAGCAGCCCCTGACGCTTCCGAAACCTCATCTGCTTCAGCAACAGACAAAGTCTGTGCTTGTGCAATGCTTGCTTTTTCCTCCAAACACAATATATGAGCACGCTCAAGAGTGACAATAACAGAAAGATGAGGAGAATAGAAACAGAGAAGGCAATGATTGGCCCATCATTACCTGGGTGTTGCACTCTTGTGACTGTAAACAAGAACTGTGAACAGCTGACTTCATGAAGTTTTTGTTCGGTTTTGCAGAAGCTCCTACTTGAAAGATTTGCAATTGATGTAAGCGCAATCAGCCAATCAGGGTCAATACTGTATGAGCTCAAGTCCCTCTTTACTAAATCTTCAGGAAATTGGTCTGTTCAAAAAACCAGGGTACAATGTATTCCACATGATCTGTCCACTTGATGGAACAACCATGTATAAGTTCATGCCCAAATATTGTGTGAAGGACAAAGCTTTGGTTCCATCCATTTGCTCCAATATAGTTTTCGACTTGACTCTAGTTTGAGCAACAAATAAAGCTTCTTTTGAGCAATCAAGAATAGGTGAAATTTGTATGCTGACTGGAGCCGAGTTGTCCGCTGCCAGATATTTGCTCACATATGCTCCATCTGGGGAAATGACATAAAGAAATCCATCCAGAGAGCCAATGGATACAAAACCTAGGAAGTCGACCACTGGGATGCAACTTGTTTCACTGAGAGGTCCAACCTTTGCTTGCCAAACCAGATTCCCCAACTCCGTCGATAAAGCATACACCATATCATATTTGGCCATGGCTGCATAAATGCGCCCATTACTTCCAGTAGTTATTTTGACTGATGATGGTACATCAAGACTGAACGTGTAACGCCACCTCATAAACGGTTGCCAGGTTGCATACGAAAAAAGCCAGCCGTCTGTACGAGCGACATAGATGCTTCCATCACATTGGTCCACCGCCAATGATGGAAGAAGGGTGCAATTTGTCATATTATGAGCACAAGGTCCCATAGACTCTGGAGACTTCATCACGCTATTGCTGAACTGGACAAGCGTGCTCCACAATGGTGTCCCATCCAACATAAATGCGTGAAGGCCTGTACCCGTGTTAATGTAAAGGACAGAGCTCCAAGATGTCACCGCAAGGCCCGTTATGGAAGAAAACTTGCCAATAGCTTTCAAGCTTATGGCTGGAACCATACTAAAAAAATCAGCTGCAGAAGCTTGTGTTGCTTGCGATGCTGGTGGAGTCACAACCACTACAGTCTGCTCTGCTGCCACATAAACCTTTCCATCA |
| **LONG CHAIN BASE2 (LCB2):**  TGTCATTTACTTTGAATTATAGAAGGCGAGCAATCAAGTTGACATTTCAATGAGCGAGAGTATTTAAATTGGGAAAGGACACATGGCGGGATATGGTTGGGTAGCGAATCAGAAAGTGAACGTCCCCTGAGACCATAATTATGGGAAATGGAGAAACGGGAATGGGAAATGGAACAACAATTTTGAGAAAAATCGAAACGGGGATATGCCATATCAGCAAGAAATTGTAAAATTTTGGGAAGGAAGGAGAGAAAAATTGAGAGAAAGGAAGGTGATGTATCGAATTTTAAGGTTAATGTCAAGATTTTGCTCTATATTACTAAGATATTGAATGAAGCAATATGTGAGGAACAATTAATGCGATGTTATTGTTTCAATTTGAGATCGGAAATGGACTGAAACAGCCGAAACGGGAAATGGAGTTTCCAGTAACTATGCCTCAAACTACACCCATGCAGTTTGCACAGCACCCAGCTTGAGCCTTCTGTAGCTCATCTGATCTGGAAACACCTTGCAATGAAAACCCAGAATAGTAGCAATAATCTGGGCCTTCTGGACCAGATTAGGAAGCATACATAATGGTTTTGCCCCTTATTTGGGCATGGAAGGAATTAAAATGTTGTTTGAGTGTTTTATGGCATTGTTTAACTCCACTCCATCTCCTCAGTCGAACAGAGAAGACACGGACTTGCATAAATCTGGGTTCATACAATTATCTTGGATTTGCTGCTTCTGATGAATATTGTACACCTCGAGTAATTCAGTCGTTGGGAAAGTTTGCTCAGAGCACATGCAGCAGCCGTGTGGATGGTGGAAACACAGTGCTGCATGAGGAGCTTGAAAATTTGATTGCAAGATTTGTCGGAAAGCCTGCTGCAATGGTGTACGGTATGGGTTTTGCGACCAACTCTACGACTCTGCCATCCTTGATAGGAAGGGGAGGTCTGATTATAAGTGATGCTTTAAACCACTCATCAATAGTCGCAGGAGCACGTGCTTCAGGTGCCAAAGTGGCAGTCTTCAGCCATAACACACCAGAGCATTTGGAGGAGGTTTTGCGGGAAGCGATTGCCGAAGGGCAGCCAAGAACTCACAGACCTTGGAAGAAGATAATTGTTGTGATCGAGGGCATTTACAGTATGGAAGGAGAGCTCTGCAAACTTAGTGAGATAGTGGCAGTCTGCAAGAAGTACAAGGTATACATCTACCTTGATGAAGCTCATAGTATAGGAGCCATTGGCAAAACAGGCCGAGGTATTTGTGAGCTTGCTGGCGTCAATCCTGATGATGTCGACATAATGATGGGCACCTTTACGAAGTCTTTTGGATCTTGTGGAGGTTACATTGCTGGCTCAAAGGATCTTATAAAGTATTTGAAGTTTGTTAGTCCTGGGCATCTGTACGCAACGGCAATGTCTCCACCAGCAGTTGAGCAGGTCATCTCGGCTCTGAAGGTCATCCTTGGTGAAGATGGTACAAATCGAGGGGCTAAGAAGCTAGCACAAATAAGGGACAACAGCAACTTTTTTCGAGAGGAGCTTAGAAAAATGGGCTGCGAGGTTATCGGCGATCCGGACTCACCCGTGATGCCAATAATGCTGTACAATCCTGCCAAAATCCCAGCTTTTTCAAGAGAGTGCTTCAAGCGGAATGTCGCAGTTGTAACCGTTGCGTTTCCTGCTACCCCATTGCTGCTAGCAAGGGCTCGCATTTGCATATCCGCAGCTCATACAAGAGAGGATTTACAACGAGCACTTGAGGTAATAAATGAAGTAAGTGACATGGTGCACTTGAAATACTTTCCCTTTCTTAAAGATGCTGAGGAAGAGCAGCAGAAAAAGAAGCTGCAATGATACCTTGTTTGAAATAGTAGACAATTGGGCTGAAAGTATCTTGAGATGATAACAGCTAGCTCAGCTTTGGAAAGCCAATAGAAGTGCTTGGTCATTTTTTTCCCATTTAATGTATCAGTATCAAGCAGGGTCCTCCCTCTATTTAATCACACTTCTTGACCATTCCAGAAATTTGGAGGAGAGTATTCTTTTTGTGACTTTAAGACCTAATGAATGTTTTTTTACCATGATTCATTGTGCATTTTGTATTCGGTTGTGAGTCGACGCTTTTTCTGAATGATAGTTGGTGTCTATTTTGG |
| **FERONIA (FER):**  AAGTGGGGCGTTCAAGCAAACCATAGCCATAATTTATAAACTACAAGACTTCTTCTTCTCTTTGTTTCGTTCATATCATTGATTGCATCAGTATCAGTACATATCTCCTTTTCCCCCATTGATACGCTCCTTTGCGCTTTCTGCTTTCTGTCAGAGGCTCGTAGATACAATTCGCAAGTGCCAAGAAGCATGGGCGATCTCCTCATGCATCCAAACATTCGGGAATTTTTGGTAATTTGCCCATCAGAGCTTTTTTTGCTTTGAGAGATGGTGTTTTGAAGGTGCCCAACCCTTCGCAGCCTGAACAATTAGGGTTTTCTTCATGGGTCTCCTTCATTTCTCTTTCTTCAATCACTGGTAAGCTTTCTTTCCGAAGCTCATTAGCTTTAAAGTCTCAGACATGTGTCTGCGATTCGCCTGCAAAATGAAAAGCGGTGCAAATTGCTCATTGCACAGTTGTATTTCTTGATCTTTCAGTAGTCAATGATAAGCTTCGACTGCCAAGCTTTCCATTAAAGCTTCACAACTTCAAGGTCACACAAATGGGTGTTTTTGAATAATGAAGCAAATCCTAGAAGTTTACAAGCTGTAAAGAGCTGAATTCCCTTTTTTGTTTCTTCGTCCTGAAGCTTTTACCAAGCACCAAATCAGTTTTTTTTATTTGAGCCAAGCATTGAATATCTCATGGTTTTCTGATGGCTTCAACAATCCTAACACGCTTGTCTTGAATGGGCTTTTCAAGATCTCTCTTGTGGAGTGAGTTATCATCTTTCTGGTGCTCCTGAGGAGCTTTTCTCGGTGTTGGGGTTCCATCAAGTTCAAAGGCTTCTAGGTCTTCTTGCTTTGTGGCGGATTTGCGTCCAAGCTTTGCTTTTGAAGGCTTGACTGATTTTTGTCATGCCAAGCTATGCCCAAGATAGCTCCTTACCCCAGTAACATGCTTGCAATAGTCATATCAATTTAACACCATCAGTCATCAGATTACTTAATATTTGGGCTGATATCTTTCAGTTTCACGTCAAAGCCTTTAATGGGGGTCATTACTTTACTTCTCCATATTGTTTCATTTCTGTTTCTGCATATTACATGCCTCTTTGTTCTTGTACAAGGGCTATCCTACACCCCTAAGGATAACTACCTTATCGCCTGTGGGAGCTCCCTTGATAGCGCTACAGATTCTGACGGTAGAACATGGGTGGGTGATGAGTCTCCTTCTGCTTCACCATTTTTGGCTGGAAATCCTGTATCTGTGGCAGCTTCTACTAGTGTTCAAAACCCATATCTCCCAAGCACAGTTCCTTTTCTTGGCGCTCGCATTATCACATCTCCAGTTTTGTATTCGTTCCCTGTTACGCCAGGGCGCCATTGGTTGCGCTTGTATTTCTATCCTTTTGCCTATGAAAATTATGATCCTGATTTGGCAATTGTCAGCGTGAATGTAGATAATTATGCTCTGGTTGCCAATATGAGCATTACTAGAGAGATGAATGCTCTGAACTATGGGTACATTCAGAAAGAGTTTTCCATCAATGTGACCTCCAAGTCATTGTTGGTGAGCTTCATTCCAGGCAAAGCAAATCATGCATATGCTATGGTGAATGGAATAGAGGTGATTTCAATGCCGGATAACATGTTTGAAGACAGCATATATAATGTTGATCTTTCAGCAGACATACCCATTGCCATGAGTGCGACGGCTCTAGAAACTATGTATCGGGTGAATGTGGGCGGACAAGCAGTTAGTGCAGCAACCGATGAGCTTTCTCGAACTTGGGGCACCGACATTGGTTTTATCCCCACAGCAGCAACAGGAGTTGCAATGAATACAGAACACCATATACAATACGCAACCGCCAACAACTACACTGCCCCTCAAGTTGTCTATCAAACAGCCCGGACTATGACAAATGATGACCATGTTAATTTAAATTTTAACCTCACATGGCGCTTCTCTGTCGATCCTGCATTCACCTACTACGTTAGGCTTCATTTCTGTGAGTTTGTTTACCCATTGACCAACATGAGGGTTTTTGACATCTTCATCAACTCAGAGGTCGCCGAACTGTCCTTTGACGTCAATGCACAGGCATCTGTTTTGACTGGCAATACAGGAAGTGGTGAGTACAAAGCTGTTGTGATGGATTTTGCTACTTCAATTGCATCCAAGAGTGCATGGAATGCCAGTGATATAATACTTGCACTTCATCCTAACAGTACGAGTGCCCCAACAAATTTCAATTCCATATTGAATGGCTTGGAAATTTTTAAACTAAACGATTCTTCTGGAAATTTGCAAGGCCCTCCTCCCCCTCTTGTTGCAGTTCCTAGTGGTACTAGCACAGCAAGGCAATCTTCGCCTTCTCATGGAGGGCTTAAACAACCAATCATCGGTGGGGTGGCAGGGGCTGCAGCTGCAGTTGCATTACTGTTGGTAGGAGGCTTTCTATGCATGCATAGGCGCAAGAAAAGCGCCAAGAACTCCTCACAAGCTTGGCTGCCTTTACCTCTCTATGGAGGTACGTCTCGGTCACTTATCAGTAAAGGGTCTACTGCATCTCCAAAGAGTGCAGCGGGTAGCTATGCATCGTCGGCACCTTCTAACCTGAGTCGCCATTTCACATTCGAAGAGATATCCTCCATGACTAACAATTTTGATGAGGCCAGGGTCCTGGGTGTGGGGGGCTTTGGAAAAGTCTATGAAGGAGTGATTGAGGATGGAACGAAGGTTGCAGTGAAGCGAGGCAACTCTACTTCAGAACAAGGTGTCATGGAGTTTCAGACAGAGATTGAGCTACTCTCTAAGCTTCGCCATCGCCATCTTGTCTCGCTCATTGGCTATTGTGAAGAACACAACGAGATGATTCTGGTATATGATTGCATGGCAAACGGTCCTCTGAGAGGTCACCTTTATGGTACTGATCTTCCTAAATTATCATGGAAACAAAGGCTTGAGATATGCATTGGGGCTGCTCGTGGTCTTCATTATCTTCATACAGGGTCTGCACAAGGCATCATACACAGAGATGTGAAAACAACCAACATACTCTTGGATGAAAATCTCTTAGCTAAGGTATCAGACTTCGGGCTTTCCAAGACAGGCCCTACTCTTGATCATACACATGTGAGCACAGCTGTAAAAGGAAGCTTTGGGTATCTGGATCCAGAGTACTTTCGAAGACAACAGCTTACAGATAAGTCAGATGTATACTCTTTTGGGGTAGTCTTGATGGAGGCACTGTGTGCGAGGCCAGTTATCAACCCCTCTTTACCAAGAGAGCAAATAAATTTAGCTGAATGGGCAATGAAGTGGCAGAAGAAGGGGATGCTGGATCAGATTATTGACCCTTATTTAGTTGGAAGAATAAGCAGAGAATCATTGAACAAGTTTGCGGAGACAGCTGAAAGGTGTCTACAGGATCGCGGCTCTGAGAGGCCAACTATGGGGGATGTTCTGTGGAACTTGGAATATGCCTTGCAGCTCCACGAATCTTCTGTGGAGAAAGCATTGGATGAGAGCAAGCCTCGAATAGTGGATTTGCCGATTCGAGCATTGGATACAAGTGAACTCGATGATAGCTCTGAGGTGAAGATAAAAAACAGTAATGAGAGCTACAGTAGCCCAAGGACTCAGAACTTGCTAAGCGAAGACTCGGATGATACTTCCATCAGTGCAGTCTTCTCTCAGCTGGTTAACCCTCAAGGACGCTAGACTGCTTTTGATCTTCTCCCCATGAAGAGATTGTAAAGGTAAGCTGGGCCAGTGTATTTTTGTACAAAATAATTCCTTTTGGTTTTTGGCTGTTGATAGGTGCACTGTTTAGTGTTCAAGAAGACTAGCCAAACCCTTTGTTGTGTACACTATTGTTGGCTGTTAAAGGTTTGCCAGTTTTTAAGGAAATATGCCAAAAAGCTTACATTTGTACACTAGCATTATTGGATGCTGCTAAAGGCACAATTTCCTATCATTCTTGCCGGATCTAGATGCTACTGTGTTAAGGAAAATTGTGTTGCATCTTAGCTTTGGTGAGAGTTGTCATGCGCAAGTCTAGGGGCTAGTTTGAATTTAGGACTTGACATATTGAGGGAATTGGGTGTGAGCTGTTTGCATTCTCCATCTCTTTGTTTCTAGCGGGTTGTTGTTTGTGCCATAGATGGCTGATTGTAATAGAAATACAACTATGTAAGCAAAGAATTTCCTTTTACAGATGTCATGTTATCCATGGAATCAGTTAGAAGTGCTTGATACTTAGCTTTTATCCATTGCCTCAAAGCTGTTCCTTGGTAGGTTTTGCTTTTA |
| **F BOX-LIKE17 (FBL17):**  CATACACACATTCAAAACAAATCCCTCCATGCTAGCCTAGCCACTGTGATGTAGGAAAATGTTTGCATCCATTGTTTACACCACGCATGTGCTACAATTTGCCCCCACTACTAGTGATTTCCATCAAGGAAGTTCATTAGAATTGACTGTCTACTAATTCCAACATTCCATTCACGGATTTGATTAAATGATTACCTCTTGGTCTTACAAGAAGGGAGCATTCATGCTTGTTACGTGCATGGGATGACAAAGCTATTGATCTCAGTGTAATGAGAAGTTAACAATGTTAAATGTGTTGATTAGAAAGGCATTCATTGCAAACCTCTCAAAGCTAGTTGGAGCTTGTAAGAAAAGATTATGTAAATGATTTTGAGTTGAGGAGATGAGACTGGCAAATGTGCCTTTCAGGACATCTCAAGTATTGCTTGTTTGCCGTATACAAATACTTTTACTATCATCGTAGTGCTTGGACACGCTTCGAGCCATCTGCTTGTCCCCTCCAGATTGTAGTCGGATCAAAGTGAGCCAGCTCCAGCTATAACAAAAAAAGAAAAAGTTCCATTAAACCAAGTAACTACAATCTGAATTGACTAAACCATCTTAAACCAAGTATCTTGCCTCCTTTTAGCTGGTATCAACAATTTAACAGAAAGAAAGCTTTTTCTGATATCATCCATCCCTACAGATGCCTACCTGACCGTGAAGTTCGTGTACCAGCTCTTCTTCGCACTTTCCCACATAGACATCTTTTAAGGATGGGCATAACAATTTCAGTCCACCCGGTTGAAGTTTTGAACAACCCGTCAAATTGAGGTCCACAAGACTTGGGCAGTCCAAAACTAGCACCTTAAGTCCAGTGCAGCCCCAAAAACTCAGCTTCTGTAGTTGCTGATGCTGAAGCCGCAACTCCCCACGTCCAGGTTTAGCTTCTAAATGATTAGGCTCAATAAAACATGTACTGTCAGCATTGTCTGCCCATGTGTAGGTCCTCTTATCAGTAATGACCTTCCCACAATCTAGAAGCTGCAGAAGAGGAAGCTTAGAAACCACCATCTGCAAACCAACTTGAGATACTCTTGGACAGAGAGCAATCAAAAGCTTGGATAGACTTTTTGAGAACGACTTGCAAATCAGTTCAAGTGCCATGTCAGTGATGCTTGACCCACTCAGATCAAGCAACTCTAGATTCTCACAGTTAGCCACAATTGCATCCACGGAACAGTCTGTAATGCCCAATCCAAATGTGAGGGAAAGCATCCGTAACGACTTTAAGCCAGGAGTGAAAAGACCATGGACAACACGATCTTGTAGCCAGGGAGAAGCAACATGAAGGCGGGTCAGACCTCTTGCAGTATTTCCCAAATTTTTCATAATAGCTGCTAGGCTGTCTGATTTCACGTGGTCCTGCTCCAATGGATCCACATCTTCACGAGGCAGGAAGTCTAAGCACAGCTCTGATAACTCTGGACAGTCCAGATACATATATTTGAGGTGATGGCATCCAGTCAACCAAAGCGTTGCAAGGCGTGGACTGCTTAGCTCCACTTCAATCACACGATCACAACCCTCAAACTTAACAGATGTCAAAGATCTGCAGTAGCGGAATAGGTCTCTTAACCCTTCACCGGATATTGTGTTTACACCACCAGGTTTCAATGATACTTCCAGAGATGAAAGCTGGTAGCGACTGAAAGTAGAAATGCGCTTGAGCAGTTGATCTCCCAGATGGCTTGTCACGTCCAGGGTGAGCTGCTGAAGATTCGAACACTTCCGCAGGATGAATGGGAGGAAATCTGCGTCGCCTTCTACCCTGTTAGAATGAGACGCCCTGATCGAGACGATTGTTTTCCGCAAACCTGCCCAGACATACTCCGCACATTGACGCCACCGCCTACATACACTGCTAGCAGCTACAAGATCTTGCGGGAAGAGACGGGATAAGATCTGCACCAGACAAGCAAGTGGCATCGCCTCACAGTCCTTTGCTTCATTCACTTCCAGATCGTAACTGCTGTTTAAGCCAAAACAGCGAAGACCAGCATGAGGACACCTGCCTGCAAATTCATCAGCGACCAGCAAAGCAGTGTGTGACCATGAATCCGCGAAATACTTATTGACAAAGTGAACCGCGAGGATATTCCGATCCGAGAAGCAAGGCTGGGGAAACGAGGGCAGCTTCAAGTACATCAAGTACAAGGTAGGTTTTCACGGGGACATAAAGAACAATGGGGTTCTACAACAGCAAGAAAATGACGTTTTTCTATCACATGAGAGGTCACAAACGACGGCACAGAAGAACCAACGGGACTAGAGAGTAGCCTCTACACAGACAAAAGAAGAATTTTGATAGGGAGGGCACCTTAAAGAGTAAGCACATAGAGGATTAAGCTTTACTCTTATATCATACAGGCGGATTAGAGGAAATCGAGGGCACTTCTGCGAAATGAGCAGACAATGCATGTTCCCAACCGACAAAATCACGAGTTGAAGAACAAAAGAATCAAGCCTTTAGGATATCAGAGC |
| **ARGONAUTE 1 (AGO1):**  CCTGTTTCCCCCTTCCCTTCAAGATCAGCTTATCTTTTTTGGGTTTCTTGCGTCTCGCCAATCGCAGTTTTGTGTATCGCCTGTTGCGCGCACACATTGTGCCGCTTCAATATCATCGGCTACCCTCGTTTTCTTTTTCTGTTTTGTCTGTTTCTCTGCATCATGCCTCCTCGTAGAGGTCAAGGGCGTGGTCCTAGCGCCCCTGCCCGAGTTCCTGAATTTGAGGAGGATAGAGAAAGCAGCTTTCGTGGTGGTGAAGTGTGGAGGCAGCAGCAGCTGCATCCTCAACAGGAGCATCGACAGCAATACCCCCATGTGCAAGCGCATCGTGGTGGAGGCCGTGCTGGAGGGCGCGGTTTTTGGCAGCAACAGCCTTTTCTTGAAGAAAGAGGCGATCAAGGTTTGTCTGGATGGAGAACAAGTAGTGGTGGAAGAGCAGGTCAGGATCCTGCACGTGCACCGTCTCAGGATTGGCGTGCAGGTGCAGGGGTGCTTCCTAGGACGGTGCGAATTGTAGCTCCCGAACCTGTGGGAAGCTTTGAGTATGGTGGAAGGGGGGAAGATCAAGGAGGTGGAGGAGGGAGCCTTACACATGGTGGACGTAGAGGGGAAGATCAAGGAGGTGTTGGTGCTTTTATGTATGGTGGTAGAAGAGGGGAAGATCAAGGTGGTGGGGGTGGCATTGGCTATGGGGGTAGAAGAGGTGAAGATCAAGGTATTGGCGGAAGAAGAGAAGAGTATGGAGGGGGAGGGGGCACCAGGTATGGCGGGAGAAGAGGAGATGATCAAGGAGGGGGTGCCAGTTTTGGCGGGAGGAGGAGTGAAGATCAAGGAGGGGGGGAAGGCCCTGGCTTTTCAATCAAAAGAGGGGATGATCAGGGAGGACCTGGCTATGGTGCGAGAAGAGTTGAAGATCGGGGAGGAGGGGGGCCCATCTTTGGCGGGAGAAGAGGGGAAGATTATGGAAGGGGAGCCCGAAGAAGTGGAGGTGCAAGAGGAGGGGGACGATTCATCGACTTGGAAGGCAGAGGTCTCGAGGAATCTGAAGCTGGAGGTAGCAGCATTGCGAGCGGGAGCTACAGCGGACGTGGAGGTGCAAGGAGAGGCGGTCGCTCATTCCAAGCACCATCTCAGGGTTTCACGTTCTTCTCCCTCTTCTGTTGTTAGTTTTATCATGGTTACATGTATTCTAGAATTTGTATGTGTAGGCAGCAATGGCCCGGAGTTCTGTAAGGACTTGCATTTGGCGACCGTATTGCAATGCCAGGGGTTAAATATTAGTGTGTCTTGTAGTGCTTAGAGAACTTCCCCATCTAATCCTGATGATGTTAGCCCTCACAAAGGAGGTTCTCTTTTCTGAAGCAACTCGAGGAAATAGGTGTTTAGTGTTGATCGCGATAGTTGTGCATGAGGTAATTCTGGCAATTTTGGGTTTTCCTGGTAAGGATGTAAGCAAAACACTCGCTCGACCAAAACCTTATTCAGGGTGGAATATGTCTGATCTTCGCAGCCATTAGCTTGGTCGAGTATGTGCTGTTTTGCAACTAACACATGAGTTTTGAAGCAGCCTGGTTGTATCTTTAATCTGCCTTCCCGTTTGTTTGTTGCATCTTGCATTCACCTGTAGCTTGTCGATCCTATAACAGAAGCAATTTGTTAGATTGTTATACATGCTTTTTTTTCCTGGCATTTATGTAGAGGTGGCTATTAGTCTAACTGACAATGGCAGCAGAACTACTGTAGATGTGTGGGAAAAAGGGCATTTTCATCCCGTGCTGTGTAGGTGTTGTGTAATTTGCTAAAGTAGGGAGCCTAGCATCACGAAATCACACATACGCACTCGAGAATAGGGGACTCAATCAATGCACACAAGTTTAAGTAGTAGTCCAAACACTGAACAAGCTTTCAAATGCAAAATCTTCCCTCCATGTCAGATCTGAGTCGGTTGGCCAAATGGTGGAGTCAGTTTTGATGTGTGCCTGTACACCATGCATTCTTCTAATCATGATTGACTTGGTGTTTTAATTCTTTTCATTTGATCAAGATGCTATTCAAAATTTTGATATCTGTGGTTGTAAAAATAGCTAATTAGTTTTTTTGGCTCATGGCTGTAGAAAGTAGTTGGTTGGCTTTGATTAATTTATTGGCTCAAGCAGGATCGTGGGCTTTATCAGCACCACCGTTGGGCGAGCTCCTTCCAGGTTTCTCTGGCTTGGGGATACATGAGGAACCATCCCAACCTGGTTCTACATCTGCAGTAGAGGAAGCGTTGCCCCAAAATGTGGATGAAGGGGAGACGTCTGCCATTGTGCCACAGGAGCTTCCATCCGCATCGGGGTCCTCTTCACGATCATCTTTGAAGCTGATTGCTCCCAAAAGGCCACTTCCTGGAAGCACAGGGCTAGCTATCAAAGTTCGGGCGAATCATTTCAAGGTAACCTTCCATCCTGGTGACAATATCTATCAATATGATGTGGATATCAGCCCGAAAATCTCATCCAAGATTGTTGCTCGCACTCTTGAACGCCAGCTAGTTGAGATGTATAGTTCTGATTTCAATGGCAAACTTCCAGTTTATGACGGCAGCAAAAGTATGTACACTCATGGTCCCCTTCCATTTGAGCATTGTGAGTTTGTTGTCACTCTTTCAGAAGAGAGAGGAACTACTGGCAGGGAGAAGAAATTCACTGTAGCAATCCGTCATGTCTCTACCCTGAACAGGAAAAACTTGGAAGATTTTATGAAAGGCAAGCAGGTTCCAACTCCACAAGAGCATCTCCAGGCCCTTGATGTGTTACTGAGAGAGCACCCTGCATTACATTTCATAGCAGTTAGCAGGTCTTTTTTCAAAAGCGAGTTAGGTAGTGCTCAGTTGGAGGGAGGGCTTGTTGCATTGAACGGTTTTTATCAAAGTCTTCGACCTACAGAGAGTGGATTGCAACTCAACATTGATTTGTCCACAACAGCATTTCATGCTAGCATTCCTATAATTGATTTTCTCAGGCAGCAGTTAAGGAATTTCGACCCACGGTATCGGCTAACAGATGTGGTCCGTGTGAAGGTCAAGAGAGCACTTGCTCGCTTGAAGGTGCAAGTTATTCACAGGCAAACACCTCGGAGATACAGGATATCTGGCCTTTCCACAAGCCCGACGAAAGATTTGAAGTTTCCCATCGAAGGTGGGGAGGAAATGCGGGTTGTGGATTATTTCAAGCTGACGTACAATTATGTGATCGAGTTTCCAGAACTCCCTTGTTTACAAGTTCAAGCCAACAAGCCTAGCTACCTGCCAATGGAGGTTTGTGTGATCTGTGATGGCCAGAAATATGGTGGCAAGCTCAATGACAGGCAAACCACGAGATTAAGAGGGCTGGCTTGTGTCCTGCCTAAAGTGAGGGAAGCTAAAATTAGGAGCATTATGAACAACGATGATGGTCCAGGCAGAGGTCCGCATGTAAGGAATTTCGGTGTGCAAGTAGCCTCTGAGATGACCTTGGTGAATGCTCGCCTTCTGCCTCCCCCTAAGCTCAGGTATGGAGATCAAGGGAAAGTTAAGGAGATAGTTCCCACTGATGGTGCATGGAATCTGTTAAACTCCTGTGTCGTGGAGGGTGGGAATGTTGCTTATTGGGCTCTAATCAGCTTTGATCAGACTGTGAATGACTATATTGCTGGCAATTTTATATCCAGCCTAAGCAAGCGGTGCAATGACCTTGGTATTCAGATGGCTGAACAGACAGTAATCCCTCCAGTCTTGCGCAGGTGGGAGGACCTTGAGACCCCACGACTCGAGAAGAATCTGAGATTTGTCTATGACCAAGCTTCTCAAGTCATTCACAGTAGTGAAGGGCAGGGAATACGTCTGCAATTGCTTGTTTGTGTAATGGCTGATAAACACCCAGCTTATGGTGAGCTGAAGAGGATTTGTGAAACCCAGATAGGTATTGTAACTCAATGTTGTCTGTCAAGGCATGTAAAACAATGCAAGTCTCAATATTTGGCGAACTTGGCTCTGAAGGTGAATGCTAAGGCTGGTGGAAGAAATGTTACATTGGCACTTGAGCTTCCCAAGATGTGCCCTGTTTTCAATAGACCGACAATTATATTTGGCGCAGATGTCACACATCCATCTCCAGGTGATGATACAGGACCCTCCATTGCAGCTGTTGTGGCCAACATAGACTGGCCTTCCGCCAACAGATACATAGCACGCGTGCGTGCACAAACTCACAGAGAGGAGATAATTGAGTACTTGAGGGAGATGGTGCAGGAGCTTTGGCATGAGTTTTGTGAAAAGACAAAGAGTCGGCCGGATCGCGTGATAATGTTTAGAGATGGTGTAAGTGAAGGTCAATTTGATGAAGTCCTGCAGAGGGAGGTGGCCGCATTAAAGGATGCTTTTATAGAGGTGGGTGGCCCAGATTACAAGCCTTTAATCACATGGGCGGTGGTGCAAAAGAGGCATCATACCAGGCTATTTCCTGCCGATGACAAATGCAAGGATAAGAACAACAATATCTTACCAGGCACCGTGGTAGACTCAACCATCACGCACCCAAGGGAGTTTGATTTTTTTCTTTGCAGTCATGCTGGGATACAGGGCACAAGCAGGCCGACACATTATCATGTGCTTTGGGATGAGAACAATTTTAAGTCTGATGATCTGCAGGGTCTGGTTTACAATCTATGCTATACTTATGCTCGGTGCACACGTTCGGTTTCTGTTGTTCCCCCGGCTTATTATGCACATCTTGCAGCATATAGGGCTCGCCTTTACCTGGACTCTCTAGGTGGTTCCGATACTTCTGCTTCGTTACGGGGTCAGCGCTCAGGTACTGGATCAAGTGGTGGAGGTGGCAGCTCTAGAGCTGCTGCTCCTGCTGTGCGTCATCTTCCTCGTGTGCAACGCAATGTGCAAGAAGTTATGTACTTCTGTTGAGAAGTCTGACAATCATCATTGACTTGTTTAATTTTTGAATGACTTGCATACACAATTTTGTCTGGTCATGTGATCGGTGCTTAGTTTCTGGACAACATTAATTACTCAGTTGTTGCCATGTGGCCAGCCTAATGCAGGTACCTGAACCTAAATCTTTGAGGAGCAGAATCTTCTGCATTTTGCTTTTGTGTGAAGACTTTTACCGGGTCTTAAGTGTTTTCTCTTTCTTGCAGTTGAGATTCAATTTTGAGGGTGTGTAACTTACACCCTTGGTTCAGCTACTGGATGCTCGACATGATTAGTATGCATGCAAATTCCACATTGTGAAGTGTTTGTCTGATACACCCTTGGTTCAGGTACTGGATGCTCGAAATGATTAGTATGCATGCAAATTTCACATTGTGAAGT |
| **ARGONAUTE 7 (AGO7):**  GCTGATCATATCTACCAGTATGATGTGAAGATGGTAAACTCACGGGTGCGACAAGTCCCTGACAAGCCTTTTAAACCTCTTCCGAAAAAGATAAGCAGAAGGATTAAAGAACAGCTTATTAAGTCTTATCCTGATGTATTTGGGAACATAAATCCAGTGTTTGATGGCAGCCAGAATTTCTTCACAACAAGCATGCTTCCAATTACTGAGCTGAAAAGCTTCGACGTGACCATCGTTGATGATTGCAATCACCAGCCGATAACGTACTCTGTGACGGTGCAATTAAGCGCGAGCTTCGCAATTGCGAGTGTGCAGAATTACATCTTTGGGCAAGTTGTCGAGATGCCTCAGGCTGTGCTACAAGCTTTGGATGTGGCAATGAGGGAAACATTGTTGGGTCATTACACAGAACAGGGTAAATCATTCTACGCATCAAGCACAAACAAACAAAAATACAGTGGATTTCAGACTGATTACATGGAAATATGGGATGGTTTCTTTCAAAGCTTGAGGATCACAGAGCAGGGTCTAGCTCTGAATTTGAAAAAATCGCATGCAGCATTCTTTGCAGATAATAAACTCCATGACAAAATTACAGTTGCAAATTATATCTTCTATTTGAACCAATGCCGTCCTATTGATTTAGAGGGCTTAAAGAATGAATTGAAAGGGGTGAAGGTGCTAGTTCACATGGAAAGGGATGCTGGACAGGAACCAATTAAGAGCCGGATGTACAAGATAGAAGCCATCAGGAATAATGCATATGAGGAGAAGTTTATTAAAAATGGGAGAGAGGTGTCTGTGGCACAGCATTTTGAAGAAACTGGGAGAACACTCCATTTTCCAGAAAGTCCGTGCGTGAAGGTGCTCCACTTGCGCAACATACTCTTCCCCTCTGAGCTTTGTGTTATCGAGTACCCCCAGCACATTTCCAAGACGCTATGCTTTGCACCGCACCGCAAACATATGCTCGATGTAGCTGCTGCCCTCGACCCCACAGAACATTGCAAACAGATTCAAGACCTCATGCCAGAACCTCCCACAGCTGCATCGCCCTTACCCTATGTTGGCCCCCCGCGCCAAATTTGGGGTGTTGTCACAGTACACCAAAAGGGTTTGAGATGCAGGTGTCCTGTTCGATGACGGAGTTAGAGGCGAGGCTAATGCGAGCACCCAAGCTGAAGCAAATGCCAAGCAGGCCAGGGATGCAACCACTACCAATAGTAATAGAAGACAAAGGATCATGGAGTCTAAAATCAATAAAAGTAGCAGAAGCAGCAGAAGACATCACTAAATGGCTGCTAATCACCTTTTCTAACGGTCCAAATCAAAAGAATTCCCATTTTTCTAATTTCCCAAGGCAGCTCTCTACAAGATGCTTCCAACTTGGCTTTAAATTAGCAGAAGCCCCTGCCCTTAGAGCTTCTGAATGTTGCATCCCTTTCTCTTACCTCTCCAACTATGAGGTCTTGAAGACCAAGATCTCAGATTTTCTAAAAGCCGCAAAGTCGGTGCGTCCGCTTCCGCACAGTACTAAGCTTCTTGTTTGCATGTTCCCTTGTAAATCCCCCTCCGGTTATGGCTTCCTCAAACACATCTGCGACTATCAACTTGGCGTCGCCTCTCAATGCTGCTCCTTGATAAAATGCACCCAACCTGTACCTGCCCAGCTCGATCAGTACCTCGGCAACCTTGCTCTCAAAATCAATGTCAAACTCGGCGGTGTTACCTCCCTCTTGTACGAGCCACCATTCCCCGAACAAATGAAGTCGATCCTCCTCCTCGGAGCCGATGTATCGCATGCGGGCAGCATTGATGACCCAGCAGTAGCTGCTGTCGTCGGCAGCACCGACTGTAATGCAGTGAAGTACATCGCCCGCATCTGCTTGCAAAAGGCTAAGATGGAAATCATTCAGGATTTCAAATCGATGGCAGAACACATGTTGAAATTTTTTTCGAGACGGAATGGTGCAGCGCCGGGGGGAGTGATCATGTTCCGAGATGGAGTGAGCGAGGGACAGTTTGAGGAGGTATTAAACAAGGAGGTGAGGGAGCTAAGGGCAGCGATAGACAGCGTGTACTCGAACCTGAGAGAGGAAGCGAAGCCAAAGATCACATGGGTGGTGGTGCAAAAGAGGAACAACACAAGGCTATTTCCTGCTCAAAAGGAAATAAATGATGGGAATGGCAATGTAAAACCAGGGACAGTGGTAGATCACACCATAACTCATCCTTTTAACTTTGATTTCTACCTGTATAGTCATGCAGGAATCCAAAAGAAAGGCACCAGCAAGCCCACCCACTACCATGTCCTCTTTGATGAGAACCGCTTCACATCTGAGGCCTTGCAAGAAATGGTTTACAAATTGTGCTACACCTACGCCCGTTGCACTCGCTCTCTTTCCATTGCGCCGCCCGCTTACTACGCTCATCTCGCCGCCGCTCGCGGCTGGCTCCTCTACGACGCTCGCCGAAAGATGAAGACTCCACTACCCTCCGACCGACCGTTTTGTATCACTGAAGCCATCCCCGAACCCTTCCAAGAATCCATGTTCTTCTGTTGACAGCTTATGGAGTTCGTATTCAGCAGATCCATGTTTTGGAGTCCCG |
| **SLOW WALKER 2 (SWA2):**  GTTAGGAAAATTTTAAATTACAAAATGAAGCAGCAAACTTAGTTTCTGCTGCCATGGCGAACAATAAGGAGGACTTGAAGAGCAATAAGGAGGACTTGAAGAGCATACAGGCTGACGTCGCCTCCTTCGTTTCCGGCCTCGGCTTCTCTACAGCTTCTGTCAATGGAGATGGCTTCTATGATGGCGACTTCAGAAAGAAAGGTCGCATCTCTGATAAGAAGCCCCAGAAGGCGCCCCTTAATGCACAGAAAAGCTCGTCGGAGCTCAAACAGGCCAACAAGGAGAGTAAAATTCTCAAGAACAAATCAATCACCCCAGAACTTCATCAATCGCCCATGAAAAGCAATGGAAAGCTTGAAAAAACCAGCTTAAAACAGTCTTCTTCAAAGAGTGAGCTTACAGAGGAACCCAAGTTTCAACAGGGAAAGGGAAGCAGAGAACTTGACAAGGATGCATTGAAGAAGAATAAGAAGAAGCAGAAGAAGGAGGAGGAGCAGGAGGAGAAAGCAGAGAAGTTTTCTTCTGAGAAGAAGCATAAAGCAAGAGCTTCTGTGAGTCTTGCTATTAAGGAAAAACAAGAAAAGAAAGCTAATGTTTTTGGCAGTGCTGATTCTAAGAAGAAACGGAAAGCAGAGAATATGGACAGCAACGCAATTGCCCCTGCTAAAAAGCAGAAAGAAGAAGGGGCTCAAGGCTTCGTTGAGAAGGATCAAAATCTCTTAGCAAAGATGGACTCTGCAAGAGACGGTTCAAAGACGCTTTCAAAAATATGGTCACTTTTGAAGTCTGGACGGTGGTTTGAAGCATCATCATCATTATATTCAAGACAGAAAAGTAATGGAACTCTTGAGGGGGGAGACAAGAAGAGTGGTAAACTTTGTAATGGCATAGAGGGGAGTGCATTGGTAGCGAAAGCAAGAAGGAAAGGTGACGAGTTGATGGAAAGGGCTGCAGCAGAGTACGAGAAATCTCGTGGGAAGGACAGTGATATGCGGTGGCTTATGGTAGCTCGTAAAACAGGGACAACCGCCGACAAAGTTGCAGCTTTCACCGTTCTTTTGCAGGACAATGCAATTGCCAACCTCAAGTCATTAGATGCTCTTCTAGGGATGGTCACTTCAAAAGGAGGAAAGCGACATGCTGCAATGGGCATAGATGCACTAAAGGAGCTATTTGTGGCAAGTTTGTTGCCCGACCGGAAGCTTAAGTATTTGACGCAGCAGCCTCTACAAGTGCAGCCAGGCACACATGAAGCAGAAGAGCTATTATTCTTGTGGTATTGGGAGGATTGCTTGAAACAAAGGTATGAACAGTTTGTTGGTTCATTAGAGGAGGCAACAAAAGATAACCTTCCTTTTTTAAAAGAGAAAGCTGTGAAGACACTTTATGAGCTTCTCAAAAGCAAGCCTGAGCAAGAAAGGAAATTACTCTCTGCCCTTGTGAACAAACTTGGGGATCCTGAAAGAAAAGTTGCTTCAAATGCTGGCTACTATCTGTCTTGCCTTCTAACTGCTCACCCAAACATGAAGATGGTTGTGGTGCAAGAGGTGGATTTCTTTCTTTTCAGACCCCATTTGGGATTGCGGTCAAGATATCAAGCGGTTGTCTTCTTGAACCAGATTGTGCTGAGCAACAAAGGGGATGGACCGAAACTTGCCAAAAGACTGATCGATATCTATTTTTCGCTCTTCAAGGTCATAACAGCGGGCGAACCCAGTAATAACCAAGAAAAGGGTAAAGGCAATGCCAATGGGACACAACAAAGAAATAAGAAAAGGAATGAGGATGAGGACGATGCGGTTGTTGAAATCGATTCTCGTTTACTATCGGCACTTTTAACTGGAGTCAACAGGGCTTTTCCCTTTGTTTCAGCTGAGGATGTGGACACTCTTATAGAAGAGCATACTCCTGTTCTCTTTCGTTTGGTGCATTCGAAAAGCTTCAATGTCGGTGTGCAAGCTCTTATCCTCCTGTACCAGCTTCTTACAAAAAACAACGCTGTTAGTGATCGCCTTTATCGGGCTCTCTATGGGGTTCTTTTATCTCCTGGTCTGTCCAAGTCTTCCAAGATGGAGATGTTTCTTGGTCTAGTATTCAAGGCCACTAAATCAGATATCAATTCAAAAAGAATGGCTGCGTTTTCGAAGCGTTTGATGCAGTTGGCCGCCGTGACTTAAAGCTCTCTTTTGAGAAGTTGGACCGACTCTTTCATTCTTGAAGAATAGTATCAACTGAATGCAAGGATCTCCCTTGTCGCCTTGCAAGGGGCTCCCCAATTTGCGTGTGGATGTCTTCTACTGTTGTCAGAGATTCTCAAGTCAAGACCGACTCTTTGGCATGCAGTTACTCAGCCTGAAGATGCAGATGAAGAGATTGAGCATTTTGAAGACTTTAAAGAAGAAGATACAACAAAGGATGATACAGTTCGTCCCGATCAGCTGGATAGTAGTGCCATTCAGAGTTCAAGTGAAGCTATTGATAATGGAGAGGTGGCTATAGCGCTTGCAGGATGGCCTAAGGAAGGGCAATATAATCCTCGCCATCGGGATCCTTCCTTCTGTAATGCAGATCAGGCTTGCTGGTGGGAACTAACAGCACTAGCATCCCACGCACATCCATCTGTTGCAGCAATGGCCAAAACACTGCTCTCTGGTGCAAGTATTCTTTATGCTGGGGATCCGCTAAGGGACCTCACTTTCAGTGTTTTTCTTGACCGTTTTACAGAGAAAAAACCTAAACCACGCCGCAAAAGTGAAGCTTGGCATGGATCCTCTTTGTCCGCGCCAGCCAGAAAGGCTTCACTAATGACGACGCTGCCCATTGGAGATGAGTTCTTGAAATTATCGGAAAAGGAGGTAGCACCAGAGGATGTCGTGTTTCATAAATTTTACACATCCAAGGTAGATAGGAAACAGAAGTCCAGCAAGGCAGAAAAGAGGAAGGAAGATGCTGTTTCCATAACTGGAGAAGATGATCTGCTGGGTGATAATAGTGATGATGATGAAATCGAGGATCTGCTAGACCAGGATGAGGGGGTGGAGATGGGCTTGGATGATCCCTCTGAAGATGAGAATGAATCAGAGGGAGAATGGACTTACCATGAACCAGATGGTGGGTTTGAAAATGCAAAACATAACAGCTCTGATGACGTGGATTTTGAAGATATGAATAGGGCAAGCTCGGATGAAGATATACTCTCTGTGGACCTGAATGAGCATACTTTTGAGGAAGGCATGAATGAAGTGAAAGAATCTCAATCACTTGTCAATAAAAATTCCAAAAAGCACCCAAAAAAGCAGTCAACTTCTAGTAAAAGGGAAAAGAAACAGAAGGAGAAAAGTGCTTTTAATAAGGACGGCTCTAGTAAATCTCCTTTTGCCGATCTTGATGAGTATTCTCACCTTTTGGTGGATGAAAGACCTGAAAAGGGCAAGATAGATGCAGTACCAAGCATGAGGAAACGAAAGAAAGCTAAGTAATAGTAGTATCCCGAGACTCCAATTCTCCTGTGTGTGTGTTTGTTGCAGGGTGTTCCCTCCTCCTAGTTATCCTAATGACCTGCTATGCAGCGTGTGTTTGTGCTTGTATGCATGTACAGTTTTTTTTTTTTT |
| **SLOW WALKER 3 (SWA3):**  GGAGCAGGAGCAGGAGCAGGAGCCAGCCCTCTTCTCCACGAAGGCCAGGCTGCAGAAAAGCTCCACCAGCAGCGACAAGAAGAAGAGCGGGCATCAGCTCAACAAGCCTCTCCTCGCTCCCTCTGATGATGATTTGGCGAGCTTCTGTGACCTGGGTCTCCCCGAGTGGCTTGCTAACACCTGCCTTCACATGGGAATCAAACGCCCAACTCCTGTGCAGAGGAGCTGCATCCCCCACATCCTCAATGGCCTTGATGTGCTTGGCCTCGCCCAAACAGGTAGTGGCAAAACTGCAGCTTTCGCCCTCCCCATCCTCTACCGACTCGCTCAACGCCTCTATGGCGTTTTTGCCTTGGTCCTCACGCCCACCCGAGAGCTCGCCTTCCAGCTTTCTGACCAGTTCAAAGCCCTTGGCTCTGCTATCCACCTGCGTTGCTCTGTAATCATTGGTGGTCTCGGCATGATTGAGCAGGCCAAAGCTCTGATGCAGCGGCCACATGTTGTGATTGCTACTCCTGGACGCATTGCAGACCTCCTTAGGAATGACCCAGGCATAGCATCTGTGTTCAAGAGCCTTAAGTTCCTCGTCCTGGATGAGGCAGATCGCATTCTTGATGTTGGGTTTGAGGATGAGCTGGGGACAGTTTTGCAGAACCTGCCTTCTCAACGACAGACCCTATTGTTTTCAGCTACAATGACCCCTGAACTTAAGGCCTTACACGAGTTGTCTGGGGACAATGCTTACTTCTATGAGGCATATCAAGGCTTGCAGACAGTTGAATCACTACAACAACAGTACATTTTTATGCCTTTGGATGTTAAGGATGTGTATCTTTCATATGTCTTGTCTATTTTGGAAGACAAGAGCATTCGGTCTGTCATTATATTTGCTTCCACATGCAGGACTTGCCATCTTTTGAGTTTAATGCTGGATGAGCTTGGAATTCATACTGTAGCATTGCATTCTATGAAGTCACAACCTCAACGGCTGGCTTCTTTGAATCGTTTCAAATCGGGGCAAGCTCCTGTTTTAATTGCTACAGATGTTGCAAGTCGAGGCTTGGATATCCCAACTGTTGATCTAGTCATCAATTACGATGTTCCCAGGTTCACTCGCGACTATGTCCATCGTGTAGGTCGTACAGCACGTGCAGGCAGAGGTGGATTGAGTATCACTTTAGTGTCACAGTATGATGTGGATCTTGTGCATGAGATCGAGTCACTTATTGGAAAGCAGCTAGATGAGTATGAGCTAGAGGAGGAGGATGTGTTAAAGGGCATTACAAAGGTTTTCAAAGCCAAACGCCTGGCTTCACAACGAATGCAAGATAGTGGGTTCGAGGATACGGTCAAGGATCGAAAAGAGAAGAAACGAAAGGTGCTCAAGGAGAAACTTGGTTTGCAAAAGAGGAAGAAAAGGAAACAAAGTGAAGCAGCAAGATGACTTTACGTGTGTACCATTGCTGTGCCATAGGATTAGCTAGAAGGTTGAGGATTTAAATATAGGAAACCAAAAAGAATTTGATTTGAGTGGTTCAACAGGCTGGACATTTCAGTAATTAGATGAACTGGCTATGATGAATTTGTATAATTTTTTCGTTGTATTTCTCAGTGGAGAAATGTGGATGGCATTGTTCAAGGGAAAAAAATCACTGTCCTTAGCATGCTATTATTTTTCTAATGTTTCTATACAATCTTTGTCTCCACTAGTTGCATCTTAA |
| **CHROMATIN REMODELING 11 (CHR11):**  TCGCTCGAAGAGATGTGTTCAAATTCAACCCAAAAATATTACTAGTATTTTCTTTAAACTTCACCAGTCAAAATTCTGTAGTGCCCTTACAAGTGGGGGAGAACGCCACAATTTCAAGGAAGTCCCATCGGATTTTTTATGGCTCAAAAAATACTGTCGAACCAACTTTTCAATTGTGCTCATCTCACCAAGGGTTAAGTTAATTTCTTTTGCCGAGGTCAACAATGTACAGCTACATGTCAAATCCCTTCCCAATCTGAGGCATGCACATCACAACCATCCAACGCTGAAACACGATACCCCTGCACTTTCACGGAGGTCATTGACCTTTCATCGAACTAGAACGATTTATGTACCGACTTCAAGGTATCAATTGAAGTGCATATCGAAGGAGTGCGCTTATTTCAATAAGGATGCAACAGGCAACTCCAGGAACTAGATTTGGATTGAAAATGAGGTTGCATAATTATCCTTCAACAGTAAGGTGCCTGAGAGGTTATCCCCTATTCTTGAAATAACTTACAAAGCATCCATCTGAGATTCCAAACGTACCTCCACTTAACAATAAAATGCTGTGGCCACGTATTGAAGCTTGATAGATGCTGTTTGTTAGCGTAAATCCTGCTTCAACATATAAGCTGCTTACAAAACTTGAATGCAATAAACTACAGCAGCAATTTCTGACTGATTAAAAACATAGTGATTGACACTGCAATCAAATACTATGTTGATCCAAATACTATGTGCCCGCAAAGCACAGGAGCGTAATTTCTAGGAGGGCTTGCTGGAGATGTCAAAGTATTCAGCACAAGATTGTAAAATGCAAGTCGATGTAAGCAACATACTTCTAATAGATCAAAAAGGGCAAGTGACATTAGTAAGATTACTGCATATAGCCCAAACATGGCATGGCTACCCCATCCACCAAAAGAAAATCTTTGAATTTGATTTCCCCTCTTCCCCTTGGATTAATACTTCCAAGCAAAAGATGGGTTTTGACCATACATTCCTTCCATCAATGGCACACCACGAATTTGTGTAGGAGAGCCATTAATGATCCAAAAAATTACTTTTCCACAATTTTACTAATCTTTGGGGACCTCCATTCGTTCTGGACTAGTCCGCTTAAACCACACTGCGACCATCTATTGCCCCCTTCATGGACCACCAAAATGCATTCACAAGCGTCCAAGGGTAGCAGAAATGTAGCCAAATCTCCGGATCTTCATCAGATTCACTCGAAGCTTGCGAAGCAATTTAAAGAGCGATGAACCCCAGAATTTGCTGGACTAAACCTGGAAAACACAGGCAGCACATTTCAGCTTGCCTTTCCTCTCGTGGCTTCGGTTCCTCCAACATCATACCAGAGTTGAACTGAGCTTCATTCCACTCATCTCAAAACTTATCCTAATCAATGATTACCACCTCCTTAGCATTGATACACCTTGTTCCATTGAGTCCATTCAGGGTTTCCAGTGAAGGCTTCATCTCACGATCATCCCATGTGACTTCTGATTTGTGTACGTCCTCCAGGGACTGGCTAAACTGCTGCACTGTCTTCACTAAGAGCTTGTCATCCTGTAAATCCTCGATGCAAGGCTTAGATTCATGAACATGAGATAGATGCACGCTTCCCTCCGGCTTGTGAGATTCCACAGACGATAGTTTGGAGGTGGGCTTTATGTCTTTCGTCTCCAATGTATCCAAATCTGGCATGTCACCTGCCTGTGATCCAACAGCTGGGAACTCAAAGTTCCCATTCACTTGATGTTTTGCTAAATAAACGCAGTAACTACAGCAGTGTATGAAATAAGCCTGTGTAGGATGAATTTGCCCAAGATTCTCAAAACGTTCACTCGTCCCTATGAGTTCCGCCTCTGCTGGAAGGTCATCTTCACAATACGCATTAGGACACATTTCACACCGAAACAAGAGACCACCAACAGTGTGAGCCTTCCTCGCACAAATTCCACATGTATGATGAGGACAAAACCATTGCAACTTTGAAACCTGGGCTAGTTCAGCCTTGGAATAGCCAAGGCACTCTGAGTGGTATGCAGCTGGACACCCATCACAACAAATTAACAAACCCCCGTCTTTGCACAACAGACAAATATCATCATGTTCATAATCAATGCCAGCCCTTTGAGCTCTCTTTTTCTGAGTCTTGAATTCCTGGCTATCATTCAAGTATTCCCGTCCAAAAACACTTGGTTCACCTTCCTCCAAGTCGTACAGATTTTGACGACGAATAGCGAACCCATCCACTTCGACAGTTGTGGAAACTCGCTTGCGCTTACTAGCTTCTATCCATTCCTCCCCAATACCCTTGTTTGACGTTGGTGTATAGTCAACACCCTCAAAGACTCTAATGGCTGTAAATGGATCCATCACTACTGACTCTGTCTGCTGAATTTTTGAGCACTCCTGCTTTCCTATTTCTCCTTCCATGGTTGATGGGGAGCGACTTATTATCTTGTCCAGATCTTCGTCGGTCAACTCCTTGGTGTTTTCTGAACTTATGAAGGTCTTTGCTCCAAAGGTGATCGTAGCCAGCAATTCAAACAAGTTACCAGGTCCATTGTCCTCACTCTGACTCATACCACCATTGTTGACAGCATGATCAAGAAAGAGCTTTTTCTCAGACCGTTGTTGAATTCTCTCTTCCACTGTACCAGCTGTAATGAGTCGATACACATGCACAGGCTTTGTTTGCCCAATTCTGTGAACCCTAGCCATGGCTTGCAAGTCCACCTGCGGATTCCAATCACTGTCAAAAAGTATGACCGTATCTGCAGTCTGAAGGTTAATCCCAAGACCACCAGCCCTTGTAGACAGCAGAAAGATGAATATGGGACTAGAAGGCTTATTGAACATATGCATGTCAATAGTACGCCTTACGCGATTGGTGGAGCCATCCAATCGTCTGAAATCAAAGCCACGGTATTGACAATAATCTTCAAGAAGGTTCAGCATCGTTGTAAATTGAGAAAACAGCACCACTCTATGTCCGGTGCTTTGAAGCTTCAACAACAGACGATCCAACAACTGCATTTTTCCTGAGCTTGTAATAATGTCGTCGCCTACACAGCTTGGATCTTCCTCAGCTCCTGGAAATAAATACGGATGATTTGCACATTTTCGAAGCTGCATTAGCAAGCTCTGCAATTTTTTCCATGCATCCCCGTCCTTGCTTTTTTTGAAAAGTGCTGCTGATTCTGCCTCTAATTGAGATAAAAGTCTGCTATTTTTCAGAAGCAAGCGCCTATACCAAAAATGTTGCATTTTTGACAGAGGACTATAGATCTTCAATTCTGTTCGGGGGGGCAATGACTTCTCAACATCCACTTTAAGTCGCCTCAAACTCAAGTTCTTGAACAGCAAGTGCGCTTTGTCCAGAGTCTTAAAGTCACATTTGGATTGAACCATACTGTAAATGTTATCGAACAACTTTGGATCGGTAAAGATATCTGGAATAATCTGGGTAAAAAGGGCCCAAAGCTCATGCAGATTGTTCTGCAAAGGAGTTCCAGTGAGCAGCACAAGGCCCTGGCGATAGATGTGGCGAACTGCATCTGAGATCAATGCATTTTCATTTTTTACCTTATGTCCCTCGTCCAAAACTAAATATCGCCAATGAATTTTTGAGCGAAGAATCATCTTCATGTTTTGAGAAGCCACCATCTCATAAGTTGTCACAATAACATCAAAAGATGATGAGTTATTGAGCACCTCCCTGCGAAACCGTTCTCTCTCTGCCTTGTCTGATGAATGCAGCCGAATAACTCGAAGTTCCGGGCACCAATGCTGAAATTCTTTCATCCAAGATGATAAAACTGAAAGAGGACATACTACCAAGTGTGGACCTCCCACGTGCCTCTCAAACTTGAGGTACCCCAAAAATGCTATTGTTTGGAGAGTCTTTCCGAGCCCCATTTCATCTGCCAGAATAGCGCTGAGGCCATTGTCAAACATGGATACTAAAAATGAAAGACCTTCTAGTTGATAGTCTCGCATTTCTCCATTAACAATACACTTAGGTTGCCTTGTAATCTGTGTTTTTGGCATTGGTTGTTTGAAAGGAAGCTTCTTGACAGATTCCACCAGCTTGTTCTTCACATCAGATGTAATGAACGGCTCAAGTACATCAAGTATGCTAAACAAAAATACATTGGCTTTATTTCTGGCAACTATCATGTCTTCACTTATCTTCTGATTGTTGCTTCCCCATGCATTCTCATGTACCCTTTTCACAATTTGGTCAGCATCCTTCTTGCTTTGATTGCTGATTTTTCTTTTCTTCTCTGAGACCACTTGAACCTCTTCCTCTTGAGCTGCCCTATACACGCTAGGCTCACCTTCTTCTAATGTGTAGGTGTTCAGCCGCAGAACCGGTTGCCCGTTGACGTAGGTTATCCTGGACTTGCGAGAGCGGTCACTTCGTCTAGGTTCATCCATTGAGGTTTTTGTACAATCATCCATCACACTATCACCAACACCGGGCATGTGGGCGGGAAGAAAGAGGGCGTGCAGAGGAGAAGCGGAGGGCAGCACACCTGCGCGCTCTTTGCTTCTGAAAGGCCCCCCGGGCGGTGTAGGTGTGTGTATGCGCGCGCGCGCGT |
| **ACTIN-RELATED PROTEIN 7 (ARP7):**  TAAATTGAGCAACTAGTCCTCTCACTTCTCATTAGAACTAGAGTGTTATTGATGGAAGATCTGTTCATAAAATTATGTTGAAATGGTGGTTTTTTTTGCCACATGTTTCCCTTACAGCATAGTCAATCTCACAAATAATCAATGCAGCACATGAGTGAGACGTCCAACAAACACTCCGTACAATTTCATGGCGCATTTGCTCCACTACCTTTTCTTTAAAATTGGTCTAGCTGTTGCTTTGAATCAGAGAGCTTTTGATACACATGCGCTGATCGCAAATGAGAACTGAGACAAACTAACTTTAATAACTGCTCTTTGAACCCCATGTTACTCAAGGGACCTCATCTCAGTAAATGTCAAAAGATAACTATCTCCTCATTTAAAATTACTATATCCATACTTGAGCTTAGCAAACCTTTGTTGAGCAGCAAAAGCTTGGATGCAGCAGCCTTCCTTACAATCTGAAGTGTGAATCCGCAATCGTGCCATGCATAGTCCTGCTATCAATAGCACTTCTTGTGAACAATGCTTGGACCCAGCTCATCATACTCAGATTTTGTTATATGCTGGTTCTGAGGAAAAACGACCTTCGCAAGAATTGCGCCTCCCATCCATGCAGAATAGCGTAAAGTGTTTTCTGGCATATATTCAGGAGGCT |
| **HOMEOBOX PROTEIN 5 (HB5):**  TCTCTCTCTCTCTCTCTCTCTTGTGTGTGTGTGTGTATGTCTCTTTGTGCGCTCCACCTATGAAAACCATCTTCACACATCCTCTTTTGTAAGCTCTTGTTTTCTAATGAACCTCAGTTGCAGCCTCCTCTTTCCATAGCCAGTTTCTCCTTCTCCTCTGTCATTTCTTCTTCTCCCCTTGTGTGTCTGCAGAGCTGAAGTCCTCTCCATGGCTCTCTGTTTGGAGATCCCCATTGGCTTCAAGATGGCGAAGCAGGTTATGCATCTCCATACCTTCCTCTCCAGATATCTGCACCATGTCCCCCTCTTTGCTATCTTCCTTATGTGATAGCCTCTCCTCTGTGCAGGTTTCTCTCTCCTCCATAGCCCTCACCTTCCTCATCTCTCTGCGCCTCATTTCTCTCGCTTTTCTCCTCTTCTCTTTTCTCTCTGTACATTCGGTAGGAGCATACTTGCATAAGTTAACAGAACAATTGATTGAGGGGCCATGGAATGGAGCGCTGCATCTGGTTCTATGAAGGCGCTTCACGATGACCGCGATGATACTCTGCTAGGGCTTGTCTCTCCTGGGCACTCCTCAATAACTCAAGAACACAATCGCAGAGGAAGTAAGAAGCGATCTCACCACTACGCAGTAAAAGATGAAATTGATGAGGATGATTTGAGCGATGAGCTCTCAAACCAGGCCGAGAAGAAGCGCCGGCTCACTGTTGAGCAAGTGAAGTTTCTTGAGATGAGCTTCAACAAGGATCTTAAGCTTGAGCCTGAGAGGAAAGCCCTACTTGCGAAGCAGTTGGGCATACGTCCTCGCCAAGTTGCAATTTGGTTTCAGAATAGGCGAGCCCGTTGGAAGAATAAACAGCTTGAGCAGGACTATGAAACCTTGAAGGCCAAATGTGACTCTATTTTGAAAGAGAAAGAGATTATATTGCGGGAACATGAGGCAGTTATGGGTGAGAAAAAACGACTCCAGGCTCAGGTTGTTCGTCTGATGAGTTTGCTTGAAAGCCCAGGGGATAATGGCGTGCATGATGTAATGGGAGTGGGCTCGGAGGGTAAGTCAGAGCTTACGTCTCCTACCAAATCAATCGATGACCAAAGCGATATCCAAGATTCGGGGCAAAGCTATCCAGTATCAGAGGGAGTGGCTGGGGTCGACATGATTGGTCCATCACATGTAGAAATTGATCCTGTGGGAGCTTGGACTATGAATGTGAAGATGGAAAGCGAATTTACGCCTTCTTTAGCTGCTGATGATGTTTTCGGGAACATTCCGCCGCTCTTCCATCAAATTGCGGCTAATGCAATCTATCTAGAGGATGCATTCTTCTATAACTGTGAGGATCACTTTACTGGGTTGGCATATTATGGCTGATGACAGTAAGCCCTTTAGTACTATACTATTATGCCTAGATTAAGCATTATGCATATGAGAAGTAAAGCATTATGCATATGAGAAGTAGATATTTAGAATTATTCAGCCTATGGTCAACCCTTCAATTTTTGTCAGCACTTCTTGCAGGTTATGAATAAATGCGTGGTAGGTTGACATGATCAAATTCATGAAGATCACCTGCCACCATTTCCTCTATGACAATCTCTTACTGGTTAAGGCTATCCTGTAGAAAATATTGTACAATAGATGTAAATATTATTTCGGCTGACACAAGCTGGTTCTAATTAAGCAGGAAACAGTCTTGTTTCAGCAATATGCAAAATGAGAACAGGTCTTTGTATGTTCGCTGTGGCGCATTCTTGTTAGATTTAGTTGTTGTTGAAGCTGACATGAGATATTGAATGTGACATGAAACTATTTGATTGTGCTCATGATATCAATACGAAATGATATCACAACTCTTTGGGGGAGAAGATTGCTAGGCAGAGGTTTGTTGTTAAGGTTGTTCGGCTTTTGTGTAACATGGATGTGTAAAGTTTTTCACATAAGTCTTCAGTACTGAACAGCACTATGATGCTTGTAAGTTTATTACAG |
| **EMBRYONIC FACTOR1 (FAC1):**  GAAAAGACAAGCACTCACAACCTACCAAAAGGAGGAGACCGTCACCCACCTTAGAAGGTGACCGCTTTTTGAACAGAAGTGCGCCAGTTTTCTTAAGTTTTTAATTCCTTTAGTCTAGAAGATGGAGAGTCGAGAGTAGCTAGAGCTCACTCGCTACCTCGCACAGATTGAACATTGCCATTCTCCTCACTTTGAGGTTGCCATCAAACGAGCGATAGTTTATATCTACTAGCTTCGTTCACCTGCAAAAGAGCTTATCAAACAAAGGCTATGGATGCATCTACAAACTGGGACACTTTGGTCAGATTCCTAGCAACAGCTATCGTTGGTGCGGCGCTATTTGCTGTGCCCGCAGTTTTGTACCACAACCGGGTTGTGTATGAGCTTAAACACCAAAAAAAGCACCACGGAGCTGACGACACACGTCGCCATCAGGGTCCATTCAAAGCTTCACATCGTCGGGTTCGAAGCCGCTCATCAAGCAGAGAGAGAAGTTCTGCATCTCTCCGTGAGGTTGTCATGCTACATGAAAAAGATGGCCAAAAACCGCACCGGACCTCTAAGTGGCCTGAGCAAGATAATAGTGTGGATAGTAAACCTAGCTCGCAGTTGCCTTTCAATACACTGAGCTCAATACCGCCTGGGCTTCCACGGGTGCAAACACAACGGGAAGGTGCGAACAATCATCTGAGTCCAACCAGACGATTTTGTCCTTTATCAAGTACTGTACTTAGGCCTAACACACCAAAATCTCCAGTTGGTGCCAGTAGCTTCTCGGACGAGGAAGACATTGCAAATGACCAGGATGAGCAGGCCTTCGAATACGAAGAAGTGAATGAAGAGTCTCTTACCTCTAATGCAACCACGGTGAAAAAGGAAGAATGTGCAAAACCTTCTGCTGTGTTGGCCATAGACCAGTCAAATGGATCTGGTGAGCGAACAGGAAATGTTGTAAGTAGATCACACAGCATTCCTGGAGAGCTTCATGGCATTCATGCACCGGATCCGGTGGCAGCTGATATCCTCAGGAAAGAGCCAGAGCAAGAGACTTATGTACGTCTACAAATTGGGCCAATTGAGGCTCCATCTACAGAGGAGGAGGAGGTATGTAGAATGATGCAAGAGTGTCTTGCTTTGCGGCAAAAATATGTTTTCAGAGAGAGAGCTACACCTTGGGACAAAGAGCTGATAACAGATCCCAGTACTCCAAAGCGAAATCCAGATCCTTTCTACTACGAACAAGAGCCAGCTTCAAAGCATGTTTTCCAAATGATTGATGGGGTGGTTCATGTCTATGCCAATGCAGAAGCGGAGCAAGATCTTTTCCCTGTATTTTGTGCAACAGAGTTTTTCACTGATATGCATCGAATTCTGAAAATAATATCACTTGGGAATGTTCGCACACTTTGCCATCACCGGCTTCGTTTGCTAGAGCAGAAGTTTAGTCTACACCTGATGCTCAATGCTGATCGAGAGTTCTTGGCTCAAAAGAGTGCTCCACATCGAGATTTTTATAATGTTCGGAAAGTTGATACTCATGTACATCATTCCTCATGCATGAACCAGAAACATCTTCTACGATTCATCAAGTCTAAGCTGAGAAAGGAACCTGATGAGGTTGTAATTTTCCGGGATGGAAAGTACCTTACACTCAGGGAAGTCTTTGAAAGTCTGGATCTAACTGGGTATGACCTGAACGTGGACCTTCTGGACGTTCATGCGGATAAAAATACATTCCATCGCTTTGACAAATTCAATTTGAAGTATAATCCATGTGGTCAAAGCAGGCTAAGGGAAATCTTTTTGAAGCAGGATAATCTGATCCAAGGGCGTTATTTGGCTGAAGTAACTAAACAAGTTTTTCAAGATTTGGAACAAAGCAAATATCAGATGGCTGAATACAGAATATCAGTTTATGGCAGAAAACAAAGCGAATGGGACCAACTAGCTAGCTGGTTTGTCAACAATGAGCTTTACAGTGAGAATGTAGTGTGGCTTATTCAGCTTCCTCGTTTATACAACGTTTACAAGGAAATGGGAATAGCTCATTCATTTCAGAATATGCTGGACAATATTTTTATACCTTTATTTGAAGTGACCATTGATCCTGGATCTCACCCTCAGCTGCATGTTTTCCTTAAACAGGTGGTTGGTTTTGACTTGGTAGATGATGAGAGCAAGCCAGAACGTCGGCCCACAAAGCATATGCCATCTCCTCTCGCTTGGGATATTATTTTCAACCCTGCATTCTCATATTATGCATACTACATCTATGCAAATCTTTACACCCTAAACAAGCTTCGGGAAACCAAAGGTATGCCGATCATAAAATTTCGTCCTCATTCTGGTGAGGCTGGTGAGCAAGACCATTTGGCTGCCACCTTCTTACTTTCTCACAATATAGCACATGGTAACAATTTGCGCAAGTCGCCTGCTTTGCAATACTTGTACTACTTGGCTCAGATTGGGTTGTGCATGTCTCCTTTAAGCAACAATTCCCTATTTCTAGATTACCACAGAAATCCCTTTCCAATGTTCTTTGCTAGGGGCCTGAATGTATCTCTTTCCACTGATGATCCTCTTCAGATACATTTAACAAAAGAACCTCTTGTAGAGGAGTACAGCATAGCTGCCCAGGTGTGGAAGCTTAGTCCTTGTGACTTGTGTGAGATTGCTCGCAATTCAGTGTATCAATCAGGTTTCACACATGCTTTGAAGTCACATTGGGCTGGAAGCAACTACTACAAGCAAGGCCCTGAAGGGAATGATGTGCACAAGACTAATGTTCCGAATGTTCGGGTGGAGTTTCGGCATGAGGTGCTGAAGAATGAGTTACAGTATGTGTATCTTGGAGCAGCGCCAATTTCAGATGTGATACAACCATAGGGAGAAACCCTTTTTACGATATCTTGGTTTCAGAACTTGAAGGTTGTGGCATCCAATTTTGATATGCCTATGTTGGTGCTTGTAGTCTGTTTAAAACTTCTCGTCTGCAGAACTTGATCATGAAAAATGGCCAATTTTTATGTTTCATGGCCTTACGTGTGTTAAGATGAGAAAAAGTAAGCATTCATTTGTTCTAACTGGTTCTCCATGATGTTTAGAGAGAGAGAAAGAGAGTGGATAAAAAAAAGTCCTTATGCAACCTCTCTTTTATGCAATTGGGAAGTGGTTGCATCTTCTCTTTAGTGTATTCTCTCAGATTGGAAAGCATACATTGGTGTATGGCTTTTCCACACCTCTTGCTATTTCAAGAATCCCGTAAAGCCTGCTCACTCTCTTCAATAAGGATAATGAAAGTGGATGATGTTGTCAATAACGTACGCTATATTTTGACATTTTAGTGGAA |
| **GRAVITROPISM DEFECTIVE 2 (GRV2):**  TTTTGAGGAAAATAAATCAATTCACCTTTCATAAATCAATGTGTCAAAACTGTTACTTATGATTTACCATTTACAAAATGTCAAGCTTTACAAGCAGAACTGTGAGGGAAATTGAAGCCAACACCTACAACATGAAACTCATTTCCCAAATTTGGGAGATATATAACATTATTTACTGATACAAAAAGAGCTGGCCATTGCTGCCAGCTACCCAAACAAAATGCAAAAAGCACAGTTTGGGTCATTTATTGTCTTTTCAACAAGGATAATTAGCCATTTGCAATTACATATTTACAAGATGGATGATACACGATCGCAGAAAAAATCATTATCACAACCTAAAATGCACATATACCTTCCAAACAGGCATATATTCAACCTTAGAGGCTACCAATCTCTTCATCCGATTCCTCTGCAACAGCCTCCTGGAAAGGGCCATTGGTTATGGTCGAGCGAAGTAATGGCCGCGGGGTCGGAGCAGGAAGTGCATACGTCAAAGCCCCAGATGAACCCTCAATAAGGCCTGCAACCCCAGCTGCAGCAGACTGCGCATTTGTTGGAAGGAAGAGATCGTGTCTTTGATCCTTGTAAGCACTCCAAACATCTGATGCACTCAAAATGTCTTGAACTTTAGCTGAATGAGCTCCCTCACTGGCAAAAGCATGCAAGACCTCAACCGCCAAAACTCTTCCAACAGAGGCTTCAGACTCGTTCCATTTCATCTGTGCACAGAGTCCATTTGTACCACCTGATCGCCAATCAAGCAGTCCCAGAAGCACCTGCACTAACCCCACCTTCAACCCCTGTGCAACCAGAGCATCTCTTGCCCGATTTCCTGCAACCACTACTCTTTTCAAGGTCTCTAGAGCAAGCACACTGCCACCTTGCCATCCAATGGCTTTCATCAACAGTGGCACTACCTGAGGTGAACCTACTCCGGTGGTTGCCATGGCTTCAGCACAAGCAGTGCTAGCAGCCAGCTGATGCAAGACTCGCAAACAGCTTAACCTCACCCTTTCTTGGGGGATTTGACTTCCTGATATTTCTCCAGCATCCTCTGTAGTCAAGCACTCCTCTGCACGATCTCCCATACGTCCTTTTGCCCCTGACGACATTGTTTCTCTCCTGCTTTCATTTGCAGTTGATGCAACGAGCTTGGGAATATACCCTAAATGACCCACATGATCAGCTAATGTAGGATGCACTCTCAGAAGAGAGACTAGGGCTGCTGACAAGAGCAATGGGAGCTCAGGGTCTGCAAGAAGAGGTCCTTTTGCATGAGTTGCTGCAACTGCAGAGACATACTGGTCAAGTAGGCCCTCAAGAAATCGTTTTGGATTTCGCAGAGGAAACTTTGGGTCTTTAAGGAACAGTCTGACATAAACACCCCCGACCTGGGGTTCATCACGCATCTCCTGTTGGCCAGAACCTTGCTCAGGCAAGTCCCATTCAAGAACACTTCCCTTCACTTGCTCGCGATAAAGATTAGATGCCATGGTGGCGACCTGTGCACTGAGAGACGCTGCCATAGCAGGACTCCAGACAAGCTCAGGGGTTTCTGTAGTCTGTTCCAGGGCTGCAACTACCGCATCTCCAGGTCCATCCCGAATGGCAGAGACGAGCCCATCGGGAAGATACCTGGCAAGAGTTATTGCCACCCTAGGACCATGCATTGGCTGCCCAACAAGCTTACCCAAAAGAGATGCAGCAGCTGCTCGTTGTTGAAGGGGAATCTCTTCTTGCATGGGCAGCAAAAGTTGAAGTATATAAACAAATCCTCCATGTTTTGCAGTAACCCAAGCCAGCTCTGAAGTACTTGCCAACGCAGAAAGAACCCTTAAAGCCCCTGGCCGACATACCGGTGCACTATGTAAAAGTTTGAGTAGAAGCAGCAGGCTTGATCGATCAGCAACCATAGCCTCAACACTTGGGGCGTGCGTTGTCAAAACAGAGAGAATACTCAAACACAACTGCGGCACTTCCCCACTTGAAGCTTTTGTGTCCACGAGGCATTCAAAAAGTGGGATCAGATGCTCTTTGGAGCTAAAAGTTGAAGCCAGGCTTGGGGTGGCAGTCAGCAAATTCTGAAGTGCTGTTAGCCCAGTTGTGAGATCCCTTAAAACGATCTCTTCGTATGACAACTTATCATCAACTAGCTTGCTGTGTGCGTCTTTTCTTGACAGCTCTACTTCAGAACTTTCATCACCAGTATCAGCCTCTGACGATTCCTCAGATGAAAGTTCTGACTCATGGACCGGATCTAACTCTGCTGTCAGCTTTGCCAGATAAATTTTTTTCCTTTCATCTACCAACTTCGAAATAAATTGCAATAAAGAGTCGCAAAAGAGCTCCGGCTGACTGAGCTCATAATCAGGCTGCTCATTGTAGACTCGTAGGTATACTTCACCTACATGCAACTCTTTGGCCAGGGATTTATATTTAAATGACTCTGCTTCTTTCATTTCATATGAACCATCTTGACATTGAGAGACACGCTGTTTATCCACAAACTTCAAAAGCTCGCCCCTTGTTGAAGAATTCCAGATAATCTCTGGCGATTCCAAATTGGCATTAAGGTTGCAAAGAAGCTCCTTCAAAAGCTCTGTGGTCAGCATGGCTGCTAGTTTGGGAGTTAGCAGAGCTTTCAAGGCATTAGCTGCAGTCTCATTATGAGGTGTCGAGTATTCGTCTGTCAGGGATCCACGCAACCGACTGAAAGCACGTGCTGCAAGAAAAGCATGTGTATTTTTGGCAATTTGCACTCTGCTCCCTACACCATGGGACTCACTTGACCCATTTTCTTCAGCCGTTGAATCGTACTGCAAAAGAAGTGGAAGTAGGTACCAAAGTACCCCAGCCTTCAGCATGCAGTTTTGCAGCTCTTCTGATACAGCCACACTACCCATAGTGTGAAGGGCAGCTTCAACAGCTACAGGAGCATGCTCTAGCTCAGAGCAATGCACAACATCTTCTATAAATCCAGGACATCGAAGCATTTCTTTCCATGCACTCCTGAACTTGCTCAAGCCGGCAAACGTGCGCATGACATTTGTCACAATTTTTGCTGCAGGATCTGTGCCAGGGGTGGTACGCTGTACTATGTACATGCATCTCGACAGCAACGTGGCCAGTAAAGACACCCCGCCATCCCGTACTAGCTCCTCACCATTCAAAGACGACGATACACATGTTAGCCATACGAGCTCTGAAGCTGCAACAAGTAGTGGAGCCCTTTCTGTAGAAAGAAAATTGTTATCATCTTCATCAACTGTGATACAGTTGAAGAGCATTGAATACCCAGCATATTTGAATGGCTCTAAAACATTCCCATATCTTCTGTACAAAATGCATTGGCCTTTCAATAGGAGTAGTAGTCTCCAAGGCTGAGGCCCTTGGAGTCCTTGTAAGGTGGCCTGCAAACACTCATAAGCTTTTTGAACAGCAACAAACTTCTCTCTTCCTTCTGGGTTTTTGTCTGGATGATACTTCATGGCAAGCTTTCGGTATTGCCTCTTCAATGTTTCTTCATCAACACGGCCAGCCAGTCGATCTCCAGATTTTCCATCATCCACAACATGCTTAGCATCTTCATTATGCATGTCTTCTTCTAACGATATCTCCAATATTTTGCACGCTTGCTCCTCAGAAAGATCCATAGGTCTTCGTGTGAGCTCCTCGCGCCACATGGTAAGTAAGGACTGGAGAAACTCCACATGCTCTACAATGGGCCATTCAGGGAACCTGATTTCATCACACAGATTTCGAAGATAATAACGATGGCACCACATCTCATCCTGAAGCTCTGGATAAGTGACTGGAGGTATAGGTGCATAGTCATATAAAGAATGGCAATGTTGGGACAACTTCTGAGTAAAATCTCCAAGATGCTTAAGAACCTGATCAATGAGTCTCTCTGCCCTCATCTTGTGTGTCCATATTATTTCCGGAGTGTCTGAATCTGCAACCATTGCAGAAGCAAAGGATGCAGATCCAGATCTCTCAAGCACGTAAAGAAGACTCTCTGGCAAAAGTCCACCAAGTATGCTCCGCTTTGCTAACGGCAGTGATGAAGATAAAGCTGCCTCAGCACCTCCATGAAAGGCTTGGTGGGTATGAGTAGCTGCAAATAGGTTTGCCGCTGAATTCAGATTTGACCCAGCATAGGTTAACATGAAGTAGAAAGCACCCGTGTTATAAAGTCGGGCCATCGCTTTTGGATTCCTTGTTACCACAGCCTTCAAAAGAGATGCTGCACCCTCCACAATAACTGGCTCCCCTGTCAAGATGGCCTGTGCAACATGAGGAAGACAGCGAGAGCTGGACAAGATACGCTTCACCCTTGGAGTTGGAGTCACAAGTTCACCAGCATCATCAAGGTCTGAATGCACATTTACCATGCTGTGCAAAATTGACAAAGCAACCTCCCCAACCTGTGCTGGTGTGAGCACCGGAACTCTAGAAGCTAAAGCCCACCGTAATTCACGGATATCACTGAGCTTTCTCCACTCCAGCATCCCTGAAGCCCAACATTTGGTATCCCAATCAATTTCCTGCTTTGACCAAGCCCTCCTGATGCCATCCTTCTCCATCGGGCCAACTTGCACTCCATCCTTGCTCATGTAATGCCATTCTTTCTGTGGCTCCACAAAAGCTGTAGCTGCTATAAGATTTGACTGCAAAGGAATTGAGGTTCTTTCTGCCGTTTCGTGCGCTGTAGTCAAAAGATCTACAGCTAGTACACAACCTCCTACAGACACGCATGCCTCTGCATTAGAAGGCACCTTCATGAGCACCTTCAACAGCAGTAAAAGTCGATGTCGAAGCGCTCTATCACTTGTCCGATCCAAAAGAACTGTTATATGGGCAGTTCCATCAAAAGGGCCTATAACAGCATAATGCTGCTCATAGACGATTGTCATTGCTCTGGCACATAACTCCCTTACTGCAGATCCACCTCCTCCTCCAAACCCATCAAGCTTGCCCACATCACACCAATCCTCTAATGTACCCATTTCATCCCAATTTGCTCCATCCACTTGAAGTCCCATATCTGCATCACATAAGAAGCGGTGATATAGTGCGCTGAAAAAAGCAGCTGGATCCCTAAGAGGGAAATCCTGAGCCCGGCCACCCAGTCCCCCACCATCAAGTAAAAGCCTGAGATAATATTGTCCAACACAGACTTCCTTTGAAAGGCTTGGGTACCTGACCCCAAACTCCAGGTAGTTCCAAGAGAGTTGACTAGAGACCAGTTCTCCTTGTTTACTGTCACTAGTCCCATATCCCAAAATATTGACATCTTCTGTGCGTTCCTTCTCCACATCTAAATGGTGAACCTCAGTTTTCAATGCTTCTTTAAGCTCTTGTCGAGTGCGCTCATTCCAAATTAGATCTGCCCGACTATGATCAAGTCCAAACTCTCGCCAAAATTCTGGCCAGTTACAGAGAAGTCTGCCTGATCCAACAGGTGTGCTTTCCATCACCACTTGGGCAGGGGCTGGAGCACTAGAAGAGAACGGTAGACCCATGCTAGACTCACTTGCAAATGCATCAATAGAGTTTGGATTAGAGAAAGCAGTAAAATCTGAAGATAAGGGTGGAGTAGCCCCATCAGCTGTAACATTGTTCTCGAGATAAGATGGCCCTCCAGCTGGACTTGATGTTGGCATATTCATAACTGTGACACTATTGACTTGTATCTGTGCAGTTGGTGAAACGACTCCATTCTCAAATGCAGAACTGGAAAGAGGATTTGTCTCTTTCCGATTCGCAACTTCTGCTGCATTTTTTGCACGAAGCAACCTACCCCTTCTCCGCTGAAGAATGCGTCTCTGTCTTCGTTTCGCAGAAAAACTAACTTGAGTTTCCGCCTCTGGCACATCTTCCTCGTCCTCACAAGGAAGTGTATGCAAATATGCCACTAGACCAGGAGGAATTACTCGAGAAAGAAGCTCTAATGCTGGCTGGTAGGAATCTGCCCAGAGGGCTACAAGCTGCCTACTGACCTCACGCCTTTCACCAGGCAGTAGAAAAAAAGCATGTTCAAGATGTCTGAGCAGAGCACCATCACGCAAAGCAGCATCTCTCATGGGCTCTGCAGCAATTGCATCCTCCTCTGCAATTGTCCGCATAATCACCGCCACAGCCTCCCTTACACTTTCAGCAGGATGTCCAAACAAAGCAAATAATCTCCTCCGTAGTCCTGCTACTTGTCGTAACATCTCTACAAACATAGAGTGGTCAGTTGTCTCGCTCTGAGGTTCACAAAGCATTGCCTCCAGCACTTCTACAACGGCCATAGATAACAAAGGTGACACAGACAAAGGCTTGAGTCTATTTACTAAGATAGTTACATAACTTTGGTTCCCAAAAAGGGAAGACTTTGCATGCATCAAAGTTGCATGGCTCTCCCCCTTTGAATTAAGGACGCAACTGCTCTCTCCTGGGCCACCTCCAATAAGCACAGCAATCAAACTAGCACCTTCTGCTGCTACCCCCTCAGATCCACTCCGCAGTAAGCCCATAATTCGTTGTACAGCAATGGGCATAGAGATAACAACTGAAAATGCAGACGTAGAACCAAGAAGTCGCCTAAGGCAAGCAATGAGACCCATTAATGTTGCAGATGCTTTCGGTGATGGAGCAGGTGGTGGAGGTGCTTCTGGAGGCAAATTTGGTGGAGCTGGAAGCATTGATAATAAGGCCATGAGTGTAACATCTGGTACCTCAGAGCTGGCTTGCAGTCCACTGTAGGGAACACATGCATTGAATTCCCTGACCCTGCGCCAGAACTTTGCTTTTGATCCAGGTAGGGACCCACCTTCAGCAACAGCATCTTTCGCAGCTGTTGCAAGGTGTTTCAAATGTAGTACCTCACCTTCAATATCTGCAAAGAGACGGTGATGAGAAGAGCTTGCAGGTTGAGGCAGTGAAGGAACTTTGCCACATGGTGGATCAAGCCGATGACCTGGAAGGGTCAGTCTTGGCAAAACTGGCACAGGCCTTTGCCCCTCCATCTGAACAACATCTTGTACTGCAGCCAAGAGACTGTCACGAGATGTGCTGGAATAAACATGAATTGGGCAGCCATCATTGAACTCCAAAGCAAAGAGCTGTGGTTCTTCTACAAAGCGGACTAGAGCTGCGACTGAAGATAAAGGCCGTACAATGACTGCCTCGTAATTATCTGGCCTCCTTTCAACCAGTGATAGCTTTGTCAACACAAGCTGTCTTGGTACAGAATCACCATCCTCCCCAATTCCGCCTTTCCCTCCTTGCCCCAGTCCTGCACCTATATTGTGCACAGTCCCATGCCCTGCAGATCGAAGTCTTATAACCGACCATTCTCCATACCTTGTCTCCTCAGCTCCAACTGCCACCATAGCTCTTTGCTTCAGAAATTCCATTGCACCAATTGTTTGTGAGCTATCAATCATCAAAGTCAGGCCCATCTGTGTCTTTGCAACATTTGTCAACTTTGAAAGGATAGCTGAATTTGACACACCAGCGGCTGCTATAAACGCTTTGCATTTCCTGCCATAAAGAGGGCAAAAAACAAACCCACCTCCTTCTACGCCTTTTCTTCCATAAGCATCAGCAAGAAGAATAATACCAGGTGAATCCACATCCCGAAAATCGAGACACCAGTGCAAATCTCCTGTTTTTGGCTGTCTCACTTCCATACCGACAGCTGTAATGCGTAAAGTAAATGGGACCCAATTGGAGGTTCTCCGCCGCAAGTGAAGTACCAAGAATTCTGCCACAGCTGAGGTCGAAGGCTGATGCCCTAAACTTCTGCTGCCTTTCAGCCTATGGAGCTCTGATAAAATTGCAACGCGATACCTGGAAGAAAATTTGATGGCTTTGAACTTTCCACGGCCATCAGTTCTGACGTTGATGATAAACTCTTGTGCGTGCTGAAGCTGGGGATCATCCCTTCCAATGACAGGTGCTGCCGTTTCGAAATCGGTCAGGACATCGTATGAATTTGTGACGCTAAGTGTTGCAGGATCAAGGGTTATGATAGAAGCCTGAGATATGCAGAGGATCCGCTTGTATTTGCCACGCCAGGAGTGCTTGATGACCATATAACGGGCAAGGTACTCGGGTTCTTCTTGTACATGAGCATCTTGTGCAGGCGCGTTCTCGGATGGGGATGACATGGCTGGGCGATTGTTGACATAATCCATGGTCTGAAGGAGGTGGAGAGGGTGCAATGGGGGGCGCGCATTCGAGCGGAGAAGCCACCAGCTCCACGCCGCTTCCTTGCGGCCCCCCGACTGGCTTGCCCCCAGCTCTTCCTGCTGCTTTAAAACGGCGCTTTGCTTGCGCGCGGCGCTACCAATTTCTGCAGATGGCAACGAGCTCAGATCGCGCTATGGCTTCCCTTGAATTCACCTGCAGCTCTCTGCACACACACTCCACGCTATTGCACATGAAAACTGTCCCGTAGGGGAGAGAGAGAGAAAGAGAGAGAG |
| **TOPLESS (TPL):**  GCTAGAATCTTTATCCCATTGTCATTTGTAGTCACAGCTAGAAGAGAACCGTCCTTGTTGAACCAAAGCCGGGGACTAGCCTGCAATGAGGATGTGCATACTGCATTACACCCTTACTCAAGGGAAAGGCAAAAACACCACAGAACCTAGCACACCCTATGTGTTCCTTTGTTCAATAGTAATCTTACAGGCAATCCACCATCTGCATCGATGGTGGTAAGCAAATTAGTGTTATCCACATCCCAAAACTTAATTTGGAATTCATCGCCAGCAGCAAGAAAATGATTCCTGGTAGTATCAAACTGCACTACTCCCAAAGAACGCTTTCTGAAACCTGAATAGGTCCTCTTTATTGCTCCCTCACTTTCATTCCATTCCACTAGATAGGATTCACCTTCTTTGCTTGTCCCACATGAGAAAAGCCTGGTTCCATCAGCACTGTAAGCCATGGTAGTACACCATTGGCCTGGAGCGTCATAGTCTACCCTTGATCCCAAAAGATCATACAACCAAGCCTTGATTTTCCCATCAATTGCTGTCGAGAAAATAAACTGAATGCTTTCTTTATGATGAGGGCATACTGAGTATACAGGAGCCTCATGACCCTCAAAAGTGTATTGTTTGCGGCCATTAACAGCATCCCAAACCTTAATAGTTTTGTCATCTCCACAAGTGATAATGCAGAGTTGCTTGTTAGGATGAGAAAACGCAAGGTCATTTACACCACCAATATGGGCATCAATCTCAAGGTGTTGACGCAAATCATTTCCTCCCGCACATGAGTAAATATGGACCATGTGTTTGGAAAATGCAACCCCAAGTAGAGTACCGTCCGGACTCCATACACATCGATTGACGGAAACAGCAGGGTCTTTGACAAGCGCAGCCTGCATGGGCATGGTGCATACACTAAGGTCCCAGACTTTAAATGTTCTATGTGCTAGCCTTTCCTTTGTTCCAACATCCCAGATAGCAATATCACCAACATTTGTTCCAACAAGCAGTATGGTCTGCTGAATTGGATGAAAATCCATGCTCATTACACATGATCCTTGATTCAAAATTCGAGCCACCATCTTTGGCAATTCGTCAGGAGAGTACATGGGAGAGGGATGATTTGGGGCAGCTCCAACAGGGTAGGTCACCTCATCCACAGCTTGCCCTCCAGAGCGAACCCTCTTCATAATGTGTTCTGAATCTGCAGATTGGTAATCAAGAGCAGAATTATTTGCTGGTGGGGTCCTAGGTCGTTTCAGAAGGGCAGCAGCTCCTGGGTTGGAAGGGGCCGCAAGTGCAACGGACCCAGCTGCAACAGATGGGTGGGGTGCTGGCGGGTTTGAGTTTGCCATCCAACCAGCTATTGAATTTGCAGTCGGAGCTGACGCAGGGGGAAACGGTCCATGTGCCAAGGGAGGAAAGGCTCCCTGTTTGGGGAGCCCACCAACCAGCGAATTGCTAGCAGCAGGGGGAGCACGGGCTCCATTTTGAGGTCCACAAGAGTGATCTACAAACAATGTCTTAATATCCGGATTGGGTCGAGGGTTCTTGCACAGCTGATGTTGCCAATTCAGACTCTGGTTGATGAGAGTTCTTAATCGTGAAGCCTTCAGGGCAGGGAACACTAATTTGTCACGAAAAAGAGGATTGGCTTCAATAAGCTTTTTAAGTTCTAGTAGCATAATGCTCCTTGCAGACTTGGTATCCCCATACTTTGACAGCTGCTCATTCTCCCTAAAATTCTCTAGGGTCAAAAGTTGTGTGATCTCCTTATACAATTCCTCGTTGAAAGAGGAAAAAACCTTAAGATCCTTGACAAGTATATCCACAGCTTTAGCTCGATCTTGTTTGTCCAAAGCTTCAAGGTACTTTTGCTTTCGGATCTCAAAGAATATCTTCATTGAGTAGCGGTTATCATCCACTTTGGTGAAGCCTGACAAGTACCTTTCTACTTCTTCCCATTCCCCTGCCTGGACCTGATCCTCAAAATACTTCATGTTGAAGAAGAATCCGGACTCCTGCTCAAGCTTGTGTACAGTCTCCTTGAACTTCTCCTCATCCAGGAACTGCAGAATCAGGAAGACGAGCTCGCGGCTCAGCGACGACATCGTGCAACAATGTCCGCTACGGATCTTAACAGCAACGAATCAAAAGATGGGGGGTCAATACAATAAACAGAGCGGCAGGACCCATGTTGCTTGAGGGCCCCTTAAAAGGGCTTATGTTGTGTATTTGGGTTCCACTCTTTCATGGCCAAGCTCCAAAGCATAATGGATTTGTGGCTATGGCAGTCAAGGTATAATTCAGCACAAGGTAAAAGGAGAGATTGGCTATGGTCTTAATCTGCACAGAGGTAACACACGAGGATTCACTCAAAGACAAATGCGTAATTTGTGAAGGCAACAAGGGAGAAGGGCGGAGTTAGAAGATTTGGGAAACAAAAAAAAAACAGAGAAGTTAGCTATGGCGGGGAATTGCGCAGGGATAAGAGAGGACGGGAAGAAAGGCTTTAACAAAGAGGAAGCGAACAGGAGTGTAAGAAAGCGTAGCTGGCAGGCAGTAGTTAGCTATGGCGGGATGCTGCAGAGGGGGGGAAGCAGAGGAGAGAGAGAGAGAGAGAG |
| **UBIQUITIN-SPECIFIC PROTEASE 26 (UBP26):**  TGAACATGTACAAGAAAAATTAACAGTCAAAAGTTCTTTAGATGCTGCCACAGAAACCGAGGGGCAGTTTTTCTCCTATACCCCATAATACTCTAATGCACTTACATTTCACTCAAGTGCTGCATTGGCAAAAGATGGACCAACAAGATCAGGCAATCTGCTTAGCAGAGTGCCCCTAAAGCCTCCTTCCATCGAAACTCTGTCAGTGTCTTGCACATAAAATTCCTCTGCTATATCCCGGTTTTCATGCAGCCCCGTGTCAGTCACCCAGAGATGGGCTCCAGGTAAAATGTTCAGGTCAGACAAAGTGGCTGAATCATCAGTAAGCACTTTCTTACAAAAATGTAGACATTGATTTTCTTTCACAATTGAAAAAGATTCCCAAATCAGAAGCCTGAGCTGATAAACCGATGTAGCTCCAGAAACCTTGAGAGCAATCCGTTTGTTTGATGAAGTGGTCCCTCGTCGAGCTCGCTTAGATACGCGTCGCTCGCCTTTCAAGGGCTCAAGAAGTGCTTTCGGGGGCTCATCTCCATCCACAAAATCCACAAAGATTTCTTCATCAACATATTGAAGCTTCTTTATAAGTTCTGTACTCTCCCTTTCCTCGATACAAACATGACACACCTCTGGGTTTGTGAGGAGAGTCGGAATTTCAGTATCATTTTTACATCCTTCAGATACTTCCATTCCCACAGCAGCTTCTGTGGAGCTAGTGCTATTGCCATCTTGCATCGCTGGCATCACAACAATAGCTTTAATGAATTGGGAGTTATTCACATTCCATCGCCCACAAAGATCTATCCAGTTATCTTCCAATACAATTGTGAACATGTCATCATTTGGATTACCCTGCACAAGCTCACCACGCCGATTTCTCTGCAGTTGTGGGGGTTTGTAAAGAAGACCCTTATGCTTCTCACAAAGTAGATCTCGTAAGCTATTCTCAAGCCTGAGGGGTTCTTCAGCCTTCTGCGATTTCTTGCCACTGCCTCCCAAATAAGAACGCCATTGATGCAGCCACATTGAAGGCACAAGATAATATGTAGACCCAGGCACTATTGGAATGCTCCCTCCAGAAAACAGAGCTTCTTGTTTTTGTCTTTCTTCAATCTTTGTAGCCCTGAGATCTTGCTGTTGAGATGCAACTTCCATCATTTCTGCATCACAGATGGAGCAAGTAGGAGTATCTACTGGAAAGCTCATGCATCCTTCACCCAAATCTTCTTGAACTTGTTGAGCTACTTGAAAAAAATAGTCCCAAACCTCTTCAAGTACTGTCTGACGCTTTGCACCTGTAAAGTTATCAGGAAGTAAAGCCTTGTGAGGGCATGTTATAGCAGCAGTTGGAGATGCATCAACTTCTGTCGGGGCTTCTGCTGCTTTGCGTCGCAACCAATTTTGTAGCCAAGCTCTAGATACAAAAAAATGTCTTCCAGCTCCATTGGTAGCATCAGCATCTTCAAGCAGCTTTTTTATTTTCAATCTCTCATTTTTGAAGGAGTTGACTGATGCAATATTCTTGGCATTCTCCATGACGCACTCAACACAGCAATCGTATGCAGACAGCTCTGGTCCACCCCCATACTGAGATTGTAAGTTGGCCCAAGCTCCTTCAGATATACGCTTCATTGCACAAACATTGGATGGAGGTACTTTGCCATGTTCACATAGCAACTCGCTGTTGTCAATGGGAAAAGGTTTCAGCTCATCTGCCCATGATCGAAGCCAACTGCTAGATATCCAAAAGTATCCATTATCTTGATTTAGAGCTGGCAATTGTCCTGAAATTGTTTTTATCTCTTTCTGCCTATCTGCTCTGTCCGAAATCTCTTTATCTAGCTTTAGCTTGTACTCCCGGCATTTGTCTAACAGATCTTTATTTTGAGCATCTATCTTGCAGCGCAACTCGTCAGGCAGTTGAAAAGAGTCATCACTGGTGGATGCCGATGTTGCACCAAGTGGTTCTCTCAGGTTGTAGATCAACATATATGCATCAGCAGAGGTCAGACAAATCTCAAACTGATCAGGTGCTTCCTCGCGTGTTTCAGTGTCTGATGCCAGCATGGAATCTGGTGTGGCACTGCAAGATGCATCCTGATAGCTAGAAGCCTTTTCACTGCTGACATTAATTTCCTTTTTTGCTTCTTTTTTAAGAGCTTTTCCAGGGACTTCCCCACAGGGATGAGAACCCAAGCTTGTTACAAGCTCATCATCAAACTCCCACCACTCTCCTTTGACATCATTCTTGATGTTTGCAACATAATGGCCACTATTTGTTGTGCTTCCTTTGTGAAATAAAATACAAGAAAGATCATATAGCAGAGATTTTTCAGCACGTGGTCCACTTGATCCATCAATGCTAAGCCTGGAGCTCATGTCAAGCACTTGTGGAAAGCTAAATTTTGAGGTCACCTTCTTCTTCATTGCAGTCTTGGCATCAAATACAAAGCGCTTGAGCTGAAAATTTAGCACTGGTGGAAGAGACCTCAATTTGGTGAAGTGAGTTGCATCCACTCTTGCATTGCAAGCCTCGCATAAGAATTGATTTTCCCCAACCAGCTGCTCCACACTGAGGTAATCATCAAGGCTTCCATCCAAACTTGCCAACCCTTTCACATTGAGCTCTAGTTCATAAAAGTCCACAACTTTCTTTGAAGCGGGAGACTCCTGCCCGCAACTTGAACACCTAGTCACATGGGACAGGGTTCCTCGAAATACATCTTGCACGATTGTCTTCACAACAGCATGCCTAGAAAAATTCAACAAGCCTTCTAGCGAAGACAAAAGAAGCTTCAAAAATTCCTGTCCATCCTGCTGTATCGAGTTATTCAGCTCAAGCACCTCTGCAAATGGAGCAGAATCCACAGCTTTCTTTACCCCAAAGCGCAGCTCACCAAAAAGAAGTGCAAGCTTCTGAAGTACAGGCTGACTTTCAAGTAGTTCTGGTTCAGCAGCAAAGAATCCCCGCATAAACGGCTTGATTCTATACAAGCACTGCAAAACGCTGTTTACATAGCAAGTAGCCCCTAAATTTGTTAAGCCTGAAGGTGAGTCTATGGAGGGGTGAATCAGTTCTTGAGGGTTAGGACCGAGCATTGCCTCTGTATCAGATACTTTTTGCCATACCGTTCCACTTTTACGCGATCCTCCAGAAGGAATTAGTGCACAGAAACAATTTGGAGAGTCCTTGTTATTCACACGGCAACCTTCACAAGGTGGCTTATTGATCCGAAAAAGCTCCAAAATGTCCTCATGGCTAACCTCATTCTTCTCATCAATTTGTCTGAGAATGGATGTTTCAGGATCTTCAGCAGTATTGCGCTGCCTCCGGGTTTTGTGTCGGCTGCCTGCCCCGCTCATACTGTGCATGTGCAGCTTAGGATGAGCAGCTTCAATGGCGAGCTTCTGAATTGCAAACAAGTGCAGGCTTGCAAAGCAGAAATGGCAACTCCTAGCTTCTTCCCCAAAATGGACTCGAGTTTGTGTGTGTATCAAAGCAAAGCAGAAATGGTGTGTATGCATGCAATCATGTGTGTTTAAGGTTTTGCCTTTCAACAACAATTTGGTGATGTTTGAAACCTCAAACGCGCATCAATCTGAGCAGAAAACCTGTAAGCTTTCTTTGTTTGTAAAGAAAGGCGCCATGTAAAT |
| **YODA (YDA):**  TTTTTTTTGACTCAAAACACAATTTTAAAAAGAGTTGTATCGTTCGGATATTTATCACCGCCAACAATATCCAATACAGGTTACAAAAGTTCACATTTGCAAACTTTGTTATATGGTTCTGTCAGCAGCCCTTCTACAAAGGTTGCATTAACAAACTTTGATATACAATTGTTCAGAATCATGTCTTCATTGAGGCCCTTAAGTGGGATTTTACTGCATTACTGTGACCAGTCATCAAGACTGAAATGGGCAAAATGCTAAAATACAGCCGTGCTATGAATGCTGCTTTGCTCTTGAATCCGATATGTTAAGGGGACGGAGATGCATTGCTGCAAAAAAAAGAATGCTTTAAGACTCCTCGCCAACATCGAGCAGTAAGTGGAGTTCGCCGGTACGCACACAAATACTCAAAACTGCACACAATCAGCTTTCCAAGCCTCCACCATTACACATAAGTTAATCTGAACCACATGCTCATGAGAGCAAGGTAAATATCATGAGACCACCGAAGCCCTCCAGGCTTCCAAAAACTAGCCTGACAAGTTACTAGCAGCCTCGTAGAGTAACTCAGACGCCTTCTGTGCGTCTGCTCGAGAAGTTTGCAAGTTTGCTTCCCAACTCCATGTGTTACTCGGAGTTCTGCAACAATGATTGAAAGTTGCAGGTTCCCCTATCTCTGCCGCTCTTTGATCGTGACCATGGGCTTCATCAATTACAAAGTGATAGCTTCAAATCAGATAGCACACTATAGATTCACTTCATGACTGCCTGTACGACTGATTGCCGTTGAACCTGCGGGCGATGTCGGCCCATAATACCTTGGACTTTGCTGTGGCGATCTTTCACGAAGCACCTGAGTTACTCTCTCATCCATCCTTTGCAGAGCACCAGTACGCTTCTGCATATAGTCGTCCCTTTCTCTAGGGGGCCGGGCAGCCGCTGGCATATAAAACCCATTGTTACCTGGACCATAAATCTGTGGCATTTGCCTATTCTCTGCAATTATACCCCATTGAGCATCAGGTCCATTCACTGTAACATGTAGAGAGGCCGGGATACCATGCCGGACGAGCGGCCTTCCTGCCCTTCTATTGGCCAAATTTGAGTGATTTCCAGGTGTTGTATACAAGTCAACACCAGTGGTCGACAAACCTCCCGGAGCACCTGTTGGGGGGGTTGCAAATCCAGGAAGAAGACCAGCTGACCGTGTTGGAATCAACCCATATGATGACCGTGGAGAAACAGGAGCACTCACAGAAGGGAACATGCCTGGTCGGGCTGGTACATGTGATTCCCTCGTGTTTGAGAACTGAGTTCCTCCTGCTCTTGAAAACCTTCTTGAACTCCCTTCATTATCTAGCCGCATACTTCTCTCTCCAACATCCAGGAAATCAGGAATGTTCAAGGCATGGGCTAACATTTCAGGACCATCATTTGGGATTTGTACAAAAGGATGATCCAGTAAGAAGGCTGCAGAAGGACGGTCGGCTGGATTCCTTTGCAAACAATATCTGATGAACGACTGTCCTTCTGGTGAGAGGCCATCTGGAATGGGAGGAGTCTCTGCTCTGGTGACTTTAAACATGGCTGCTACCCCTTCATACTCGCTCCATGGAGGCTTCCCTGTGACCATCTCAATGACAGTACATCCAAGGCTCCATATGTCAACGGCAAACTCATGTCCAGTATTCTTCTGCATGATGACCTCTGGAGCCATCCAGTAAGGGCTTCCTTTAAAAGACAAAGGGACTCCTTGCTCCTTTATATGCTTTGCCATGCCGAAGTCAGCTAATTTTACTGTTCCATCCTGATCCACAAGTATGTTTGCCCCTTTTATATCCCTGTGAACGGTATGCTTGGTGTGCAAAAAATGTAACCCCTGCAAAATCTGTCGAGTATACCGCCTTATGACTGGCTCTTCAAACCTTTTAAATTCTTGATAAAGCTTGTAGATAGACCCCCCTGAAACAAGTTCCAAGTATATGTACAAATTTCCACCCACCATCTCACTCCCTTTATAATGGACAATGTTCTGATGTCGTAAAATACTTAAAAGCATTATCTCCTGCGCAAGCTGCTTTGCGGATTCTTTTGACTTAGGATCATCCCTCACAAACTCAACCTCCTTTATGGCACAAAATTCCCCTGTTTCACTATGAATTCCTTTATACACTTTGCCAAAAGACCCGCTACCTAGCAAACACCCTTTCAGCCATTTTCCAGCTGATGCCCCAGCCTCTGCTTTGCCAGGACTCCGAGGTGGTACTCCAGAAGAGCGTGGTGAAAGTGGTGTTGAAGGTGCAGTACCCCCACCTGACGTTGAAGGAAGTGGCAGTGGATGTCCATCACTCCAAGATGATGGATCATAAGCTGTACTATTGGACAGAGGAGGATGTCGAGGAGATACAGCCCCACTTTGAGCTCTTGGACTTAAATCTGAGCTTGGCCTTCTTTGCTGCAATGGAAAGTCACTTGATATTGAATTATGCCCTGAATTATGGCCAGAGGTGGGACTAGAGACCTGTTGAAGATGGTTCGATGCAGGGTTCCAAAGCCAAGGCTGCTGCTCTTGATGACCCATCCCCCGAGGGCTAAGCACTGGGCTGGAGAATGTACTAGTCGAAGCACTATTCTGAACTCCATTGACATGTATGTGGATGTGAAGATCGCCTCCTTCACCCTGACCGTTTCTGAATGCCAACCTAGGAGAAGCAATAGTACCAGCAGGGCTTCCCGGTTGCGTCCCATCAGCCCGTGCAGTAGATTGAATAGGGAAATGTGAAGTAAACCTTTGAGAGTCTTCAAATGGTGAGGAGTGCTGTAGCCTCAAAGCTTCCCCTTCAGAGTGCCTCCTGTAGGAAGTAGAGAGCTCATGACCAATGCCGCTTTCTGATGAGCTATCGCTTGATTCCGAATCCGTGGGATATACGGAAGCCTCCCTGCTATCATTGATTCGTGACTGCAGCGGAGTTCGAGCACAAGGTTGGATGGTTGGCTGTGCTACGCATGCATTCTGCCTTGGGGGGAGAGGCAAAGGTTTTGGGATATCTGTGCATCTAAAAGCTGAATTGGATGGAGATGGTGATTGTGAAACCCATGAAAATCCAGTGGCAACCTCTGCAGAGTTGTCACCATTGAATAGCGAGTTCCTTGACCTATCAACGCTATCATCATCCTCCTGGCGCCGATTACCTCGATTAATCCCGGATCTCGCTAGCCTGGGGGGATTCTGGATTGATGAATCCTGCTCTGACCTGCAACTTGACCTCTCTCTCCCACTCAGAGAGGAATCCTTCACACTATCGGTGCTTGCTTTCTTCCTTGTGTCCTTTGCTCGTCTGAAGAACGACGGAAACATCGGCATTTGCAAAGCGGTCTCAGCACTACATGTGCACACTGACGACCTAGAAACCTGAGAAGGATACCGGACCAGTGAAAAGCTGGGTTCAAAGAAAACCCTGATGCAGGAGGATTTTGAAGAAGCCTCAGATCGACTAAGTGAAACCTCTGAGTGAAGTCCTAGCTCCAGCACTGCCAGGTTGAGCACTCTGATAAACTCTTCACTATGTCAAAAGCCGTGCTGGTAATGACCAACCGAGTTAAGAGGAAGCGCGCTGTGTATTTCGATGTTTGTGAATGTAGAACAGACTAGCATTTTCGAGATCCAGCTGCATTTTGGGCACATTCCCTGTATTGTAGCTCCAACTCAACGATAAGATCATTGCTGTGGAGGAAGGAGAAGATAGCGTATGAGAATGGCAGCTCTGTGTGGAGGACCACTACAAGCTTGAAAGGCAGTTCGTGAGTTACCTATCCCTGCTCTCATGGGTCAACAATGCACTTTTCCGCAGTCTCTTGAACCACAAAGATGATGAAAGCTTGAAGGAACGTCTGTGTCAAAATTGCCCTGCCTCAAGCTCAGCACGCTTAGATCTTCTCCCAAAATTTAGTGCTAAAATGGCATAACCTGCTACACTCCTTGTAGAATCTGTGAAATCTTAACATTGGGTCTGAGAATCCATTGAAAATTTCCCGCAGCCTTTGCGTTGGCTAAGACTGGATTTTGTCTGCTATGAGTGCACGGCAAAGCCTGCTATACTTCTGGAGAAACCTGAGAAACCCTAAAAATTGAGTATGAGAATCTAAGGAGACCGTGACAAGGGATGAGTGTAGGCAGCTGTAATGTGGCCCAGCTGAAAAGAGCAAAAGGGCAAGGGGAGGGTAATAATCAGGCGAATTTTCCAGCACAGACAAGGTGGGCATCAAAGAGTGCAAATCACCAGTAATGCGGGAGAAAGCAAAAAAGCAGGAAAGCAAAAAAGAAAAGCTGCAGCCACAAGGCGAAATAGAGGGGAAGGGCATGCAGGGAAGGGGAAGCCCCTCAAGGCTCAGATGGGTTTCTTCTTCAATCTTCACCTCCTGGAAAGTGCAATGCTCATGACGATGGTGGGCCTCCTCTTCTCCCCCTCCTCCAGCTTGCACACTCTCCCGAGGGTCCAAGGCAAGCCTCACTTCACTACTAGTGCGCCCGTGTGTGTGTGTGTGTGT |
| **ABA-HYPERSENSITIVE GERMINATON 2 (AHG2):**  ACGACTGTTTGAAGCGTCAACTCTCGATTTGGCTTTGCAGCTCCACTAGAAGTGGACATTATAATGGCAAAGCTTTACATGTTTATACCGAGGCGTTAAAAAGGTTAGCTACTAAAAATTAGTAAGGAAATGAAATCAGGGGAAAAAATCAGCACAACATACAAAAGTCAATCAGCGATCAGAAGTTGAAAACTTTTTCACATGACATGAAAGTCTCTAATGTTCAGTTTTTTTAGGATGTTGTATCAGTAGGAAAAGAGGGCAGACAATGAGCTTCTTTGTATAAGCTGACCTGATTTTGCATCTATGTAAGCTGTGGGTTCCCGCACACACCGCTACCTGATTTGGTTTCCCACCACAACTGTTTCACAGTGCCCTCAAGTGCGCCGGCCATGTGCTGCAATGCTCCTTTTGGATACCTGGAGTATCTTGTGTCATGTTCCTAGCTAGGAAGGCATGGAGGCAGATGATGTGATACATCAGGTACCTTTACCCCTCAGCACAACAAACTGCTTTCATTACAGGAATCTGATCCAAATTCTTAGACCCACCCCCTGGCTCTTTCAACTTTCCTGTGCATAAATCCAGTATCCCTGGTCCAAAATTCAATAAATTTGTGAACTCTTCAACTTGAGAAGCTGTGGGAGTGTCATGGGCAGTTGAAAGAAGACCCTTGACATTAACATTCAGTTGATGGCAGATTTGGGCAAACACACAACCAGTCATGAAGGCATCATAACCGGCCTCATGTAAGAGGTTATTTTCTCTGTCTTGGTACCTCTTAAACGCAGGCATGATGTCTATATGAGCTTTACCAGAAAACTGGTTGGTAGCTCCCGCAAACCTTGCAGTTCCAACCCCAATACTCCTTGATTTGCACAGATAAGAATAAACAGCATTCAATGAGGTATGTTTCCTTTTCCAGGACGATTTAAGGCTTGGCACAAACTTCATAAGATATTTGGTATCAATAATGGTTGGAAAATGAGCTAAAAGAGAAGCTGAAAACTCTTCTGCAGAAGAAGGAAGCGGCGAGAGAAATTTATCATGTATGTGGGCTAAATCAAGAGCACAGTTATGTCCAATAAGAGGTTTTTGGGATGACACCATGGCATCAACAATGTGCCGAAAACCGATTGCCTCGTTGATCTTAGCTTGTTCACATCCTTGTTCTAGCTCACTCTTTATAATTTCAAAATATTCTTTGGACTGGGTGAAGAAAATCTTGATATCGTGACTTGCAGCAGAAACACAATCACTCCTTACAGTAAAAACAAGGTCCTTAAAATGTTTGTCAAGCACCAGTTGTGCAAGACAAACTTGGTGGTGATTCTCCAGCTGCAAAGTCAAAGAAGGGCACTTGCTCAATACACCACTATGTGGCACCTTAAAATCTTTAATCGGACATCTATTCAACAGCTTGAAGTTAAAAATATCTGCCGCTAGGTCACTGCCATGGGAATTCACATCCAAACTGCAGGTACTTATCTTGCTGTGCCACTTCTCTAAATCCACCCGTAGTCGTTCAGCAAACACTACATCTGACGTGTTGTTTAAACTTCTACTACATCCACAACTCTTCAGTCTAAAAGGAACATGTTCATTGGCCAGCTGTACCCTTGCTTCAGATTCTTGTGCCTTAGACAAATATGATATACCATCCTGTATGCACAAGTTGAAGTCAAAACCATGAGCTGCTAAAAAATCCAAAGCAGCAGCCTCGCAAGAAAACGTTGCAGCAGGTAGACCAAGAGAAAGCTCATTTCGAGGGAACACATAAAAATTGTACGGGTAAGCGAGGAACCGGTTGCAGGAAGGGTCAAAATGGAAGAGGGCGAGGCCGACCTGGAAGACAGCGAACTTCTCAGCGAGGTGCTTGATTCTGTGATATGACCGGAGGAGGGGAGATGGGGGGGCTGCATGCGAGCTTTCAGGGGGCACACTGTGAATGCCACTAAGCTCCAAGTCGAAGGCAACAAAATCTGCAGCGCCCAAATGCGCCTTGAAGCTGTCAAGCGAACTCCAAAAATTCTGCCTTGATATGTTGCCCTCCTCTGCTTGTACTTGCCATCTCCCTTTCTGTTCTTCTAGTTGATGGGTGCTGTTCCCCTGCTGTTTTGCTTGTTGATGGGAGCTAAAGGCTCGCGCGCTTCCAATAGTCCTCCTCGCACTCCCCCGCCTCAGCCAATTCATTCTTTGAAATTCATACCTGCCTCCTGACTGCCCTTTGCTATTTACTACTACTAGCTGGCAATTTACCACGGGGAAACATGGTGTT |
| **PLETHORA 2 (PLT2):**  ACACACACACACACACAATCTTAGAACAAGGCAACGAATGTTCTTACTGTAAGAGCATGCTTTAATCATGGATGGGGCTGCGATAAAATCTGTCGATGAAATGTCCGCTACAAGTAGCGATGTCTCCAATTGCACAGGAGATGAAAGAGAAGCTTTCATGAGATCTTATGGTGAAGAGATAAACCAAGGTCACCCAATGCTTGAGGACTTTCTTGGGAGCAAAACCAAAGCACACCAGGTGTTTGACAATGGCAGTTCTATTAATACGCATGCATTGCGCGGTGTAAATGAGGGAGGCTTCCTGCCGTCTTCCCTCAAGCACTCTGTGCTAAGTGTTTACAAAGACTCGAAGACGATAAACGATGGATTACAAGGGAGCTCTGCTATTGATAGTGGCAGACTGAAGAGGGCTTTAGGTTGCTTCAAGGATAGTGTAGCAAATGAGGGTATGCATCAACAGTATCAATACAATGGGGCAACTTTTGCTAACCCCACTTTATTGACATTAACACAACCAATTACTGGTCTTGATTCAACTAATCATCCATTTTCATACTACAAAGCGAGAGATCGCGGCGCAAATGAACTTCAAAGCATATTCCAATATTCTGCTACTGATAGCTCTGAAAGCACACTACAGCAAAGCAACCCTGGTTTGAGCCTAGAAGACTGCAATTTGGGGAGGGCCATCCTCTACCCTAATTGGAGAACTAATAACATTGCAGCAAATCTACATGATATGGGGAATTTAAGAGAAAACTACTCACATTCTTATAATAGAGAGGGGGAATGCATGGCAAATGGGGATTGTAATATTGGAGGCAATAGGCCTAATACTAATGGGTGCTTACAAGGAGGTTTTCCTAATAATTTTAATTTTAACAAGTTGCAGGGTACTTCAATAGCCCCTAGTGCTACTCTGGGGCATTCCCTCTCACTCTCTCACTTCCCATTCAATCACCAATCGGCTCTCTCTCTCTCTCGAGACGCTGCTAATTCTGCATTAAAGAGTAGTAATATCATGATCAACCCTCAATGTGCTGATCAATATAAGGTTGATTACAGCAATATACAACCTGCAGAACCAATGAAGCTTTCCATGAGAAATTGCAGTAGTCACTTTGATGCAGCAGGAACAAAGAGAGCAGATCATCTGCTATGGCATCATCTTCCTCAGCAAGAAGGCTTTGCAGCATGCGCTGCAAATAGTGAAAGCATAGAAAGCACTGTTAATATGAAGGCAGTTAGTGATCAAGATGATGATCCATGTAAGGGTAAGACTGTAGGACCTGCTGCTGCTGCAGGTTTAATGGAAAGCTCCATTCCAAACAGTAATGATGATCACTTGCAGCTTCAGCTAGTTCATTCAAGCGCTGAGCAGAAGATATTGCAGCATAATAATAGTGCTTGCAGTCTTGATGCTGGTAATGCGGAGTCCACTTCTCGCAAATCAATCGAAACCTTTGGCCAGCGCACTTCTGTCTATCGAGGGGTGACCAAACACCGTTGGACAGGAAGATTTGAAGCCCATCTTTGGGATAACAGCTGCAGAAGAGAGGGCCAAACCAGAAAAGGAAGGCAAGTCTATCTGGGTGGGTATGACCTGGAAGAGAAGGCGGCTAGAGCATACGACCTTGCAGCTCTAAAATATTGGGGTCCAACAACAACCATCAATTTTCCGCTTGAGAACTATGAAAAGGAGATGGAGGAAATGAAAAGTATGACCAAACAAGAGTTTGTTGCATCTCTAAGAAGGAAAAGTAGCGGGTTTTCAAGAGGTGCTTCCATTTACCGTGGTGTAACAAGGCATCATCAGCACGGACGGTGGCAAGCTCGGATTGGAAGGGTGGCTGGGAACAAAGACTTATATCTAGGCACATTTAGTACACAAGAAGAGGCTGCTGAAGCATACGACATAGCCGCCATAAAATTTAGAGGATTGAATGCAGTGACAAATTTTGACATGAATAAGTATGACATTGAGAAAATATGCTCCAACAATTTGCTGCCTATACAATCAATGAGAAGGGTAAAAGAAAATGTGGGGGCAGTTGATTTAATGCAGGGGGAGGATGCAAAAGGCAGTAATGCCATTGGATTAAGTGCTCATTTAGCATATGGAAATGGAAGCTTTGATATTGCAGCAAGGGCAGGGAGTATACAAGAGTGGCAGTTAATTAATCAGCTCGCTCAGCCAGCTAATCCATTATACCATGATAATTCCTTGTTGATGTGGAGCAAAATGGATCAAGAGAGAGCAAAATATGCACTCCACACAGGCTTGCTCTTCCATCACAGTAATAACTTAAAGTTCCTTGAGGGAACTTATAGTTCTCTAATGCAAGGAGCTACAAACTTTCCTCATGTGGAAAGCTCTCCTCACTCTATGCAGACTCAAGGTGCGCCTGATTTGGAAAGGCCTCCTTTTATGGGTGCGACACAATCGTGCAGCAGATATAATTTTGGGGGTGGCCAGAGCCACTTGGCAGAAGAACCCTCACATGTTTGCGAACAAGTACAAGCTCCTGAGTGTGTAAGTTTGCAGTTGTAAGAGCATATTCCCACGCTCTTTCATTTTAGTATGTCTGACATTAAAAATACTCCACATACAGGATCATTGCAATTTTGCACTCTTACTTTAAAGCCCATTTTGCTGTTTTAGGCTAAGTGTCACATTTTCCATGTTGGTGCAAAGTTACTTGAAGCCTCTACATGTTTATG |
| **WUSCHEL RELATED HOMEOBOX 8 (WOX8):**  CACACACACACACATATATATATACACAGATACCTGCAAGCACGCCATCGCCATACGCTTCCGTTCATTCTTAGCAGCCGCAGACCTCATCTCCCCTGACTCAAATTCAAGCAAGCTCTAGTTTTCAGCGGCATCAAAAAGTCAGCGGCACAGTCGCAGCCATTAGATCCAGCCAGTAGGGGACTACAGCCGTTGGATCGTGTCTAAGGAGTTTAATTAGCGTGGTGCGTACGAGGGGGACTTGTTTGACGCTGCAGCTGGGCGGTGCTGGCGCTGCAGAAGCTCTCTCTGCTCTCACTTTCAAATACAGAGATTCATGTAGTAGTACTCCCACCACCATCTCTGCAGAAAACGCAAGCTCCTTCAAGCACCCATTTTTTCATCCTCATCATTGTCTGCTGCAGCTTTCTTCTTATAATTATTCGACCCGTGATCATTATATGGCGGCTCCTAACTCAGAGAGCGCATTGAATTTAAACCCAAGGAATCAATCTATACAAGCCGCAGTCAATGCAGGATATCTAAGTAGAGAAGAGGCGCAGCTGCTAATTATGCAAGATCCCGGTAATTATGCCACACATGCGCTACCGTTACAAGGGCTGTCCATGAATAATATGCAGGCGCAGTTCTATAATGAGCAGATCCTGCGTTCTATGAGTGGGTCGCACTTCACGGGAATAGAACCCACAGAAAATCAAGAGCTGATCAACGTAATGCAAGCACAGCAGAGCAGCTCGACATCTAATGCAGGGTTGCAAAACCTATTAAGCATGGAGAACGTTGGCAGATCGGTTTCAAATGCGCAGGCTGCGCCAGTGCGTTACGAGGAGACATGCACACCAAGGCCAAGGTGGAGTCCCACGCCAGAGCAGATTGAGATTCTGGAGGCACTTTTCAACTCCGGAACAAGGACCCCGTCACGCGATATGATAGCGGACATCGCGGTGCAGCTGCAAAAATATGGCAACATCGCAGAAGCGAATGTCTTTTACTGGTTTCAGAACCGCAAAGCGCGAGCCAAGAGGAAGCAGCTGCAGCCAGCGGCATCAAGGTCATCAAGCAGCTGCCCCTAACTTCTAATGGCAGCTCAGGCTATCAGATATTATATAATCCTTTTATTTTCTTAGTTGTCCTGCTTGTCCTAACCATAAGGCTAACTAACTAGACTTAGCCTAGTACCGGTTACCGGGATGTCCGCTTTGCCTCGTACGCGGCCAAGCGGACCGATAACTAGCAAAATCCAAGAAACTAGTCCATCTCTTGTCTATTATATCACCTTCTCTTCTCTTTTATCCAAACTAAAATTTTGGTTCTTGCTTCAACAAGACTGTTTATGTCCTCCACCTCAATACATTCTAACATAGCAATGTGGATCACATTTTGTACCCCCAAGTCTTAAAAAAAAAATGACCAATCTTTTTATAGCATATATGTTTTTTGA |
| **MATERNAL EFFECT EMBRYO ARREST 5 (MEE5):**  GCGGTTTTGAACCCACGAACATTTCTATTTTCAAAATCAAAACCCAGCGCGACTGTGCAGACGAGAAGAGGAGAAGAGGCTTGCAATCATGGACGACACACTGTATGACGAGTTCGGGAACTACATAGGCCCGGAGCTCGACTCAGACGAGCAGGATTCCGAAGCTGATGATGAAGACGACGGAGAAGAGGAGGAGGAAGCAGAAGAAGCTGATGGGCACAGCCGCAGCCGCTTTCCGGACGGCAACTCCGGTGATGGCGCCGACAACGGCTACGGTGCCGACCTTGCTCTCGATGCCATGGACCTTGACACCAGTAACGGCATTGTACTTGCAGAGGACCGCAAATACTACCCCACTGCTATGGAAGTCTACGGCGAGTCTGTTGAGGCCCTTGTCATGGATGAAGATGCCCAGCCCCTTGAACAACCCATCATCAAACCTGTCAAAACCAAGAAATTTGAAGTGGGTTTCAAAGAGGGTAGCATGCTGTCGCATGTTTCCACTGAATTCCTTCTTGTCCTCATGTCGAACCCTGTGCTCGTGCGCAATGTTGCCCTTGTTGGGCACCTTCACCATGGAAAAACCCTCTTCATGGACATGCTTGTCGAGCAGACGCACGAGATGAAGACTCTCGATCCTAACAGCGAGAAGCACCTCAGGTACACGGACACACGCATCGATGAACAAGAGCGGCAGATCTCCATCAAGGCTGTGCCCATGTCCTTGGTTCTCGAAGACAGCAACAAGAAGTCTTTTCTTTGCAATATCATGGACACACCCGGGCATGTGAATTTTTCAGACGAAATGACTGCTGCGCTGAGGCTTGCAGATGGTGCTGTACTTATTGTAGATGCTGTCGAAGGTGTCATGGTAAACACAGAGAGGGCAATTAAACATGCTATACAGGACAGAATTCCCATTGTGGTGGTTGTAAATAAGGTTGACAGGCTAATTACTGAGCTAAAGTTGCCACCTACGGATGCATACCATAAGCTGCGGCATACAATTGAGGAAGTCAATCAATCGATTGCCAGTTTCTCTACAGGGGCAGATGATCCTCAGGTTGTTGATCCGATTCTCGGAAATGTGTGCTTTGCAAGCGCAACTGCAGGATGGTCTTTTACTCTCTTATCATTTGCCAAGTTGTATGTCAAGCTGCACGGCATCCCATTTGATGTAGAACAATTTGCTTCTAGACTCTGGGGGGATTGGTATTTTCATCCTGACAGAAGCTTTAAAAAGAAAGCTCCTCCGGGTGGAGAGAGAACATTTGTCCAGTTCATTCTTGAGCCGCTTTACAAGCTGTATAGTCAAGTGATTGGAGAACACAGGAAAAATGTGGAAACAACCTTGGCTGAATTAGGGGTCACACTGAGCAATGCAGCGTACAAGTTGAATGTGAAGCCTTTGTTAAAACTTGCTTGCAGCTCGGTATTTGGTTCAGCCACAGGATTCACTGACATGTTGGTTCGTCACATACCGTCTGCGAAAAAAGCAGCTATCAACAAGGTCAATTACAGCTACACAGGTCCTCAAGATACAGCTCTTGCCGATTCTATGCGAGCTTGTGATTCGAAGGGGCCGCTGATGGTCAATGTCACAAAGCTTTATCCTAAATCCGACTGTAGCGTGTTTGATTCTTTTGGAAGAGTTCTGAGTGGAACCCTTCGTACAGGCCAGACTGTAAGGGTTCTAGGTGAGGGTTACTCTCCAGATGACGAAGAAGATATGGCGGTTAAAGAAGTCACAAAACTGTGGGTTTATCAGGCAAGATACCGCATACCTATAAGTCAAGCTCCAGCTGGTTCATGGGTTCTTATTGAAGGAGTGGATGCCTCTATAACAAAGACAGCAACGCTGTGCCCGGAATTTTCTGATGAAGATGTTTATATTTTTCGGCCGTTAAAGTTCAATACCTTGTCAGTAGTGAAAACAGCCACAGAACCACTTAATCCTAGTGAGCTGCCAAAGATGGTAGAAGGCCTCCGAAAGATCAGCAAAAGCTATCCGTTGGCTATAACAAAAGTTGAGGAATCAGGAGAGCACACCATTCTTGGTACTGGAGAGATATACCTGGACTCTATAATGAAAGATCTCAGAGAGCTTTACTCTGAAGTGGAAGTTAAGGTGGCAGATCCTGTAGTATCTTTTTGCGAAACGGTCGTAGAAACATCGTCACTGAAATGTTTTGCAGAGACTCCAAACAAGAAGAATAAGATTACAATGATTGCCGAACCTTTAGAGAAGGGCCTTGCTGAGGACATAGAGAACGGTGTGGTCAGTATAGATTGGCCTCGTAAAAAGTTGGGGGATTTTTTCCAGGCAAGGTATGACTGGGACGTACTTGCTGCTCGTTCAATATGGGCGTTTGGTCCAGACAAGCAGGGTCCAAACATTCTTCTGGACGACACTTTACCCAGTGAAGTTGACAAAGGACTTCTTAATGCAGTAAAAGACTCCATTGTCCAAGGATTTCAGTGGGGTGCTCGTGAAGGCCCTCTTTGCGATGAACCCATCCGAAATGTAAAATTCAAGATTTTGGACGCAACTATTGCTCAAGAGCCTCTTCATCGCGGAGGTGGCCAGATAATTCCTACGTCTAGAAGAGTTGCATATTCTGCATTTCTCATGGCGACCCCTCGGCTAATGGAGCCATTCTATTACGTGGAGATACAAACACCAACTGATTGTCTTACAGCGATTTATACAGTGCTTTCTAAACGCCGTGGGCATCCAACTGCAGATGTTCCCAAGCCTGGAACACCTGCGTATATTGTGAAGGCATATGTACCAGTAATAGAGTCTTTTGGGTTTGAGACTGACTTGCGGTATCATACACAAGGCCAGGCTTTTTGCTTGTCTGTTTTTGACCATTGGTCAATAGTGCCTGGAGATCCTTTGGATAAGTCTGTCACCTTACGACCTCTGGAACCTGCTCCTGCACAGCACCTGGCTCGAGAGTTCATGGTCAAAACTAGACGCAGAAAGGGAATGAGCGAGGATGTAAGCATCAATAAGTTTTTTGATGATCCGATGCTTCTTGAGCTCGCTCGACAGGATGCTGATCTTCAGCAGATTCTTTGATTTCCTCGGGAGGCTAGTATTGGCATCTGCACCCTTACCTATATGGCACTTAAATTTGGACCATGTCTATCCCAGCAAACCACACGGGCAAGTGAGGGCCGGCACAGACAGCGTGCTTGTTGTCAAACATGAATGCAAAAAGTCCTCACAAATCAGTACCGTAGAGCTTGTGCAGTTAACCACAGAGTTAGTGGCTTTTTGACATCAGGCTGATATTACAGTAGTGGCCATTTTGGAAGAGGCATGCCTTTTTGAGAAGTGGTACATTCAGAGTCTTCTTGACAAGTGCACAAGCCGAGATGTGAGTGTATACAAATTTTTTTAGAGGGTATCCACACAAGTTTACAAGTGATGATAGGCTGAATTGCATTGATTGCAAGGCACTAGTCAGCACTCATGACTGCTTCACTTTAGCCGGCTTAACCATATTAAGAAGTTGGGCAAGTTTGTCCACTTGAGCGTTTGCAGACTTAACCAGAGGGCTTTTGGCTCTCAGTGTGCGCGAGCATCGATTCCTCTGCAGCTTGCAATGATAGGAGCCTTGCTGGAATCATTTTCGTAATCTTAGGCTTTGTAGTTCACATGAAAGGGGTCTGAAGCTAGTCAAGGGCTCAAACCATAAAGAGCTCCAATGTAAACGATTATAATGAGCATCTTCTCGTTTATCCTGCTTAGCTTCTCATTAATCTAGATCTAAACCTCAAACTGTTTTCTCTGGAAGCTGAATTGTTAATTCAGCATCTTTGCACCAATCTCCTGTGAGGTGGTGAAGTTCTTATACTTCCAAGTTTTACTTAAGTTTAGGAAGTTCTAGCCCCTGAATGTGTAATTTAATTGGCAGATGTATCGTCTAAATATAAATGGTGTGTTATGTATCTGTATATGTAACTTAAAAACAGGCATTCTGGGATTGCATCCGAACAGATTCTAGCTTGATGGCCACAAGTCAGGAAGCGGTCTG |
| **MATERNAL EFFECT EMBRYO ARREST 63 (MEE63):**  TCATGTACATATCTATGTATGTAAATATAATATATAGATATAAATATATATGTGTTAGGTGCTGTTGGTTTAAAGTATAGGGCTAAATTCGTTACAAGCGCGGAGCCATGGCGGACTCGGATTTGGGAGAATTCTATGTACGCTACTACGTGGGGCACAAGGGCAAGTTCGGGCACGAGTTCCTGGAGTTCGAGTTCCGCCCTGATGGCAAGCTGCGCTACGCCAACAACTCCAACTACAAGAACGACACCATGATCCGCAAAGAAGTCTTCCTCACGCAGGCTGCCCTACGTGAGTGCCGCCGCATCATTGCCGACAGTGAGATTTTGAAAGAGGATGACAATAACTGGCCAGAGCCCGATAGAGTCGGCTCTCAAGAACTCGAAATTGTGATGGGAAATGAGCACATCTCTTTTACGACCGCAAAAATTGGCTCCCTTCTTGATGTTCAAAGTAGCAAGGACCCAGAGGGACTTCGCATCTTCTACTATTTGGTGCAGGACTTGAAATGTTTTGTATTCTCCCTCATTGGCTTGCATTTTAAAATAAAGCCAATCTAGATGTTTTTTGGAAGAGCACCTACATGGTTTCAAATAGCAGCTATATGGAAAAGATTTTATCTTTTGCAGTGGCTGTGAGCACCTTTTTATATCATTGGTGTGAGCATTTAGGCAGAGTCTTGCAACTGGCTTCATAGGAACTTGCTTTCCATGTCAATAGTTTTTGGGCTCCTTTTTATCGTTATAAACTCTCGCTTCATGACAAGAGACACGACTTTTATATTGCTGGCAGGATTATATGCAGATTATTGTCACAAAGCTCGTTATGTGAGTAATACAGAGCCACAATTCCGGGTGTGTCAACATAGTTAACAAATTATTTTGAGGTCAGATTGGTGTAATTTGAGTGCATATGAGAGCAATAGGTTACTATCTGTTAGTTTTTTTGCATCTATTTGATATATTGTGATTTCCAGTCAAGGTTGGTTTAAATCAGGGCTGGCATTTTGGGTTTGTTTTAATGCCTTG |
| **MATERNAL EFFECT EMBRYO ARREST 58 (MEE58):**  GTGAAGAGGCTTGTAGGGAAGGACAAGCAAGATCTTTAAAATGGTTTTTCACCAGCAGAACCCTACGCCGGTGTTTCTTGCTCGCCATAGCCAGGAAGCCCGTAGCGACTGACGAGGAAGACGCACACAGCCCTCCGTTTCGAGCTCGGCGTCCTTCATTTCTGCGCCTATCTTGCTAGCAGAGATGGAGCTCTCCGTGCAGAAAGGTGCTAAGGGTTTTGAATACAAGGTGAAAGACTTGTCGCAAGCTGATTTTGGCCGTCTGGAAATCGAGCTCGCTGAGGTTGAGATGCCTGGCTTGATGTCTTGCCGCACCGAGTTTGGTCCGTCGCAAGCGTTGAAGGGAGCACGCATCACCGGCTCTCTTCACATGACAATTCAGACTGCTGTGTTGATCGAAACCTTGACTGCCCTTGGTGCTGAAGTACGCTGGTGCTCTTGCAACATCTTCTCTACCCAGGACCATGCAGCTGCTGCTATTGCGCGTGATAGTGCCTCGGTCTTTGCTTGGAAGGGAGAAAATCTTCAGGAGTATTGGTGGTGTACCGAGCGGGCCTTGGATTGGGGACCTGGAGGTGGCCCAGATCTCATTGTTGATGATGGCGGTGATGCTACTCTTCTCATCCATGAGGGAGTCAAGGCCGAGCAAGCCTATGCTAAGGATGGTACCCTCCCCGACCCCACTTCCACTGATAACCCAGAGTTTCAGATCGTTCTCTCCATTATCCTTGATGGCCTTAAGAAGGATTCCAAGAAGTACCACAAAATGAACGACAGATTGGTTGGTGTTTCTGAAGAAACCACCACTGGTGTGCACCGCCTTTACCAAATGCAGGCTAATGGGACTTTGCTCTTCCCTGCCATCAACGTGAATGATTCAGTGACCAAGAGCAAGTTTGACAACCTCTATGGGTGCCGCCACTCTTTGCCTGATGGCCTCATGAGGGCCACTGATGTCATGATTGCTGGGAAGGTTGCTGTTGTTTGTGGCTACGGTGATGTCGGCAAGGGTTGTGCTGCTGCCATGAAGGCTGCTGGTGCTCGCGTTGTTGTTACTGAGATTGACCCCATTTGTGCCTTGCAAGCCCTGATGGAGGGACTTCCAGTCTTGAGGCTTGAAGATGTTATAGACACGGCTGATATCTTTGTGACTACAACTGGTAACAAGGATATCATCATGGTTAGCCACATGAAGAAAATGAAGAATAATGCTATTGTGTGCAACATTGGTCACTTTGACAATGAGATTGACATGCATGGCCTGGAGACGTACCCTGGTGTGAAGAAGATAACTATCAAGCCACAGACAGATCGGTGGCTTTTCCCTGAAACGAAGACTGGCATCATTATTTTGGCGGAGGGCAGGTTGATGAACTTGGGTTGTGCCACCGGACACCCCAGCTTTGTCATGTCTTGCTCTTTCACCAATCAGGTGATT |
| **HASTY (HST):**  TGCAACCAATACATGACGCATCATATATGACCATAATGGGTAGTTCTTCCCATCCAATTTGTCCTCAGCGGCCAACATACGAAATACATCATTGTTGTCTGACATGATTGCAGATTTGGTTTAAGAAATAGTACAGTTTTCCTGCCTCTCAGTTTCTTTTTCCCTTGTGCTACTATATAAGTTGAGCCAAGGTGGTCGGTAGAGGCGCATTCATTTTATCTATGCTTTCAAATTCCTGTAACTACTGCTGAGATGTAGCAGTGGTATTGATAAAAGTGCAAAAAAAAGGGTCTGTTTGGACTTGCATTAATACTGATGTACAAATAAATATAATGAGCACAAGCATGCTCTGATGGAGGTAGTTTTGAAGATCTTGGCTACAGTAAGTGGTACCTTGAGATGAAAAAACATCAAAAACATTTACATTCATTGGTAATGTAAATAGCATACAAATTGCATGCCGGGGAGGCAGGACTCGTATATAATGTCAAATAAACTGTGTGCATCCTCCTGTACAAAAAGGCTCTCCGGAAGAATAAAATGATGTGATAAGCACTCACAAAAATGCCGCTAGACCAATCTTACCTGCTTCCTCATTCTCAATAGCAGCTACATGGTGTGTAGGTCTTGGACAATTTGCAACATTCGAGATTGGATTTGGAGACTTGGCTGCCATCAAAGCCTTGATATTACCACCACCAGCCTGCAAGAGAAGGCTCTTTATATACTGCCTATGTTCTTTAGCACTGCAGGTTGTGGATAATGCATTTTCAAAAGCCAATAGAACCTCTCCTGTAATTGAAGGGGATGATTGTAGGATCTGCCGTGGAGCTGCATGCTGCTGTGCCATTATCAAATATATAGTCCTAAAGAGATTGATCAGCTCTGTCTGCGCTGACGCATTTGAGTCCATGGTAAGTGCCTCGATAATTGCTGTAAACAAATCAGTTGCCACATAGCTTTGAAGTTCCATATTGCCAGAAATGATAGCTACTGTTACTGTGGCTCCACAAAAAACTAAGGCTTTATGTACTGCTTCAGTATCTGGCCAATTCAAAGCTTGTATGCATATTGATAGAGATGCCTTGGCTATACTATGATGCTGTAACACAAGTCTCATCATACAATCAGATCCAAGTACTGCAGAGCTATCAAACTTCATTGGTTCCATTCGAGACACTAACAACTGATCTCCAGAAGGTAAATTTGGGTTGAGGGCAGGGGATGCCAATACTGCCAATAATGTGCAAGTTTCTCTCGAGAGATCCCTGAGCAGTTTTTCTTCTAGCACTTCTTCCTTCAGGCCAAGTCCTTCACTCTTGCAAGCACCCTCAGAAAGCTTCACTGCTCCATTCTTTATCAGATTCATCCAGGAAAAGGATAATGCTGAGTCGCAGAAAGCAAGAGGACCTGGTAGAATTTTGCCAAGCCATAATTCCCTCATATGCCAGGGACAAGACTTGACAAGAGGAATCACTACGAAATGCAGCAGTAGCCGCACGTGCCTGTATTCCATTGCTTCAATATTTTCCAATAGAGCGATTTGAAAAGCATTACAGTCATGGCCAAAGAATATGTCTGTGAAGTGTGTAGCTGCCAGACCAAGTAGGCTGTAGCTGCTGTCACGAACACCCTTCAACCAATTCCGGACTTCATTTTCCTTTCCACCTATTTGACTATCTGTATGCATTTCCACATCATTAACAATAGGAGCATTTGCTTTTGAAGACTTTGTTCCAGCTTCTCCAATGATGGATGCCTGCTCTGCGCTGCTCATTAGCAAAGCACCTTGTACCACACTAGGTAATGAATGCTTTATATTGGGGGACCATAAAGCGTGTATGCATCGCAGCAACTGCAAAAGCGGTGTAATGATCCATTTTATATGATTTACTAAAGGATGTAGAGGTTCTGTTGCAGTCGCTCCTTGACAGAAGAAATCGGGATGTTGAGTTGCACATCGTTTTACAACCCTCTCTGTAAAGACGACCGAATGGAAGATTGACCATAACTCTTCATTCGCATTATTGCCTGTTGGTTTTGTCAATAAAGCCACAAGATTTTCTGGGCTTACAAGAAACCGCTCTTGCAAGTTATTACTGCTCCACGTAGTTTGTATGGGAGTCAGAAAGCAGTCTAAAGCCTTTGCTTCACATTCTTTCCCTGCTGCTGAGCCTGCCACCAAAAAAGCCTCAGCAATCAAGTTATGCTCGCCTTGCTTCAGTAAACCCTGCTCTTGTAGAGTAGATATGGTTTTCCACATGACCTCTAAATGAGCAGAAACCGTCTTGTCAGCTGCCTTTGCAATTCGCAAAAGGGATGTACAGACTTGCAGCCTGGAGCGTGAATTTTCAATCAGTGAGGAATCTTTTCCAAAGCTCGTTGAAACCACTGGAAGGCTTGCAAGCAATTCAAAAATCTTATTTAAAACACTTAAAGTTGCAGCAGGTGAGCTTTTCAGGAAAGGGCCCATGGCATCCAACAGGCGCCCATGAATTTCTACATAAGCAGAACCGCTCCACTTGGCCATCAGAAGAGTGCTTAGGATTCCTTCGAGTGGTGCCTGCAGAGAAGGAAGCTCTGTGGATGGAAAGAGATTTTCTCCACCAAAAACTGCATAAATAACAGCCTCCAACAATTTTTGGGCACTGTCAAGAACTCCCAGTGTCTCGTAAACGCGTTAAGACAGCAGTTTGAAAACTGGAAATGGAATTTAACGCCACATCACCATGGATATATTTGAGCTTGCAGAACTACCACATGCCTCCTTTATTCTTTCGGAAATTTTGATGACAGCTATTAAAGGACGCCGTACTGCCACCAAACGAAGCAACTCCAACAAGCGGCCACGATAGTTTCCATAATCCTTCACATCAGAAATCTCTTCAATGTTTCTTGCTGAACCACCAGGAACCTGCGCTTTTGAGCTGTTTTGAAAATACCAGTTGAATGTATGATCTAAAAGGCTTGCATAAACATCGTTTGAGATCAAGATCATAATGCCCTTCTTGTCCTTGAAAACTGGTCCTATAACACCCAGCTGTCCGGCAATTAACTCGTTTCCATGACTATATTCAGGATCAGCAGGTATGTCCCTTAAAACTCCCAGAATAACAAGGTCGAAAAATGAACACCAATTTTACTGTGCTGATAAAACCAGAGCATCTGCTGAAGATAAGCCGTAAGGTGATCTTGAAAACCCACAAAACATTGAAGGTTTTGTGATCCAAAGGCAACCAATGCCTCAGTTAGATATTCTGCAAACTCTGAGTCATCCTCTTTATTTGCAGATGGTGCATTTTGTAAAGCTCTAGATGCTTGAGACAAGATTTCTAGAACACGTCTTAAGGCAACATCAAATGCTGACGCACTTTCATCCAATGGCCTTTTTCTTGATGAAATTTGCTTCATAATTTCACCAGACCGTAGACGAAATTCCACAGTGTTCAGCAAAAATCCACAAGCTTCGATCAATCCACTCTCAGCTATCTTGTCTACAGGTGCCCATTCCACGTAAGCCAGAACAGCATTCAGTGTTGCATTGACCACAGCTGCATGCTGCTTTGCAATGTCCAGCTGATTTCTTTGCAATAAGGTCAGAGCTTCACCGAAATGACACTCTAGCATCTTATAAAACAAAGGGAATATCTGTGGCAATGATTGTGTTAGGCTTTGAAGCAGTTGTCTACGCCTGTCTCCTTCCAAATCCTCGTTATAGACTGTTATGTCCTCAGGAAGCCACCTCAACACCATGACGACCAACTCTGCGTATGCAGGGTTCACTGCTGCCAGCTCCATGAGTTTGAACAACAAACCTTGCCACAAAGAAATATCTTCGTGTCTTACAACTTCAGCCACCAAAGCTGCAGTCTGGCTCTTCAAAAACCATTCATCCTGAGAAGTTGCAATCTCAGGCACCAGCCCCAGCACCAAATCAGCCAGTTGTGCCCTAGTCTCCACCATTAGCTCTTCCCATCGCATTCGCACTAGATATTGTAACAGCTTCAGCCCATAGTGACGAACTTCGGATGACAAGGAGCTTTGTTTAACTAGAGCAGATGCAATGGATCCAAGAATTTGAACATCTCCAGTTTTCAATGATTCAAGGAAGGTTGAGGCAGCAAGCCTTGCTTCAGGAGGCGAATTGTAATCCAAGGCAGCATGAATTGCACGGGCTGTGATCTCCAACTGCGAGCTCTCCATGACCCACTTAAAAACCCAAATAAAAACGAGGGCTTTTTTCTTTTCTGCTAGGTTTGCAATGTGCTTGCTTGCTCTTCTTTCTTTAGCGTGGGAAGAAAGCACAAGAAAGGCGGGCTCACTCTAATCCTTCACGATCGTTTCCCATGCAATGCCTGTAGTGTAGGCTTCTCATTTGCGTGCACTCAAAACTAAAACCCAG |
| **METHYL-CPG-BINDING DOMAIN 9 (MBD9):**  GAAGATAGAGAACAATATATTACTTGGAAAGGCAGATTTCATGATGTAGCACTTGTATAAAATGTGTCGTAAAATGCAGGTTGCATTAGATTTAACTGAATACCTCACTTTTGCTAATGAGAACTACAAAATTTCTTACATAAAAGTAAAAGCCCTGAGCAACTCCAGCAGAATCCTAGACTGCTGCTGCAATGCTGGAGATTAACACAAGGGCTGTTCATTGTGCTTAAGTAGGCAAAATTTTACTTTACAGCATGCACATTTCCTTGGTTGATTTCTACAAAGCTCATAAGGCTACCAGCAGCACCCTTACAAATCCCAAAGAATCAAGGCCTTGGCAATCTTAAAACTGGGAACTACAATATGGTACAGAGTTCAAAGCAGATTGGCTCATCATTTCTTCACACTTGCGCTGTAGTCTTCTTTTTCCTACCCGATTTGTTGGGCTTCTCCGAGTCTTCAGTATCTGGATCTACTGATGATCTTTTGTACTTGATGGCTGCATCCAAAGAGTAAATTCTCAATGCCAATGCGCTCAGTGTATTGATCCCAACTCCAGCTGAGAGATGTGTCCAGTTACACCAGGATTCCTTCAAGCAACTTGCTTTGACCATCTGTTCGAGCAATGTAACAGATTGCACTAACTCGAGTATAGAAACCGCTGACTTGACAAAGGATCTCCAAGCTCGTCTGCGATTTGGTTCGCTTCTTGACGGCTCCATAATATCAACGGACAACAAAGATTCTATGTCAAGCAAGCTTGCTTTTAGTTTCTTGATAGCCCACAAATTTGTCAAAAGCGAAGGTTGAGATGGTGGGAGCAAGCTCGACATCGTGTTTGCAAAGGAAATCTCTTCCAATGTTCTGGATCCTGACGCATTTTTCCCTTGATCATGATGCTCAGATGTAGGTTCTTTACCAAAATGTGTGCTGGGCTTATCAGATAAACCATTATCAAAAACATAGACATCTGTAGGTCCATTTTCTTTGAGCCCTGCATGTCCATCAATGCTTTGAATTGACTCAACAACAATCTGGATCTTCTCTACTTCCATCGAAGAACTGCAAAGCCCTGTTTCAAGGGTTTTCATGTCAGACAGTACATCTAATGATAATGAATTCATGGTTGTCATGTGGAGGGCAGGATCCAGCAAGGGGGAAACAGGAAGAGCAGGTATAAATCTAGGGATTTTGTCAGCAAGGCACCCGATTTGCAAGATTCTCACACGATTTGAGGTATGCAAGGTAAACCTTTTTAATACAAGAGAGAGGTCTGGAGGATGAAATTCTTGGCCCTTGCTTTTTGCTTTCTTGGCCTTTGAAAGAACCAAGGCTTGACCTTCATCTTCAAATCCCCTCCAGCTGCATGCCCCATAATTATGACCCTGAAGTTCTGCAACTGAGTCATAGGTATGATGACAACATGGACAATGGATTCTTGTTGTCCACACTAACTCCAAGCAATCACATCTGAATATCTTTCCCTCACCTAAGGACTTCTTCTTTCTGCCCCGTTTACGAGAAGGTTCTTCACCAGGTGCTTCTGACTGAGATATTGATCCAAACTTTTTGGAAAGAATGGTAGCAGCCTTTGTATTTACAGAGACCGGAACAGCAGATCTTGATTTGACTGTGTGCTGTTTATCTGGTTCTTTTTTATACTTAGAACTCCAAATCAAGCACCACTTTGTAAGAGCAGATTTTAGGCATCTTTCAGTAGCAATCGTGGGTTTTAACCACTTAAGCAGCTGCTCAATACTTTCGTCATCACAATAAGCAGACCATTCATGGATGCCTGGGTGCGCAATAGCACTCATCTGAGCATTTCGAACATTTGAGCCAGTTGAAACCCTTGTGGCTTCAGATAAGCACGATGACTGGTTGCAATTAGCAGCATTTGTATTGTCATCTACTACAGAGCCTTTTTCAACTATTACCCAAGGAAATTTTCCTGCCCAGCCAAGGGCCCAATAGATCCTTCCCAGATCATCTCTTCCCAGACAATCTCTGCGAGGGGCCATTTTCATCAGATCAATCTCTACTTTCTCAATCTCTGCCTCCAGTAACTCCAATGCTGTCTTACCAGGTGTTGCTTGAACATTGGGTAAAGGAGAGGCTTCAAAATCACCACCACGATGCACATTTGAAGAGCTTAATGGAATTGAACTCTTATCTATTTGACCTTCAAGTTTCCAACCTTGTAAAACACCACTGGATCCCTTCTGGTCATCTACAGCAGCTAAATCTATTGAGCACCTGAAGGTGTTGACAGGTGTTGCATGGAGAGAATGATGAACTTCGTCAATGCTGCCTTGCATGCTTCCAAGGCAATTGGCACTCGCCAATTCCTGCAGTGGGAAGTTTCCAATTTGAATATGTGTTACATTCTGTTCAGGATAGGTTACTCCAACTTTGTTGAGATCCTCTGAATGATCCCTAGAAGCCTGAGCAGTTATCATGGGAGGCACATCTAAATCAGGATGCCTACTGCTCATCTGTGCTTCTTGTATGGTTAGCCCTGAAGAGCCTTTAATCAGAATAAGATCCTGTTTTTGAGTGTCACCATACTTTTGATGCATTCCAAGAGCAGTCTCGGACTCACTTTGAAATAACTTATCAATTATTTTCCCATCCATAGTACCAACTGTAGAATCGAGTTCATAACCACATGAATTCTCCGCTGGTATTCTGAAAATTTCTATTCCACTCTGGTTTCTAGTGTTTTCGTCCAACATGGGTGTGCTAGATTTCAAACTCTTTCTGCCTCTTTTCTTTGGCAATGTTTCCTTGGATCCAGCTTCTGTGTTATCTGATACAGGAACATCAACCACCCTGTGGTTTCCTTTCATTGCAGCAGCCTGGCGTTCTATCAAGAGAACTCGTAACCGCTGTTGAAGTTCAGGTAACCCCTCTATGCTTTGGTCAATGTGATCCCTGATGAAAGTTGATTCCAAGACTTTGTCACATAGATACTTTAACAATTGCACCCTCTCTGATCCACTTAAATGCCAGTAATCCTTGCCATCCATGCTCCTCGCCAGCGAAGACAAAATGCTTCCATCTTCCGACAGTCTGCTTTGATAGAACTTAAGAATTGTACTGGGTTCAGAAGCAACTGGACAGCTATTTGAACCTCCTTGCTCCAAAGCAACACAAGATGGACAATACCAATTCCCTTCTGGAATCATGGAAAGAGGGGGCTCTAAGCAGTAAATATGATATTCCGCATCGCATCCATCACACAATAGAGTGCTATCATCATCCTTGTCTATTCCACAAATCTTGCACACCCCTTCCTCCCAAGGAGCTTTTTGGAGTTCGTCATCTGACATCATTGATGAAGAGATATTGCTATGCTCCGTTGCAGCATCCTTTCCTTCTGTTACATTTACTTGGTTTGTTTCTTTGAGCTTAGCAGCTTCCTCTTTACCATTCACCAGCTTAATCACCTTCTCCTCATACAAAGTTTCAAAGTGCTGCGACATTTCTTGTCCAACTTTAATAAAATCTTCACGATCTCGAAAAACAACAGGGACATTTTGAAAAAGCTGCCGCATGTCTGCAGCATATGATTCATGTGAATCACCGTAGGATCCAGCAGCCAATCGAGAATCAATCATTCGGAAATCAAGAGGACGGGCAACTAATGGAAGGGGTACTTCAATGTTGTCATCCTCTTTTTCAAAATATCCCAATACTGTTCCTCCGAGAGAATTGCAGAAGAACCGCACATCATCTGGTGTGATAAGTTGTCGAAGAATGACACGACATCGTTTCATTACAAGGTCAGGAGAGGCAACCATATACTCAACCTTCTTCTCCACTTTTGGAGGACTTACATACTTTTCATTGGCAACTTTCTCCAAGACTGAGAGAACAGCTCTCTTCGTTGGTCCAGATGCATTTCCCTTGTAAACCTCTTTACTGATTGACCATTCTAATGTTTCTCTGGCCCAGTCAGGAGGATTCATTTCAAGTGCGTCCCTTACACAATTTCGGATTCTTGCACCCACATTTGTGGCAAGCTTTCGGACTGGTTCTAGAACAATGGCCCAAGGAGGCTTCCTAGAAATTCCTTCGGAATGTGACTCATCGGAAGGCTTGCCATCTGAGCCTACCTTGCCTACGGGCTCAGAGTCAGACTTGTCATCTGCACCTGGCAGCCAGCAGGAGAGTTGCTTTTCTGCAGCAGCAAGCAGCAGAGTATCAGCTTCAATGGCTGCCACTCCATCCAAAGCACCACAAAGAACTCCACCATCTCCTTGTATGCACCGTAATATCTTCCTGCGCTCTTGAACACTTATTTCAAGACTCTCTGCACATGTATTTAGTGTTAAAAGGCCAATAACATACCGCCTAGCTACTTCAGGCCAGGTCAGGTCATTCAATGGCATAAAGCTAGCTGACTGCTTCTTCATCGTTGCTGCAGCTTCCCCCTCCTTCTTTTTACCACGCTTTGATTCAAGAGCCAAATCGGAACCAGCTCCCAATGCCATTTGCTGAAGATCAGATAGGGCAATTTTTAAAAGAGCAATATTTGAGGCAGAAAATGCTACACTGCAGCCTGGAGAGCTAAAAGTTTTCTTCTTTGTGAGCCTTTTACTCTGGAAGAAGCCTTGGTCCTGAACTTCTGAGTTGGCATGCTTCACAACATCCAAGTCATTCTGGAGAAAGCCTTTATCTTGACTGGCACGTTGGCTTGCCATATACATGTCCTTTTTGCTATGGCCTCTGTCAGTGTCCATCACTGAGGCATCAGCTACAATTTCTTCCAAGTCCCCCAAAGAAGGGGGCTCCTTCTGACCAAGAATGTCTGAGAACCGACACAGAAACTCCCAAATCTGAAGCAAATCCCCAACAAAATTAGCAGATACTTTTTGACAAACCGGCAACCCAGTAGGTGGTGTGGGACGTAGCAATTTTAGCTTAGCCTTTTTGGCTTCCAGATACTTCTGTACAACTGGTATATTGAAGCCAAACCGATCCTGCCCCATCCATTCATATAATTTTGAAAAAAAAAACTGTGCATCATGTAGTTGTCCTTGCGTCGCACCCTCTACGTTATTTGAAACATTGAGTCCCAGAGCAGATAGAAGGGAGCTTTCCACATGCTTGCAGCCCAGCTGAAGTGAACCCTTTCCATACAATTCTGCTGCACGTTTTACAACCTGTTCTGCAAGCAGTCTCCAAGCTCCCGAAGGCGAGGTGCTCTCCACAACAATCTCTCCAATTTCATCCCCTTCCCCTAGATTCCTGACACAATTTTCAGGCTGATGATTAAGTTCCACGATACTCCTTTTTTCAACTACTGTAGACCCAGGGCAAGTAGCCTCATTTAAATCCAGCTGTGAGTAATTCCACCCTTCATCTCTTGCTATAGATACACAAAGGTCTCCGTCAGTAAAGCTCGTAAGGGTTCCTTGCAGCATGCTTAGCTCATCATCTTCATCATAAACATGGTTAAGGTACTTCGTCTCTATCTTCACAGCTTCTGGTCGAGCTTCCACACCAGATTTGTCTGGTCTCTGTGAAACCAAGGCCTTTGGGAATCTTCCATCATCGCAAGAATCTAACGGAACATTACAGGACCTTCGTGTAACCCTGAAAATAGGAGGCAACCCTCCACCATCTACAACCTCTGAGATGCAAATAGACCCTGTTACTCCATCATGCCAGGAGCAACGATAGCCAACAGGCCAGACAGAATTATCACTGTTATACCCAGGTTGCATATCTACGACACCCAAAGCCTCCACTCTCAAGTCCATGCATTGCAAAGGCAGCTGATGCTCAAGGTCCCGAGCAGGGTCCTGCAACTCCAAAAAATTATCAGTCTCCATCTTTCTTCGCTTTATCAGTTCTGAAAACTCCCCATCGCTCTGTTGAGTTTCTTGTGAGACATCCCTTTTTCTTCCTCTTGCGACAGTATCTTTTGAATTGATATCGTCTTTCCTCTGCTCCAGAACACCCAAAAACCTGGCTACTTCCAGTTTAGAGCGGAATTTTTGTTTATCGGGAGAAAAGAACATAACATCCATGGCCTTGTCAGAATTGGCTCTTTTTTTGACGCTAACACTCCACTCCCCTTCTAAACTTCTACCTTGCTGTCCTAAAAACTCCTTCAATTTCTGCAAATGCTGTCTTTCCAGGTCTGTATCCTTTGATGAAGCCTCCATATCATCATCTAGGTCTTTGCTCAATGCACTAGCGCTTGTGAAAGACGAATCCCTCTCCCCAGCAGAGATGGACTCCAATATATCTGCTCTTCCAGTGTCTGTTGGCACATCCTCCTTAGAACGACACAGCGTCTTCAACCTTGTCACACGTGTTTTCGGTTCTTCCAAGGTGTTGTTGCTAAGAATGCTTGGCGGCATGCCCATATCTGCATCCCCATCCCCTTTCTTCTGCCTCTTGCACTTTTCTTTTTGACTTCCGTCGGAAGGAAGCTCACTGTCAACCAAAAGTGGGCTTTCTGCAGCATCCATAACACCGTCATGAGGCTGTGATGGGTGGATTTGCAAGCCATCAACAGTCGCAACTTTGGAACCTTGACCGGCAAATAAGCTGCCATTGTTAATTTGAGGTGTGCCCTGAGATTCGTGGCTGCACGCAATTCTCCCTAGTGGCCAATGCCGTTTTCCCTGGTTTGTGCTGCATGCCAGGCAAACCCACTCATCTATGGCAGAGGCCTGGCTATCTCGAAGTTTCAGGCACTTCGAGTGGAAACTCAGCCCACAGACATCACAGATCAATGTCTTTTTACGAACTTCCGGCTTGCCACAGCAAGTGCAACTTACAAAAGCACCATTTTTAAGAGGGGTGCCCGCTTCTCCGAGGAACTCCGCAGCTGGTCCAACATGGTTTGTGCGTAGTCGATACAAAATGGCAACCTGCATGGTGTCGAGGCCTGAGAGACTTGCAATGGGCAGTGTCTGTGTAGTTGAACTAGCAACTGGAAGAGTGTGTGAGGCCTGCATGGCTTCTGGGACATACTGGCCATACTCAACTTCTCCTGACGAATCTCCTTGCTGATTTAAATCAATCATCAGGAACCCTCTGCCACCCATGAAGACAACTCTGAGAGAATAGAAGCTAAACCTGCAACGAGAGACAAGTTGCTCGCCTGTCTGGCCAGCGACGCTTATCTATGGAGCTTCCATGGTGTACAAGAAGAAATAAACCCTAAAAGCAGATCTGCCAGTGCCTGTGTTGAAGGAGACGAGTTCAAAAGACTGCTTAGGGCGATGCCGCTCCTCCGCCCCGCCCCGCCCCCTGCTCG |
| **GLIOMAS 41 (GAS41):**  ATTGACGCTCTTTCAGCTCTTGAATTGATGAGCTGCACTTGCTTTCAGTTGTAAAGACTCCGTCTGAAGAAGGTTAACATCTCTTCTCAGCTTCATAGTCTGGCTCTGTACCTGTTGTCGAGCTGCTTTTAACCGTACTAACTCGTCTAAATCAGAATGCTTTAAAAACCACTGCACTTGTGGATGGTCCTTTGTATCACCTCTTTTCTTCTCAATGGAAGTTTCAGCTGCAGGAGTCGGAGAAAGATCGCTGAGCAATCCATTGACTATCACAGAAGGGTGATTCCTGATGCGTTGAACAAAAGCTTCGGAAGGCTCAGAAAAAACGATTTCGTCATACGTCTCCACAACAACTGGCTTCTTCATAGAAGGTGGGCCCGCATCATCGTCTGGATACAATTTCAAGTGATGAGACAGCTCAATGGGCTTTTCTGCTGCATCACCATGAAAGATGACCGTTATTCCAATCTCAAATTCACCCCATCCTACCTCGGTAAGCTCAAATGGTGCTGTCTCCACCACCCTTGTAGGATTATTGAAACTTGGGTGAAGTGTGAAGATCACTCTCTTAATCACAGAACCAAGGTCCTCGTTGGATATGCTTCTTATGTAAACTGTCCATCTGTGAGAGCGGTACTCATCTGCCTTCTTGCCTAGCCAGTAAGAAATCGTTCCGTAAGCAATCGGGCACGATATCTCAACATCTTTGACACGACGAACTGGAAGCTGCATGTCTTGGTAATGCTTTTTGCTGAACGAGTCCCTGAAGCTTCGTGCAACAAATTTCTTTGCTTGAAATCAAGCATTCATAGCTCATTTGTAGGCGCCTGTCTGCCCGCAATTGATGAAGGCAGCTTCTGACGTAGCACTATAAAGTTTAACAAAGAGGACGTAGGAACAATACTTTAAAGTTTAACTTGGGCGTTAAGGTGATTCATTGCCAAAACCGGGTTCGAACCCTGTAGGC |
| **ENHANCER OF AG-4 1 (HUA1):**  GTGGCATTCCAGACTGGAAGGAGGTGTCAACAACTCCTACAACCAAAACACAGTCGTTGCCTGTGCGAGAGGGAGAGCCTGACTGTTCGTTTTATATAAAAACTGGAAGCTGCAAGTTTGGCTCCAAATGCAAGTTTAATCATCCTGAAAACAAATCAACTGCTGTTACTACAAATCAAAGTAATAACGGCAATGTGAAGGCATCGGCAGCTTTAACTGATGCTACAGTTGATAATGGATACGAAGAAGGTGAAGAGGCTAAGGCTGCTGGCTACAATGCAAAAGGGCTTCCTCTAAGACCTGAGGAGACTGATTGCTCATTTTACTTGAAGACTGGAAGCTGCAAGTTTGGATCTGCATGCCGCTTTAATCACCCTGCAAGCGTGGCCATTCAACAAAGCCAGACGACAGTTGCTCCTGTGATGGGAGGCTTTACTGCACCATACGGAAGTTTCTCTATATTTCCAGGGGCTCTACCAGACTATGGGTTTAGCATGCCAGGAGCGGATCTCGGATTGTCTAGTCTGGCTCCCCCTACGGTGTATCCACAACGGCCTGGTGAAACCACCTGCTCGTTCTATGTGAAAACAGGTATTTGCAAGTTTGCAACCAGTTGTAAATTTCACCATCCTATTGACCGAAAGGAGCCCTCCACTAAGGTCACACTTGCCGGCTTTCCCAGACGAGAGGGGGAGCAAGCTTGTCCATTCTACATGAAGACAGGCACTTGTAAATATGCCCTAACGTGCAAGTTTGATCATCCTCCACCTGGTGAAGCAGCTGCCAAAGCAGTAGCTGAAGCTGGGAAGTCGGAACAGCCTGAAGCTGGGAAGTTGGAACAGCCTGAAATAGGCGAGCTCACCCCCAATTCCAGAGTTGCTCGAGGCAAAGTTGTATGGAATGACTGAGGGAACAAAATAATGTTGCCTTGGGCTGTAGATGGTAGTGGTGTGCAGCTTCGTGTGATGTGGGTTGAGGTCCATGTAACGTTTTGATTGTGAGTAGGATTATTTAGGCTTACCTGATTACGGTAATGTAGTGTTTTTACTAGAGTCGAGCATTTTAGCTAGGCTAGGGATATCTACAATTTTTTTTTGGGTTCATTGTTTGCAGAATCTTGTTTTATTCCACATTAAGTTCACATCCGTTGTTTTTCTTTTGTGGATGGAACTTGTAGTCTTGTTTATGAATTGAAGGAAAGAAGCCAAGGCCTTCTTTTATACAAAAGTTTGGGAGGATGTAATCAAGTCTGCGCTTCACGTAAAACTCTGATGATAGTCGTCACATTCTCTAACAAAGTCAATTGTGATGGTATTTATGAACATCTTCAGAATGGTTCTTCAGAATGGTATTAATGAACACCTCCAGAATTGAAGTCTGGCAAAA |
| **SEUSS (SEU):**  CATACACACACACACACACACACGAGTCTTTCTCTATGAGTTTCTCTCACACACAACCCACAATCAAATTTGACGTTCTCTCCCTCCCTCTCTCTCTCTGTGGGTTGTCTGCGCAGACCTTCACCTGCAAGCCCGAGGAAACGCTAACCCTAACCCTAATCCTCACTTTTTCTTAGCTTATAATCAAAGAGTCCCTGCAGCTGAAGCTAAGCAAAACAAGAAAAATCAGACTTTACATTTATCCATCTGGATTAACCGGATTTAACGGGCAAGAGCACAGTCAAGCTGGCCTCAGAGCAACAAGCAGGGCCCGGCCACCAGCCAGCCCTGCATCCTGGTGGCCCTCATCCAGTGGCAGGGACTCAAGTAAAACCAACTCCAACCTCCATGGCAGGTTCGCAAGGCGACAGCCCCATCAAAGCAGATGGAACATCTTCGCACTCCGATCAATCATCTAACTTGTCTGATTGCTCCCAGGTTCTCCAATACTTTAATTGCTCCGATGGTCTGTCTGTATCAGCAAGTCTTATGCCCACCATGGGATTTGGAGAGGGAAGCCAAGCGCTAGTGAGTTCAGTCAATCTCAGTGCCACAAGTGCCAGCTCTCTTACGACAGGGGGCAACTCTCTTATATCAGGTAACTCAACTTTACAGCAGAGCATGAACATGAAGGCAGACTCCTTGAATTCTACAGGTGCAGCTTCCATGGGCTTGCCTACTTCACCTTTGTCTTTCTCATCTAGCAATATTAGTTTGCCAGGTTCTTCAGGTTTTAGTATGACGTCCTTGTCTCAGTCAGGGGTGAGGTCTATCAACAGGCTAGATGCGCCTCACGACCCTACTATACATCCAACCAAACTAATGAAATTGGACAATGGCTATCCACCCATGCTCAACAGTGAGGAGCCATCATCTAAGTTCGGGTGGCAATACAATGTGAATAACTTTGATGTTGCACGCCAAATGCTAGGTGCCAATGCGGCTGGGAGGTTTGACCATCAATTCAAAGGTCAGGGTCTAACAGGAGCACGGCAAGATTTTCATAGTAACGAGCATCAGTTGGCTCAGTTGCATGCTTCTATGTCAGCTGCGAAGGCTATGCAAAAGGATTACTTGCAGCAGCAACCTGTACAAAACAGATTTCAGCGGCCAAACCCTTTGCTATCAGCTCAACTTCATAGGCACGATGTCTTACAGCAACAGCCTCAACATATTCATCCTCAACTTCTACAGCAGCGCCTTTTGCAGCAGGAGCACCTCTTGCGAATGCTGCCACCACAGCTCCAAAGGTCACATGTTTTGCAGCAGCAACATCAACTGCAGCATCAGCAGCAGTTGCAACAGCTGCAACATCAAATGGCTGGAATGAACAAGCAACCAGATAAGGGTTTTTCGGGATCTTGTTCCCGGCGCTTGATGCAGTACCTGCACTATCAGAGGATGCGCCCAGCAGACAACAATATTGGGTTTTGGCGAGAATTTACAGCCGAATTTTTTGCCCCTTCTTCTAGAAAAAGGTGGTGCCTGTCCCAGTGCAGTACTGGTGGGCGGCATCAGCCAAATGGAATCTTCCCACAGGAACTATGGTGTTGTGAAATATGTGGTTCAAGTCCTGGTCGTGGATTTGAAGTCAGTGTTGAGGTTATGCCTAGACTTTGCAAGTCCAAATTTGACAATGGGGAACTTGAGGAGCTGCTTTTTGTGGATCTTCCTCATGAATACAGACTTAGTTCAGGACTGATTGTTCTGGAGTATAACAAAGCTATTCAGGAGAGTGTTTATGAGCAACTTCGAGTTGTTTGGGAGGGTCAATTAAGAATTATTTTCACTCCAGAGCTGAAGATTCTTTCTTGGGATTTCTGTGCCAAGAGTCATGAGGAGCTCCTTCCTCGCCGAAGTATTGTGCCTCAGGTGAATCAGCTAGTTACTCTGGCTTCTAGATATCAGGCTGCCATGGCACAGAGTAATGGAAGTGGGCTACCTGTTCAAGATCTTCAAGCATTCTGCAAAGTGTTTGTTTCAAGTGCTCACCAACTGACCAACAGTTTGGAGCCACCCTGTGTGAATGAGCTCGGGTTTACTAAGAGATTTGTTCGCTGCATACAGATAGCGGAAGTCGTGAATAGTATGAAGGATTTAATGGACTTCAGTCGTGACCACAATTTGGGCCCTATTGAAAGTCTCGCTAAGTATCCAACTTCAAGGTCATCTGTAGAAGTCACAAATTTTGTCAGGCAGAGTGAGTTGGCGGGGCTGGATCGACCTGGAGTAGAAGAGAATTCGACCTCCAGTTTCCTACAATCAAAGATTGATGATCAAGGAAACCTGATAAGCAGGCCTGTACTTCAGCCACGGTTGTTGCAGCAGGGTACCAGCTCTTTTCCAAATTTTGCACATATTGATTCATCCACAGTAGGGACACCAACTGCTGGAGGTGGCTCTGCAGCAAACTCCCTACAGGCCTCTCCTACCAACTCCCTAACTTCGTACCAGAGCTGTTTTTCTTCATTGACTAACAACTCTCAACAACCTATTACTTCTTATCAGAACCCTCTTACTGTCAATTCGGGTAACTTGAATATACTAAACCAGCCTTCTCTTCATCAGCACATTCAATCTGGTGAGCAGATTGGCAGTGGTGCAGTGCAGCAGTTTCTTCAAGGAATGATTAGTTCTCACCCAAGGGCAGGACAAGCTTTTAGTCCAAGGAGCTTAGGGATGAACATTAGAAATACAGAGGGAATGATAGGATCTGGCCTTTCTAGAAGCATATCAGGACTGAACAGTCCTGCTGGCAGCGGCAATGTCGGTGGTTCAGGAGCCATTTCTGAAACTCGGCAGCAATGATCTTACATTTTCTGTGCGTTGATGGCCTAATTTGCCACAACTTTAGATCACGATACTTCTGTAATGTTTTTGCAGAGCTTGTTTTGATGCATGGCTTCATCATCAGGGATTTGGAAAATTGAGTTGACCATAACTAACAACAAAAAACAGAGTCACTAAATCAGCTTGATTTAAGGTTGTGCCTCTTTGGCAACAAAGCTAGAAGTGCAAACAATCAAGAAAACAAGTATGGGGTCTTGGTATAGTAGCAAATGTACATTTTGGATGGCCATCCTCGGAAAGATAGCTTTGTCCAAAGAAAGCTCCGTGCTGTATGGAGTTTTGTGAATGCTGTGGGCCATGGTCTTACTTTTTACTGAAGCTGCAAAATCAACATTGTGGCAGCTTTTTGGACATGACATTCCACGTACGTAGTCGTTTGCTACCTGCCAAATAGTCAAATGGTTACTTACGTTGCAAATTGGCAGGCTGGTAACTTGTATTTCTCATGGAAGATACGGTGGGTTTCCCAGTGGAAGTATCATAGCTGGTTTTGACCCATGTTGAATACTTTGGCCGCCGCTTTGTATTTGTATCAAGGGTGTTAGCCACAGAGGCCATTTATGTTACCGAAAGGTGCAGCTTGCTTACTTTTTTTTTGGCTGTGCCATTGTATGTTATGACTATTACTTTTGTAGTTTTTGGCTCAGATATTTGGAGTCTCCAAACAGTGTCTATAGTGGCCGATCCACTCTTATTTGCATGTTAGACAGCGAAAGTTGAGTCTGTGTCGTTTGTAAAGAGGATTATGCTGTTTTTTGATCTTTTGTACTTAATTCTTATGTTG |
| **FLOWERING PROMOTING FACTOR 1 (FPF1):**  TTAGTAGTAGACATCTTACCACATAGATCAGCATGCTATAGGTCGCGGTCCTCGAAGTTATAGGTCGCGGACCTCGAAGTACTCGCGATTTTTGAGGACAATGTCGTACATGTGCATTGTTTTGAATTTGCGGAAGTCGGAGGGGAGGGAGATGAGGAGGGAGGAGGAGTAGCAGCGATGGTATTGGAGCGAGTCATCGGAGGCGGCGGACGAATAGTATCGCTCCCAACCGAGGCCCTGGAGCTTTTCCTCTAAATCTGAGTAGGAGGAAACAACTTCACTAGTGGGTAGATAAACTAAGGCTTTCCTTCTGCCCTGCTGCCACTCTTGGCCTCCATCAATAGGCTCCACCATGGGGTTTTGAACAAGCTTTGCTACACCGTTCTTGAAAACCCATACACCTGCCATTGCCTCCTGCACTCTTGTGTGTGTAGCCCACCTGCTTTTGTTGCTTTTCGCTCTCTGAAGCCCACCTGCTTTTGTTGCTTTTTACACTTAGCCCACCTGCTTTTTGTTGATTTTTGCTCACAGCGCGCTTGTGTCACTTCTGTGTGCGTAGCCCACCTGAGTGTCATTTCTCTGCTGCATTATGTTAGTGTGCGTGCCAGCCAGGAGATGATGTGAGCTGTACATAGAGCTGTGTAGTGTGAGGTAGGCA |
| **GIBBERELLIN 20 OXIDASE 1 (GA20OX1):**  TACCCGCCATGCCCGGACCCGAGCAAGATCTTGGGGCTCGGGCAACACACAGACCCGCAGAGTCTCACGCTGCTGCTGCAAGATGAGGTGGGCGGTTTGCAGGTGCTCATGGAGGACGTCTGGGTCGCTGTCAAGCCTCGCCCCGATTGCTTTGTTGTCAACGTAGGAGACACTCTCGAGGCATGGAGCAATAGTAGACTTAGAAGTGGCATACATCGTGCTGTGGTCAACACTACTTTCCCTCGTCTATCAATGGCTTATTTCCTTAGCCCTGCACTTAGCACTCTCATATTACCTCCACCTTCATTAGTGGACAAAGATGACCCTCTAAAGTATAAGCCCTTCACGTGGCTTGACTTTCAAACTGAGCTTCTCAGACAAAGACGAGTAGTTGGTAAACAAGCGCTCAACAAGTTCTTCATTG |
| **TERMINAL FLOWER 1 (TFL1):**  TTTTCATCCAAGTTTTTAAATGAGCGTGCAGACTTGGTTCCTGCATACACATACACGATGGCAGGCCACGTTCGTTACCTGTTTTTAAGTACTCAAGCACGCAGGCGGCAGAGGGCGAGCCATGGCGAGCGAGAAGCTATGGCTTCTGTGGTTGGTGATGGGCGTTGGGGTGGTGGGAAGCAGGGCAGCAGCAATAGAAAAGGAAGAGAAGGGCTGTGTGATTCCGCCGTGGGTGGATGCCTATGCGGCGCCGCGTGTGAAAGTGTCCCTTTCCTTCCTCGCCACGCCCGTCCGCTCGGGCCAGCTTCTTCCCAGAAACCTTACCCAGATTCGCCCTCATGTCTCCCTGCACGTGCCTCCTCTCCTTGCCCATAGTTTCCTTACCCTCGTCCTCGTCGACCCCGATGCCCCCTCCCCTTCCTCCCCCTCCGTTTCCAATATCCTTCACTGGATTGTCTCCAACATCCCCTCTGCCCACCTTTCCGAACAAGAGATATGGGAGTTGGGTGTAGAGAATGTACCTTACAAAGGGCCTGCACCTCCCAATGGAACTCATCGTTATTATGCCTTGGTCTTTGAGCAGAAAGACAAGATCCATGTCGAGCTTATTCAGAACAGAGCAAACTTCAGCGTTCGAGATTTCACCCGAAGATATGATTTGTACTATCCGATTGGAGGGACTTTCTTCAGAGTTCGTGCCAATACGTAAAATTGGTGTCTTGCTGTATATTATCATGCATTGTGAATTGATTAAGCATTCTGATCAATACATTTTCTCAAATTCTAAACTCAATAAAGCTACACATTGACATGATATACGTGCTATGTGCTTAACATGGTGAGTGTGCAAAAGTTCATAGGGAAACCAAAGATGAAGAAAATGAAAGCATTTTGGAATCCCGGGCATCATTTGGATGCACACAGTACTACAGATGCAAAAAGT |
| **MORF RELATED GENE 1 (MRG1):**  CTATAATGACTCAAAAAAAAAAATCTAGCATTGATTCATGAATTACCTTTGAAGTCATCTAAAGGATGGACCATGTATGAGACTTCTCTTAACAGCTCTCTCCAGCTTTCATAGTGATCTCACTAATGACTGATAAAGCTGGAGTGCTGTGTCAAAATGAGCAAAATCACTTACTGCTCTGATTAGGCAATATGGTCTGGTTTATCTTGCAGCCAGTCACTTTTCTAGTACGTTATGGCCCACATAATTTGGAATTTTAATGTCACTTTTGGACTGTAATATTGCCAAGCATTTCTAAGAGCAAACATCTCATTGAGACCAGCATACATTTGCAGCAGCAGTTGCACTACTGCACACAGAGCAACGGGGAAGAAACACACATTTTGTCATCTTCCACAGGCTCAAATTGCCGCCTGGATGAGTTATCTGCAATTGATGTAATTAAGCTGCTGCATACTTCTATGCTAGTTCGTGTCTATTCTATTGCTCGCTATGCACCAGCACTCCAAACTAGTTTGCCTTCACTTCATTATCCTGCAATCGGCTTTTGCTTTCTTCACTCCAGGAGACCTTCCCAGTAGCGTTGATAAATTACCAGCAAACACAGAAAAAGCACGGAGCATGCTTCAATTGCAGCCATGCTAGCGAAGAGAAACAAAAGCCATTGAACACCATGCAGCTCATAGAAACCGATGCTGCTTCGGCTTGCATGTCACATCCTCTTGACCAAACCCATGCCCTTCACCATCCCTGCTAGGAGATTATAAGCAACCTCAAAACTTACCAATGTGAAAGAAGCACCTCAAGGGTGCCATCTGATACAAGAGGCAATTAACATTTTTAAATGAGGCTTTGTCTACAAGCTTAAGGAGTGCTTTGAGTGAAACAAAACCCTATGTGAATGCACTCGGGTATTTTTTCATAAAGACCTGCAATTCTCCACCATTGGAAGACACGTGGACCAGATAATCTGCAATAATTCCCTTAGGTTGAAGTCTATAATATTCTGCGTCATTTCCAAAATGTGTAATTCTAGCAGCTAAGTAACCCTAAAAATTTGAGAGTATTACTTACATTCTTACCTCCCCCAGGGGTGTTAGAAGCTAGTTTATATGGTACAGGCAAGTAGAGTACACAGAAGTGTTGTAGCAAATACAAGAAATACAGTCCATTATAGGGAGACGTTTAATTAGAATTTCCTTCATTGGTTATGGTGGTGCACTAAACTTGGATGACAACCCTAGCAGCCTGTGATCAACTTTGTAAGGAGCACTTGTTAAGAATGCCTCTCCTTTCCTTCATCTACCCCAATATCAACCTTTGGTCCATCATAGGTGGACAAGAAGAAGACACTCTGGTTCTTCTGCAAATACTTGAGAAAATCTGCCAGCTTCTGTTGCAACTGTGTGAGAGCATCATCCTCCATGTTGGTGTACACAAGCAATTCAGGCAGTTTCACAAATAGGCGCAAAAGATGCTCTGCTCCATAGACAGACGAGGGGGCAATAGTGCTATTTTCAGGTACATGCTCGAGATATTGCCCTCGCTCTGGTTTATACAGCAACATGGCAGGTAGAGACTTGTCAAAGTATGTGCGTAAGCCATTCAAAATCTCCACCAAAGCATCACCAACAACGCCTTCCTGCTTTGTTTTGAATTCCAAATACTTTTTTAGAATGTCGTCGACAGTGGGGTGACGTGGAAGCTTTATCAGCTTGCCGAGTTGTGTTACAAACTCCCAATCATCAACCAGCTGCTTCTTTAAAGTGCCAGGAAGGGGAATCTTCATCACTTGTTCTGGTTCATCAGTATCCCTATCCTCAACACCTGAATCTGCTTTGCGCTTTTTCCCCCTTGTCACTGCATTACAAATAACAAAGTAAACAGTGCTGTAAGCCATGATGATATGTATCCCCATTGACACAACATCTAGCATGAGCTGTCCCTCCCTTATAAAGCTTGAAAAAAACAAAAACCTTTACACATAGAACATAACTTGCTATCTCTCCTAACCTACCAACTGGCAGACCTTAGACCCAATTCAGGTACAAACTCAATCAGAGCCACCCTTCACTGTAACAGTTTGCTTGCCGTGGAAAATCTCACAGGTCAACTACTCTCATTGGCTGGATTATTATCTCAGCACTCGGCACACAAAAGACACAAAGCGAGAAGTTTGTAACGATGAAATCTGTGAGAAGAAAAGGTGGACCGGCTGAGGCCAAATGCAAAGGAAAAAGCAAACAGAAACAAGAAAAACATCAAAAAAAACTAGAATCATTTAAAGAAATGAACTTGATGAAGATTTGGCTGGCTAGGCATTCCACCAACATAAGCTATCATGTAGATCAACATGCTCATTTTGTGTGTTTTTTGATTGGCTCAATACCCCTCAGCTTGATGGGTTGGCATATGTTAATGCAACCTTCGAGTCTTCGAGAAAAGTGGAGGACTAGATGGCATAGATCCTGTGAGTTGGCTTCCAAAGCAAGTGGATCATCGACAGATTGTAATCGAGGCTGCAGATATCTCGCGTATTCTTCCACACACAACAGAGGATAATGCATATGATTTTATTCGCAAGGATATGATGAGGTTCACACGGCAGACTCGTTCAACTACAGCTCGTCGTCGTGGAGAGTCTGGTGGTGGTACTAGTAGTTCTTAGCCTCGTAGAGGTCGTGGACGAGGTCGAGGAGGTAGCTCCAGACAGTGTATTGCAGGGCCTTCTAGTCCTAGGCGACTTACACCCACTCCTTCAGAGTGTGAGAGTGATGACTCATCCCATTCAGAGGGAA |
| **UNUSUAL FLORAL ORGANS (UFO):**  TCTCTCTCTGTCTCTCTCTCTGCATGGAGTTCACATAGGGGCCGCGGGGGGTACGAAGAGTAGACGCTGACTGACCGGGAGACAAAAGAGAGCAAGCGAAAGAGAAATTGAGAGGGAGCGCCCGTGTTCTTCTGTGATGCTGGGACAGGTGGAGGACCCAACAGCTGCAACGCTGGAGTGGAGTAGCTTACTGCTCCGTTCCTTTTTGATGGAAGCGGGGCTCTCTATGGTAGAGATGGACGCCCCTTTCATGAGCTATTATCTGGATCACACCGGCGGCGCCCTTGTCGACACCGGTGGCGGTGGCTATGGCGCTGCTGCTCGCTTGGAGCAACAACAGCAACAGAAACTCCAGGATGAAGACGATGATGATGATGACATGCAACACCTCATGATGCAACCCCCTAAGAAGAAGCTCATGATGAATGCTGTCGCTGACAGCAGCTCCGTTGTGACTTCTGTTGATAACATAATGCAGGGAGGCTCTGGCTCTGGTGCCGACACAGCTGCTGCTGCGTCGCCCTGGCTCGACCCTCGCCTCTGGAGCAAGCTCCCTGAGAAGCTTGTGGAGAGGGTTGTCGCCAGCCTGCCCCTCCCAAGCTTCTTCCGGTCGCGGCTCGTTTGCAAGAGGTGGTACAGTCTCTTGTTTTCCGACAGCTTTCTTGAACTGTGCGCGAAGGTGCGCCCGGCCAGGCCCTGGTTCCTGCTCTTCCGGCGGGGGGTCTGGTCGGAGGCCTTTGTGTTCGATGCCGTGGGGAAGGCGTGGTTCCGCTTGGACCTCTCCTTTCTGCCCCCGCGGTTCACTGTGGTAGCGGCGGCGGGCGGCCTGCTCTGCTGCATTTCTGAGGCACGGGGCTGCAAGACAGTGCTTATCTGCAACCCCCTCACCCGTGTCTGTGTGCAGCTTCCCGCCGCCCTTAAGGAGCGCTTTGTTCCAACTGTGGGCCTTGTTGTCGACTCCATCACCAAGGCTTACCGTGTTGTTGTTGCAGGGGATGATCTCATCTCCCCCTTCGCCGTCAAAAACCTCACCACTGAAATGTATGACTCTCGCCTGCAGCAGTGGCGCATGACTGCCCCCTTACCTCGCCTCTGCAATCTTGAGTCCGGCAAGACAACCTATGCTAATGGCTTCTTCTACTGCATGAATTACAGCCCTTTCAGCGTTTTGGCATATGATACTGAGCAGGGAATATGGAGCAAGATCCAGGCACCCATGAGACGCTTCCTTCGTACGCCCAATTTGGTGGAGTGCCGTGGCCGTCTGGTGCTGGTGGCAGCAGTGGAGAAAAATAAGTTGAATGTGCCGAAGAGCATCAGAATTTGGGGGCTGCAGCACTCGCGCACAAGCTGGGTGGAGTTGGAGCGCATGCCACAAGGCTTATATGAGGATTTTATGCGTGTGTCAGGGCACAAAGCATTCCACTGTATTGGTCATGGTAACCTCATCCTTATCACACTCCCCGACTGCCCCGACATGCTGCTATATGACTTCTATGAAAAAGTGTGGCGCTGGGCGCCGCGCTGCCCTTTTGCAGGTCACCCACCGCCCCCTCATGCCGCAGCCTCTCATTCGCTTTCAACAGGACCTCAAGGATTTCATGCCTTTGCCTTTGATCCCAGGCTGGAAGCGTCTGTCTACTGATGATGAATTGACTGATGATGAAGATCAGTTGAGTGCTGTTGTTGATGATCAGGTTGGTGCTTCGCTACATTGCTTCTTATCGTTTCATTTCAATTCTGCATCTCTAGGGTCAAAGAACTGGACTAGTGGCATTGCTCTCTTTTTTGGGACCTACTTCTCCTGTGTCTAAGACAAAATTACTGTCTATTATGGGACATCTTGCTGCTTTTTGTCATGATGTTCGTGGGAAATTAAAAGACTCTTGTTTTTCATTGCTGAGTGATCCTATCTCAAACCTGTGATCCAAGCTTCTGCCAATTTACCTGTCA |
| **VERNALIZATION INSENSITIVE 3 (VIN3):**  AGAGAGAGAGAGAGAGAGATGTTGGTGAAGAGTACCTACGCCCTTATGTGAGAGCCCTTAACGCATAAAATCACTCCAGATCTGTGGCGCATAACTAAATGTCACCGCCCAAAACGTCCCAATTGGAGCCCACTTCAGGAGCGTATTTTTTAGGAGCATATAATCCAACGATGGGAGGCCTAAGCCTATCTGAAAGGCAGGAAATCTTGTATCAAGCTGCACAAGGGGCTGAGGGGGGCCGTGAAGCCCTCGAGTCATGGACACGGAAAGATTTACTGCAACTTATTTGCATTGAAATGGGCAAGGAGAGAAAGTATACAGGTGTTTCCAAAGTCAAGATGGTAGAGCATCTGCTAAAGCTGGTTTCTGCGAAGGAACTGAGAGAAGGGGCAGACCCTGATTTTACTTCTCCAATATCTCAGTTTCCAGCACCACAGAGCTCATCTAGGCGGCAGCGAAAAGCAGGCCGTCCTGCCCGAGTTCCAGCAGCTATCCAAGTGCCGGCAGCTGCAAGCATATCAAAGCCAGAGCTTTCTTGGGTGTGTAGAAACACAGCATGCAGAGCCCAGCTGCCACAGGGTGTGAGTTTTTGCCAGCGTTGCTCTTGCTGTATTTGCAAGAAGTTTGATGATAACAAGGACCCTAGTCTTTGGATAGTTTGCACACCTGAGCCACTCAATGTAGAAATGGACTGCAGGCTATCTTGTCACATAGAGTGTGCTCTCAGCAGTGACATGGCTGGGGTAGTGTTGGATGGACCAGATATTCTACTTGATGGAAGCTATCAGTGTTGTTCTTGTGGCAAAATAAGTGATGTGATAGGTTGCTGGAAAAAGCAGCTATTGATTGCAAAGGATGCTAGAAGAGTCGATACTCTTTGCCAACGTCTCTCTTTGAGTTACAGGTTACTAAATGGCACCTACAAGCACAAGTCACTTCATCAGCTTGTAGAAAAAGCAATCCACAAGCTGGAAGCCGAAGTGGGCTCAATCACTGAAGGGTCTGCTAAGTTTGCTCGTGGTTTGGTGAATAGACTCTCTTCAAGTAGTGAGGTACTTGAATTGGTAATTTTGGCCCTTGAAAAAGTAGATGTTCTTGATGAAGAGCCGGTTTCGAATCACAAAGCAGGAACAGAGACGGTAACTATCCAAGAGAAGAATGCCGGTATGTTGTGCACGATAGAATTTGATGATGTATCATCATCTTCTATTGTGTTGACTGTAAAGGGTGGTGGGGAGCTTGTGATTGGCTATCGGATTTGGCATCGCAAAGCATGTGATGCTAGCTTTGCAAACAATCCAACTTGCATAATCACTGCCAATCCTGGGAGGGCTCAGATTTCGAGCCTGCACGCTTGCACAGAGTATGCAATCTATATCGTTCCATTCTTTGAGAGAGGGATTGGAGAGCCAGCAGAAGCTCGATGCTTCACAAAAAGTGTGGAGTTGCAATTACCCAAGGAGGCATGTAAGGACGGGCACCTTAACACTGGCTTGAATGTTAGTATTAAGGAGGATGTTTGCATTTCTGACAAGCTCGAATCCAATTTCAAGGTTCGTGAACTTGGCAAGGTCTTGCATTCCGCCTGGGCTGAAGAGAAACAGTCTGCCCATGTTCGGAAAGGTATTTTTTGTGGTAAAGGTGGGGTTACAGGCTTGATTGATCGCAAGAACAATGAAGAGAAAAAGAATGAATCTACTTGTTTGGGATTGAACGTTCTCTCAAGCCCTGGAGCAGACTTGTCCCATGTGGACAACAGTACGTGTAAAATAAGCCCTGAAAAAGCACCTAGCAGCGAATTGAATGCCTCAGTAAATTTAGAAGCTGGTGTGGAAGAAAGTAGAGTAACTGTGGAGGTAGAGGTGGCTCCACATCCGATTAGAACCCTTTCGCGGAGAGATTCAAGTGTTTTATTACAAGAAGGTGAGGCTAAAGGCCGGCCTGCATGTGCTGAGGTTACAGATTTGGATGACACAAGTGACCCTGGAAATTCTCACACTTCACAAAGGGAAGAAACGGAGGCCCTATCACAGGTTGTTTCGGATGGAATGCAGAACAATGCAGCAAATGAACTGCACGATGTTTTAGACAATAGGCGTAGAATCGGAGATGGTGAGTCCAATGGGCATGGAGAAAGCTGGGCGATGCAAGTTCGAAGTGCAGGAACAACTATCGGAATGGAGCCCCAGACAGCTATTATGAGGAAGAGGACGGCGGAAGGGCTTGGTAGGGGTGATAGTTACGGGCTTGTGAATGGTTGTGGAGGAACTGTGGGAGGTACCCTTTGTGCTGCCCGCAACTATGAGTTCTGTGTGAAGATAGTGCGCTGGTTGGAATGTGAAGGCTATCTAAAGGAAGACTTTAGAATGAAGTTTCTCACCTGGTTTAGCTTGAAGGCATCAGAGCATGAGAAAAGGGTCGTCAGTGTATTTATTGATACACTGCAAGACAATCCTGCAAGTTTGGCAGGGCAGTTGGTGGACACTTTCTCAGATATCGTTTCCTCAAAGAGACACCACATGGTTTCCAACGGCTTCCGTAATAAGCTGTGGCACTAGATTGAAAAAGTGCAACAAACAAGCTGAAACTAAGCAACCTAGTTTAGCTCTAAGTTTGTTTTTGGTGTTTTTATTTTTTTCATTTATATAGCTGCAAACAAAGCATTATTTGTGGGTTGATTGATGTATCATTTGTGATTGTCCTTCAGCTAGACAATTGCCTAGTTCTTAGAATGGCCAAACATTATCTCTTTACTCCTTGCAGATGCTTGGCCTTCAGTCCCCCCCCCCCCTTCTCCTGGGCGCCCGCCGTGCCCCATCAAAACGAATCACTCAGTGCTCACCAGTTATTTCTGGTAAAGTAAGTATGTTATGCCAGCACATTTGAACCTCTGCTTTTGATATAGCCTGTCATTATTGAAGTTATTTTGTTGTGATATTTGCTCTTCCTGTCTTCCATTGGTTCTCTATTTTGCTGTCATGCGCTCAAGTTGTTTATGGGCCTTCGGTGTCGAATGACTGAGCGTAAGTCGTGTTTTTTACTCTTTTAGCCTAGCAAAATTTGCACTTTTGGTCAATTTACCCTGGTTCTATTGCCAGTATGGGACTT |
| **PROTEIN ARGININE METHYLTRANSFERASE 10 (PRMT10):**  GAGAGAGAGAGAGAGAGCAGGCAGCGAAACCATGAGCAGAGGAGCTGGTGGAGCTTCCATGGCTGCTCCTGCTGTCGACAAGGCCGCCGATTACGCCAATTACTTCTGCACCTACGCGTTCCTCTTCCATCAGAAGGAGATGCTCTCTGATTGCGTCCGCATGGATGCCTACCACGATTCCATCTTCAAGAACTCCAAGCACTTCAAGGACAAGATTATTCTGGATGTTGGAACAGGCAGTGGAATTCTTGCCATATGGGCTGCGCAAGCAGGTGCAAAGAAGGTTTATGCTGTAGAAGCCACAAAAATGGCTGAACATGCTCGCCGATTAGCTGCTGGCAATGGGGTGGAGAATATTGTGGAGGTAATCGAGGGATCTATCGAGGACATAGAGCTACCTGAAAAAGTTGATGTCATCATATCAGAGTGGATGGGCTACTTCCTCATACGCGAGTCTATGTTTGATTCAGTCATTCATGCACGAGATCGGTGGTTGAAACCAACAGGGATCATGTATCCAAGTCATGCAAGAATGTGGGTTGCTCCTATGAGATCAGGTCTTGGGAAGGTCAAGTTGCAAGATTATGAAAATGTGATGGTTGATTGGGATGCGTTCGTTCAGGATACCCAGGATTATTATGGTGTGGATATGAGTGTTCTTACTGATCCCTTCCAAGAAGAGCAAAAGAAATACTACCTACAGACAGTTTTATGGAATAATCTTCATCCGACTCAAATTGTTGGAACTCCTGTGATTGTAAAAGAGTTTGATTGTCTTACTGCTACTCTCAAGGATGTGGCTAGTGTTCATTCCTCGTTTCAAATGCAATTATGGGAAAGCCAGACGAAGGTCTCTGGCTTCGCTGGATGGTTTGATGTCCATTTTAAGGGGAATGCAGGTGAGCCGGCTGATCAAATAGTGGAATTAACAACAGCCCCGAGTGCAAATAAATCAACTCATTGGGGCCAGCAGGTTTTTCTTTTACACCCACCTTTAGATTTTGAATCTGGGGATGTAATTGAAGGCGAACTGGACATCACACGAGCCAAGGACAACCACAGATTGCTGGATCTCTCATTCAAACACAGATTGATCAAGTCCAATAGTAGTGGTGCCACGGGACCATTCACCTGCTCAAATTTCTACCTTGAATGAGGCTATGTATGCATTTTTGTTCCCGGTCTGGCCTATTTGCAGGTGCACATTTTGAGTGAATTTTGGGAAAAGGGATATACCTTTAGCTATTATATTGGTGAAAGTAGTAACTAGCTACTCATCACTGGCATATCCAAGGGTCCCTGGCTTTTCACGTATTCTTTATGGACCATCTGCTGTATTTTCGCTTGGGATTGCAGGTTGCTTCGTACCTTTCCATCGAGAATTGTAAAGAAGTATAACATTGCCGCTGTTTCTCGCGGCTTGTTGCATCAACAGTTTTTGCCAAGTTTTACCAACAATTTTTGCCTTGCCAAGTCTTATGGACAAAGTCTACCAGATTTTTACTTGTTGAGCGGCTCTCTAAGTTGATTCCGCTGAGAGTTTTGTAACTTGATTATATGGCACGTAGACCTAAAAGTAAATTGCAATTTTAGAACCCCAGTAGAGTTTTCCCCCCTGAAATCAAGTTTGGGTGAACGTATATGGTGATGCGAAAATTGCTGCTTCATCAAGCAGCTAGCATAAACATTGCGAGCAACGAGAGATGCTACATGGGAGATAATATGTGGAGTGCATGTAATTTGTTAGGTTGTGTGATGGAAGGCATGACCTTCTCATGCAATTTATTAAAAAGGAGTGTTTTGTGAATCCTGCTCTTTGCGTTTCATTCAAGGAAATATAGAAGACGCACGGGACAGGTGGTCGAGCG |
| **AT-HOOK MOTIF NUCLEAR-LOCALIZED PROTEIN 21 (AHL21):**  TACCTCGAAAGGATTGAAGACACAGCTCCAAGCACTAGCACTATTGGTGTCAAAAGGATGGCATCTCTATTGAAGCTTGGCTTTCAAGGTAGTTTGTGGGTCCTAGAGCACATGGACAAGCACAACTTGTTCACGCATAGCTTGAGGGATGGATCTTGGCTCTTAAAGACAAATGTAACTAGGAAGCTTGTGTAGTACATGGGTTTGGACCTTTTACCTTTATTTGTGTGATTAATCTTGCTAGGGGATCTAGGGGCCCAATGTTAGAATAACATCAATACGGTGGTTTTTGTGTGACTGGTCCCAATTGCTCTTGCTAGGGGGTATCGAGGGACCTAGTGCTAGAATAACATCAATGAGGTGGAGGGAGGGACACCTTGGGCCAAGGAAGAGTGACATGTGACAAAATAGAAGGATGTTAGTAAGTTAGAGAACGTATTGTAACAATGTTAAAGAAAGAGATAATTCTTGTTAAGGAGGAACTAGCTAGTGAAATAAGACATTTTTGGAGTGACATGAACAAGGAAGGGGTACCATGGGCCAAGCAAGGATAGGCATGATGTGGGAAATGAGAATCTTGGAGGAGGTTGGGAGGCAAGGTAAAAATTGCCATAATATTAGGTTAGGTTGGGGTGGATATGGTTTCGAAAAAAGACCTGCTAGGATCGGGGACGTGAAAGTTTGAGGCCATCAATATGGGGGGCGAGGAGGAGGCGCTATCGACCAGGCATAGGAGTCATGAGGCAATTGAGAGTTTGGGAGAAGGTTAGGGGGCAAGTTAAAAATGGCCATATTATTAAGGTTTAAGTTAGGTTGGGGAACAATAGCTGAGGAGGACTCACCAAGGCTAGGATGAGCATTATGATTAGTATTATTTGAGTCTTCGTTTTTATTTTGTAGTTGAAAAGGCTCTTCATTATCTGTGACGGGAAGCTGGCGGTCCGAGTCCTCAAGGGGCAAGCGGTCGTAGGAGGCGTTTAGGAAGGAGGCGGCAATTACGATGACGGGCCCAGCGGCAAGTAGGTTACCAACAACACTACCACCTATGACATTACCTTGGCCACCGGCTAAGTAGACGGTCAAGCCGGTAGCCCCAGGGGGGGCCGGATGTGGAAGAAATGCTCCCGAGAGAGAGAGAATTTCAAAGCTCCCTTGTAGAGTTACCGTTGAACCGGGTGCAGCGGGTTGTCGTAGCGTCACGTTGTTCACGGAGCCACTGCCGCTTAGAATGCAAAGACCACGATGTCGAACACGCGCAAAATTTGCGACGCACTCAACCACGTCACACGTGCTTGCGATCTCGAGCACATGTGCGCGCAATGCGGTGGCACTGTCCTTGCTTATGATGATGGGCGGCTTGGGTTTGTTCTTGGAGCCTAGGGGTCGGCCGCGTGGGCGCCTTCCGATTTGTCCGGCCATAAGCTCTTTGCCCTTGTTCCGTCTCTTTGATTTGTTGGTATCGCCACTCATTAAACCCCCGCTGCTTGGGCCGCTGCTCTCGCCGTCGTTGTCGTTGGCGTCGTCGTCAATATTGTTGTGTGCTACGCCGGCTTGCCGTCTCGCCTCTTCTTCGCTGGCAACCAAGGCCTGCCTTGCCTGCATCGCTGAGATCATATTGCCAACTGTAGTCGATGCCGTTTTCAATATCAATGTGGATACCGGCATGCTAGAGAGAGGGAGAGAGAGAGAGA |
| **KNOTTED-LIKE FROM *ARABIDOPSIS* *THALIANA* (KNAT1):**  TAAATGGGAGTATATCATGAACTAAAAGAAAAAGAATTCTATGGCTTAGGAATCAAAAGGCCAACCTGCACAACCTCTAGCTGATTAATTTGCAAATTGCACAATTTTGTATTGAGAGCGCCACGGGATTGATGTGTAAACATGAATAGTAAAGAGCAACATAAACTACAAAAGGGATAACCTTTTAATGCGAAAATTCCAAACTGACATAATGTTGAGAGGGGGAGAAAGAAAGGGAGATGTAGCTTGCTTTTATTGGGCCACTGAACACTCGGTTTGCTGCAAGAATGATCCTCAATTCAGTGTTGGGAGGATGATCCTTAATGCAGAGTTGGGAGGATCATAGCTGCAGCTGCGGCTGCTGCTCCCGCTGCAGCATCCATGGTCTTGACAACGGCATAGGAAGTAAGCGAATGAGGAGCAAGATGTCCATCTGTTGCAGGTGCATGTGCATGCAGAAGATGATGGTGATGGGCTCTTGAGTTGTCCACCATTACATACTGCATATACTCTGACGGTTTCCAGTGTCGCCTGCGCTGATTTATGAACCAACTGTTTATTTGCGTCTGATCAAGCCCTGTTGACTCAGCTAAGCCTGCTTTCTCTGCCTCAGATGGGTACGGCCATTTGTAGTGTTGGCTCCACCAGGCCAAGAGCTTCTGCCTTGCATCCTTGGGCAGTTTTCCTTTCTTCTTCTTCTTCAGGAACTCCTGCTTCAGGCTGCAAATGGAGCCGCTGTACTTGCGCAACAGCTGTTCTTTTAGCTTCTGATCCTCGGCAAGTGGGTCTATCCTCTCCTTGTGAAATTCGACCTCCCTGCAGCTGGCGTCTCCCACTTCTTCCTCCACAGACGACCCCCCAGTGTTGGCCCATTGTTGGGCATTGGCCTCCCCTTTCTCGTCACCTGATTGAGATAGGCGCAGGGTTCCCTTGCTCAGCGAATTCAACTGCAGCTCTATCATTCTGAAGAAGGCCATGGCCTCCTTATAGGGCTGCATCAGCTCGTCGTGATACTTCTGCAGCACGTTGCAATACGTCTCCATGAAGTGGTCCAACTCAGGGTCTGCACCCAAGGCTGCTGGAGAGGATGATCTAAAGGTCTGGTACTTCTTGCTAATCTCGTCCAAACTTGAAACAACTTCTGCGGGAGCTCCAATCTGCAGACACATACATGTCAAAAATCAAGTAAGCGACAGTTGTAATCATTGAGTACCCAAACAGGCTCTTACACAGACAGTAGCGTGTGAGATAATTCTAAGGAGTCTTGTAGTATTGCGACAGTTACATAATTTCCATGCTGTAGATTCGTTATTTTTGTCGATTATTTGAAAAAGGCAGGCAAATTCACAACCTTGTGGCAATTCACATACGCCATAACAAGGCATGGGTATGTTCCGTGGGACACAATTTTCGCTCTCATTAGGTCTGTACTGTGCTCTGCCGAGGAATGTGTATTCTCCACCTCCATGGAGACCCTCCCCACAGCTGCTCTACTTTCATGCATCATTGAGTTCGCGCTCGGGCTCACAAGCAGCATTGGAACACTCGTGGTAATCACATAACTACAATCACGTCCACCTACCAATTGGGCACCCTGCACGGCTTTGGGTGCCCCTACATCAGGCTTCTCTTCCTCAGTCCGGAAGATGACAGTACTAGGTTCGAGATGATCAAGCGCAGAGATAATGGGAGTGTGCGTTTCAACATGATGGGATTGGTTGAAAGCATGCGTTGCTTGGAGGAGGCCAGACTGCAGGCAAGGTTCGACGATCGCCGCATTGTAGTATGTGAAAGAAGGTGGAATCTGCGAAGCAGCGGAGACAGTATTGGAATGCAAAATACTGAGCTGCTGCCTCTGATCATTATGGTACTGTTGATGGGGAGATCCCTGAGTATTATGATGGGCAGGTTGATGCGGATCATGTTGGGGATGATGATCACGATGATAATCAGTTTCTGCACCTAGATCTGTGGTCATCAGCGCCATCATCGCCCCCACTTGGCCCTCGCCATAGCTCACCGCATTGCATAAGCCTGAATACTGCATAGCTGCCCTCATGTGGCTGTCATAGTCCATCTCGTCTACCATCCTATCTGTCCCAACTTAAGATCTGTGTCCGCACCTTTAATCGTTGCTTGTAGTCAAATCCTGCATGCTGCATACCCGCTGGAGAGGGGAGAATGAGATGCACGAGGAAGCATGGGAAGAGAGACAAGGGAAAGCTGGTATTACATTACTCTCTGCTCAAGGAAGCGACTGCGCATTTTCTCATCCGTTTCAGCAACATGCCTCCGTAATGTCCCCTCTTGCCCAAAAAAGCACCGTGGTAAGTTGCTAGTAAACGAGGGGCGGAGGGCGCTGGAGCGCCTATGTGCGCGCAGGCAGAGAGAGAGAGAGAGAGGAGTAGACAGCATATCGATAAGGCAAAAGTGTGCAGAGAGCAGCAGTAGCTCCTCATTTGAGAGAGAAAGGGCCTACATAGAGAGGAAAGACGTCCCAGAGAGCATTCTTGAGAGAGTGGCTGGCGTTGTAGCAGAGAGAGAGAGAGAGAGAG |
| **GLYCINE-RICH PROTEIN 2B (GRP2B):**  AGCAGAGCCGTCCGATGACCATTATACTGTGTACCAGGTGAAAACCCTAAATCCCAATTGAAAAGCCCTAACCTGCCTTCTCACAGCTCCCGTGCTTGCATTTCACGCGAGCCAGCCCCTCTTCTCTGCTCCATCTGAGCTTCGCGGCTATGGCCAAGAGGACAGGAAAGGTTAAGTGGTTCAACGTCACCAAGGGCTTCGGCTTCATCACCCCTGATGATGGCTCCGAAGATCTCTTCGTTCATCAGACCTCCATTCTCTCTGAGGGCTTCCGCTCCTTGGCTGATGGCGAGGAGGTCGAGTTTACCATCGAGCTTAGTGACGATGGCCGCACCAAGGCTGTCGATGTAACTGGACCCAACGGAGAGCCTGTCCAAGGAGGCGATGGAGGTGGTGGCGGTGGAGGTGGTGGCTATGGTGGCGGAGGAGGTGGAGGCCGTGGCGGGGGAACTAGGGGCCGTAGTAGAGGAGGGCGAGGAGGA |
| **MULTICOPY SUPPRESSOR OF IRA 4 (MSI4):**  AACTTGTTTCCCTTAAACCTACTTGGTACCTGTAAGTGGCAGTCTACTCGAGTTCCCGCCTGCGAAAGAGCTTCATAATCTGCACGAAGCTGAAGAGTTCATCGCGCTCTCGTCCTTCGCAACGAGAACTCTCCCCTGGTTAGGGTTCTTTCTGTTGATCTTGGCTCACTGAGATGCGGAGCGGTGATGGTGGTCCTGTAGAAGATCGCTACACACAATGGAAGTCCCTTGTTCCAGTTCTCTACGACTGGCTTGCAAACCATAATTTAGTGTGGCCTTCCCTCTCTTGCAGATGGGGCTCACAGTTGGAACAAGCGACATACAAAAATCGTCAACGCCTATATTTATCTGAACAGACTGATGGAACTGTTCCCAACACTTTGGTAATTGCAAATTGTGAAGTAGTGAAGCCAAGAGTTGCAGCTGCTGAACATATTTCACAGTTCAACGAAGAAGCCAGGTCCCCTTTTGTAAAGAAGCACAAGACAATCATTCATCCAGGAGAGGTCAACAGGATCAGAGAATTGCCTCAGAAGAGACAGATAATTGCAACTCATACAGATGGTCCAGAGGTGTTTGTCTGGAATGTTGATACTCAACCAAACTGCCAAGTTGCTGTGGGCGCAAGTGTTTCAAAACCGGACCTGACTTTGACGGGACACACAGACAACGCAGAGTTTGCCCTTGCACTGAGTCCAGCTGCACCGCATGTTCTCTCTGGAGGCAAAGACCAATGTGTAGTACTGTGGAGTATAGAAGACTTTACAACTTCAGTTAAGGACCCTTCACCTTCCAAGCCTGTGACACCAACAGGAGGCAAGCAAAAAGGTAGTGGGACATCTCAGTCCTCACTGGCCGATTCTACAAAGGTTGCACCTCGTGGAATTTTTAAAGGACACTCAGAAACTGTGGAGGACGTACAGTTTCATCCTTCAAGCGATCAAGAATTCTGCAGCGTAGGAGATGATTCATGCCTGATTTTATGGGATGCTCGTGCTGGACACGAGCCTGCATTGAAGATTGAGAAAGCACATGATGCTGATCTTCATTGTGTTGATTGGAATTCCCTTGAGGACAATCTGCTGTTGACTGGGTCTGCAGATAATTCGGTGAGGATGTTTGACCGTCGAAAGTTGTCGGGAAAGGGTCAAGGAACCCCAATTCACAAATTTCAGGGCCACTCAGCGGCAGTGCTGTGTGTCCAGTGGTGTCCTGAGCGGAAGTCAGTGTTTGGAAGCTCTGCTGAAGATGGCTATGTGAATATATGGGACTATGAAAAGGTGTCTCAGAAAAAAGAGGAGACAAAGCCATCAAAGCGTGCAAACTGGCCTCCTCCAGGGCTTTTCTTTCAACATGCAGGACATAGAGACAAGGTGGTGGATTTCCAGTGGAACCTTCTGGATCCGTGGACAGTTGTGAGTGTATCTGCTGATTGTGAGAAGACAGGAGGAGGGGGAACATTGCAGATTTGGAGAATGAACGATCTGATTTACAGACCAGAAGAGGAAGTGTTGGCAGAATTGGAGCAGTTCAAAAGCCACATACTATCTTGTGCAGATGAGAAATAAAGATTTGGTCTGTATGCTGAAGTGCTACTTATACCAGCTGAGTTTTGATCATTTTGGGAGCAGCTCTTTTCATGCTGTGTCCAGGCCAAGAGTTGTTCCCTTCAGAATTCACAAGGATGCTTTGGTTTTATAAAGAGATGACATGATAATGTTGTAAAGGCTTGCTCTCATTTGGGTCTCACGTCATTAGCATTCTCGGTCTACGGATTTCAATGACTTTGCAGTGGAAGTGTGTGCGTGCATGCATGAGCCCAGTTCAGAGGCCTGACTGTGTGCTGTTAACGTATTTGTGTGGTCACACGTGGGTGCATTGACCTGTGCATGAGTGTGAGTTGGTGTACGCATATATGCTTTCATGCATGTGCACATGAGTGGATGTCTGGGTGTGTGCATTGCGAAGGTAGATAATCTAACAGTTGTGTATATGATCTAGACATTAAGGTTCCTGCTCATGACAATGATTTAATTAAAAAGATCTCGAGTGTGCATGGTGTACATATGCCGATATGGAAAGATACTCTCACACTGGGGTTGTTTCATGTGGGTTACTGATAGCTTTTTTACGTTTTGGCAAATTTTGGTACAAGTTGAGCAACTGGATGATCTTCACAAGTATGTCGATGACAAATCACTGTGAAGCCAGATTACGTGGTGGTAATTTTCTCTTGATATGCTGTGAAGTGGTTTTCTATCAGTGACTTGGGCACCCTTTGCAAGGCTACTATCATGGAGGGCTCTTGCCTGATAGATCTGTGAGGCTCTTGCCCAAATGCATTTGTTTCTACGATTGGCCAATATTTGGAAAGGGTGATACCTCCTCGTGGTATTTGGGTGTCTGTAATGGCTGTTCGGGAAGGACTCACTCTCCAAAAATAGAAGTGATTTCCAACACTTGTTTCATAGAAGATCCTAACTGCTGTTAAATGCAATGTTTGTATTTTTTGAAAAGTGAAGCTATGCGATTGATGACACTGGCTTTCTTGAATTAGGAAGCCCATGATTCCTCATACTCGGTTTCAACTTGGGAGCTATCAGTGCTATCTAACTGATGTAAGAATTTTAGGCTAGAAGCATATCATTGCAAGAGTTACATAAAAATGCTAAGGTATTTTCAAGAGAAAAA |
| **ENDO-BETA-MANNASE 6 (MAN6):**  TCTCTCTCTCTCTCTGTGTCTGTGGGGAGAGGCCATGGCTTCTTCTACTGGCAGCTTCCCTACCATGTGCATTGCGTTCTTGGCTATATTCGCTCTCTTTTCTTGGTCTTGCAAGGCTTCTTACTACCCAGGCTCCAACTTCAATAGCTTTGTGCAAACAAGAGCATCCCAGTTTGTTGTGAATGGTCACCCTTTCTATGTGAATGGCTTCAATGCCTACTACCTCGCCTACGTTGCTGTCGATTCACGCGACGAGGCTTCCGGCATCCTGCAGCAGGCTGCCGGCATGGGCCTCACCTTGTGCCGGACATGGGCATTTAATGATGGAGCCTACCGTGCCTTGCAGGTCTCTCCTGGAGTCTACGATGAATCCTCTTTCAAGGCTTTAGATTTTGTGTTAAGTGAAGCCAAGAAGAATGGGATGAGGGTGATATTGAGTTTGGTGGACAACTACCCAAACATGGGGGGAAGAGCTCAATATGTGCAATGGGCAAGAAATGCCGGGGTCTCCCTTTATGACGACGACGACTTCTACACTAACCCCACCATCAAGGCCTTCTACAAGAACTATGTCAAGTATGTGATCACAAGAGTGAACACTTTGACTGGAGTGGCATACAAGGATGATCCTACAATTTTTGCATGGGAGCTCATGAATGAGCCCAGGTGCCCGAGCGATCCATCTGGAGACACATTATATGGTTGGATTCGGGAGATGTCGTCTTATGTGAAATCCATTGATTGCAATCATCTAGTCGAGGCGGGGCTTGAAGGCTTCTATGGGACTTGGAGTGAGGATAGGCAGCATCTAAATCCCGCAGGGATGTCCCTCTCAACAGGGACTGACTACATTAGGTTCAGTCAGATCTCTTCCATTGATTTCACGACGGTGCACTCCTATCCGGACCAATGGTGCGTAAAGTCCGTCTCTTGACTCAAATTCTGCATGTTGGGAGTATAAAAGCGCCGAACTCGCATGGGCAGTCGCCTGAGTGCGTATTTGGTTGTACTCATTGTCTGATTCAACTGAAGATATGTGTATCCTGTACTAATGTTTGTGATGTGACTAAATGTGTCCCTAATTGTGTGGTTGGTATAAACAGGCTACCCGGGCGAAGCTTCTCTGAACAATTGGCATTCTTCACAGCATGGGTAAGTGCCCATGTGAGTGATGGGTTCGCCGTGAAGAAACCTCTTTTATTTGCAGAGTTTGGACTATCCAAGAACAGCCAGGGCAGCTACTCGGACGAGAACCGTGTCACTCTCTTCTCCGCCCTCTATGCCTCCATCTACAGCTCTGCCATGACGGGTGGACCGGCAGCAGGGGCGCTTGTTTGGCAGATTTGCACTCAAGGTGTAGAGAACACCATGGCCACAGACGGTTACGCTGTTCTTCTCTCTCCTGATTCCCCTATCACCTCCCTAATCTCTCTCCAATCTCAAAAGCTCACTGCTCTAAACCAATGAGCCACACACAAACAAGCACACATAGAGACGCACTCTTTTTGCGTGCGCGCACACGCGGAGCATTGACAATCACTTAGACACAGTAAACACTCCCACACACACACATGGAATCAAAGACACACCCATAAACACACACACCATCAACATTCACATAGATGCACATATCAAATTCAAAAGTTATCCATGATCCATATGCAAATGTATACACAGAGGTGATGGCTGCAGTGAATGTGGAGTACTATAGAGAAGTACACGGCCTTACACACATACACACACATGTCACACACACACACAAGAGAGTCCTTTTGATGCATCCATCCAAAGCAAACATCACTATCAACAAGGCCCTAGATTGTGTCGTTGATAGCTCCGACCATCTAGATTACTTTCTATACACATTTCCATTAGGCTTTTAAGCTCTCCTTTTTCCTAGCACCATTGTACACACACAATATAATAGTGGCTACTTCAATTTTTTGAAGAATAACTTAAGCATATAACCTTTTTTTGTAACTTTTTCCTCACGTGAAATTTAATTATGTACATGCTTCCTTTACACCAAAGAAGGCAAGTTCCACAAATTAAGTGTGTAAACAAG |
| **JUMONJI DOMAIN-CONTAINING PROTEIN 22 (JMJ22):**  TTGCTCGACATTAAATAGTACTTAAAGCCTTTTCTTAAAAATCAATAAAGAGCTGTTGAGGAAGATTTTGGTGAAAAGGAACCTCCGTATGAAAATCTTAGCAGTTGCAAATATTGGCAAGCTCAGCATACTTGCGTTTGAGAGTGTCATCTAGACCAAAATGAACTGATAGCATTCTTTTCAATTACTGTTAGAATTGATTGAGAAAAATGAGGCACTACAACTCTTTCCCAAGAGAAACAGAAAAGGAGGCAAGCCGTATGGCAAACGCAGCATACATGCATTTGAGAGTGTCCTCTTGACAAAACGGAACTTCCCCAGAAATATGGAAAAGAGAGTCAGGGCATACTTGCAGGGCAAGCCCATGATTATAGCTTGAACCCATCAACAAGTACAATGGTCATTTGATTCTATAAAAGTCTGGAGTGAGATGGACATTTGAATTGGTGCCTGCAACACAAACACACACCGACTTGATGCCTTCAATTCCTTAGGGTTCCGATTGAAGTGGATATGAGGAACCTCCTTGAAAGATGTCATCTTCCATTTTGCTGTAAGAAAGCAAAGGCTACATTACTTAGCGTGCACATACTCCACTGTTATCAGCAGAGTTAATGGAGCTGATGAGGCTGAAATTGAATGATTTTTGCCTTCGGTGATGTTCCTTTTGAGTTTGAAAACCTTCAGGAAATACCTGCCAGCACCAGCTATATCAGCAGGAAGGGTAGCTAAAATGCCAAAAAAATGGGTATGATAAGACCCTGCACACCAGCAGAGATACAATAATGTGTGAAGGCATCAGAAAACGCATGAATTTCTCAGCTGATTTTTCAGGAAAGCCTGGAAGTAGTAAGGAAACTCTCAAAGCACTGTCATTATAACCTGTTAATCAGAAGCCGAATTTAAACCCACCAACTTTAACGTCTGTTACCATCTCCCAAAACGAGACAGAACTTCTTTGCTTAGCTTTCTCTGTAGCTTTTCTTTCCAGTTCTTCAATGGAACCAGGAAATAAATTGTCATACATGCTCTTGAAATTATCATACAAGTTGACTCGGTCTCTTGTACCCGATACCAGCTGAGCCGAATTTGGCCGTTTCAGAAACTCCAAGACATTGAGAAGATTGCTCCTGCTCACATAATTTTGTGTAATCGCAACAGAATCCTCCAAATTGATAACCAAATGCCACCATCCATTTGGGACAAACATCACTTCACCTGCTCTGCATATGCACTCTACAGGTTTTTTCTTCCACTGTTTTGTCTGCTCATAGAAGTTCATAAACCATTCCATTATTGACACAGGAGCAGCCACCTCGGCACCATCTGGACTAGGATGCACACCTGGAGGATTAACATCCGGAGGGAAAAGTATCCACTTTTTGGCACCCCTTACCACAGCATTCCATGCAGATGTGCTGTTGGGATCAATGTGAAAAGAAGAACCTGATCTAGCAGGCCCAATTATAAGCCAACGAAAATTAGGTCTGTCCTTCCCCAAAATAGAGAATAAATCCTCCCTGAAGTAACTTGGGACCACATAATCTTCATTTAACACTGGCATTTTCTCTGCAAACTGAGGATCAAAAAGGTACAATGGGCGCTCTTCACAGATAGATTCTGAATATGAAAAATAATCATCCATTGTCATTTCCACGGGACCTACGGCAAGCTTGGAATCTCCTGCTGCTTTCACCAGATACTCCTTGTTCCACTTCGTCAATGCTGGCCAGTTAGACAATGCATCTGTTATTATCACTGGTTTATTTGGCTCTTCATAACTGTCGACAAACTCCTGCACAGAAAGGTTGGCCCGCCTATCAATGTTGTCCACCAAAAGCCATTCTTCTTTCATTTTCAAGCTGCTGCAGAGCCAGCTCTGAAAGAGGTAGTCTGAGAAAAAACCTGTCACTTGTAGACGAGGCTCCGGATAAAGGGAAGAGGATGACGTGGCGTCGGCGTAGGTGCATCTCCAGTCAACTTTAAACCTAAAATCGCCTCCGAACTTATCCAACACAAGATTTCTCCATAGGAAATCCTGGTGAGAAAAGACATACAAAGCTTTACTCACAAGAGAAAGGTTAACCAGATCGATGGCTTGCAGAAACCCTAGAATTTCACACAGGGTTTCATCTCCGAGGACATGGAGGGAGCCGAGGCCTGGGTCTCTGATGTTGTGCGCGAAAGCGGAGAAGAGAAGGTTTCCAAGAGGTTGGACCCCATAGCTGCAAAGTGCTCTTTCCAGCTGCAGGCCTTCACGCTCATCGTCATGGGTCGCCATTGATGAAGTTTTGCTCCTCTTTAGACTGCAGTCCTCCATATTGGGTCTCTCTTTATTCTTCTTCTTCTTCTTAATCTTCTTCTTCTTCTGATGCCTCTCGCCTGTGCTGTCATCTCCATTGGAGCCCCATTTCGCCATGTCTTTGACCCTTCTCTTAGAGAGAGAGAGAGAGA |
| **WUSCHEL RELATED HOMEOBOX 13 (WOX13):**  GATTCACAATCCTGTAGTACATGCTCACCAAAGATAATTATACTACTGCCAACACATCATTAACCGCATTAACTACTTATCACATCAACCCTTGAGCCATGTTACAGGCAGTAATCACAGCAACATCTTTCATTTCCCCTCTTACTTGTATTCTGGCAAGATTTGGGCTGTGGCCTTCCTTTCTTCCACTTTAGTGCCCCGCTGACGATAGAAATTCAACTGATTTGGAAGTCCCATTAGAGACAGACTGACTTTCCTTCAAGCCAGCCGTAGGCGGTCGTAAGCATTGCCACTCGGAATTATCCTTCGACTGTTGCACACCATGGACATGCTCTGTAATCTAAAGAGTGGCAAACTTCTTTATTAACAGTCTGTCTGTTTCTCTTCATCTAATCTGTTTTCCTTGAGTAAAGTACACACCCATGTGTGATAGAATCGCTTTCAGATAATGATAACTATACAATGAATGTTTAAATTTAACTATAAATCCTTCCAGTTAGAAGCTGGTTACAACACCACCTGAAGGGCATCACTGTAAAACTTGTCAAACTACAAAAAATTAGCACTTCCAGATCTAGCAGGTTGTCTCAACCAAGGATTTGTTCGAACTTCCAAGATCACCACCAGATTATAGAAGCTTCCTGGATATTTAGAATTGACTGCCTTGATCCGGAAAGCCTTCCGGACTACTATCTGCAGGCTTGGAAAGATTGAAAATCTTAAAACCAGCATACAAGTGCCATTGAAACTAACTTTTGTACATGGAAAAATGTCGGATCTTAATTGAAGTTATTGCTTAGCATTTGGTTCCTGAAAAGCCTCATCGCATGACAATGTTAATCTCTGGAGCTTCAATGGTGGCGACTCTCTGCCCCAGTAATGATCATCTGCCGTCCTTGAAATTCTTGCAACACTGAAAATCCAGAAAAATCTGCTTTGCTTTCAGGCTCAGTCATGGGAATGTCCCACAGTTTACTGTCGACAAAACGGCTGGCTTGCATTTCACCATGCCCATTGAATGCCGATTTGTTGAAAGAAACAGCAGGGCTACTAGCGTGTGCATGCCTTTGAGATAATTCAGAAAATCCCTGGTAATGCTCAATGTTATCAGCAGCCCCGCTAGTCCCTGCAGCATCCAAACCGTCATTTGTGAAGTCTCCCTCGGTCTTACCCTTCTTTTCTTCTGGAAAATCAACATCCGTGTCAAGCTCAGAATCTCCATTGTTAGGCCCACCAAGCTGCTGTTTTCTCTTGGTTCGGGCTCTTCGGTTCTGAAACCAATTGTATACATTGGTTTCCGAAATTTGGCCATGCTGACTAAGCTCTATAGTTATCTCCTTTATTCTTTGCTTATTGGGTGTGCCACTCTCCTCCTCAAAGAGCCTCTCGAGAATGTGAAGCTGAGTTTGACTCGGTGTCCACCGCTGCCGGGATGTCAGCTTGTGTCCTATGGAATGCAGAGAAGAGTCGAAGGGGATAGATGGGCCTAGCCATAACCCTGGTGTGGAGCCTTGCTGCGCCATGATCGCCTTGTGCATTTCCACGAGCTGTTGGCAAATGGTTGCATATACAGAGATCTGTCTTCTTAGGGTTTCCAGCTGTTCATCAGTCATAACTTGGCCATGCGGGGTTATGGGGCAAACTGGGTGATGTTGGGTTGTGAGCGCATGACTATCAGCGGATGATGAGTGCTGTTGGTTTGCAAAAGCATGGAATCGCATGTCATAGTGCTGCTGATTGGCAAATGCTTGGATGTGCATATCAGGAGACGCATGCACCCCATGAAAGGGGGTCGCGTATACACCGGTATTCGCATGTGGCAAGTTAGCCTTGGCAGACAAGTCCTCTCCTGCTCTATTTTCGAGCTCCATGTGATTCAACCCTCTTCAGTCCCCCTCTGATTCTAGGGTTCGCCTGTCTTCATAGGAATGGAGACCATTAAGCAGCTCTCAGAGCAAGAGGCGTGTTTTGGAGGGGGACTCAAGTGGTGGGTGGTTAGCGAGGAAGTGACATTTGCGGCAGCGCATGTGGGAGAGATTTGGACAATGCGCAGTGAGTTCTTTTGTGTGTTTGCCGTGTGGGTGTGGAGAGCTTA |
| **FASCIATED STEM 4 (FAS4):**  AATGGAAAGCTTCATAATAGAGAGAGAGAGAGAGAGTGTGATTTCCAGAATCTTTAAACACCTTGCTTTTTCTTCCTCTTTCCATAAAGGCTCTTTCCATCTTGTTGAGCTTCAAGTTTCAACTTGGTCCAAAGCCTTTCCAACTGTGAATGTGAACTCTTTTGTACCCATAACAGAATTTCCTGGTACAGAAAGTCACTATGAGTATCCCACCTACAGTTGAGCTTGAAGCGGCTATCCACTGATGCAGCTTCTAAAGCATGCAGCAAATCACCGGAACGTTTGTGGGCATTTCCAGAAGGAGAAACTAAAATTAATGGGTCTGCGGCTAGGTGGGGATGCAAGGCCTTCAAGGCAGGAAGTACTTTACCTTGAAGAAAGGCACATGCAAAAACAGCAATACGATGTTTTTTGCTTTTCAAGGGAAACTTGTGTAATGGGAGTTGCCACAGATGAGGACCAAAGCATGGGGACACCCAACAGGAAACATCATCAGATAACTGATCATACCATGGTGGGGGATCTGAAAGAGGCTTTGAGAAAGTACATAAAGCATTTGCATGCATTACCAGCCAATCTGCATTGACACTCGTCAGAACTTTCATATAAGGCCTAGATGTTCTCACAAGCTCATTGAACACCACAAAATTCGGTGCATTCTTCCTCAAGGAGGATGACGGATGCACATACACTTTCTCTTCTGTATTGCAAGTTTGATACTGAATTGTCTTGTGTTTTTTCTGTTCACCTCCCTCTTTGCTTTTCAATATTTCCTGCAAACTCACCTTATGCGCGACTCTATCTGCCCAACCTGCACAAATTGCTTGCCTCAACACATTCTCATGCTTAACACTCAATTCATGGTTCACTCCAGCCTTCCACATGGTTTCTACATAGGCTACGGAAAGTTCATCGCAAAAAGAGATGCAATTAGTTCCTCTTTCTTTACCGAACACAAGATAAATCTGTACAAGTTGTTTTCGCAACTTCGACATCTCATATAAAATCCTTGCGTGCAAGTGGTTGTTCCTGCAAAATTCCTCTGGATCATTGGAGACTTCATATGCACGCAAAGCCCTAACCACACCAAGAGCATCACTAAGGGGATGGCTATAGCTTGACCTGACCTTTTTGACAGATGTTCTTAACTCTGTTGTTTTCAATGCTGCATCTTTTCTCTCTGACTTACCATCAGCATACATTCTCTGTGACTTCTTTGATTTACTAACTGATTTTTCTATAGTTTCTTCATTTGGATCCCCTGGACGAGCTAAGTCCGTCACAAAAGGACTATCCAAGCTTAGAGCTGCTACTGTTGCCCCAGCATAAGCAAGCAAGATGCTTGTATCTTTAATATCATCTGAGCATCCAATCTGCATTGCAGTAAGCAACATTCGAGAATGACGAGGACTGATGGGATACAATGAGATCGCTTTCCCCAGAGGAGTCAAAAGGCCAGTGCTTGAGTCAAGTGCTGATAATGCATGCAGACAATGCTCAGCCTCTGCAAGATCTGTTTTATCAGGTTCAGACAAGAAAGGGAAATGGCTCACCTTGACAATATTCATGCTCTTTAACAAAAGAACAAGGCTCTCAATGGGAGCCCGATATATCTCTGGCTGAGAGAAGTCCCGGAACGTGTCGTTGAATATAGCAGATGAATATAGGCGATAACAATGCCCAGGGCCTGTCCGACCCGACCTACCAGCACGCTGGGCTGCAGATGCTTTGCTTATCCATCCGACCTCATACCTTGTAATACCGCTTGCACGTTCAAAAATTTTATCCTTGACACGGCCACAATCCACCACATATCTGATGCCCGGAATAGTGATTGAAGTTTCAGCGACATTTGTGGCTACAACCACTAGTCGAGATCCCTCAGGAACAGCACCAAAAATCTTCAGCTGTGAGGCTGCTGGTAGAAGTGCATAGAGGGGCAGAACATACATCGGGCCAGCTACTTTTGCTGGTACCTGCTTTTCGACCACAAATGGCTTCTCCAACTCTTCCCTCCTTCCCGTGCCAGCAAAAGAGTCAAATGCATCTTTGATAGCTTCCAAGCAGATAGAATTTTCTGACCTCCACTCTGAATCACGCTTTGGCCTCCGTTCCTCCACTTCAATACCTTCTGTTTCTGAGAACTCCAGATCACTTTCTGAACTAGAATCAAGGGGTGATTCATTGATTTCCCAATTTTCCACCATCTCTGCTTCATCATAGAATTCATCCTCTGCGTAACTGCCATCAGCTGCTTCTGAGATATTCTTAAGATCATCATTATCTCCATTATTAGGTTGCTTTTCGTGCTTCTTTACACCTTCCTGTTTTGGAGGAAAAGCTTTCTGAAGCCTCTTGCACAAATAATGAACTTCTGCTTGCCCTGTAAGAAACACAAGAACACCTCCAGGAGGAAGAAATTTATGTATAGAGCAAACTTTTTTGTATGCTTTGCCCACATAGTCCACAAGCTCTGTTTTAGCAGAGAAGTGGACTGTAACGGGAAACTGCCGTGCAGGAACTTCAACCACAGGGGGTGCACAAGCAAACAACTTCCTGTTTGCCACAAACTCATCTACAAGCAAAGTAGCACTCATTATAACAAGCTTCAAGGGGTATATGGAAGCTTTGCCTGCCCTACATTGCTCTTCATATAAGCTCTGGCGAAGTGGCAGGACTCGAGACAGCATACCTATAAGAATATCTGTATTTAAACTCCTTTCATGTGCTTCATCTAGCACAATAACTGAATACTTTCTCAGCAAAAAATCTGACTGTACTTCTCTGAGAAGAATGCCATCTGTCATAAATTTGATGTGGGTATTTTTCCCAGCTTTTCTATCGTGTCGCACTTGAAACCCCACTTCTTCACCTAATCTAAGGTTCATCTCATATGACACTCTCTTTGCTGTTGCGAGAACCGCAACCCGGCGGGGCTGTGTCACACCAATCATTCCTGCTCTCTCTAGACAGGCACTTGAAGAGAACCCAGCCTCGTATAAAAACTGAGGAACTTGAGTAGTTTTACCACAGCCAGTCTCTCCACATATAATTGTGATTGAATTTTCAGCAATAGCCTCCATAATCTCTTGCTCCATCATTATAATGGGAAGCTTTTCTCTCGCACTTTTTACCTCCTCTGGTCTCCAAACATGTACAACTACTTTAGAGCCATTATCAGGAATGCCTCCACAGGCCTTAAGAGCATCACTATCCTTGGTGATAGTTCCCCCTTGGACCGTCTCAACTTTAAACACATCATCTCGCATGATGTCCGTATTATTAAATTCTGCTAGCTTTTTGCCCTTTTTCTTTTTCGTTTGTTTGGATGCAACTCTTCTTTCCCTTGAATTATCATCATTAAAATTTTCATGCTCTTTAAAATATTTTCTGGCTTTCCTTGATCTTTCTTCAATTGAAAAATTATTATCTATGCCAATACTGAGATCCGACAAAACAAAACCAAGTGTTTCTGTGCCACCTTCAGTTTGATCAATAATTGCTGGCTGACTTGTGGTGTGCTGCACTTTCAAAGCATGTTTTGTATTACCCCATAACTCTCCATCGCTTTCACAACTTTTCATGGGAAAACTATTGCCTGCTTGAAATTCTGTTTGTTTCT |
| **SWITCH1 (SWI1):**  GAGTCAAGTGATACAATGCTACAATATATCACGGGCTTCATTCATGAAATGCAAATAAAAGCAACCGTCTTCAACAATGCCAAACCAGAAGCAAACATTCTGAACAAGTTAAGACCCAGTAATTCTTATCGTAAAAAACATAAAAGAAAAAGAGGACCGCCACTGCTTGTAAGTTCGCCCATCTCCGAAAAGGAAGCTTTGCAAGGTAGTGTATCTTGACTGAGTACTTGGCTTTTCATATTAGAATGTGTACAATAGATTTCAAATTTTCTGCGCATGTGAACATAAAATCCGCGTTTGTGAATGCTTTGCAGACTCTTTCATATGCAAAAGCATCTATGAAATTGAGCAGAAAAGCCTGCCACCAGATGCATCTCAGCAGCTGAAAAATCTTCGTGCTGCCCTGGTAAAAGTTGGGTGGTGTACATCTATCCTCCTGAAAAAATGATGTTTTAACTGTTTTACAACAAGGAGGATGCACGTTTTTGCAAACGTTTATGCTTCACTGCAAAAAGTTTTTATTTGCGCGGTTGCCTAGCTTTGAAGATTCTAATAAGCTGGATTCAACTGATTAGTCCAAACAGCTCCTCATAGCCGTTGTTGAAATATCATTTCCGATCGTTGTGGGGCATTGAGTGCAGGTCAGTGAAACTACAAAAGAATATGTCACCATTGCGTATCCAAGCATGGCATCTTTGGCAGAACATTTTGAGGAATCGAAGGTGTTAGATGGAGCTTCCCAAGAAAGAATAATTACAAAATGCCTTTTTAATCAGCTTTCCACATCCACTTGCATGCCAAGGTTTGATGAAGAGTTTTCAATGACACACGAAACTGCTCAAAGAGTGCTCAAGGGCCTTATTTCTTCCACACAGTGTCTCCGACAGGGCCATCTTGAATCATTCTGGTTAATACCAGGCCTAGACACCACCTGCAGAACGACAAACACCCAAGCAAATAAGGACGAGAACTGTTGCAATGCCAAGGAAATTGCTTCATTATCCCCAAACCACGATACCATAAAGAAACATCGACCCTCTGAAAAGAACTCCCCAAAAGTGCTCAAAGTCTACACACACAAGAAATTTTGGAGGGGAAAAACAAAAGTCAAAAAAGAAAAACTATCTGAGAGCGAATCTTCTGGCAAACAAAATGAGCATAATAACAGCAGCACCCAAGAAAGGGAGGTGCCTACGTCATTAGATGATGCTGATCGTGAGGAACCCTTGCTGCATACAGTTGATAGCAACCCTGGGTGTGTTTTCACAGAAGATCATGAGAAAAAATCACAGAACCATGATGCCTCAGAAGAAAACCAGGGCATTGTCAGATGGGGGGTCCGGAAAAAGATTACCTCTAGGTGCCGCAGTAACACCTTTAGTGATGATTCCAAAAGTAAAGGACTCTATTGTAAAGAGGATATGCACAAACAGAATCAGCACAACGTAGCAAAAACAGGTCACGGGTCAAATGCATATAAGCATGTTGCGGTGACACGAATACTTACAGGCATTGAAAAAGGAAAGATGCTCAAAGTTCCCAAATTAATGGAAGGAAGATGGTCTACTGAAAGGTTTAATCTCGATCCCTTGGCAAATTGAGTTACCCCTCATGAGCAGAGAAAAATTAACTCATCTGGTGACAGCGAAATTGAACTCCTGAATGTTGTTGCAGATATAAAGCAGCTCAACTCAAGCTTTTTGAAATTATGAAAGACAAGGGTGCAGTTCCTGGGAAACCCCTACTAAGGCCTGCACTGCGTGTAGAAGCCAGAAAACATATTGGAGATACAGGACTTCTTGATCATCTTCTAAAGCATATGACAGACACTGTGATCAATGATGGTGAGCGCTTTCGTCGTCGGCACAACTCTGAAGGTGCAATGGAATACTGGCTGGAGGATGCACGTCTACAGGAGTTGAGGAAACAAGCAGGTGTTGACCAGTACTGGATACCCCCTCCAGGATGGAAAATTGGAGACATGATAGCTGACTTGTCTAAGCCACATGTAAACTCTTAAAAAACTGTCAGCTTTTTTTGAAGAGAAATTTTTGTGATCCTTCTTGATAATGCCTCAATTTGTGAATTTCCAGTCAACTACAATATGTGTGTCTGCATGTCGGTGCCAGCCTTTTAGAATCAAGAAGTACATCTGTTTTCAATCGCGAGTCTGACTTCAAATCCTTTTTCTACAAGGCAGCAGCTCGACCGGGGAGCCTTGGAAAACCAGGAGAGGTTATATTCACGAAGGCTAAGCAGGAGATTTCAGAGAAGAAGAGCCCTGTTTCTATTGTCTTAAGGAGGAGCCCTCGTTCAAAGGAAGCTGAGAATAAGGAAAATGTGTCTCCAATGGGGCAGCAGGGCGAGCTCATCAAGGCCTTTCAAGGTTGGAAGAAAACACAGGGCTTCTCTCAAAGAGAAGTTCTCTCTGCAAAATCCGTGAATATGGCGAGCTCACTGTAGTGCAATTCTCGGACAAAATAATCTTCGGAAGATTAAAAGAATTTAAGATTGTCGGCTTGTACTAGTACTTGTGTTGCATTTTGAGTCATCACTTTGATTTAGTAGAGAAGAAGAAGAAGTTC |
| **SLOW WALKER 1 (SWA1):**  TGTTGATAGTGTAGACGAATTTTCTAGCGAGTGTCGGGGCTCGAGGTATAGTGATAGTAGTGTTGCAAAAGACTATCATTGAATTTAAAGCAACATTTGGTGTCATCGTTGGATGATTTGCCTCAATGTGGCCTTGCTCAAGAATCATAACAAGAATCCTTCATCTAGGTAGTCCACAACACAATTAAGTGGGCTAAATCCCTCTCTAAGAAAACAGTGATATCGTCCAAACATCATGTGACATTAACGAGTTGATATTGGATCATCCACGAGAATATGAGGAGCAATGGCTTCGCAATACGCCAACAATCCACTTGAAAATAAGTTTGGTGCCCAGAACACGATAGCATTCATCACTGAGGGTTAACAAGAGTGTCAAAAAGCCTTCGTACAAGGGTACTAGAATTGAAATGCATGACGTCTCTCTCAAAGGGCCAGTGTTGCATATCCCTTAGTCAGCTACCATGCTAGTCCACTATCACCTTGACATGCAGCGAGTTTTGCACCTCCACCTACTTGGTCAGTAAAACCAGAGATCTCATCGATATAAATTGCACAATGTGTAGAAGATGAGGGGCATCCTTGTCTTAGCCCAATGGTGTTGATAATCTCATATGATAACCATTGAGCACTGCATTCTCCCCACTCCTAGCTCATACAGACTAAAGCAAAGTAGATAGTTCCATGGTGCTTCAAATTACTGCTAATGTTTATTGAACAGATGCCTGAATCAAAGGTTGAACCAAGCCTTGTAGCACTTGTAAAGACTCCTGCAGACGAACTTCAGTTTCAACGTTCTCCCTCAGTACTGCCACCTGATGTAGAATTGATGGAGAAAGAACAAAGTTGCCAGCACATTTGTGAAGCACCTTGTGAGCAAAAGGAATTAATATCCTTGAAAATTTGGGCAATGTCACATATCTCCTTAGAAAATCTAATAGCAATTCCAGCGACGTGCTATCCAGATTAGACACAGCGGATATCAAACCCCGGCGAAGAATCAATTCTTCCATAACAGCCAAAACAACTGTTGGGTTCGACATCTTTAGGGATGAAACAAGAGCTTCTTTATACTGGAATTTGCGGAGATATATATCATGTTCAGCAAGCCGAGGCCTTCTTTGTTGAGCTACAAAGTAATCTCCTTCCGTAGCCTTCTCTGCACGACCTCGCAAAAAATAGCGGTAATTGCTGGGTCGCAAGACAGTTTCTAATTTTGGTTGCTCTTGGAATATGCCGGCAGCATTTTCTGTACTCCCTTCTTCATCTAAAGCCACCTTTTTCTTCTGGCGAATAAATAATTTCCCATCTGACATCCCTACAACCATAGTTGCCATTGATGGGGAAAGGTCCATTGACATGATCGGAGAATCGTACTTTGATGCATGAACCACCTTGAATTCATTGATATCAAAAACCTTGACATGGCCATCCAGGGACCCAGTCAAAAGCCGCCGGGAAGTAGCAATGTCACTATGTCGGGCTTTGATGGGCGGGGTAAGGCATAGGCATGTTACTGTTTTTTGATGGCTGGCAACTGCATGCAAAAGCTTTCCGCCTCCCAAAACATCCCAAATCTTCACAACATTACCACCAGCAGTTGCTATGAGCCCTCCAGATGGAAAAAACAAAACGTCTTCTAGAGGCTTCCCATGCTGATGTGCCATGACCCAAAACATATACCTGCAGCTGTAGTACTGATTTTGCCGAGCGAAGATCCCACAAATTTACTGTATGATCATAGGATCCTGTAGCCCAAAGGTCTGCACTAGAAGGGTTCGCACATCCACTTCGTACATAGTCGGAATGACCTTCTAACTTCAATATTTCAGTCTGAGTTGCAACGTCCCACCACCGAACTGTATTGTCATCGCTTCCTGAAAGTACATGTAGCTTATCTAAAGGGGAGTACGTAACCCAATGTACTGCCCTTGAATGTCCCTTTAGTTGGCGAAGCACAAGGCGACTATTGATATCAAAAACCTGGATGATCCCCATTTCACCCCCAGCAACCACTAGTTGCCCATCAGATCTAAAAACGCCAGAATATGCCACATCAGAGAAGCGTGAGATCGTCTTTTTAACTTTACATGTTTGCCCATCATAAATCGAAACCCTAGTGGAAGAAGTAACAGCAAAGTCGTAAGGGGGTTCGGGGCAGAAGTGAACGCAGGAGACGGCAGCGATCTGTTGGAGAATTTGCTTGGAGGAGAAGGACTTCCAATACTTGGACTCCAAAGCAGAGGGTTTCCTGGAGGCCGCAAACCTCTTCGCCCTCACTGGCTTGTAGCTGCTGCTTTGCACTTCCATTCTCACGCGCGCACGCACACAATAACCCCCTCCCGCTGTGTGCAGCGTTTAGAGCTAGCTCTTCTAAAACCCTCTTCCAAATTCGAAA |
| **BRI1-ASSOCIATED RECEPTOR KINASE (BAK1):**  ACACACACACACACACACACACTTACGCCCACAGCCACAAGGAGAATTGTTCAAGAAGAGGAAGCGCGTTCTCCTTCATCATGTTTCTATAAGCAGATCCTCATTCAGAGAAACCTCGCAGGTCTCTCTCTCACACACACACACTCACACACACAAACACACACACGCACGCGCGTGCAGAGAGAGAGAGAGAGGGTACCTTTCTTTCTCTCTTTAGTCCTGTTAGCTCCACCCGCAGAGAGAGAGAGAGAGAAAGTAACTGTGTAGCCTTATCAGCTCCAAGCTCCACGGAGAGAGAGAGAGAAAGAGTGCCCATTTTCTTGAAAAGCCCCTTTATCCCATGGCTCCCTCTTCTGCTTGAATTCTTCCCCGCTCTCCTGCAAGCCCATGCCTGCCCCACAGTCCGCCCTTGTTGCTGCCCATCTACCGTAGCATTTTCAAAAGGCTTCTCGACATGCGGGCTTCCTTCTTCCTGCTGCTGCTTCTCCAGCTCCAGCTTCTCCCTCGCATTTACTGCAATTCTGAAGGTGATGCCCTCCATAATTTGCGGCTTGCTCTTACTGATCCAAGTAATGTGCTTCAGAGCTGGGACCCTACTCTTGTGAACCCATGCACTTGGTTTCATGTCACGTGCAATCCAAGCAATAATGTTATCAGGGTTGATCTGGGGAATGCTCAGTTGTCTGGCCTGTTGGTTCCTGATCTTGGTGAACTTCAATCACTTCAGTATTTGGAGCTCTACAGCAACAATATTTCTGGAGACATTCCCAAAGAGCTTGGAAGTTTGGGTCAGCTAGTAAGTCTTGATTTATATCAGAATAAATTTACGGGATCAATTCCTGACACATTGGGACAGCTGAGTCATCTTCGTTTCCTACGTCTAAACAACAATACTTTAGATGGCAGCATTCCGTATTCCTTGACTAGTGTCAACGCACTTCAAGTACTTGATCTGTCAAACAATAATTTGTCAGGGAAAGTTCCTACCAATGGTTCCTTTTCGCTTTTCACTCCCATCAGTTTTAATGGTAACAGCCAGCTTTGTGGCTCTGTTGTTAGTAAACCTTGTCCAGGAGAGCCTCCGTTCCCTCCCCCACCGCCTTATCAGGCACCTCCGAGCCCTTCTTCAACTGGAAATAATGGAGGAAGGGCATCAACATCCAGTACAGGTGCTATAGCAGGTGGAGTTGCTGCAGGTGCTGCATTGATTTTTGCAGTTCCTGCAATTGGGTTTGCATGGTATAGAAGGCGAAGACCACAAGAGCATTTCTTTGATGTTCCAGCTGAGGAAGATCCGGAGGTGCACTTGGGGCAACTCAAACGTTTCTCCTTACGTGAGCTTCAAGTGGCTACAGATAACTTCAACAACAAAAACATACTGGGAAGAGGTGGGTTTGGCAAAGTATACAAAGGACGTCTTGCAGATGGCACACTAGTGGCAGTAAAACGTCTGAAGGAGGAAAGGAGTCCAGGTGGGGAGCTGCAGTTTCAGACAGAGGTCGAGATGATAAGCATGGCCGTGCATCGCAATCTTCTACGCCTGAGAGGGTTCTGCATGACCCCAACAGAGCGGCTTCTTGTTTATCCCTACATGTCTAATGGAAGTGTCTCCTCATGCCTGCGAGATAGAAGTCCTGGAGATGCACCGTTGGATTGGCCCAGACGAAAGAGTATAGCCTTAGGTTCTGCCAGGGGTCTTTCTTATCTTCATGATCATTGTGATCCCAAAATTATTCACCGAGATGTTAAGGCTGCCAACATTCTGCTGGACGAGGAGTTCGAAGCAGTAGTTGGGGACTTCGGGCTTGCTAAACTTATGGATTACAAAGATACACACGTAACGACAGCAGTGCGGGGCACTATTGGGCATATTGCACCGGAGTATCTGTCAACAGGGAAATCTTCCGAGAAGACTGATGTGTTCGGGTTTGGAATCATGCTGCTGGAGTTAATAACTGGGCAACGAGCTTTTGATCTTGCTCGTCTTGCCAACGACGATGATGTCATGTTACTGGATTGGGTAAGCTGTTAAGAACATGGGTTCATAATCCTATATTCATTTTTGTAGCAGTGTTATTTGAAGGCAAATTTAAGAGAAGGTGGCTAAAGTTTGGAACTGTCTCTGATTTTCAGGAAATTTCTGACTATTTTATTGCATAAAAAGTACGCCAAAAATTGTGTACTCTTGGTGAAAATTAGGAAGAATATGAAACACATGTTTTTTGGATAAAAATATCACTTGGAATATTTTCCAATGGATAGGCTACTTATGTAGTTCAATCCGGGCATGTTCTCCCAGCGTGGCTACAAAATTTTAAAGGGGTCCTCCCAAAATAAACACTCTTTCGAAGTGAGAGAGATGGTTTGATTTTTGATTTTTGATTTTGTTAGGAGTGTTGGACTGATGTGTTTGGATTCTAAATCCTGCAGGTCAAGGGTTTACTTCGAGAGCGCAGGATTGACATGCTTGTTGATCCCGACTTGAAGGCCAACTATGAAACCCATGAAGTAGAGCAGCTAATCCAAGTTGCGTTGCTTTGCACGCAGAGTTCGCCCATGGACAGACCCAAGATGTCAGAAGTGGTTCGGATGCTAGAAGGGGATGGGTTAGCAGAACGTTGGGAAGAGTGGCAAAGAGTTGAGGTGGTCCGTAGCCAAGAGGTTGAGCTTTTACCTCATAGAAGTGATTGGATTGTGGATTCCGTCGACAATTTGCATGCAGTGGAACTTTCTGGTCCAAGATAGACATAACAACCCTTCATTCTCCCAATGCAACTTCCAAATCGGTGGGGCCTCTATATAAAGCTGAGTCTTGGGTTCTTGTGTATATTGAGTTCAGAGTAAGGAAATATATTATCCCAGCCTCTCTCGTCGTAGTTGTGAAATATCACGAGACATCTTCGACAGCATATGCATGCCCTGACAGCTGCAAAAACTTGTAACAACAATTCTTGGCAAGCTTTAGCTTTAGTGAAGGAGAAATTGTTTCCAAAGAAACACCACTATCAAGTGGGGGTTGAATCAATCTACCTTGTGAAGGCACCTGTTTTTGGCTTCTTGTAATTTGTCTATTGCAAAACAATCACTGCTCCACTGACTGTGTGCGTCGTTTATCTTTGGATCTTTAAAGAAGAGAGTTGTATTTTCAATTCGATGTGAGAAAACAAGTTGGCTTTTGTCAATTATGGTGAGTTGGAGTGGTCTGCATTTGCACTGGTTAGACTTATGGGTAAGGATGACGATAGGGAAATGACAATGTATAGACACGGAGAAAG |
| **FERTLIZATION-INDEPENDENT ENDOSPERM (FIE):**  TTTTTTGAGAGGTTCAACCGCATCCACATTGAAACATTTAGACGTGCTCATACAAATACAACAAAAGTTTAGGGGCTTTCTATCATACAAAGTTGCACTTCTTCAATGTTCTTCCATGGAGGACATTGATTTAAGCTTTCTATCCAATTGTAAGAACATGTATATCATTGATTTAAGCTTGAGAGCTATAAAAAACCACAAGAGACTACTGGGCACAAAAAGAATTACTTTGTGCCAAAAAAACTGTATGGAGTGAGAATTTTGATTCCCCACAGCCATAAATACTGCCCTCTTAGTACTGAAATCTTTTAAAAGCGTAATGGAACTTTAGATTAGTCCTAGCACTAGCATTGCTTTGTTTGGATTGGTCAGTCTTGTAAGCTGCAAAAATTCTTCTTGTGAAGCTCGAGTGCTGTGCATACAAAATTTCATCCATCCAAGATCTTTAGAGGAAAACCCAACATGTTCAGTGGTCATCTTTACGGCACAGCATCCCATCTCCAAAAACTACCATCCTCGCACGAGCACAAAATAGTACTGCACCATATGAAAATACAGAACTTAAGAGCACACATGAGATTATACACCTTCCTGTTTTTTTGTCTCCTCATGCTTCCTACTGAAACTTCTTTTCGGGGGAGATACTATGAGAGCTACTAATTTCTTACACTCACACCCTCTTAATATTAAGATGCCAAGGATTGAGCCAACTCCTCCCCATTGAGAAGCACTCACTCTACCAATTCAGCGAATGCTCAGCAGCCTAAGGAAAGTACTTTTAAGAAAAGCTCAGTATTTTATTGCTATAACATGCACAAACCTCCCATCGAAAGACATAGCAGTTTGTCGTATTGGAGATTTGCACTGAGCATAAGATAATTTGGCCAACAATGTGGGGGGACTTGTTTGAAGCTCCCACACAAAAACCTTCCCTTCTCTGTTCCCAATTGCTAGAGAATTGAAGTGGAAATCACATGAGAACTTGATGAACCAGATGTCACAATCAGGGACAGGATACTTTTGTAACACGTCAACGGCACCATCATTGCTTCCACCGTGCTCTTTCATCAAAGCTTCCCATAGAACTATTTCATTGTCCACGCTCTTTGATAGAATGAAATCTCCAAGCCAGCGGGTGCAGTCCACATAGTTCGAATGAACATTTGCTGTGAAAATAGGAAATTGCACATACTTTGTTGAGAACTTGGATGACTCATCTTTGCAATCGAGAGATTGTTCAACATAAACCCAAAAATCTTTTAAACTCCAAATCTTGACAGTGTTGTCCATACCACAACTAGCAATCAGGTCCTTGTTAGACGGGTGGAAGTCCACACTGAGAACTTCATTGCGATGTCCATTTGATCCTGCAAATATGAGAATACACGCACCTGAATTTACATTCCACAATCGCACAGACTCATCCTTGCTTGCAGAAGCTACCAAACAAGGCTTAAGGGCTTGTGTTCTCAACTCGTTGATTGAATCACCATGGCCAACGCAACTTTTGTACAGCTTTTCTTTGCCCACATTTATGACGCGGATGACACCGTTGCTCCCACTTGCAATTAACAAAGGAGAATTTGCATCATCACAAGCCCAACTTACAGTGTAGAAGGCTTCATCTTTATCATCATCAACATAAGTTTGCAACACTGCAATTCCGTTGTTCTCGAGCAAGTGATAAATTGTTACCCGGTTACCGCCTGCAGTGGCGAAGACGTTGTAGAAGCGGGAGTCAATGAAGTTGAAGCAGACAGCGTAAATGGGGCGTTTTCCCTCCTGGAGTCTGTTGGAGACCTTGTATTCCTTCCTCTTTTTAGCGCCAGCCCCCGTGAGACCCACCACAGGGTCGGTCCCCAGCCCGCTCTTCACAGACATGTTCACAACGCACTCCTCACTAGCGACCTCAGATCAAAA |
| **MULTICOPY SUPPRESSOR OF IRA 1 (MSI1):**  AGAGAGAGAGAGAGAGAGTAGTAGTAGTTTATTTTGTAGGTGTGTGTGTGATGTCGAAAGAAGAGGTTGAGTTTCGGGATGAGATGGAGGAACGCCTCATAAACGAAGAATACAGGATATGGAAGAAGAACACGCCATTTTTGTATGATCTGGTGATTACCCATGCGCTAGAATGGCCCTCTCTTACAGTGCAGTGGCTTCCTGATCGCAAGGAGCCGCCTGGCAAAGATTACTCTGTGCAGAAGCTCATTTTGGGTACCCATACCTCAGACAATGAGCCCAACTATCTCATGCTGGCTGAGGTTCACCTCCCTTTGGAAGACTCGGAGAGCGATGCCCGCCAGTATGATGAAGAGCGAGGAGAGGTTGGTGGCTTCGGTTGTGCTAATGGAAAGGTGCAAATAACTCAGCAAATTAACCACGATGGAGAAGTGAATAGGGCTCGGTACATGCCTCAGAATCCATTTATTATTGCAACAAAGACAGTGAGCGCAGAGGTTTATGTTTTTGATTATAGCAAGCACCCTTCCAAGCCACCTCAAGAAGGGCGTTGCAACCCAGATTTGAGACTGCAGGGTCACAAGACAGAGGGGTACGGCTTGACTTGGAGCTCTTTGAAAGAGGGTCATTTGCTTAGTGGGTCAGATGATGCCCGAATATGTTTATGGGATTTGAGTGCAAATCCCAAGGGCCAGAACATGGTTGACGCAAAACAAATTTATGAGTACCACAGTGGCGTTGTGGAAGATGTTGCATGGCACTTGAAGCACGAGCATTTGTTTGGATCAGTCGGAGATGATTGTAGGTTGCTGATTTGGGACACCCGCAAAAGTGGTGATAAGCCCTTAAATTCAGTTGAGGAGGCTCATCTTGCTGAGGTCAACTGTCTAGCCTTCAATCCTTTCAATGAGTGGGTTCTGGCCACAGGATCCGCTGATAAAACTGTTGCTCTTTATGATCTACGAAAGCTGTCCAAACCTTTACATACCTTTAGCAATCACACGGAAGAAGTGTTTCAGATAGGTTGGAGTCCTAACAACGAGACTATTCTTGCATCTTGTGGAGCAGATAGAAGAATGATGATTTGGGACCTTAGCAAAATTGGGGAGGAACAGTCTCCTGAAGATGCAGAAGATGGCCCACCAGAGCTACTCTTCATACATGGTGGTCACACAAGCAAGATTTCTGATTTTAGCTGGAATCCGAAGGATGATTGGGTGATCTCAAGTGTGGCTGAAGACAATATCTTGCAAATTTGGCAGATGGCAGAAAACATTTATCATGATGATGATTTCGGACCCATGGATGATGGGAAAGCTGTTTAATGAAGCAGCTTATTTCTTACAACAAGATTTGTATCACACGTGTGCCTGAGACGAGACTTGCTCATACAGGACATCTTGCCACCTGAGCAAAATGGAACATGGTGGCGTTGTTGCCTATCACCTTCATGTGAACTTGCATCGTTGTAAACGTTGTTTTTCTTTGAGGTGAGCTATGTTTTAGGAGCAAGCTGCCAGGTGTCTTTGGTAGACACCAACCCAGGTTAGGTGCATGAGATTTTAAAGTTTTATACCATGTGTGCTTTTGATTTTTAATCTTGTGACTTCCTTGCCAATGCAGTAGCCTAGAAAAGTCGCATTCTTGTTGCATTCAAGTTGGCAGGATGATGACATTTGGGGGCAACTTTTGTTTGGTTTACTTAATTTTTATTTTGCAAGATGTTTTTCAATACTGAATGGTTGTCTAGACTGTACTGTGTTATTTGCTTGGGCTGCGAAAGACTAGAGAAGGCTTTGGT |
| **BABY BOOM (BBM):**  AGAGAGAGAGAGAGAGAAACTAGATTGTTGAAATTAGTGGGAGTTTTATACAAGAAAATTAAGTATCAGTCCAAGCGGCAAAAAGGGGAGTGGGGCAAACGCCCAAGTGGTTAGCTTTCAAAGGAGAAGAAGCGGAGGCAAAGGGGTTAGGCCAATGAGCTGCATGCAAGTTGATAGCGCTTCTTGCGTGGAGGCTCTCGTGGTAGCTGCTGCCATAGTTGGACCTCACAAGGCTAGGCGTCGTATACGTGCAGGTAGCTGCTGCTTCCGGCCTCGCATGCATCATACCTTCTTCGCTTGTCGCGTGCCCCATACCCATATTATTGCTGTAGCTGCCTGCGTTCACCAGCTGCTCAAATGCGCAGCTCTTGCTCCCCCCTCCTCCTTCAGCACCACCATGGAAGGGTAACTTGGAGCTGAATTCAAAAGCTGAACGATTTTGACTGGAAGATGGGTGCTGATCCAAGCTGCTCAGCAAGTTGTGCATTTGAAGGTCATGTACTTGCACATGGGGATCTTGGTATGTTTTCAGGCTACTTGTAGTATGGCAATCCTTCTGCTCTTCACTTTCTTCCATATTCTTCTGATGCACATTATAGCATGACTCATAGCTTTGATCTGGCTTAGAGCTGCTCCAAGTTGTAGTTGCCTGCTCATCACTAGTACAATTATTGATGCTTTGTGGATACCCTAATAAGAGCTGCCAATCTTGAGCACTTCTTGCTATACTCTCACTTACATGATTATGCACATTAATATTATTAGTGCTAGCATTGATATTATTGGAAAGATTGCTTGTATTGCTATTAAATGAGCTTCCTTCCATTAAGCCAATAATGAGATTATTTTCCCCCGAACCTCCATTAAAATAGTGATGGTGCCCATTATGAAGAGGGTTGAAGAGTGAGGCTTCATTGATTGGGGATATGTCTTTGAGCATGCGCTTGGCGAGTGTGGTGATAGGAAGGCTTGTGCTGCTGCATATCTTGGCGATGTCGTACCTGCTCATGTCAAAATTGGTCACCGCATTTATGCCTCGGAACTTTATGGCCGCTATATCATATGCCTCCGCAGCCTCCTCTTGTGTGCCGAATGTTCCTAGGTACAAATCCTTGTTTCCTGCCACTCTTCCAATTCGAGCCTGCCAGCGTCCATGTTGATGATGTCTTGTGACTCCACGATAGATCGATGCTCCTCTAGAGAAGCCACTACTTTTTCTTCTCAAGGAAGCTACAAACTCTTGCCTTGTCATGTTCTTCATCTCCTCCAATTCTTTTTCATAGTCATCAAGCTGGAAATTGATGGTAGTTGTAGGACCCCAGTACTTCAATGCTGCAAGATCATAGGCTCTTGCTGCTTTCTCCTCTTTGTCATATCCACCTAAGTATACTTGTCTTCCTTTGCGGGTTTGCCCCTCCCGTCTGCAGCTGTTGTCCCAAAGGTGTGCTTCGAACCTTCCGGTCCAACGATGCTTTGTAACCCCGCGGTAAATGGAGGTCCGCTGCCCAAAGGATTCAATGGACTTTCTAGGGGTAGACTCTGCCACTACATTAGCTGGTGCTGGATCTGGGCTGTGCCTCTTGGAGTCCGTAGTGGTGGAAGAACTCTCTTGCACAGCTAATGTTCGATCCGTTGGGTTTGTAGCGTTTACATGCATAATGCTGGGTGTCATTGATAGAGTTAACTGTGATGCTTCAAAAAGAGGCAATATTGGAAAGCTGCCCTCTTTCATGGCCTCCATATTACTTTCCTTTAGCATGCCATTCTCTGTGTTAGATAAACTCTGGGACCTCATCCAAGAGTTCATCAATGAGAAGCCCAGCAAGTTTGTGCTGTTTTGCAAGCCTAATTCACTGGTAAGGCTGGTGTTAACATTGTGTGTATTGCTAGAGGAGGTTACGTTCGCATTTCTAGCTGAAGAGGTGATATGGGTGTTGCTTGTTGTAGTGTAAGGTAGCATTGAGATTAAGTGCTCATTAGTAAAAGATGATGAGTGATTAGAGCTAGGTAACACTGGATCTATGGGATTTTGTAGATGCATTGTGCTTGCAGGTTGGAAGCTATTATCTGCATTGTAGCCTCCAGAAAATATATCGTAGCTTTTGTGAGGTTGTGGATTCGCTGAGGGGCTGCTGAGAGGTTGCTCTAGTGTGGTATTGCTGCAAAACAGTTTGCCTTCAGCTTGTGGGATTAAGGGATTTTCCTCCTTAATGTAGTGGGATAGGAAGTTCTCCAGCTTTGCAGAGCGCGGCTGCGTGTTGTTATTGTCCGCAGTGTTCAAGTGTTGGGCCAGTGATATGCCGGTAGCAGCAGCTGCATCTTCGTGATCATCATCAGCTCTGATGTTGTGAGTGTAGTGGCCTTCAAAGTCCCTCATGTTTGACCAGTCAGATAGCCCGGAGTCGAGATGTAAGTGGGAGGCGGAGGCGGAGGCGAGCGCGGAGTGGACGATGCCACAGAGGGAGTGATGAGGAGGGAGTAGAATGTGCTCCATAAAATTTGGGAATGAATGCGCGCGTCGGCTTTGCTGAAAATATTGTAAAAGCGAGGTGGCGATCACTGGCGCAGACACACACACTGTGAAATACACAGTAGTCACTCAACACAGGGCACACAACACACTGTGACACAAACACACACTGTATTCACTCAACACAGGGCTCAGTAAAATTACACACAGGCCTCTGGACTTCCACACGACAGTCAAGCACAAGATGAAGGAGTGCTGCTTCTGCTGCCCCATTTCTGGTATTTATACTTTATATTTATCCCAATCTATTTTATTACTACTCTCCCTATCTCTCTCATTTAATAGTGTACCATTCTCTATTTTCAAATAATAGTTATAACTTATTGTTATAATTTCTTCACCCGAAACCTTTTTTTATATTTAATTTTCTTCATCGGTTTTAGTCCCCTAATAAAAATTTAGCTCTCCATGTACCGAAACATATGACGAATTTCATTTTCAT |
| **MATERNAL EFFECT EMBRYO ARREST 29 (MEE29):**  TCAAACCCCATCCACCCTACAGTCAAAACTCAATATCGTCGTCATTATTATACTGACGCGCTCTCGATGGTTTAGGGTCTGAACGTCGGCAAGAAGATAGAGATTTTGGGTTTGCAGGGAAAATAATTTTGAGACTACCCATGGCGCAAGAAAAGCAGTTAAGGGTATGGATCTCAGACAATCTTCATGCCCTGTTGGGTTTCTCAGAACCCAACCTGGTGCACTACTTGTCCGGGCTTGGTAAACGCACAACCTCCGCCCAAGAGCTTGCCTCAGAGTTAGTCAAATTGGAGCTTCCCTCTTCTTCTGAAACTGTCAGATTCGCTGAAGAGCTCTTTGGACGCTTTCCGCGCAAATCCACAGGACCCAACAGCTACAAGCAGGCGGAGCGTGAGGCTGCTGCATTTGCCCGCAAGCAAAATCATTATCAGTTGTTAGATGATGATAATGATGATGCCCTTGACAACGTGCAGGAAAACCAGGTAAAGAAGAAAGATGATAATGTTGGGTCCAATGGTTCCGTGCCCAGAAAGGGTCCTAAACACATTCGGAAGAGGCCTGCAAATGTTTATGATGATGATGATGTGAGTACTGGCAGATTATCGAAAGAGAGAAGAGTACAGAACAATATTGACGACGATGATGATGATGAAGATGAGGATGATGAGGCTCAAAGAGAGCGTGAGAGGGAAGAAGACCAAAGAGAAAGAGAAGCTCTTGAGAAAAGGCTTAGAGAAAAGGATGAAGCCGTCACAAAGAGACTTATGGAGCCTAAAATGACCTTCAGAGAGGAGGAAGAGGCAAAGCGACGTGCTGAAGCTGAGGACAGAAAGGAGCTGCTACCCACTTTAAGAGAAGCTAGTAGACAAGTGTACTTGAAGTCAAGGGAGCAGAAAAAGATTGATGAACTTCAGGATCAAATCGAAGATGAGCAATACCTGTTTGAGGGAGTGCAGCTTACAGCAAAGGAGAAGGAGGAACATAGGTATCGAAAAACTGTGCTTGAGCTGGCCAAACAAGGAAATGAGGACATTGATGGCATCGAAGAGTATAGGATGCCTGAAGCATATGACAAGGAAGGCAGAGTCAAGCAGGATAGGAGATTTGCTGTGGCTCTGGAGCGGTACAAAGATGTGGATGGTGAAAATGATAAGCTCAATCCTTTTGCAGAGCAAGAAGCTTGGGAGGAACATCAGATTGGTAAAGCAAGGCTCAAGTTTGGTGCAGCAAATCAAAAGCAGAAAAGTGGTTATGATTATGTTTTTGAGGATCAAATTGAGTTCATCAAAGCCGATCTGCTTGAGGGGTATAAAGACCAGGATATGGATATTGAAGAAGCTGAGGAACAAGAGAAATCAGCAGCAAAGTCAGCTCTGTTGCACCTGCAGGAGGAGCGCAAAACTTTACCAATTTTTCGTTATCGAGAGGAGCTCTTGCAAGCTGTGAATGATCATCAGGTTCTTGTCATTGTTGGAGAAACTGGTTCAGGAAAAACAACCCAAATCCCACAGTACCTTCATGAGGCAGGGTATACAAAGAAAGGAAGGATTGGTTGTACACAGCCTAGGAGAGTTGCTGCCATGAGTGTAGCTGCACGCGTCGCACAAGAAGTGGGGGTGAAGCTTGGCCATGAGGTAGGATACTCAATTCGGTTTGAAGATTGCACCTCAGACAAAACAATAATGAAGTATATGACAGATGGTATGCTTCTTCGTGAGTTCTTGGGTGAGCCAGATTTGGCAAGCTATAGTGTTATGATGGTAGATGAGGCTCATGAGCGAACTATTGCCACAGATGTTTTGTTTGGCCTAGTGAAGGATATTATCCGTTTCAGGCCAGACATTAAGCTTCTCATTTCAAGTGCCACATTGGATGCCAAGAAGTTTTCTGATTATTTTGACCGTGCTCCTATTTTTAGCATCCCTGGAAGGAGGTATCCAGTTGACATTATGTATACAAAAGCTCCAGAGGCAGACTATCTAGAGGCAGCTGTTGTAACTATCTTGCAGATTCATGTGACACAACCATCAGGAGATGTGTTAGTTTTCTTTACGGGGCAAGAGGAGATAGAGGCTGCAGAAGAGATACTAAAGGCACGCACCAGAGGCCTGGGTACCCGAATTTCTGAGATGATTATTTGCCCAATTTATGCAAACCTGCCATCTGACTTACAGGCCAAGATTTTTGAGGAGACCCCTGAAGGAGCCCGGAAAGTAGTTCTGGCAACCAACATTGCAGAAACATCTCTCACAATTGATGGCATAAAATATGTGATAGATCCTGGTTTTTGTAAGCAAAAATCTTACAACCCACGTACAGGCATGGAGTCGCTTATAGTGACACCCATTTCAAAAGCAGCAGCTCAACAGAGGGCTGGCAGGGCTGGGAGGACGTCGCCTGGAAAGTGTTTTAGGTTGTATACCGCATGGTCTCATCAGCATGAGATGGAGGATAATACTGTACCAGAGATCCAGAGGACAAATTTAGGGAATGTTGTTTTAATGCTGAAAAGTTTAGGCATTGACGACCTCATAAATTTTGACTTTCTGGATCCACCGCCTGCAGAAACCTTGCTTAGGGCTCTAGAACAACTGTATGCACTGGGAGCTCTAAATCACCGTGGAGAGCTGACAAAACTGGGACGCAGAATGGCAGAATTCCCTCTTGATCCGATGCTTTCAAAGATGATTGTGGCGTCTGATAAGCATAAGTGCTCAGAGGAGATAGTGACGATAGCTGCAATGCTGTCTGTCGGAAATTCTATCTTTTATCGGCCCAAGGACAAACAGGTTCATGCTGACAATGCAAGAATGAACTTCCATGCAGGCAACGTGGGCGATCAGATAGCCTTGCAGAAGGTTTATGATACCTGGAAGGAGACAAACTTTTCAACACAGTGGTGCTATGAGAACTACATACAGGTACGGAGTATGAAGCGTGCTCGTGACATCAGAGATCAGCTTGAAGGGCTCCTTGAACGAGTTGAGATTGAGCCTAGCAGCAGCCCAAATGACATAGAAGTAATAAAGAAGGCCATAACATCAGGCTATTTCTACCACACAGCAAAATTGCAAAAGAATGGTTCGTATCGAACAGTGAAGAACCCTCAAACTGTGCATATTCATCCAAGCTCAGGCCTTGCTGAGGTTTTACCTCGCTGGCTGGTATATTTTGAGCTGGTGAATACAACCAAGGAGTATATGCGACAGGTTATTGAGATAAAGCCAGAGTGGTTGGTGGAGATAGCTCCCCATTATTACAAAAAGCAAGATGTTGAAGATGCTGGCTCGCAGAAGCTGCCCAAAGGCATGGGAAGGGCCAACATGGATTGATCACTGTGACGACAAGTATATGCATGAGAGGAGAGCTTCAGATTATGGAGGTAAATGATGTCCAGAACTAGCAGTGTTGGTTCTCTCTTTTTGTGATGCCTGATGAATATCAAATCAAGCAGAGCGCTTATAGATTTTCCAAGATTAAAATCAACATGGATATTTTGGAGTGTGTATGGAAGTATTTGGGACCCTTGGGCCTTGCTGCTGCCTGTGTCGACATGTTGCAAGCTTTGTTTGAACTTTGATCATAATGGTGGTTCTTGGAGGCTGGAGCGGGTTTTCAATAGCATGGGGATTGCAGAGATTTATTCTATGCTCTCAACAAGGTCTAATGGATGCACCACCTCTTGCAGAAATTGCATTTTGACAGTTTTCACTAGTGAGACAGCACCAAATACAGATGATTTCTGATCTACTTCTGTATTTGCTGCTTTGAAGCAGGTGGGGTGTGGAAAGTACGTCTTTCCTTGCTCTTGCAGCTCATTCAGGTAAGCAAGCAGTAATGAGACGGGCGCATGGATATAGATTTGTCTGAGCGATGTTGGCTGTCCACTAATCCAACTCTACAGTTGTTGTATGGGTCTGCGTCATGTCATCATGCATAGACATTCAGGTTCCTTAAACCAAAGGTCATGAAACACAAAGCTTTGAGCATGGACAGCAGAGATCATAGATACAAAGATCTGTGTACTTTTTCTTCACAAGCTTTTGCTCTTTACTTTTGTGCAATGTTATTGAGGTATTTAAGCTCCAACAATGTATGCCCCGACCAGTGTAAATTATTGCCATGTTTGCTATTTGTTTTTTGATGGTAGCCTCACAAAAATTTCAGTCCATCAGGAACCAATCTTTGAAAGCTGTTGGCAATGAACGGTATGAGAGTAGATTTCTATGTACCAGGCTTGTGG |
| **AGAMOUS-LIKE 62 (AGL62):**  GTGTGTTTACGTGTGCGCGCGCGAGAGTGGGCTGCTTAGACGTGGCAAAACTTGCCAAATTAAACCCTTTACAAACGCCCTTTTACATCCTGAATTTCTGCGTCGCTTGGTAGCTACAATGTGCATTGCTTCTCTTGATCGCCCAGACAAACTTAAAATGCTGATTAGCAATGTTAATTATCCTGTTTAATCATTGCTAATTACTATCAGAAATTGCGTATATCAACAATACTTAATTAGTTTCTAATTAATCTCTATTAGTCTTAATCATATATACATATAATGGGCTCTTTAGATGCCTGTGGATTTCTTTATTTTTCATTCCAGAGATTTACTGTTTTGTACTATCTATCTCTATAAATCTTAATAACTTCTGATAGAAATTGCTCCAAAAAAAAGAAAGTACTACGAAAAAATGGTTAGGTCTTTGCCCTAATTTCTGCTGAGGAGCTGAATGATACTATCTGAAACAGTGATCAAGGTATTTAATTGAGAAATCAAATGAGATTTTCTGGAATTCAGATGATTTGCAACAATGGGTCGTGCGAAAATAGAGATCAAGAAAATCGAAAACTCAAGCGCGCGTCAGGTGTGCTTCTCAAAGCGGAGAGTAGGACTCATCAAGAAGGCAAGCGAGCTCTCAATTCTATGTGGGTCAGAGGTGGGCATCATTGTGTTCTCTCAAGCTGGCAAGGCCTTCTCATTCGGGCACCCATGCATCGACTACGTGATCGACAAGACGCTCAAGAGACCTGTTTCCGTAGACTCCGACAAAATCGAGGTCATACGGAGGCTCGAGAGCGAGTACAACACACTCCTGCAAGAGCTTGAAGTCGAAAAAGAGAGACATATCGCCTTGCAAAAGCAGCTTCATGTGGATTACTACAACCATAAGCATTGCTGGAACCAAAATTGGTGGCAAGAGCCCATTCATGCCATGGGTTTGCTAGAGTTGAAGCAGCATGCGGAGCGGTTAGAGGCATTCTACAGGCTTATTGTCGAGCGGGCTCGATACTTGCAGTACGTGTCTAGCTTGGATCTGCCCTTAATGCAGCAGTATCAAGTGATGCTTAACAGACAACAAGCATCAGCGGGTGCTTTCATGCTTAAGCAACAGTTCTCGCCGGACCATCTTCATCACCATCAGCATGCCCCTCAGCAACTTCCCCAGTATATGGACATGAATAGTATGCCATACAAAACTGTGCGCTCTTCACGGGCTCTTCACAAGGAGTTTACACAAGAAGTGGATACAAGGGATGATACCCAAGGAGAGAGAACTATAATCTGCTATGAACACAGATGACGATCAGTATTCTTGAAAGGGGTGCTTCGCTCTGGTGCATGTCATAAAGCATTTTTCCACACGGCATGCGCCCATACATGTCAAGCCGGAGATCAGATGCACAGTTGAATAACATTTGTTTACTGACCACTTGTGACATGCATGCATGTCTCCTGATTGATGTTATATGTAGATTTAACATAAGTATGCATGCAATAGAAAATGGCAAATTTTGGTCTGCTCCTGTACGATGCCTACTGGCGAGCTGTTGGGGATGGTTGACCAGTACTGACTTGGCCACCAGTTACGCCTGACGGACACGTAATGAAGAAAGCCGATAGAGTTTTAGGTACTTCAATTATTAAGGTACATTTTTTAAAGAGAGACAAGATATATTATTATGACAGTGTTTAAGGTCTAATGATATGGTATGAAAGTACAGCAGCCTCCAAAATAAGATCCTGAAACAATCCTGCATGCCTTCATTCTACGCACTATTTATGATGTATGTATACCGAATACGTCTAGCAGCCTCTGAACTGAGTACCTTAAACAATTAAACAAACTTTAGTTTAGAAAATTAGGCATAATTAGTTAAACAAAAACTTAAATTGCCAAATGATTTTGCAGGCATGCATGTGTTCTACATGATATCCGTCAATATGATGTTTCTTATGTCCTCAACCACATGTACGTGTGTGCATATTACGTGTCTGTTGATAAGATGTGTCTTTGTGTACTGTTCTACATGTATGTGCATGCACCTTGAGATAAAAATGTGTCTTATGTATGTACTACCACATGTATGTGCGTACCACAGAGCTGAATGTTGTTAATTGTTAACTTTTTGGCAATGAAGGTAGCAATGGAGAGAGACATGGGTTCGATTGCTGGAACTTCAAATCTGGAGCGCTGTTTTTCAGTACAAGGTTTGGCGAATTCGATACCAAAGGAAGAACCCAATGCTTCTTTCAGCAATATGCTGCTTCTAACAGCAGCAGATGAGGATCAAATACACATGAGTCAAGAGCGTGTGAGGTGGGCAAGCCTAGAGCAGTCGTGTGTGCTCGAGCAACCAGAGCAACTGCCAGATGAACAAGCGTTGATGGGAGAGGAAGGGCAACAGGAACCGCCTGATGAGAGCGAATTGGAGGCTCCCATGCCAATGGATCAAGCATTATATCTAGCAGATGATTATACCACCGATAACGAAAATTTTGACACCGACCTCTCAGACAATAATGAATACATGGATAATATGCTTGGAAGCTTGGCAGGTTCCCTCTCTGCTGATCATCATGAGGATCACAGTAGTGTGCACGCTTCAGTTGACCTCTCTGCTGATGAAGAACATCAAGGGGACAATGTAATTAACACCGCTAATGACGCACATCGTAGTGATATGGTCGTCAACTTCTCTGCTAATGTGGATCATGAGGGTGATATGAATATCAGCCTGTCAGCTTATGATCTTCATGGTAGTATGGCTACAAATGTTGGTGGTAATTCTGAAAGTGGTTATAACATCAATGTACCAATTGAAGATATGGATGGTAAGTGTCCTGATATGGCTCGCATTCGCACAAATATGGACAAAGTGTCTTTTGATGATCCAGATAACCATGTCAGCCTGTCTTCGGAGGATCACGGAGTGCTTGTAGCAGGTCTCTCTGCTGATGATGATGCTAATGCTGCTTGCCTCTCCGCTAATGATGACGATGACGGCAGTAAACTAGATAGCCTTGATGCCACTGGTACTAGCTTTGGGAAGTTCTATGCTGATGGTGGTGAAACTTCTATTATGGCCAACCTTTCGGTCATTAACGAAGTGGAGAATGTTGCATCCAGCCCTCGTGCAGCTCAAGAGAACATGTGTGATGGAAATTCTGGAGCCCTTTGGCCCTCTTGCGATGCTACTTATCTTACCGAGAATTCGTCCATACAAGAGGATTCAGATCAGCTTATAGTTAGCATTTCAAATCAGGATTAGCCACTGCTACCAATCGTTAATGGAGTTGATCATCGCAATATTGTGAGGAACAATGTGCAAGGTAACAGTAATTAGCCATCTAGCAGCGCGATGTTTCAGCAGGAATGTCTTCATATCACTAAGTCGAAAAGATGGTCTTAGACCAGTGTTCACAGTTTCGGCTAGAAACTCGAGAAAATCACATAAAATTCTACAAATACACGACGTATGTACAGACCTCTGCCCACTTAACGTTGCTCAAGTGTAGCACACAGAAGGGCTACCATAGGGTCCTGCACATAGTGTGCATCGACTTAACAGTCATCATAATCAGTCATCGTCATCATGATTTAAGGAGTCCTATGCTTTACGGAGGCAGCACAAGCTGGTATTATTATGCTAAATATGTATATACATATATATATATATATA |
| **AGAMOUS-LIKE 6 (AGL6):**  GACGTTCTCAAAGCGGCGGGGAGGCCTGTTGAAGAAAGCCCATGATCTCTCTGTCCTCTGCGACGCTGAGGTGGCAGTCATTATCTTCTCGAGTAAAGGAAAACTCTTCGAGTTCGCCAGTCCCAACATGGCAAGCATATTGGACCGCTATTTGAAGTGTAGTGAAGATGTAGGATGTGCCGATAACAACAATCTTTCGGATAAAAGAGCATTGACCCACTGCACAGAAGATCTTAAAACTTTGCAAAGAAACTTTATTGGTGAGGATTTAGAGCGGCTATCTTTACAAGATCTTATCAATTTGGAGCATCAAATTCACGACACTTTAGGTCGAGTTCGCACAAAAAAGGGGGAGCTACTCATTGAACAGCTCGAAGATATCAAGGAAAAGGTAAGGACAACAAGTGCGAGTTCGAGCTTCTTGGCTAAACTAGTGGACTCATTCCCTTTGGATGCGGCGGGATCACAGTATTCAATGAATAACAGCATTTGCACGATGCAGAACATTCCAGA |
| **ARGONAUTE 4 (AGO4):**  TTTTTTTGGAACAAAACACTTTTCTTTCATTTGTAGTGCATCAAGCTAACACTGCTTTTACATCAAAAAGTTAGAAGGCCTCTCCTTTTCTGGAGTGGCAACCATTGTCTATACAGCAGCTCACAAAAGGCAAAACACATTCTATGGTGACCTTCATCAGGAGTACAAAAATATTGCCTCTGTAATTACAGCTGGTGCTTAAGACCCTAGATGTACTGTAGAAAACAGCTCAGTACATGTTCGAGCATGAAGACCCTAGCAGTAGAACATTTTAGTCTTCATTTGCGGATGCAGCTCCGGTAAATGGATAGGAGTTGACCGCTCAGATCTCCCGGTCATTGACGAAGTTTCTGAGCCACCCTCAGAGTCCAAGAAATTCCGGGCGTGCGTGGCCGCAAGGTGAGCATATGCAATCGGAGCAACGGTTGAAACTGCTGTGGTGCTTCGAGCATATGTGTAACACAATGCATGAGTCATCTGTTGCAACTCATCCACTGTGAAGTTGTTCTCGTCAACCAAAACATGGTAATGAGTGGGGCGAGTTGTACCAATCAGGCCTGCGTGTGCGCACAGGTAGAAGTCATAGTCCCTTGGATGGCAGAGGGTTGCATCCACAATGGTACCTGGAGCTACATTTCCACCACCGGTTGCTGGAAAGAACCGAGTATGGTGCCTCTTCTGTACCACAATCAAAGTTATTCTCGGATCATAGCTGCCAGTGTTGTCTATTTTTTTGAAAGCCTACACAATTAAAACAGAAATCATTATATAATAACTATACTAAACAACCGCATGTGTTTAAAACATCAAAATCAACAGGCTGACTGTATGTAACAGCCATATATAGCCATGTGACTTCATTGTAAAGGATTGTAAGCATTTCTTGCCCTCTTGAATGCCAGATATTCATCTCTTAAAACTTGCTCAAACTGGCTTTCACTAACACCATCCCTAAAAATTATCACTTGCTTAGGTCGAGGGTCAGTGATTCCAGGAACTTTCTTGCAAGAAATGTAAAAATCCTTCAACAACTCAACAATAATTCCACCACTGGGGTTGTCTTCTTCATAGAGCCCTGCTATCATTTCAACTTTCCGAGACTGTGCTCTCATACGAACACCGTACTTGGAAAAGTACGGCCATTCTCGTGATGCCACCACTGCAGCTATTGAAGGAGAGTCTGAATATCCAGGAGGCCCATGAGAGACGTCCATCCCAAATATGATTGTAGGAACACTGGATATCTTGGGAAGTTTAGAGTTCAATTCCAATGCCAGCAAAGAATTATACCCTCCCACCTTTGCATTAATCTTCAAAGCAACATTGGTCAAATATTGGTCCTTCACTAGGTTATAAGGAGGAGCTGGGGAGGAGCTATGCATTGTGTGACAATGCACAGATTAGTTTGCAACATCTTTTTTAGAGGCCCATATAAATCACTCTTGCTTTCCGGAAGAATACACAACATAAAAGCAGGCGGTGATCGCATTTTTGATTTCAAATCCTGTATTATTCGGTTAACCCTTTCTGCAGCAGATAAGCCTTGATCACTGAGTTGCTCGCATAGTATGAGTTCGGGGCGTGAGATTTTCATTCCCTTGGTGCTGCAGACTATCTCCAGATCATTTGCAATCTTTTCCACAATATAGCGAGGCTTTTTGTTATCAAAACTTATGACTACCCAGGATTCAATGTCGACAGCAGATGCCATTGTTTTATTGTTGAAGTTCCACCTCCCATTCCGTGGTGTCTCTGTTCTAGACTCCCCAAACATCAAAGTGGGTGCGTCTAGTAGCCTTGCAGGGATCCTCCGCATTCTAGAATCAATTTTTATGTTAAAATCCTTTATGAGCTCATTTGAAGAATAATCACTAACATCCATAGCCTTTTGCACAACATCCCTTCTTTCATCTGGTCCCTGGCGAGCTTGATCAATCATTTTCTGTCTCTGGGCACTTGACAGGGCTTTGGTGTATCTTTGTCCCGCCACAATGTTGCAAAGCTCTACTGGGATAAATGTGGGCCTCTTGGTTCTACCCGCTGCTATGCATGGCAAATTTGGAAACCGAAGATTTATGTTGTATTGCATTTCGTAATATTGTAGTATCGTCAACTCAGTCTCTACGAATTGCCCAGATTCATCCTTCTTCCTTTTCGTGAACTTCTGCTGCGAGCAAGAAGCATCACTGAAGCCTATAATTCTATGCACCATCTGGGTGTGGGGAGTCTCAACCTTGACCCCTTTCAAAACTCTCTTCACCTTGTCCCAATCAATGCAATTCGGACTAGGGGGATTAAAGCGTTCCTTAATGAAATCCAAGACGCTAGAAGCCTTGATGATAGTTGTTGTAGCAACGTCAAGGTTTAGTGACAGCCCCCTAAGAGAAGGCCGAAAGCTAACGTGATACCCTCGACAGGTTTGAACTCCTTCCCCAATATCAGCAACTCCAAACAATGGGCTGAAGTAGTTGTCCTTGATTAGGAGATAGCCCTTGGAGGATGCATGCTGCTTCAGAACAATATCCAGCACCCGCAGAGCATCTTGGGCCCTGTCATTCTTTTGTCCATTCAAAATTGCATTGATGGCTCCCATCTTCACAGTAGCTGCATACTCAATTTTAACCTTAAATTCTCTAGTGTTCCTGGATGACATTTTGCGCCGCTTTGTTGAATCATCATCTGCTGAGTTTGCTTTGTTCATCGCAGACTCCTCCAACAGGACTGTAAACTCCTGGGTTTGGAACTCGAGGGCACCTGGCGTGAATAGACTCTTCTCTCCATCATAGGCAAACCGCTTGCCTCCTAGTTCTTTTGCTCCGTATACTTGTTGAAGCTTGCTGATAATGGACCGACAAAGGTTTTTATTTGCGATACCATCGTTTCCACCAGGCGCTGAGCCCCCATCTGAGATACTCACATCGTAGTGGCAAACGTCCTGCGATCCGGAGTAGTTGACCATAAAGTGATTGCAGAGCAGCTCAATGGGCCTGCCCGCTTTTCCTAATCCGGGTCTTTGCATTTGTGAAGGCGGCTTCATTGCAGGTACGTCCGCCACATCTGTTGGGTTCGTTGATGGCTCTCCTGTGAGTGCCGCATCTCCTGCTGCTTCTGCCACCGCTGCGGGGTCCCCCATGGCTCCAAGCTTGAGAATGGCACTTGTGCAGAAGAACTGAAGAACTAAGGGCTGCGCAGTGCGAATTAAGAGAAAGGGCACAACTGAGAAAGGGAAGGTAAAAAGGGGGGGAAAAGAGAGAGATAGAGAGAGAGAGAGGGAGAGAGA |
| **ARGONAUTE 9 (AGO9):**  TTTTTTTTGGAACAAAACACTTTTCTTTCATTTGTAGTGCATCAAGCTAACACTGCTTTTACATCAAAAAGTTAGAAGGCCTCTCTCTCCTTTTCTGGAGTGGCAACCATTGTCTATACAGCAGCTCACAAAAGGCAAAACACATTCTATGGTGACCTTCATCAGGAGTACAAAAATATTGCCTCTGTAATTACAGCTGGTGCTTAAGACCCTAGATGTACTGTAGAAAACAGCTCAGTACATGTTCGAGCATGAAGACCCTAGCAGTAGAACATTTTAGTCTTCATTTGCGGATGCAGCTCCGGTAAATGGATAGGAGTTGACCGCTCAGATCTCCCGGTCATTGACGAAGTTTCTGAGCCACCCTCAGAGTCCAAGAAATTCCGGGCGTGCGTGGCCGCAAGGTGAGCATATGCAATCGGAGCAACGGTTGAAACTGCTGTGGTGCTTCGAGCATATGTGTAACACAATGCATGAGTCATCTGTTGCAACTCATCCACTGTGAAGTTGTTCTCGTCAACCAAAACATGGTAATGAGTGGGGCGAGTTGTACCAATCAGGCCTGCGTGTGCGCACAGGTAGAAGTCATAGTCCCTTGGATGGCAGAGGGTTGCATCCACAATGGTACCTGGAGCTACATTTCCACCACCGGTTGCTGGAAAGAACCGAGTATGGTGCCTCTTCTGTACCACAATCAAAGTTATTCTCGGATCATAGCTGCCAGTGTTGTCTATTTTTTTGAAAGCCCTCTTGAATGCCAGATATTCATCTCTTAAAACTTGCTCAAACTGGCTTTCACTAACACCATCCCTAAAAATTATCACTTGCTTAGGTCGAGGGTCAGTGATTCCAGGAACTTTCTTGCAAGAAATGTAAAAATCCTTCAACAACTCAACAATAATTCCACCACTGGGGTTGTCTTCTTCATAGAGCCCTGCTATCATTTCAACTTTCCGAGACTGTGCTCTCATACGAACACCGTACTTGGAAAAGTACGGCCATTCTCGTGATGCCACCACTGCAGCTATTGAAGGAGAGTCTGAATATCCAGGAGGCCCATGAGAGACGTCCATCCCAAATATGATTGTAGGAACACTGGATATCTTGGGAAGTTTAGAGTTCAATTCCAATGCCAGCAAAGAATTATACCCTCCCACCTTTGCATTAATCTTCAAAGCAACATTGGTCAAATATTGGTCCTTCACTAGGTTATAAGGAGGAGCTGGGGAGGAGCTATGCATTGTGTGACAATGCACAGATTAGTTTGCAACATCTTTTTTAGAGGCCCATATAAATCACTCTTGCTTTCCGGAAGAATACACAACATAAAAGCAGGCGGTGATCGCATTTTTGATTTCAAATCCTGTATTATTCGGTTAACCCTTTCTGCAGCAGATAAGCCTTGATCACTGAGTTGCTCGCATAGTATGAGTTCGGGGCGTGAGATTTTCATTCCCTTGGTGCTGCAGACTATCTCCAGATCATTTGCAATCTTTTCCACAATATAGCGAGGCTTTTTGTTATCAAAACTTATGACTACCCAGGATTCAATGTCGACAGCAGATGCCATTGTTTTATTGTTGAAGTTCCACCTCCCATTCCGTGGTGTCTCTGTTCTAGACTCCCCAAACATCAAAGTGGGTGCGTCTAGTAGCCTTGCAGGGATCCTCCGCATTCTAGAATCAATTTTTATGTTAAAATCCTTTATGAGCTCATTTGAAGAATAATCACTAACATCCATAGCCTGGCATAAATCTTTTGCACAACATCCCTTCTTTTCTCTGGGGCTTGGCGTCCTTGGTCAATCATTTTCCGTCTCTGGCCACTTGATAATGCTTTTGTGTATCTCTGCCCTCCTTTTATATTGCAAAGCTCTACTGGAATAAAAGTGGGCTTCCTGGGTCTTCCAGCTGCTATGCATGGCCAGTTTGGATACTTAAGAGTTATATTGTACTGCATTTTATAGTACTGCTGTATCGTCAATTCCGTCTCTACCAATTGCCCGGATTCGTCCTTTTGCTTTTTTATGAACTTCTGCTGGGAGCAAGGAACATCACTAAAGCCGATAATTCTATGCACCATCTGCGTATGTGGAGTCTCAACCTTAACTCCTTTCAAAACTCTCTTCACCTTGTCCCAATCAATGCAATTAGGATTACGGGGCTGAAAACGTTCCCTAATAAAATCTTTGACGCTGGAATACTTGATGACTGTCGTTGTAGCAATGTCAAGGTTTAGTGACAACCCCTGAAGAGAAGGCCGGAAACTAACATGATACCCTTGACATGTCTGAACTCCTTCCCCAATATCAGCAACTCCAAACAATGGGCTGAAGTAGTTGTCCTTGATTAGGAGATAGCCCTTGGAGGATGCATGCTGCTTCAGAACAATATCCAGCACCCGCAGAGCATCTTGGGCCCTGTCATTCTTTTGTCCATTCAAAATTGCATTGATGGCTCCCATCTTCACAGTAGCTGCATACTCAATTTTAACCTTAAATTCTCTAGTGTTCCTGGATGACATTTTGCGCCGCTTTGTTGAATCATCATCTGCTGAGTTTGCTTTGTTCATCGCAGACTCCTCCAACAGGACTGTAAACTCCTGGGTTTGGAACTCGAGGGCACCTGGCGTGAATAGACTCTTCTCTCCATCATAGGCAAACCGCTTGCCTCCTAGTTCTTTTGCTCCGTATACTTGTTGAAGCTTGCTGATAATGGACCGACAAAGGTTTTTATTTGCGATACCATCGTTTCCACCAGGCGCTGAGCCCCCATCTGAGATACTCACATCGTAGTGGCAAACGTCCTGCGATCCGGAGTAGTTGACCATAAAGTGATTGCAGAGCAGCTCAATGGGCCTGCCCGCTTTTCCTAATCCGGGTCTTTGCATTTGTGAAGGCGGCTTCATTGCAGGTACGTCCGCCACATCTGTTGGGTTCGTTGATGGCTCTCCTGTGAGTGCCGCATCTCCTGCTGCTTCTGCCACCGCTGCGGGGTCCCCCATGGCTCCAAGCTTGAGAATGGCACTTGTGCAGAAGAACTGAAGAACTAAGGGCTGCGCAGTGCGAATTAAGAGAAAGGGCACAACTGAGAAAGGGAGGGTGAAAAGAGAGAAGGTAAAAGAGGGGGGGAAAAGAGCGAGAGAGAGAGAGAACAAGAAGCTGCTTAGAGAGAGAGAGAGAGAG |
| **ARGONAUTE 10 (AGO10):**  CATAATTTTTGGAGCTGATGTCACACATCCACATCCAGGGGAGTTCATCAATTGCTTCTGTGAGTTTTGTAGGAATGTGAGTTACTTTATAGAGTTTTATGATTAGTATATGCATATATTGGTGAACTAGCTTATGGCTTTCTCTAACTCAATCAAATGTCTCTTTGCTACAAGGTTGTTGCATCACAGGATTGGCCTAAAGTGACCAAATATCCGGAATTGTTCTGTGCACAAGAACATCGACAAGAGCTCATCCAAGATTTGTTCAGAAGCTGGGTAGATCCTGTGAGAGGGCCTCAGACGGGTGGTATGAGCAAGTTTATATCCTGCTTTTGGTTCTAATTAATGATCTTTTAAGAATGATATTGGGTGTCATTAAGTGTCATTTATTTGTAGGATGTAATTGTCAGGCAGCTTTTAATCTCTTTTCGACGAGCAACAGGTCAAAAGCCTTTGGGAATTATATTCTACAGGTAAGGCATGAGGCTGTAGTTAATTAAATATGGCTTTTCTTAATTAAGACGGCAGCGCTGGACATATTTGTGGGAGGGATGGTGCAAGTGAGAGCCAGTTCTCTCAAGTGCTGCTTTATGAAGTGGATGCTATTCGCAAGGCTTGTGCATCTCTGGAACCGGACTATCAGCCTCCTATCACATTTGTAGTTGTCCAGAAGC |
| **SERRATE (SE):**  GTTTAGGGTTGTCTAATGGTCCCCGTTTCTGCTTCCCGTACCTTTGGGCTTACACACACACACGATATGATCGTGTGCTTCAATGGTTTTCACCTGAGACAGGTTGCAGTTTAGATAGGGCTCTTACGCAGCTGCATATTCATCAGCGTCTTGGTGGATTAGGGTTAGGGTTTCGCTGGCTTGACAGGAAATTGCCCAACAGAATTTCTCCATTTGGGTCGTCAGAATACCTAGGGTTTGAGTTTGTCTGTCCATTTTGCCGTGCTTTTAGCTCTTGGTATGGCAGATGTATTGGATATTCCCCCGGAGCCTGCCCTTTTGCGCGACGATAAGCGCAGAGACAGGGTACCTGACCGCAAAGAAAGATCCGTTGATGATCGCAGACGCGATAGGGATCCCAGAGACCGGCGGGATGAGGGGAATGAACGCGATGAGCGGCTGTCACGGCGTGATCATTTTGACAGGCGAAGTGACGATGAAAGACCGGGGAGTGCCATGACTCGAGATAGGGACCGGGATTACAAGAGGCGCAGAACTCCGAGTCCTTCACCCCCTCCTCTCTATGGCCATAGAGATCGAGAGCGCAGGATTTCTCCATTACGCCGCTCCCCTTCTTCTTACAAGCGGTCCAGGAGGGAGGATGACTATGATGGAGGCCGGCGAGGTTCTCCTAGAATGGGCATGGATGACAGACGGGATAGAAGGATGGGAGGTGGTGGTGGTCGAAGTAATAGCTATAGCGGTGATGAAAGAAGCTATGGCAGACATCATGGGTTTCGTCCTGAGTTTGGTAGGGGTGGCTTTGCTGATGGACCTTTCTCCTATGATGTTGGTCCTCGTAGAGAGGGACTTATGACGTACAAGCAGTTCATCACGGAGTTAGAGGATGATATCATTCCCACAGAGGCTGAACGGAGGTACACCGAGTACAGAAACGAATTTATCTCGACACAAAAGAAGGCTTATTTTGAGCAGAACAAGCAGGAGGATTGGTTGCGGGATAAATACGATCCATCACGTCTGGAAGCAGTCATTCAGAGGAGAAATGAAGCATGCAAAACTGCCGCTAAGGAGTTCATTTTGGAGTTGGAATCTGGCTCTCTGGACATAGGCCCTAATGTTGTAGGACAGAATCCACAGGGTGCGCAAGAAGTTTCCGAGGAAGAGACGGATGACAGGAGGAGGAATGGGCGTGGTTCTGCCAAAGAGCAGGAGTTTGATGCTCCCAAAGCTCCTGCCATCTTTTGCGAACCAAGAAGGATAGAGAAAGATGTTGAACAGGCACGTGCTCTTGTCAGGAAACTTGATGGTGAAAAGGGCATTGAAAGGAATATACTTTCAACCTCAGAAATGGATAAATCTGATGGGGAGAGAAGTGGTGGTAAGATGAATATTGTCGTTGTGAGAGGTGCTAATCATGTTCAGGGCTATGAAGGTGTGGAGCTCTTAGATGTGGTGATAACCTATCTATGGCGTGTTCATTATGTTGATTATTATGGTTTCAAAGAGTACAAAGAACAGCCCAAGGTAATGCGGCATGTTAGAGGAGACGGCAAAGTGAATGAAGATATGGCCTCATCTGTTGAATGGGAGAAGAAGGTTGATGGCACTTGGCAAGGAAGAATACAGGGACAGGATCTTTTAGAGCTCATGTTGGGTAAGGAAAGAATGGAGTCTGCCACATTGCTAGCTTTGGATCCCCTCATTCGAAAGATAAAGGATGAGAAATATGGGTGGAAGTATGGTTGTGGAGCAAAGAATTGCACTAAGCTTTTCCATGGGCCTGAATTTGTTCAAAAGCACCTGAAGTTGAAGCACCCTGAGCTTATTCAAGATGTGGCAGTGAAAGTTTATGAAGAGCTATATTTTGAGAACTATATGAGTGATGCTGATGCTCCTGGATCCACTCCAGTTATGGCTTCCCAGAAGGATAGACCTCGTAGGCCTCCTAGACCTAGTGCGCCCGATGAGCCTGCACGGATAAGTGCTGGTTTGCCTTTGCCAGCTCCCAGCAGGGGAGGTTCTCGGGAGGCGGATAGAGGGGGCCGAGGTGCCAGAGAGAGTGATAAAGTTGATAAACCGGAGAAGGTTCAAGAGGATGAGCAGTTTGAGCAACGGAACAATGATCAATCCCCTTCGCAGGATTACCAGCAGTCTGGTGGGCCTTATGATTCTGCTGGGCCCTTCGAAGGAGGAAGAGGTGACACACAGATGTTCGACCCATTCAGTGGACCTGGTGGTATGCGAGGGCCTCCTTTCGGTGCAGATATGGGCATGCCTCCCGTGTTGATGCCTGTTCCAGGCGCTGGACCTCTTGGGCCTTTCGTTCCTGCTCCTCCAGAGGTTGCCATGCGTTTGTGGAGAGAGCAAGGTGGTGCAGGACCATTTCATCCAGCAGGAGTTTATGATGGCCCATTTGATAGTGAAGGAGGCAACTCTCGTGGCAACAGGAAACGGGCTGGTCCATCAGGAGGTGGTAGAATGGGGGCAGGTTTAATTGACACCCCACCTTTGCCCTTACCTTTACCAAACATGCGACCTGATGCCCGTCGTCCTCTTCGCAGTTATCGGGATCTGGATGCACCGGAAGACGAAGTTACAGTTATTGACTACAGAAGTCTATAAAAGGCTCTGTATGCCATGTGTATTCTTGTCTAAATCAGCAATGACGACGTGGAGTGTAGTGAAAGACCTTCACGTTGTCAACGCACTTTTGTCGTTTTTAAATTGCAGCCATGTAAGATTGTGATCAAGGGCCAATTTGAAAGCCCACTCAGCTGTGCCTAAGTAGGATACATGCATTGAGGTACCCACGTAAGCAATCAAAGTGAAAACTTGAAGACAATTTATGGATTGTCCTCAACTTCTGTTTGTGATGCCAGTCTTCGTGATGTTGGATGCTTCCGCTCATACTAATTTTGCTACTTTTGGGATGGGAATATTGAATCAGTGAAAAATTGTTTCATGTTAAA |
| **HIGH EXPRESSION OF OSMOTICALLY RESPONSIVE GENES 1 (HOS1):**  AAAAAAAACTCGTTTTTTGCTCCACATATTTTACAACCTACGAAATTTTGTTTAGTACTAAGGTCTTATATTCGAAATCTGACTGTTTTTGTCCTCTTAATCAAACCACCAGGCAAAGAGCCCAAACATTGCAGTGCAAGTTTGATTCTTGCTACAGATAAGTTACCCACTCAATGACTACTTCATCAGAATCCGAGAATTCCTGCATGTCCCAATTGCACTCTAGTACAACTCATGTAGCATATACACATGCAATTATCCAGTTTTATTAACCTTGCAACAAAAACCTTGCTTCAAGCATTTCACTACTCAAGTAGTCCTCACTGTGCTTGATCATTTGGCTTGCAGAGGCTTAAGTTCCAGTTTACTTTTTTGAAAGCAAATGTGATCCAAGTGTCTCTGACTTGGTACAATGTCCCCAGAAAAAACCAATTTAAAGCAAGCGTACAACGCAATACAAGACAAGGCCTTCAATAATAAGCTCTCTTTGTAGTCCTTTGAGTTTCAAAACCAGTGATACTGCACCAAGGCTTAAACTCCAGAACCCTGAAAAATAGCTGATCATCCCTAGGATGTATTAGTTTTAAGGCGGCCGGTACAAAGCCTTTGCAAAGAGTGCTAATAGTTAACAACTGAACAGTGTCAGCCTAAATGCAGCAAAATTGCTTCCTAGCTTTTCTAAACTCGAATCTTCCTGGAGCAGGAAGGATCCAAAACATCTGTATCACTGAAGTACAAACAAAACTGAACAGTTATGTCTAGGGGTGTAACAGCCACTCTTCATGCAGCTGCGAAAAGGTAATCTTCGTCACCAAGCCGTAGATCCCTGGATCTTTTCAATGGGAGTAAGCACAATGGCAAGGCAGCTTTTCAGAACCAGATTGATCAGAAGGGTTCTGCTCTACATGATTAACCCCGGCACCAGATTCAAGTGTTCAGAATTAATGCTCTGTTAGTTTAGAACAGCTCTTGCTCATGACCTGATGGCAGCTGACTGCAACAGCCAACCAAAATGTGCAGGCGTACCATTGCACCACTGTGCCAAGTTTTCATCCAACCTGGCAGAACAGTGAAAGACCTTGCAGTGCATCACTGCATTGACCTGTCACAGTTAAAAGAAAATTAACTTATCATCAAACCCACAATCGAGTAAAGCCTTCAGCACAGGTGGCATAACAACACTGCAAGAACCAGTAGAACCTTCCAGTTCAAGCAACTTGAACAGAAGCGCAAACCGATGGTCAAGCCCTGGCATCTGCCGCAGACGTAGCTTCTTTCAACCCCAAGAAGCCCATCAAACTTGTTTTGACAGGTCGGGTTGCCGTCAACTCCAAAATAAAATTAAGAGGCGTTGCTACAATGCATGGCTTCTGAACGATCTACTCCCACTACTTGGCCTCCCAATAGCAGCTGAGGCTTTCGCCCTCCCACCAGATACAGAGGGAGGGGGTAAATCCACGCCATCGTCTTCTGTTCGCCATCTCAGAACATTTTTCCTCCTCGAATATGAGTTCTGGATATCGGCTCCTGATGGCAACAATCCATTTTGCTGCTCACTTCCTGACATTTTCAAGCTGTTTGCAAATACAACATCCCCTGCAGTTGAATCTTGCAAGCAGACATCCAGTTGATCATGGGCTTTTCTCTTTCCATTCGGGCTTGAATGTATCATCTGTCGCCTGGGCCTATATTTGGCCTCTCTAATTGTCAAATCAAATGTCTTATCATTTCTTGGTGACTCTTTCTGTTCCAAGGCTCCATTTTCAGAACACCTGAACAAGGGAGCAGCTGTTCCTGGACCATTATCAGTAAAAGCGGACGCCCCATTCTGCAATCTCTCATTCAAACAGGTCCTACCATCAGTTTCGTGGACATATGATTTTGATTTGTTGAAAGATGGAACAGGTTTAGCAGAACCATCACGCTCCTGAATATGAGCATTTGAAGTGGAAGAAAAAATATTCGGGCTCTCCTCATGAATATAGCCCTTCATATCTTGAGCTAATACTGCTCGCAGATTCCTGGTTGCTAAGGAAGTGCCCTCGCTCTCTGCATTGTAAATCAGTTTCTTGCCTAGAATAGGACTCAAGAGTCCTTCTGAATACTCTATTTGAGGTGTGATGCCACTGGTGGTGGCAAGATCCAGCTGAGACAAGCCATCCGGTACCGCAGATGTAGTAATGAATGGCTTTCCGTGAAGTATAGATGGTTGACGGTAGTTGCCCCATGCTAGTGGATGGCTCATCAAATCAGGTGAAGTACTGTGGGATAAGGGCAGAGAGTCGCTCTTCACATCATAAGAGATAAATGCTTGGTTTGTTGCACCGCGTAACTCAGCTTGTACAGCTGGGAACATTAACTTTAAATTGGGTGCTTCAGAAGCAGCTTCTTTTTTCCTTTCTGTTGAAGGAAGCAATAGATGATCAAGGCTTCCAGTTCGTAACTGATCCTGTTGAATCTTTGGTAGCAACTCAACACCCTTCTCCACCAAAGTAGTTCTTTGTGCGCATACAGTCTTCACAATTTCACTTATTTCCACATCTCTACATTGATCCAAAAATGACCTTTCTATTGCGGAGAGCCTCCTATGCACCACATACGCCTCGCGATTGCGGCACCTCATAATGTAAAACATCACCATAAGACTGCCTGCTGTAGAACATGGGTTTATGGTTGCTTGCTCCAAAAGACATGTCAAAAGAATCTTCTCCTCATCCACGTGCCAAGGAAGCTCTATCATCTTAGCTACCAAGTTTTTCCTTATGCTTAACCAGCAAATTTCTCCCACCAACAGTTCCATTTCTGATGACCAATCTCTAGAATTGTCTTCACTGCCAAATTTTGATTTGGAAATTACATCCCTTTGAGTTTGTCCTTTAACTCTCGATATATATGAGCTCTGATATAGATATGCATCTGTCAAAAGCCCACATTCTAGCCTCACCCTCACAGCAGTTACAGCATCTTGAAGGGCAGGAATTTCATCTCGGTCTACAAATCCTGAAAGCCTTAGGCTCCCATCTCCATTGCTGCAATGCAAAACATCCAAAGCCATATGTGGTTTTTGCCTCTCCAGTAGGACACGAGCAACTTTAGGATGTATATTACAAGAAACGACCTCAGGAAGAAGCTGACATGCCTCCTCCAAAGCCAAGTCATCCTTCTCATCCAGCAAAAAGAAAACCCATGATTCTAGCATTGCATGCCTTGATATGTCAAATGTAAGAATATAATCGTCCAATACCTGCTTCCAATCCTCACTGTGGTTTGGCCAATGTCGGTCAAAAAGATAGTACAAAAAAATTGCTTTTTTGGCCAGAAAGAGGTCTGCATTTGCTTCCAAAAAGAACAAGTCTACAGCTCCGCGCACACTTTCTGGAGGGTATACTGCTGCTGAAACGCGTATGGCAGACTGCCCTCCAATCCCCTCTCTCCTTCTAAAAGGTGTAGAAGCTGTCGTCTGCCTCAAGGACCCTAATTCTAGGTTTTCCCAGAGATATGCATTCTCATTACCTTTTCCGAACCCTACGTTTGACATTGCATCCTCAATAAACAAAGTTGCTGAAGTGTAAGAAGTCACGTCTGATGCATTCCTTGTATGATCAGGCCACACTCTGCTGTTGGCTGCCAACTTACGTCCTTTTACAGCAATTCTCCAATCACTCGGTGAGGGATGAGATGACTGTAGATCCTCAAGAAACTGATGCCTGGCACACCAGCACATCACATCTAGATGCTGAGATACTCTCCGGGTACCCTCTATAAGTTGTTGAAGCTCAAAAATTGGTGTGGAAGTTGCATCTGATATGGGACCTTCCAGAGATTCCAAAATCTGAGACAACCCAAGAAGCTTTGAGGAAAATCTCTCCATATCACCCTCTCTGCTTCTCATCTGTGTGGTTCCTAAACAATATATGTCATGTAAGCTCCGGGTGATGTGTAGAAATGTTTTTTTACACCAGTCTTTGACCACTGTTCCATCCAGGAGCATGGAGACAATTGCATGGCTAGAGACAGCCCTGTCATCCATACAGACATCTGAAACATAATGACAGATCACTGAGACCAATTTGTACTCTAAAGCAATATCAAAAAAGAACCAGAGGCGCTGAACATCAGCATTTAGGTGTCCATCCCTCTCCCTAATATCTTCTGTCCTAGAGCTTATGAGACCAGCCTCACAGCATTCTTCATAAAGGCGTGCTCTCATTGAAGGTCCATGCCTCCTGATTGGTGCTCTGCAAATTGGGCAAACATCACATCTCTGGCCACATTCTGCACATAATGAGGCATGGTTACAGGAAGTCAAAGAGTGCTCAACAATTCTTCCACAGCTTCTCAGGTCCCTTGTTGCTCTGCATTTTTCAACGATCGCTTCACTGCAAAGCTCCCTGGGCTCAAGGCTGACAAGCCGTGACAAAACATCCTGGCATTTGTCTGTGATTCTAGGGTAGAGACGATCTCCATCGTTTTTCTGAAGCGACCACGCGGCTCCCGCCATGCAAGCTTTCCTGCTCTCACTGCAGAAGCTGTAGGCGTATATGCACGAGGGCGTCCGTCTATATGTGCGCGTGTGTTCGTACGTGCGCGTGTGTGTGCGCGCGTTCCAAATGCGAAGCACGTAAAAGCTTCTCGTTGCCCCCTTCAGTATACAAAACACAGTGCGGTGGTGAACCAAAAGACGCCTATGTCAATCTCCCTGTAGTCTCCAAACACTTTAGACTCG |
| **ABA DEFICIENT 3 (ABA3):**  AGAAGTTTTCCCAAGCTATGGGAGCTTCGTGTCTGCTCGAAACCCTTAAAAGCATTCTGCTAACAGTAGCTTTCTACCTGATGAGCTCCTTCTCCTCCTCCTTCGCAGAAGATTTAAGTGCAGAGGAACATGAAGTCCACTATGGAGACCATGGAGGTCCTTCTGCAAGACAGAAAGGCAAAGATGAAGCCAGCATAGAAGTTCCGTGCCATCATGGAAAGCTTTCTGAAGAAGCTGCAGATCATGAAAGCTTCATGGAGCATGGAGCCAGGTGCATTACAGAACATGGAGCCACTTGCCTTACAGAAGAAGATTCACATACAGGCGTTGCAGAACACGGAGAAGAATTTTTTTTCGATGCAGATGATGGAGAGCTGTCTTCAATACATGGAAACTTTCTTTGTAAAGAGAGTGTAGGCACAAAACCTACCTTTGCAGATGGAGGCGTCTTGTCAGAAGACCAAGCAAGCCTTGGAAAACTCTCCTTTGCGGAAGATGATGCAGCGCATAATGAAGACCCTCGCAAGCAGGAGTTCTTTTCACTGTATAAGGAGCAATACGGCTACGCTAAAAGTGCCTTATCTATTGACAAACTGCGCTCCGATGAGTTTCCTCACCTTAAAGGTGATATATATTTGGATCATGCTGGGGCAACTCTTTATTCAAGATGGCAAATCCGCTCCAATTTGGAGGACTTATGTTCAAATTTATATGGGAATCCCCACAGCCAAAGTGGGTGCAGCAACATTTCGTCAAACATGGTTGCAAATGCTCGTGAAGAGGTTCTGAAGTTCTTCAATGTGACAAGAACAGAGTACAAGTGTGTGTTCACTGCTGGAGCAACAGCAGCTCTTAAACTAGTTGGAGAATGTTTTCCTTGGACCACCAAGAGCAGCTTTTGGTACACCATGGAAAACCATAATAGTGTGCTTGGAATAAGAGAATATGCTCTTGCAGCAGGATCCTCAGCCTATGCCATAGAGGTAGAAGATTTTGGTCAGCAACAGAGTTTCAAATTAAGGGCACGCCAATCGCGCACAATAAGCTCTGCTTGCAAGTTAGAGGAAGGCAAGGAGGACAAGAACACCTTGCTCATAGTTAGTTTGACGAGGCAGAGAATTGTTACTGACTTTTGAGCCATGTGTTCTGCAACAGGACTCACATTTAATTTGTTTGCATTTCCTTCGGAGTGCAACTTTTCTGGGAAAAAGTTTAACCTTGATTCAGTCAAGCTCATACAAGATGGAGCTCATGCAAAGGATTCTGTGAACAGGTGGATGGTGTTAATAGATGCTGCAAAGGGTTGTGCAACCTCTCGGCTTGATCTTTCAAAGTACCCAGCAGATTTTGTAGCCATTTCTTTCTACAAAATTTTTGGGTATCCTACCGGTTTAGGGGCGCTTCTCTTACGCAATGAATCTTCTAGGATATTAGAGAAGAGATATTTTGGGGGAGGTACTGTTGCAGTTTCTGTAGCAGATGTAGATTTTGTTCAGAAGAGAGAGAATATTGAGCAGTGGTTGGAGGATGGAACTGTTCCTTTTCTTGGGATTGCTGCTTTACATAGAGGTTTTTCGATTATTAATAGGCTTGGCATCTCTAACATTGGAAGGCATGTTGGAAGCCTTGCGAAATTCACAGCTGCTCAACTATCAGGGCTTAAGCACAAGAATGGGAGCAACGTCTGTGTGCTATATGGCAATCATGATTCTGTGCATTATTGGGGGGATAACTGCAGTCAGGGCCCAATTGTGACATTCAATTTAAAGCGTTTGGATGGGGCATGGGTCGGCCATCGAGAGGTTGAAGAACTTGCATCTTTGAATGGAATCCATCTCCGGACAGGCTGTTTTTGCAATCCTGGTGCATGTTCAAAGTACTTAGACTTATCAGAGTTAGAGATTCGAGCTAACCATGAGGCTGGCCATGTGTGTTGGGATGATCACGACGTTATTGATGGTAAGCCAACTGGAGCAGTTCGAGTGTCTTTTGGCTATATGTCTACCATTGAGGACTCTTTGGCTTTACTTGATTTCATATGCAACTACTTTATGGAAAATCATAACTCTTATCTTGAGAATCAACCAACCCTCCGTGAGCTTGCGAGCTTGGCCTCAAATGATTATTGCAAATCAGATTGCAGTATGTCCTTGGAATCAATTACTATTTATCCTATCAAATCTTGTGGTGGTTTTGCTGTGGATTCGTGGCCACTCGCGGATTCTGGCCTTCTTTACGATCGAGAATGGTTGGTGATGAGCTCTGCAGGTTTTGTGTTAACACAAAAGAAGTGCCCCATGATGTGCCTGTTAGGGACTTACATAGAACAATCAACAAATACACTTCAGATAACGTCTCCAAACATGAAAACAAGACTAGAAATTCCTTTAGTATCTGCACCTCACCAGGAAGCTGTAGTGAGATTTGATTTGTGTGGAGAAGGGTCCATTGGTAGATCATATGAAAAGGAGGTTGCAGACTGGTTCACAGAAGCTTTGGGTACGGCCTGCACACTGGTTAAAAAGCAGCCAAGAAGCAGGCATCTGCGGTTAAGAGGGGGGATATGTGAATCCCAGGCATGCAAGGATGCAAGAGAACTCAGCTATGCAAATGAAGGTCAGTTTCTGCTAGTTTTACGGGCAAGCGTTGAGGATATAAATCGGCGAGCAATCTCATCTTCACAGCGCATCCAATCCAAACAGAAAAAGAAAGCTCACCATTCATCAATGCAGGTAGACGCCATGCGTTTTAGGCCAAACTTCGTGGTATCTGGAGGGTCTGCTTTTGAAGAAGACAATTGGCAATCCCTTGACATTGGAGGTCATAAGTTTATGGTTTTAGGGGGTTGCAACAGATGCCAAATGGTCAATATAGATCAATCAACAGGAAGGTCGCAGGAAGGAAGCAACCCATTACTGACACTTGCCACCTACAGGCGGTCCAAGGGCAAAATACTGTTTGGGCTTCTTTTGGCGCACAATCCTTCACAGCAGCATGAAATAGAACAAATGGAAAGAAATGGTGCCAAAAGTCGACATGAAAGAATCATCAAAACAGGTTGCAGAGTTCATGCAGAAAGGAATCGTACGGCCTAATTTAAAGAAGCTCATTAGAAGCTCGGACTGAACAGCTTCAACGACTTAAGGCCACAGGCATACACTACTGTTTTGGTAGTTTTGCAGGATTTTGGGCTTCATCACTCCTGCAAATTTGTGGCTGGAGGCTTGCTTTGAAGCAATGACATGTAATTAGATCACTTCAAATTTCAGGGATCAGGAACTGGAGAGCTGGCATTGAATCTTATTTGATCACTTCAAAAGTTCAAGGATCAGGAACAGGAGAGCTGCGTTGTTCACCGTCATGAAATCCCAACATGCCAGATGAGTGAATGTCAGCCATCCCATTTCAGTACTACAGTGACATCATCATGTGAACTTACACAGAATAAAGATCCTTTTTTTTTCATCTTCCATTCTAGCGGTTCGACGAGGTTCATTATTGGTTCTCTTGAAGCAGACAGAAACTATCAGTGTGTATAATATGTATATACATTCGCACATAGTCATGTATAAGCAATGGGTTTTCACGTACCATCAGACATTGACAGATCAAATAGTGGCCTGTTAGATAGTCCAACATGAACAATTTTGAAAGAGAAATGCTCAGTGCTGGTTACGTATGAAGTTAATTTTTGAAATTCATCCTGTTGTATTCATTTTGCCAGCATTGTGTTTATGCTATTTGAAGTTGTTAGTCCTCACCATACTAATGACATAGAAG |
| **PYR1-LIKE 5 (PYL5):**  TGAGGACAACTGTTCTCTTATCCTCACAAAGGGCCCCATAGCGAGTCGTCTTCTCCATTCGGGGGGGATGGATGTGAAGCTATCGATGATGCATCTGTGTGTGGCCGCATGGTCACTATGGGGTAGCTTCTCTGCTCTTCTTTCCTGAAGCCCCTCTCCCTGCCTCTCTCCCTGCCTTCCATTCCACAATTTTGCCCCTCTTTGCCTCATCATTTCGTTCGCCCATCTCCTCCTCCTCCTCTGTCTGTTCTTCATTTGGTCGCCCAGATCAATGGCAGTGCATCAGCTGTGGAGGTTCCCAAGGGGAGCAGATGATCTGGAATCTATGCTAAATGAAAGGGAGGCAGTGACTCCGGGTTTGGAGCAGTTAAACAGAGATGAGCAAGCCAACCTACGAGATCTGATTTTCAGGTATCACACCCACTTCATAGCTTCAGGGCAGAGCTGCTCCATGGTGGCACAGAGGATAAAGGCCCCCGTGGAGGCGGTCTGGTCTGTCGTGCGCCGCTTCGACAACCCGCAAGCATATAAGCACTTCATTAGCAGCTGCTTCATGCAAGGAGATGGCCAAGTGGGCAGCACAAGAGAGGTGAGGGTGGTTTCGGGTCTCCCGGCTGCTAGTAGTACTGAGAGGCTCGAAGTCCTGGACGAGGAAAGGCATGTTTTAAGCTTCAAAGTTCTCGGAGGGCAACATCGCCTCAGAAACTATCACTCCACTACTACCCTCCATAGGTATGTGATTGATGGGTGCCCTTCCACTATTGTGATAGAATCTTATGTAGTTGATGTGCCAGAAGGAAATACCAGAGAAGAGACCAGGGTGTTTGCGGACACCATTGTGAAGTCCAACTTGCAATCCCTCGCCCGAACCTCAGAGCTCCTTGTTCGTGACGCACGGAGGCTGGCCTTTCTGGCGTAGATTGGCTCAATTATGGTCTCTTTTGGCGAGCAAAATGGGAGGACCATGAAGGCCACAACACCTTTGCCTAGTTTTATCTCATGCAAATGTGTGAATTCATGGTGGAAAGATTACCGATCTGATTTTTTTTAGTTCCAGATGGCAATGTTCGACTCCCTCTTTTGCCAAAGCTTGGTGATCTTATGAAGGCAGAGCAAATTCGATCACAAGCTAATCACCAAAATTTACACTTGCAGAAAGCATGTCTGATGTCTCCAGTGCACGCTACTTACCATATCGTCACACCATCATTGGAGTGATGCTTTTACAGTGTTCAGAAGTTCTCTTCCAGCCGCCCCTTTTTCATTGGTTTATTGTATGTCACCATTCCAGCAGTGAATGATTGCGTGTTAGTGTATACAGTACCAACCTGTACAGCTGCGTGGGTTTTAGTCTTAGCATCTTTGATGTGTATTATGTCTGGGTATTGTCTACTATGGATAAAGCATGGATGCTATGGTCAGGAACAATGGTCTTCCATAAGGTGAGTGTGTACTTTAAATTTGCAAGCTTTGTCTTCTTTGCAGTGGAATGGATTCTTGTGGTAAAGTGGTACTTCGCTGCATTGTCCTTTCTTATCAGCAAGTTGCATATACTGTTTCGCTGCTTTGTCCTCAGAAAATGCTGCGTAAATGGCCCATCAGTATTGCCTGGAGTTGCTTAGTTAAGTCATGTAAATTGTCAATGACATGATACCCTCTGGGCGAGTTGTTTTTGGAGCCTCCTATGTCCGGGACATTTGATTGTCGATGTCAGTGGTTTTTCATGAGCTAAGAGCTATTTTAAAATTGTAATATGAGATAATTCAGAGGTAGTTTTCA |
| **ULTRAVIOLET HYPERSENSITIVE 1 (UVH1):**  TCTCTCTCTCTCTCTCTCCTTGCTTGCCTGCATTGTTTGAATGACACGGCAACGAGCGGGGGAGCGCGATGCTGCCAATACACGAACACATAGTAGGGGAGCTGGTGCAGGAGGATGGCATAGTGGTGATAGGGGCGGGGCTTGGTTTAGCCAAGGTCCTCGCCTCCGTGCTGCGCCTGCACTCCCCTTCTGAAGGTGTTGTCCTCATTCTCTCTGCCTCTGATGGACAGCGTGCCGCCATCCAAGAAGAGCTTCTTGAGCAAGACCCATCTGTTCCCATCATCTCAGAAATCAATAATGAGACATACTCTGTCTCTGATCGTGTTGCTTTCTACACGCATGGCGGCACTGCTTTCATCACCTCCCGCATCCTAATCGTGGACCTCCTCAATGAACGTATTCCCCTTGCCCGCCTCTCTGGCCTCATCTTCCTCAATGCCCATCGCCTCTCCGACGCATGTACAGAGTCTTTCATTGCCCGCCTCTATCGTGCAGGCAACCGAAAGGGCTTTGTGCGTGCATTTTCTGACAAGCCCCAAGCTGTGATTAGTGGATTCTCGAAAACAGAGCGAATTATGAGGAGTTTGTATGTGCGCAGATTGCATCTCTGGCCTCGATTTCAGATGTCAATTGCGTCGGCGTTGGAAGAACATCCCCCACAAGTTATCGATTTGAGGGTTCCTCTGACATCTTCTATGCATGGCATAAATGCAGCAATTCTTGAGGTCATGGATGCATGCCTGAAAGAGCTCAGAAAAACCAATAAGCTTGATGTTGATGATCTTACTCTTGAAAATGGGCTCTTCAAATCATTTGATGAAATTGTCAGGCGGCAGCTTGACCCTATATGGCATACTCTTAGCAGAAAAATAAAGCAACTTGTCTCAGATTTGAAGACTCTGAGGAAACTTGCTGATTACCTTGTGCGGTATGATGCGATCACATTCTTGAAGTATTTGGATACTCTACGTGCTTCTGAGGATGTGAGATCTGTTTGGATTTTTGCTAACCCAGCACACAAGATATTTGAACTTGCCAAGCGTAGAGTTTTTCAAGTCATTAGGACAGATACAGGCAATCCGATTGTGTCAGAGAAAGGCAACAAGTTTTTTCGTGGCCGGGGTAGACAAAGCAATGGGTCAGATATGGGTGCAAAAAGGAGAAAAAACAGTGAAGGAACTGCATCAGAAGCAACCAATTCAGATGCCAATGCGAGCGTGGACCAAGCGGGAGGCGTGCAAGATGTTGAAGTGGTTGTAATGGCTGAGGAGATGCCGAAATGGAAGGTGTTGCGTGATGTATTGGATGAGATCCAAGAGGAACTACAGCTTATTGATTCAAGTAAGCCAGAGTCCCCTACTTATTCAGATGAAGGGCTTGGAACTGTATTAATTGCATGCAAGGATGAACGCACCTGCCTCCAGTTGCAAGATTGCATTAACAAAGGACCCCAGAAGCTTATGAGTGAGGCCTGGGAAAAATATCTGTTGGGGAAGGCTGAGTTACATGGTATGCGTACCCGGAAAAAAAGAAGCAATTTGGGTTCTAGAGGTGTTGGTGTTTTGAATGGTAAGCTCTCTTCGAGAAATGTAAGCAATGAGGAAGGAAACACAGCAAGGCAGGAAGAGGTAGCTCTTCTAGCAGCAGCTGCAGAGGTGTCATCTCGCGAGGAGGAAAGTGGGACAAGCAAAAGGGGGGGCAGAGGTCGAGGCAGGGGCAG |
| **ELECTRON-TRANSFER FLAVOPROTEIN:UBIQUINONE OXIDOREDUCTASE (ETFQO):**  TGGCAGAGCTGGTACCTAATTGTGCCTGACAAGAACATAAAAGTATGAAAGCACGAAAGTAGGTCTAAAGCCTTTCTTTTTGTAAAAAGAAACTTATTGCTTGTTCTTCCTATACTCATGTTGCTTGAGAGCACAAGGACTCTACGTTTATAAACAATCTACAAGAGGTATTCTTGGTTGACATCACACTTTACATGTAGAGTATACATTCTATACATATTTACAATGTAAATTGTTGAGGGAGCAAACTCCATTGTATATGAGACACATATAAGTGTAAGCTATCCTTGACGAAAGGCCTTAAATATGTATGAACTCTGTCATGTTACAGTCCAAGATAATAAGCTATCCTATCCTTCAGGACAGGTCTCATTAAACATGACTACACTTTGTGAAGTTACATGACCGAATATCCAGGACCGCCACCACCTTCGGGCACAGTCCATTCAATGTTTTGGGAAGGATCCTTTATATCACATGCCTTGCAATGAAGACAGTTTTGAGCGCTGATGTGTAGCTTTAGTTCCCCATCATCATTGCTTACATATTCATAAACACGTGCAGGACAATAGCGTGACTCGAGCCCTGCAAATTTTCGAGCATTCAAGCTTTCTGGAATACATGGATCCTTCAAATGTAGATGTGCAGGCTGGTCATGATCGTGGTTGGTGTTGCTTCTGTAGAGAGAAGTCAGCAAATCAAATGAGATCTTTCCATCAGGCTTTGAATACATCTTCTCTTTATGGATGTATGCAACTTCTGTAGCTTCATGGTCAGGCTTCCCATGCTTCAAAGTCCAGGGCAATCGGCCTTGAGAGATATACCGCTCAAACGCCGAAATTGCGAGTCCTGGTAACATGCCATACCGAAATGCAGGTCGAAAATTTCGTGCTTTGTACAGCTCCTCCCATACCCATGAGTTCTTCAAAGCACTCCAATATCCCTCCATATTTGGCTCAGACTCTGATTCTTCCAAAAATGTAGTAAAAGCAGCTTCAGCGGCAAGCATTCCTGATTTCATTGCAGTATGTGTGCCTTTAATCTTTGGGACATTTAGAAAACCAGCAGAGCATCCTATTAGTGCACCCCCTGGAAAAATGGGTTTCGGTATGGACTGGAATCCCCCTTCATTTAAAGTACGAGCTCCATATTGTATGGCAGTACCACCTTCCAACAAAGATCTTATTGATGGATGTGTTTTGAATTTCTGGAATTCCTCGTAAATATTAAGAAATGGATTCTTGTAATCAAGAGCCACAACAAGTCCCATTGCAACCTTACAATTATCCATGTGATACACAAAAGATCCACCATAGGTTTTGCTATCCAATGGCCACCCAATTGTGTGCAAAACTAAGCCAGGTTGGTGCTTAGTTTGCTCCACTTCCCAGACTTCCTTTACACCCAGTGCATAAGTCTGATGTTGAGCCTGTGCAAGCTCCCTTAAACAGAACTTTTTGATAAGCTTCTGCGAGAGAGAACCTCGACAACCTTCCCCAAATAATGTAAGCCGTCCTCTCAACTCAACACCTCGCTGAAAAGTTGACTTTCTGTCCCCATCTTTGGAAATGCCCATATCGTTTGTTGCAATGCCAACAACTTTGCTTCCTCCTTCATCAAAAAGTATCTCACTACCAGCAAACCCTGGATAAATTTCAACTCCTAGGTCTTCTGCTTTTTTTGCGAGCCATCGCACAAGCTCACTCAAGCTTATTACAAAATTGCCATCGTTTTTAAAGGGAGATGGTAATGGCACAGCACGGTTAGCAGTAAGCCACCAAAACTTGTCTGTTGAAACAGGTACGCTTATTGGAGCTCCTTGATGTTTCCAATCTGGTAGAAGCTCATCGAGTGCACGTGTTTCAAGGACATTGCCGGAAATAATGTGCGCTCCTATTTCGGCTCCTTTTTCGATGATGCAGACAGAGACGTCTTTGCCATGCTGTTTGGATAGCTGTTTGAGTTTGATGGCTGCAGAGAGGCCGGAAGGGCCTGCGCCCACAATCACCACATCAAAATCCATGCGCTCTCGTTCTCCCGGGACTGCCTGCGAGCAAAATCCTAGTGCCTGCAGGGGCTGCCATGGCGACCTCTTTGAAAGCAAACCGCCGCGACCTCTTGTGGCAGAGGCAAGCCATCGTAGCTGCTGCATTGTTGTGGGTGCGCGTCTGCGTGTGCGTGTGTGTGAGGTGTGTGTGTGAGAGTGTGTGTGTAGTGAGGCGGAAAGCTCAATCAATGTGCAGGAGAGGGAATTGCAGCATCTTATGAGGAACGAGAAGAAGGCGCGCCATAGCCGACACAGAGCTGCAGGATCTCAAGCTTGCTTAGGGGAGGGGTAAAGCGAAAAGAGAAGAGAAAATCTGAGAAAGTAGCACGCATTACAAAACCCAAAAGAAAAGGAGAAGGAAGCTTTCTG |
| **Y-FAMILY DNA POLYMERASE H (POLH):**  GTTTTTTTGGAAGAAAAAAGGCCATGGAGAAAGAAACGCCTTCTTCTCCTTCTGTAATACTGCATGTGGACCTTGACTGCTTCTACGCAGCGGTGGAGCAGGTGAGGCTTGGGATCCCTTCAGAAAGGCCTCTCGCTGTGCAGCAATGGGAGGGTCTCATTGCAGTCAATTATGCTGCCCGAGCTGCTGGAATTGTCCGCCATGACCGAGTCACAGACGCTTTGCACAAGTGTCCAGATTTGCAGCTTGTGCATGTTGAAACTATTGGGAATGATACATGGGATGGTGCAGCTGCTAGACGTGACACCGCAAAGGTGTCGCTGGAAAGGTACCGACAGGCGTCAAGGTGCGTATTTGATATATTTCATGAACATGCTGATATCTGTGAGCGGGCAAGCATTGATGAAGCCTATTTGGATGTTACCAAGAAGGTTGAACTTTTCATGAAGAAAAATTGCAATTGGGACCAAGAATTAGAACGTTTGGCTGGTAGTCAACGTCTGCTAGGGGGCTCGGTTGAAAAAGCTGGCATGGTTGTAGAGAATGGAGCAGTTGAGATTTGCGATCCTGTTAGTAGGAGGCTACTGGCTGGAGCAATTATGGCTGAGCAGATACGTACTGATGTCAGATCAAAGTTGGGGTACACTTGCTCTGTGGGAATTGCAACCAATAAACTTCTGGCCAAGATAGCATCAGCTAGGAATAAGCCAGATCGACAAACATTGATTCTACCAGGGGCAGTTGCCGATTTGATGGATGCGCTTCCTTTAAAGAAAGTCAAACTTCTGGGCGGTAAAAACGGTGAGGAACTTTCTGAGAAATGGAGTTGCAAGACAGCTGGGGATGCTCAGACAATACCTTTAAAAAGTCTATTAGCGTCCTTTGGCGACCGTTTGGGAAATTATGTGTATAGAGCAGTGCGAGGAATTCATGAGGACAAAGTACAAGAAAAGCAGACAACTAAGTCCATGCTCGCAGCCAAGTCTTTTCAGGCTACAAACGATCTTTCTGTTATCAGGATGTGGCTTGGTGTCCTAGCTGAGGAGTTATCAATCAGGATGCTGCGTGATGCTACAGAAAATCATCGTCAACCTCACAATTTGCAGCTATACTACCGATCAGGATTATCTAGGCAAAGCGGAGACCATTCAAAGTCATGTTCTATGCCGCATGCAGTGATACAACTACTCATGTCATCATGCTTCAGAACTCCAAGAGCATCTGCCAGTAGGCAGCATGCTAGTGAAGCAACGGGATTATCTATTAAGTGTGACAAAGAATTCAGTGGTGAAGGAGTGGCAAAGTTTAAAGAGGAGACCAAGTGTGATAAGTCGGAAGTACCTCAATGCATTGAAGGTGAGGCTGAAAGAGAAGTACTTCTATTCAAGGAAGAATCTGGCTTTCTCGACCCCGAGGATAAAAGATTATTCACGGAAGAGTGTGGGAGAAATATGAAATACAATACAGTTGAAGATCAAACAGCCAAGGAGTTGTCCAAATTGTTGCAGCAAGTTTCCCTTCAGCTATTTGAGCGCATTGAAGATGCGTTCCCATGCACTCGTTTAGCCATTGCTGCCAGCAGTTTTCATGATGCACCAGCACAGGGTTCTCAATCTATCCAGCATTTTTTTGGTAGTGCCTTTCAAGGTTCCAAGGTGCCTTCTGTCAAGTCTTCGATGGAGGGACCTGTAGGGGAAAATAAACTTTTAAGTAAGCGTAAAGGTTTGCTAAGATATATGGATAATGGCACTGGTCTCAAGAAGCCTTGTGCAACTTCTTTGAATGAGTTGAAAGGGCTATACTGTGTGACGGAAGGTATTGAGGAACTCTCGCACAATGAAAATCCGAGGGACAGCTCTTCAGTAGAGAAAGATAGCTGTAGAAGTTGGGTTGTTGAACAGGCAAAAGAGTTATTAGAGGATGAGGTGGTTTTGCCGGCATTGGAGGTGCTGGATTCTGGTCGGTGCTGTGAAGACCCAATGGATCAACAAGAAGTAAAGCCAAGGGATGGTTTGTTTGTTCAATGTGCGCATCCGTCATCAGAAGTGCCCGGTTATGGTGGGAGCTGTGAAGATCCGAAGGATCAACAAGGCGCACCTTCCGATGTAGGGTGCGGTATCAATTTGAAGGACATAGATGTGGATGAACAATGCCGCATTCTTGAGGAAATATGTAAGGCTAGGTCAGCTTCTCAAGTCAGCAGCATGTCTTCACGTGCTAAATGTGTGCAGTCTCAAGAGACACGAGCTATAAAGCGCAGAAAGCCCAAGAGCTCTGATTTCAAGTCTGGCCAATTGTCCTTGTCTAAATATTTTCACTCTTCTGATGCTCAAGGATGAAATGATCCAATTGTACAACCAATGGAGATTGTAAAATGGCCACCAAAGGTTTATAGAGAATTTTTCACTCACATAAGGTTTTGTTGACATGCAATATTTATGCTGAGGTTTAGTGTATAGGCTACCTTGTGGTGGTGCATTCTTAAATTGTAATCATGATGTCCTACTATATAATTACACCCAATGCTAAGTTTTGCAACCTTATGCATTCTTAAATTGTAATCATGATGTCCTACTATATAATTACACCCAATGCTAAGTTTTGCAACCTTA |
| **DAMAGED DNA BINDING 2 (DDB2):**  GAGAGAGAGAGAGAGAGAAGCTTGCAAGCGTTTTGCACAGTTGTGGGCTATGGCGGGAAGGCCTCAGAGGGCTGTGCGCAAGGTTGTGGTGCAGAGGGATAGCGAGTCTGAGGAAAGCTCCGATGAAGAAGTGGAGAACGACCCTCTTGACGAGGAGGAAGTCGCCGCCGATGGTGAAGAACTCAATGATGGTGAAGAAGATGATGCAGAGGACAATGTAGCAACTCCATCTTCCTCCACGCTGAATGAGAAGAGAAAGCAAAAGAAACCCATCACCATTAGCCTTAAAGCAGCTAAATTGTGCAAGGTATGTAAGGCCAAGGATCATCAAGCTGGTTTTGTGGGTTCTATTTACTTAGACTGCCCTAACAAGCCTTGCTTTCTCTGCAAACAGCCTGGACACACAACTGCAACATGTCCCCATCGCATTGCATCCGAGCATGGAGTCACACCTGCTCCAAGAAGACAATCTTTGGGGATGATCGATTTTGTGCACGAAAGGCAATTGCGAAGTCGAGTCCTGAAGATGGTGCCACCCCCTGTCATTCCAAATCGTGTGGATTGTGCCATAGTTAAGCTACACAGCCGGCGGGTCACAAATTTAGAATTTCATCCGACTAAAGATAACATACTTATATCTGGAGATAAGAAAGGTCAAATTGGTATTTGGGACTTTGAGAAAGTTTACGAAAAGACTGTATACAGCTCAATTCATACTTGTATAGTAGGTAGCATAAGGTTCCATCCGGCAAATTCTGAGATGATATATAGCTGTGGTGCTGATGGTTTGGTCAGCTGCACTGACCTGGAAACAGGGCTTCCTAATAAAGCAATTGATCTAAACCCGGATGGATGGAATGGCCCTTCGACATGGCGGATGATTTATGGAATGGATATCAATCCAACTAGGAATGTTGTACTTGCAAGTGATAACACTGGGTTTTTGCACCAGGTTGACATAAGGACAAACAGTACAATAGGAAAGCCATTATTAATACACAAAAAGGGATCCAAGGTGGTTGGCTTGCATTGCAACCCAGTTGATTCAGACCTCTTCATCAGCAGTGGCAATGACCACATGGCACGAATTTGGGATATGCGCTTTTTGGATTCCCAAAGTCCTCTGGCAGTACTGCCACATCCAAGAGTGGTGAATTCAGCGTACTTTTCGCCTGCAACTGGAAACAAGATCTTAACAACTTGTCAGGATAATCGACTACGAGTTTATGATTGCATTTTTAGTAACTTGGCAGAGCCTAGCAGAGAGATTGTACACAGTCATGACTTCAACCGCTATCTTACATGCTTTCGAGCTGAATGGGACCCAAAGGATACCAGTGAAAATCTGGCAGTCATTGGCCGTTACATAAGTGACAACTTTGATGGTGTTGCATTGCATCCCATTGACTTCATTGATACTAGCACAGGTCAGCTTGTAGCTGAGGCTGTAGACAAGAACATCACGACCATTACTCCTGTAAATAAGCTGCACCCTCGATTAGATGTCCTCGCTTCTGGCAGTTCAAGATCGTTGTATATCTGGCGGCCAAAAGATGAGATGGAGGATGATGACAAGGCAAGGGAAGCTGAAGATGAAGTTAAGAAGAAGATCACCATTTTCACTGTGCCGGATGATGCTGCTGCAAAGAAAGGGAAGGATAAGAGGAAGTTTGATGGAGATGATGATGAAGATGATGACATGCTTTGCAATGGGAAGAAAGGGAAAGGCAAGGCTGTGCGCCCGTCGCCTACAGCGAAACAAAAAGCGCGCTGAGGGCTCTTCCATTTTTCCATCTCATAGATTTAGTCTCACATCCCCGGGTGGACATTTCTGTACATTATAGGCTAGGATTTTTGTTTCTGTAAACCATTTAGTATATGGTGCGTAAGCAACTATCTTCTAATTTGAATATGATGTTTATGGTTTGTCATGCAAACTCTCTTTTCTCTTTTGCCCAAGGGAACATCAAGGTGAGGTGTATACTTGTGACATCCAACGACAAATGTGATGCCACGAC |
| **STRESS ENHANCED PROTEIN 2 (SEP2):**  ACACACACACACACACACACCAACTCATGCTCTGTTTGCTGCTGTCTTTGCCCCTTCGAGGGCGCCATGGTGTCGTCGTCGTGTGCGAGGAAGCATTGTCGGCGAAGAAAGCATCTTTATCATCATCATCATCATCATACTCACCCCATGTATCCAGCTACCCCTTGCACCAGTCAAAACAGGGGGTCTACTTACCTCCCAGACTCTGCAGCTTGAATGAGTTCGGTACGCGCATGGCTTCCGATCGCTCTCATGACAAAACCCACCTCGATTTCTTCACATCGCTTTCTGCGATCATCGAGCTCTCTGCCCAAGTCCAGAATTGGGAAGTACTCTCTGGTCGGCTTGCCATGATGGTGTTTGCATCAGCTCTAATGCTAGAGGCTTTGACTGGCAATAGTGTGTTTGAGAAGATGGAAACGCAAAGACTTCTTGAATATTGTGGTGCTACTTTTGTTTCCATTCTCGTTGCAGCGGGATTTGCGGTTGCGTTGCAAGCAAAGGCTCATGTGGCATACACAGTATCTAAAGGTTATGAGAAGTTAATAAATGGGCTTGTCGATAATGTCATCGATAATCTTCTCTTTGATGATGAAGTATGCTAGCTCTCTGTCTTGAGAAGCAGCAGCAAACTTCTCTTATGTCACCTTTCTAGAGGTATTACTGCGGACCCAAGCAAGCTTTAGCAATATGAAGCTTGTTTCTTGTAAATATGGAGGTATGTGGCACAAAGAACTGCCACTTCCAGATGTTCTATATTTAGTGCAAGCCAATATTTGTTGGGGCATGACTTGTTGGGCTTTAATCATGGCACCACCCAGCTATAGAGTTGCATGACCTTTGATTTGGTTAGATCGGTCGAAACTCTTGTTTTCAAATCACCGCAGTGGAACCGTGTGTAAAGATGCTGCCCTTTTATGATCTGAATGCAAGGTCATGTTAAGTGTTCATAATCAATAGTTTTTGTAATTTTCATTTGTGTTGATTTGTAAATTTTAGATCTCAAGAGGCTTGTCAAATGAGGGG |
| **SALT OVERLY SENSITIVE 1 (SOS1):**  CCGCCAAGAGCAACCCTGGACAGGTAAGCCACAACAGTTGCTGCATTGAAAGTCTGACCCACCACCAGCTCCAAGAACAACCGAAAGATAACGATTGCAGTTCCATCATTTATCAATGACTCTCCCTCAATCAAAGTATTCAGTTTTTTTGCCTGCACCAAGCTCCTTGAGAAGTGCAACAACTGCAACCGGATCTGTTGCACTTAACAACCCTCCTAATAATAACGACGTGCTCCAGGTCCATTGGTATGGAAAGGC |
| **HISTIDINE KINASE 1 (HK1):**  TTTTTTTGAAAAAATTGATTACTTGTATTCAACCTTGCACAACCATTGCAATGATATAGAGGTAGCTCCAACTAGACAAACTGGGAAAAAGAAGAGATTACAAGGGCATGAAGACCCCTGGGAAAATACACATGCCTTTTACATGAAAGGAGTTTGATGGCAACGCACTTACACTGGCGCACACATTTCTCATATACGCAGTGCCGTGTGTTGGTCTCCACTAAGATAATGCCAAATATGTGAAAGGAATTTTGAGAGAACAAGAGCATATGGCCATTGGTAATTTGCCTACTTCTCTGCACATCATCCTCACGAGACAACACAATATTTTTACAGTTGTACACGCTTATGGTGTCCATAACCATGACCTCTCAAAATTCTACATCTCTTTGCGACGTCTTGCCAAGCTATAGATTTTCCATCAGCAATGGCATTTTACCCTTTTTGTAAGGTTGATATTTATTTCAGTACTCATGGAGAACGGTGAACCAATTCGCACATTGTACGGCCCCGGACACTCATGCCACGATTCTCGGATATGCATTGCACACTCCATATAATGCACTATTGCCGGCGCTGTACACATTGTGTCAACATACATACTCCTCCAAGAAATTTAGCATTTGAAACATATTCCTTAACTGCCAACTTGATCAACATACAGTAGCACCTGTCATGTGACTTCCGTACTTCTCCCCAATAATTTGGTGACCACATCAAGTAAGGCATCGATTGATAGTGGCTTTGTTAGGTAGAAATCCATACCCGCCTCTATACATTTCTTCTCATCTTGAGCCATCGCATGAGCTGTCAAAGCAATGACAGGTGTGCGAATACCATACCTGCTTTCTTCACTACGGATACGTTGTGTTGCTTCATATCCATTCAAAATAGGCATCTCACAGTCCATTAGAACCAGATTGAAAGGTTGATTTTTCTCAGCACTTCTCAATTCTCCTGCCAGCTTTACATCAGTGCCTTCTGGAGGAGAAACTTTGCTCCTATACAAGTTCTCTAGAACTAGCCGAGCAGCTTCAGCACCATTATCAACGCATTCAACAGTGGCCCCAAGGCGAATTAGTTGTGTTTTTGTTAACCGCTGGAGGATTGCGTTATCCTCGGCCACAAGTATGTGCATGTCAGACAGGGCATTCTGGAATCTGACTCTAGAAGTTGAAAGATTCTCTGAGCTTTTATGAACTAAGGAATCAGGAGCTGATGACAATGGCTGATCTTTTCTTTTTGATTTAGTATTGTCAGGAGAATCCATCAGCTGACCTACTTGGGAATCCAAGGTCGCCAAATTTGGTAAGCTATTCATCATATTAGAAGCATTCCTTGTCACAAGCGCAACTGAATGAGCTTTAAGGTTTCTCTTTGCATCCAAGCTTGATTGGAGACTTCCTGTATCTTGCTGGAAAGTGCTCATACAAGAGAGAGATGTATACAATGAGTTTGCAGCACTTAGGTCTACCTTTTCACGGTTCCTTGTAGACAAATGTGGAGTTTCCGCTTCAAAAGAGTCCCGCACTCCATTGCCGACCAACTCTTGTAGTAGATCCCAGACACACTTTAAGCGAGACGCATACAGTGGCTTATGCAGGATCAGATTGCAAACTCTCGTTTGCCTTCTTAAAAACTGAATGTCAATGCTGGGAGTATTGGCATTCACTAACCATGCAACCCTGAACGGGAGAGATTGGTCACTCTCCACTAGCATTTCTTCCAAATCTGAGCACAAATTATCAAGAGATGTTATGGCAAAGGTAGCATCTACAATTAATAACAGATGTGCCTGTGCGCCATAGAGGTTAAGCTTTTGAGCAGATCCATTGCCATCAGATGTGGGCAATCCTTGCAAGCAAACATCAAAGCTAGTTTCATTCCTTGCAAAGTTTTTCGCATCGAGTTCATCCACCCAGGAGTCATATATGTGACTTTCAGGTGACCTTTGGCCCGGCCTTTCAGACAAACTGAAGCTTGCAGAAAAAACTTCATGTTTAATTCTTTCGATTGCAGGTGCAAGCTCATCCCAGTTTGCAATTGTCCATACCTGTAATCCCCTCTTCTCCATCCACTTCTTCACTACCCTTTTCCCTGCCTGGCCCTGCATGGCAAGTAGAACATGGACACCTTCCATTATAGGAGGGGAGATGAAGCCAGTTTCCTCCAAAGTATTAGAAAATACAAAGGCTCCAGTAGGGTCTAGCTGCCAACCAGGAGAAACAGAATCACCACTAACACCAAATGTTGCTTGTAAGGAAGCCACATTTTGCAGTTGGTTGTCCGGTGTGCTGTTGTCTTCAGGCACATGTTTAGCGTGCTGCTTTCCACACTTGAAAATCAGATTAAATCGAAATCGAGTTCCAGGTTCACCAGGTTCATCTTTGTCAATGATGTTGATGTCTCCTCCCATTAACCTTACCAGTGATCTCACTATGCCAAGGCCAAGGCCTGTTCCGTCATAGTTCCTTGGTCCAGATGACTCAACTTGTACAAAATTTTCAAATACAGATTTCCATCGTTCTTTTGGTATGCCCCTTCCTGTGTCATCCACCTCAAACTCCACATGCATGTAGTTGTCATCCTGATCTTGCTCAGATAAACCCTGAATTAGCTTACGTATTTCCTTTTCCCTTGACGGGGACCTTATAAGTTGATTCCATGGCCATGACAAAAGCCTTCCCCGACAACTAAACTGTTGTCTCGTGCCCGAAATGCTGAGTGGTTTTACCCGTCCTCGCAGGACAACATGCCCTTCAGAAGTGAACTTTACTCCGTTGCTTAGAAGGTTGGCCATGATCTGCTTCACTCGTCCAACATCCCCTATCACCCACGAAGCCTTCTCCACAAAACCATCTGATAGGTCCAAGGCAATTTCTATCCCCTTCTTCGAGCCCACAACAGAAAATGTGTCTATCACTTCTTCAAGGGCACTCACCAGCTCAAACTCCGACTCCTGCAACAAGAGCTTTCCTGCTTCTATCTTGCTCATGTCCAGGATTGAGTTCAAAATGCCCAGTAGGTTGGTGGCACAGGATCTCATTTGGATCAAATTGGACCCCAGTTCCGAAAATTCAGAAGCATCACAAAGGCAAAGATCAATTAGCCCAAGAATAGCCGCCAAAGGAGTCCGAAGATCATGACTCATGTTGGCAAACACTAGACTCTTGTGGCCACTCTTTGTCTCAGCCCTCTTTGTCTCCTCCAACTGCTGTATTAATGCAGCCCTTAACCGCATCTCATTGCTAACACCTTTGGTCAGAAGCGTGACAAAGAAACACCCTACAATCCCCATACTGGTAGCCAATGCAACCAATACAGCTAGAGTAATGTGGCTCCACTTCTCTGTCAAGCCCCAAACACTGGCGTAAGGGAGTACCACAGTACACACCTGCATGTACGTTGCATACAGATGTCAAAACTTATATGCCGACATCCATTGCCATGACAAACCTGTTTATTCTTGGAGGAACTGAATTCTTTTTTTGTTTCTTTGAGCAAGTAAAACAACGCTAGAGGCATACCAATGTTGCTCCAGAAATGTGCAGAGGTGCACTGTCGATGATGTAACGCGCTCCATGTATCTCCAATTTCATTACAGTGAAGGATTGGCTTTCCATGGTTGATATGTAAAAGAGGTGCGGCATTAAATATTCTGCAGCACCTGCAACAAGAGCATTATCGGACTGAGTTGCCATTGTCTCTTTTTGGGAGGTGTTATCCATCTTCGTGGTTGAAAAGCCAGCTCCCGTCTCGGCCACTAAATGACCATCTGTAGTGGCAAGAAAAATGGCACCCCCTTGAAAGTCAAATTGATTGAGGAGGTTACTGATGTCTGGTGTAGGTACACCAACCGAGACAGCAGCCACTGGAATTGCAGATTGGCTTTGAAGGACTGGAGATAAAAAGGAGAATGAAACCTCCCCGGAATCTATCGGTAAAGAACCCCAGCTTGCCGTTCCATTTTTACCCTGCAATGAGCTTTTCACTACTTCATTGTCCCAGTATGCTATAGGGGGTACTTGCAATGCTTCTCCAGTTGGGTAACCTGTAAGGTCATTGACTTTCTGATGGTACCACTGATACCAAGGGCGTGTGTCCGCCATATCCTCTCCAGTCTTCCATGGGATTGCAGGTGCATTTGAGGACTTAGAATAGTCGCGAGAGAGATAAGATGTGTTAGCATAAGTGAGATATGTTCCTGTAACATCTGTTGCATAAGATAGCAGTACCCCACTTGCTCCAAAGAACGTGACTCGTGCTTTCTTGGCAAGAAACGAGAAGACTAGAAATAGAACAGGCCTCACCTGGTTGTGTAGAATTGTTACAGAGGATAGGCTGTCTTGTGTAAGTGAACTGATGAATGTGGCCAGAGAGGCCGCAGATGCATCATTCCCGTGAAGCACAGCATTCACATTGTCCTTGCTGTGAAGAAGTAGCTCACGGCGGAACTTTGAGGCTAGAGAACCCACAGAATGCTTCACATTGGTGGACGTGAGCACCCACGTGAGCACCGTCATGATAATTAGCAATATGGCCAGCAGCCCCATCAAAGCGAGCCTGGAAGTGAGAAGTGGGTTGCACATGACTCCACAGCGAGACCGTGTTGAAGCATTGCCTTTCCAACAATTCAGGAGCCGCCAGATACGGCAGTCCTGCATCCCTTCATGAGAATCATCGTCTTTGTCATCATCAATGTCCTTGTCCTTTTTGTCTGTGAGCTTCTTCTCGCTGTTGTGTCCCTTCTCCATATCCTCAGACATGCTTGTGGCCCATGGCATGTCAAGAACAGCTTGTTCCTGGCATAAAAGCCCAATATCATAATTGAAGAAGGTGTCTGCAGCACGTGCCTTTGCTGGAGAGGGAGAGGAACCCAAAAAAGAGACCGGGCAGCAGGAGTTCGCAGAAGCAAAATGGCGTCTTTCGAGCAAGTCTTTGTGCATCTTGGTAATTGTTAAATATACTAATGCTAATAATCAGAGAAGCATCTGCATGTCAGTGGAGCAGAACTAAAGCATTTGAAGATGCTCGAATACAGAATCCATTGCGTCCTGTCGAAAGCCAAGTCAGGTCGAGTTTGTAGTACATAGTTCAAAGACAAGCTAGGCCTTTATCTTCTTCTGCAATGTACTGTGAATGTGAGCAAGACAAATGCATACCAAGATGAGCATGTGGAGTACAAGCCAGTGTCTAGCGCACACACAATTCTGTTCAATCAACTGGAAAAGAACAGGAAGGGACTCCGAGGGAGGCAAGATTGGGAGCAGAAAAAGAAAGAGAGAAGACACTGTGGGAAAAGAGAGCAAAGCAGAGGACGGGGTAGATCAAACACCGAGTATGAAAGGGACACTCTTGGAGGAGGCACACCAGTGATCAGTGTGCTGGCAAGGCAAGGGGGGGGGGGGAAGAGGGGGAGAGGGGGAGAGA |
| **IRON REGULATED 2 (IREG2):**  CGAAGTATCCAAGTTGGTAAGTTGGTAAGAGCAATAAGTGATAAACTTGAGGTTGTATACAAAGAGCTAAGGTACCCTTAATTGGCCCCTTTACGAGACTCAAAGTTCTAAGCAATTGAAGAAAAAAGCTCTTATAAATATTGTATCATTAATATTTACATATATACATAAACATGGCCTTGGGATACTTCAGCAATAGGTTTTAAAAAAACCATCAATCAAATTGATTCTACAGCCTACCCTTAAACCGCTCACCTCCACTAAAAAGATACCGCAATGATTGAGGTCTTCTCGTGTGGAAGCTCTACCGTGACTTGAAGATGCGATCCAAATGCAGCAAATGGCCTCGAACACGATATACTTGAAGAGAGTAGAGTATGAGGGACATTGTCACGGCCCCATAAGACAAGTAGACCAGCTTATCAAAGTCCTTGGGGTTGGAGATCAGCATGCCAGCAACAAAAGAGAGCATCTCTAAGAAAGATTGCATCGAATTTTGAACTCCTCCAACTATTCCACGTTCATATTCAGGGGTAGATTCCTGCATAAGTTGCAACACTGCCAAATCAAACATCCAAAGACCAAAACGTGAAGTCACGACACCCCCCATAAGAAGAGCAGAGGATATGGTTGAGTTAGATATCCAATTTGAAGTCACACAAAATGAAAGGAAACTCCACTGCATCACAATGGACCAAAGCCCTGTTCTTATTGTCTGTAGACGGCCATGAAATTTTGGATAAGCGAAAGTTGCACAAATACCAACTAACGCAGAAAATCCTCGTACAAGCCCTAAGATGTAAGAGGAAGTGCCTCTCCAACTCAACGCAGCTGTCATTAAAGACCCAAAGCTTAAAACTGTAAAATACAACATGGAAAGTGCCACAGCTGCTAGTAGCATTTCCTGTGCTGCATACACTTTAAAACCTTCGACAAAATGTAGTTGTAAAACTCTCTGGTGAAGAATTGAGATCCATTGACACGCTATTTCCCACAATCTTTGAGACGAATATGTTGTATGAGATCTATCTCCACTTTGAAAATTGTTGACTGATTCATTTGTCCTCCCAATCTCCTCAGAAACACAACTACTCACAAGCTTATCAGTCGCAGGATCAATGCTCTCTCTGGCAATGTTTGGTACATGCATTTCTATGTCATTACTGTTATCAATTACAATCCTTTTTTGCTGAAGTTCTAGAGATTCGTTGTACAAGCACCTCAGCAAATAGTACTCGAACCCAATAGACACAACATTCCATACACATAATAAAATGGCAGCTGCCAGAACTGATACAAAGCTCATTATCATTCCCACAAGCACAGGAGCCATAAGCTTGCACGTAAGATCAATGCCTCGCATTGCTGAGTTTAGTTCTGTGAGTGTTGCTATGTGCTGTCCTTCTGCTACAGCAAGCACCCAATCTCTCTCAACAGCAATGTTTGTAGCAAGGGCTGATAGGGCCGAGAGTCCACCAAATAAATTTACAAGGACAATGAGACTAGCAAATGTTTTATAACGTCCAAATGCAAAGTCCCGATCGAGAAGCAATATTGCTAAGGCCAATGCTGCAGCCACAACTGACCCATTCCTTGTTCCCAAAGAAATTTGCAACACCTTGAGGCGTGGGGTTCTATCCACCCATTTTCCAACAGTGATGCCCATGGAAACCACAGCCAAGTTTTCTACAAGCCCGTACAAAGAGGTGAGAAGTAATGAGTTTGGCCAAATCGCAATCATGAATAACCCAATCCCAAACTCCCACATTCTGGTACCCCAGCGTGTGAGGAAGTGGCTGACGTAGAGGTAAGCAAGAAGACGGAATCTGGAAGGATACAAGCCTTTGAAGCTTCCATGACGGTCCTGCGTAGTTAGAGGTTCTTCCTCCTCCCGCTGCTCCTCCATTTTTTCTCGTTGCTTCTCTCACTCTCTCTCACTCTCTCTCTCT |
| **PHOSPHATE TRANSPORTER TRAFFIC FACILITATOR1 (PHF1):**  TTATTATCGAGATCGCCGTCGACAGCAGCTGCAGAGAGTATATTAGCAGAGTATATTTCTGGCATCTTCTTTCGATATTTTGCACGAACGCACGCACGCACGCACACAAAGCTCTTCGCATCTACAGGACCTCTCTAAGCGCAAGGATCTACACAGGCTTCCATAATTTTGGCAAGGAAATCTGTGTTGTCAATTAGGGTTTCTGCAATGCCACGCACTCGCTACATGCGGGTCGTTAAGAAGTGCGACTCCCCGCTTCTGTGTGCTTCTTGGACCGCTGGCAGCCCCAACTCTCAGAAAGAGGACATTGAATACATTGTCTGTGGGAAGACTTCAAGTGAAACAGCTCCTCATGGCAGCTTAGAGCTCACCCGCTATGACTTCAAGGAGGACTGTATATCAGAGGCGGTTCACAATCTAGTGGAAGATGATGTACCTCAGACTCTAGCAGTTCATCCACGAGGGGATGGTGTTATATGCTCGTTTTCAAATTCTTGCAAGCTGTTTGGGTTGGAGGGGCTAAAACTTAAAGCTTTGGATCAAGGTCTTGTCCCTTTGCAGGGCATTGGAACACAAGGAAATCTGGTTTTTAGTGCAGATGGGTCTAGACTAGCAGCCGGTGGAAAGGATGGGCACCTGCGAGTCTTTGAGTGGCCAAGTTTAAATCTTTTGTTTGATCAGCCTGATGGCTGCAAGTTTCTTAGCAGTCTTGACATCAGCTTAGATGGTGCTTTTCTAGCTGCCATACCGGATGGTGGGAACTACTGCCGTGTCTGGGAAATTGAGAAGTCAACAATTGTAACAACAATTCAGAGCAGCAACAAGAATGAAATGTTTGGATCGTGTAAGTTTTCAAGGGATGGAACAAAGCCATTCTTGTTTATAACCATCTCTAAAGGTGGAAAAGGTGTTATTGGAGTCTGGGAGATGGAAGCATGGAACAAAGTGGGCGTAAAGGCGCTCTCACAAGATCCCATTACATCATTATCCATCAGTCACAATGGCAAGTGGCTCGCAATTGGATGTGCTGCTGGGGATGTTCATGTTATTGAAGTGAAAAAAATGGAGACCTCTGAGCTTGTAAAAGATGCACATTTATCTGCAGTCACATTATTAGAATTCTCTCCAAATGGCAGAGCATTTTTGTCATTGTCTTCTGATTCTACGATAAGGGTTACTAAACTCAACGTAGCCAAGCAGTGGAAAGAGTGGCAGATTTATTTGCTGTTGATAGGCCTGATTTTGGCTTCTGGAGTTATTTTCTACATCTTTTTTGAGCACTCCGATTCTTTCTGGAATTTCCCTTTGGGCCGAGATCAGCCTGCCCGGCCTCCTCGTGAGGCAATATATGGGATAATGCCTGATTTTGATGGAGGAGAATTTATGTAAAGCCATAAAAATTTACCAAACAGACTTAGATTTGTCCACTAGTGGATTCGCCCCGGCGAATGATGATGTGTTGTTGAGCAGCCCAAGGTGCATTACCCGCATGGCATTACTACTCTGAATTTTGACTACACTCATTTGGTCTAAGATTTTATAGATAGAAACACACAGAATGCAGACGGGATTGGGAGAATAGCTCCCATGCTTTGTGTCAATGATAGTAATAGAATATTTGATGTGCTTTCTTAAAATGTTTGCCTCTGTCGTGCATTATTTG |
| **PYRIMIDINE 2 (PYD2):**  GACACAAAAATTGAGAGCATCCCAATCATGAAGATCTACAAGAGATATTGCCAATACATGAATTAGTGACATTGCTATTACATTATTATGCAAATAAACTCTAGGAGCTTGGTAGAACCTAAATACTCAAGATCCACCAATCATCGCGACTGTATTGGGTCTTCATACTTGACTTGCATGATTGGGCTATTTGTAAAGGCAATGTATTCACACATGAATTAATTGATGATGCGGTTTGGTAGGGCAAAATATGTCATACAAGAAAAATTTGTCCCCTATGATCTTGTCATTTGTGTAATCTGATAAGTCAAACCAATCTTAAACTTATTTATAAATTGAAATTGTACAGATTGGTGCACTCTTTAACAAAAAACCCTTGAGAAACTACTTCGCATCTTGCTTGCGGGCTCTGAGAGATGAAATAAAGGCTGAATCAATTTTGTCAAGCCCATTAAATAAATAGCCAAAGGGAGGCAGAGATATGTATCTTCCTGATCCTGGGCTCACATTCAACTTATCATTTTCCCAAACAACTCTTCCTCTGCTTATTGTCATTTCAACCTTTCCCGTCATGGTCCATCCTTCATAAATATTTGTATCAATCTTTGAATGATGAGTCTTAGAACTGATAGTGAAGGTTGCATTGGGATTCAATAGAATAATATCTGCATCAGACCCAGGGATGATAGCTCCTTTTCTTGGGTAGATATTAAAAATTTGGGCACAGGCGGTACTTGTTACTCGCACGTAGTCCATAGGTGTGATTTTTCCAGAGTTCACCATAATATCCCATAAAATATGCATTCGTTCCTCGATACCATTAACGCCATTTGGGATCTTCCTGAAATCACCTTTCCCTGAAGCCTTCTGTGTGGAATTAAAAACACAATGATCCGTTCCTACGAGATCTTTCTTCTCGCGATCTCAGAGACGCTCTAATAGTAGATACCACTGTCAAAACCCCAGATCTCCTTGAGATGCAAACTCGCCTCCCAACCATGCTGATCATGACTTGATCAGCCCCACCGAGCCCGAGCTAGAGACCCTCGTGGAAATTGCTAGCCGCATTGCAATCCATGCCATACCCTTTGGCGTCCCATACGGTTGGATGATCGCTTACGTTGCTAGCTCCACGCTCCACATCTGCCTGGTGTGTCCCTCTATGGCGGTGGTACTCCCCTTTATAGCCTCTGGGAAAAGCGCCTGCCAACAAAATCACAGAAGCCAAAAGTGATGAAGCACAGAAGCCAAAAGTGATGTTGTTGACCCATATTCCCACGGATAGCACTCTAGCCCGCCTATCCCTAGCGTGTACATGACATACTCCCCTTCCTTTGAAGAGCTCACTCTTGAGCTCCAGAAAAAGAACTCAAGGGGAAGTAGGCACACTTGCAACACTTAGAGAACACCCTCTATTGGTCAAAACCAATAGTCTCAAGAACAATCCGACCAAGAAAAATAATTAACCTACTGAAATTGATGACATGAGCATCAAAACCTAGTCACTATGTGCCACATGAATCTTCTGGATAAGTCAAGTCTAACAGTGCTACTAGTAGACGAGGTAAGCAACCAG |
| **ACTIN-RELATED PROTEIN C3 (ARPC3):**  TTTTTTCCCATCAAACAAACAAAGAACATGCATTGCACTCCAATACATATTACAATGACTCCTTTCTAAAATCACAAATGGCAACAGCCTCTGACCAAGACCACATTGTAAGTGGGGTGTGCATTGAGGGCACTGCATTCATACCAAGACATCTCTTTCCCACAATCCTCAACACTTTTACCCTTCTGCATGAAAATTCACTATGACATATTTTGAAAACTTTAAAAGGATTTATTGATTGTTTTGACACACTTATTCCCATTGATTCAGCCATTATCTCATACTTACACTTGGCACTCCCACTGATTCACCAATCTTATACTTACACTTGGCACTCCCATGTTTGTAACCTTGTCAAGGTTACAAGGTTGAAGTATTGACATCCACCAGCAGAGTAAACACTTTTTCGATTTTGTAGCCCATGGACAGTCCGGAAACATTCCAGAAATGTTTCCTAAGCAATGGCCATGTTCATGAATCGCCGTTTCGAGAAAGCAACCCACCATTTATTCTGTGTACCATTTGGGTGGTAGGCTCGCTCCATAAGTCTGCCACTTGTTTCTTCCCTCAACTGCTTCAAGTAGCTTCTGAGCAATTCGCCTTCTTGTTGAGACACATGAGGTGTAAACAAGCCCCCAAGAGGAAAGCCAGGTTCTCCAGGTATTGGAAACCTTTCAAGACCCAGAGTAATGATAGCTTTTGTTCCTTCCGCTTCAGTCCTGCAATTTTCTACCCTTTTGAGGGCCATATTAACATAGAGTGTCAAGTAGATCAAGAGTCTGTCAGCAGAGCTCTTGACATCAAACTGGCTCAGCTGCTGCTCTAATGTGGCTCTTCAAACGAAGAAGAGGGTATCCCTTCTGTATTCACAAAAGAAGAGTGATAGACCTGCAACAAAATATGGAAACAAGATCGTATAATGCGAGTGAGTTTCCCCTCTCCCAACCTCATTTAATGGCTCGCATTTTTAGGGTTGCAGAAGGGTCAAGCTCATTACTGAGAGCTCCGGCATGCCATTTCTCTTCTTGCTCCGCCGCACTGCTGCTTTGTGCCGTCCACCAAAAGAAAATACCGTGTTCGCATCATCGGGCAGTAGCCCAGTTGG |
| **ACYL-COA-BINDING PROTEIN 2 (ACBP2):**  CTCGTATCAAAGCTCTCTGTCTCTCTCTCTCTGTAAAGAAAGAAACCTTACATTGCACAAATTCTCGAGACATCTTTGACGTTTAGTATGTACAACACTAATCACCTAAGAAACTAAGGTTCAATATACATTAACCAACATATTACTGGATAACGAGTGTTTATTCAATCCCATGTTGACTGTCATGTGAATAGTTCCATGTAAAAGGTGCTCATTATATGTACTTGGTTGAGCAACAAAATTACAAGGCTGCAAAAGCTTGTTACTTAAACCAGACATTTCTTCAGTAAGTTCTTCAAACTATTTGATATCTTCACCTCCAGAGTTCTCCTTACAAGGTGATAAGGAATGTTCATATGGTGTACAGCCATCATTGTCTACCAAGGATGGGTCAGCACCATTTTCCAATAGGAAAGCAGCTATTTCATCCCATTCACAAGTGATAGCATAATGTAGAGCTGTTTGTCCGTCTGAATCCTTATCATTAATCTCTGCTCCCCGAGAAATCAGTAATTTGACAATATCAAGGTGACCCTGGTCAGCTGCCCAATGTAATGCAGTCCGATTATCGCTATCTTTCTGATCAACTGAAGTATACGATTGCAGCAAAGTTTCCAAACCTTTCAAATCGCCTTCCTTAGCACATTTGTGGATACCCTCCAGACCCTCCTGCTCAATGGCATCGCTTAACATTGTGCTAAAAACAGGACCCATCCTTGGAGCCTTCTTACTGCTTCCTGTAGAAAGGCCATGAGCTAACTCTTCATCTTTATTCCAATCTGGATACAGCTCTGAAAGCAGAGTGATGTAACGTTGCATTCCCTCTTCTGGCGAAACATCTCTTAAACTTTTCCACGCATTCCATTTGGCTCGTGCAGAAAGTCGATAAGCTGAGGGTTGCGGAGAAGAGCATGGTCCCTCGGTTGCTTGTTTGTAAAGGCCATAGAATTGAAGTTTCCTATCATTTGATATTTTGGAAGAAGGATCTAGTACCAGCTTTTCAAGAAAGGCAGCTGCTTTCCCAAAAGCTTTATCCAAGTCAGAACCATCAATGCCTTCCCAATCCTCGTCGTCTGAAAGAAGGCTTTCCTCTTTGTCGGATCCTTCTTCAAGACTTGCTACATTCTTAGTGCCATCTAAGGGAGGGGCGGCATCTGCGACAAAGTCGTCCTCTGTGTGTGGCCCTTCATGTGCTACATCCGTGCCTTCACTAGATGCACCTTCTGAAGGCATAGTTCGTGTATTGTCTAGCTTATCCACATTGTGAAGGGTATGCTCACCTTTTGAGAGCGTAAACCCCAAGGTGTCTCTAATCGTTGCAGACGGCTGTGCTACATCAAGGGTTTCCACACTTGCAACCTGTGTAAACCCTGGGTCTCTCTCTTCCATTACCTCGGCAGAATCGGGGCTTTCCGCATGCTCCCTCTCAACTCGAAGGTTCTCACTTCGAAAGGAATGCACAATCGAGATGAGCTTTACAAGCATAAAGGCAAAGACAAGGGACATGAAGCACGAAGTAACCCATTCCTGCCAATCTGCCATGGCTAATGATGATCAATGCGAGCGCGACAAGGAGGGCGGTGCTCGCTGGCTTTCTGGCTACGTTGTGTTGATGATTGTGATGTTGCACATGGAGGATCGCGGCTATCATGAGTAATATAAGTGAGGAAAAA |
| **HEAVY METAL ATPASE 2 (HMA2):**  CGACGGTGTTTCTTATAGTTTGTACGTGCAACTGGTACTATGTAGTAGGTCCCTATTTAGTCTCCCGTCTCCTTGCTCTTCCTCAGAAGTGTCAACGCAGGCAGAGCTCCAATGGCGACCTTACCATCGCTCAAGTCTCATCACCACAAAGAAGGAAACCATTTGCTGCTACGCTACGGCAACAAGCTCTCATACTTCCTTGTCCCTCCTCACCAAGAGGTGGAGCTCTCTGAACTATGCTTTACCGATCACAACCGCGGCATGCAGGATCCAAGAATCGAGCAATTTTGCGCTCGAGAGGGTGGTGGCCTTCTTGCTCATCTTCACCATGGCGAAGTGATTGAAGACATTTTCTTGGAGGAGTGCATCGAGGGGGCGGGATGTGGCCTTGGCTCTTTGCCATGGGAATTTTCAGACTCATGTTTGAATGTGCTGTCGGGCGGGTACCAAGACCTTGAGCAGCACAAGGCAGGGAAAGAGGAGCATGTGCACATGGAGGGTTGTGGTCATGAGCAGATCAGACATGGGGATCATTTGGACTGGCTAGTACCTTTGAGTGATGGCTTGTTTGCGTTGAGCCATGGTCTTGGAAATGACGATGGAGGGTTGGAGCATGGCCGGCTTGTAAAGATCGGTGATTCACATGGAAAAATGAGAGAACGTGAAAAGAGCCCTTACGATTTGTTCAAGTTTGAGGTTCCGCAAACTGGTGATTGGGATACTAGAACAAGAAGTTCTCCTCAGCTAGGCTTTGAGCATTCCAGAGCTGGGAAATCTTCTCCAATAGAGCAAGTATCTGATGCCATGCAACCTTTGTTAAAAGAGCAAGAGGTTAAGATAAGCATGGGAGTAGATATGGAACCAGTGATATTGCAAAGGACTACCATTGATGTGCTTGGAATCTGTTGTCCAGCTGAAACTCCCTTGGTCAAAAAGATTCTTGAACCTGCCCCGGGAGTACAAGAAGTCTCTGTGAATGTTGCAGCTAGATTAGTAACCGTACACCATGATCCTTTCGCTACCCCCCCTGCCAGGCTAGTCAAATTGCTGAATGCCGCACATCTAGAAGCCAATCTTCACGTAACAGGGGAGTGGAAAGCAGCACGCAAATGGCCCTCTCCATACACATTCGCTTCTGGCCTCTTTGTGGCAATTGCAGTTTTCCAGTATGTGTTTGATCCTTTGAAATGGGTGGCTCTGGGGGCTGTGGCAGTGGGCACACCACCTATCATTTTGAAGAGCTTCACAGCCTTGCGACGTCGTATACTTGACATCAATTTACTGATGCTTATAGCAGTTGCTGGTTCTGTTGCTCTTGGAGACTATCTAGAAGCGGGCTTGATTGTGTTTCTCTTCACTCTCGCAGAGTGGCTAGAGTCAAGGTCCACTGACAGGGCACGACTCGCAATATCTTCTGTGGCAAGTTTGGCTCCCCAGAATGCTGTTCTTGCAGACACTGGTGTAAGAGTTCCCGTCGCAGATATCAAGGTTGGGACTAGGCTGGCTGTGAAGGCAGGAGAGTCCATTCCTATTGATGGCGTTGTGGCGTCTGGTAGGAGTGTTGTAGACGAGAGCAGTTTGACTGGAGAGTCAATGCCGGTCGAGAAGGAGGTTGGCATACGCGTGTGGGCTGGCACCATCAATATGACCGGCTACCTCTGTGTTGAAACATCTGCCCTAGCTGAAGATTCTGCTGTAGCCAGGATGATAAGGCTAGTAGAGGATGCTCAGAATCAGCGCTCTCACGTAGAGCAGTTAATGGAGAGATTTGCCAAATACTACACTCCACTTGTGATAGTTGGTGCACTAGCGTTTGCTATAATCCCAGTAAGTGTGCATGCACATAACACTCGACACTGGCTGTACTTGGCGCTGGTTCTCCTTGTGGTCGCTTGCCCTTGTGCGCTAGTGATTTCAACACCTATTACTACAACATGTGCAATAGCCCAAGCTGCTCGCACAGGCCTCCTTGTGAAGGGTGGGAAACACTTGGAAACTCTTGGAAAACTGAAAGTGATTGCGATGGATAAAACCGGAACGCTTACTGAGGGCTGTTTTCAAGTAGTACAAATGCATTCCTTGGGCAAGGATGCCAATCTACAGAAAGTTCTATTTTGGTTATCATGTATAGAGAGCAAAGCAAGCCATCCAATAGCTTCAGCTGTTGTGGAATATGCAAGGCTTCATGGTGCAGAATCACCCAATGCTAACACGCTTGTGGATGACTTTAGAATACTCGTTGGAGAAGGAGTCAGTGGTATTGTTGATGGTCATGAAATTTCTGTTGGGAATGAACGCCTAGCAAATCGGCTGCATTGGATGGAAGCTGCGGCTGCTGAATCTTTTATTTTGGAGAGTTGGAAAAGCCAGGGGTTGACAATCTGCTGGGTTGGTGTGGATGGAAAGCTTGTGTTGATTTTCAGCGCCGGTGACCAGCTTCGTACAGAAGCACGGGAGGCTGTTAAAGACATGAGGGACTTGGGTCTTCAAGTAGCTATGCTTACTGGAGATAGTCTGGCAGTAGCCAGCACTGTACAAAGAAAGCTTGGACAAATTGATGTTCATGCTCAATTATTTCCCGAGGACAAAGTGGAGCTCCTCAAGCAATTGAAAACAGTTGGCCTCACTGGCATGGTTGGTGATGGGATCAATGATGCTCCCGCTTTAGCGGCTGCTGATGTTGGCATAGCTATGGGTGTTGCCGGAACTGCGATTGCTATGGAGACTGCTGACATTGCCCTCATGACTAATGATCTCAGAAAGCTGGCAACTGCTGTAAAACTTGGGCGAAAAGCTCGCAGAAAGATTCAGCAAAATATCTTCTTATCCATCATAACCAAAATACTGGTGATTGTGCTTGCAGCAGTAGGGTATGCATCTTTGTGGGGTGCTGTTCTTGCAGATGTTGGCACTTGTCTACTTGTCATCTTCAACAGCATGTTATTACTAGAAAGAAAGAAAGATGGATCAGGTTGTTTGGGATTATTTACATTTGGGCGCAAGCCCAGAAGTAAAGTGTGCCAAAAAGATGTCTTACTAGTTAGTGAAAAGGATGCCAAAACGGAAGCGGAACCCTGTTGTCTAAAGGTATCAAGCCCTTCAGATGGTTCGAACACTTGCTGCAAGAAAAGGGAGGGTGATGGGAGTTCTGTTTGCAATAGCCATCAGGAAAACCAATCAATCAAGTCCTGCTGTGCAGCAAAGTCGTGCTGTGATGCAAAGAACGCAAACAGTGGAATGAGACGTAGAGTGAGCAATCGAGACCCTTGCGATAAGGATTCACAAGAAATTGCTGAGGGACCGTGCACTGCCATTAGTATTGCTGATCTTGAAGGATTATGCTTGTCATCTCAGATTGTGAACAACAGCAAAAAGAATTCAAGCGGGTCCTCTTCAAAGGAGAATGCAAGTTGTTGCTCTAGTGGTAAATGTAATGGTTCCAAAAATGAGCAATTCAGCAGCAACGTAATACAGTCAGGAGATGATGGATCTTGGGGCACCGCCATTAAAAATGACATTGAAAACAATTCTCACAGCTCTACGCGAGTCGTGAATTCATGTAGCTCTAGAACCAGGATTGACCAGTGCTGCACGAATGATACTTTGCTGCTGGCTGCTATGCCTGCATGTTGCAGCTCAAAATGCCAGGCCCCTCCAAGCCTAGAAGCTCAATCCACTAAGGGCTGTGAACAAGGGTGCTGTGAAGGGAAAAGTCGCAAGGCAGAGGAGTCAGCAGCAGTACAGCCAAGACCAGTGTTGCAAGAGAAAGTTGAAGGAGTAGCTTCTGAGGGAAATGGTAAGATCGGCTTGAAAGACAATTGCTGCATGTCTAGGCAAGTTGTGGGTTCATGTGGCTCTAGGAACAGTGCTGACCAGTGCTGCACGAATGATTCTCTAGTCGCTACATGTTGCAGCTTGGAAAGCCAAGCCCTTTCAAGCTTAGGGATTAGATCCGCAAAGAGTTGTGAAGAAAGATGCTGTGTAGAGAGGAACTGCAAGGCAGAGGATTCAGCAGCAGATCAAGGGCCAAGGCCAGATTGCCAAGAGCAACCTAGGCAAGATGTGAATTTGTGTAGTTCTAGGACAAGTGTTGGGCAGTGCTGCATTCATGATACTTTAGTGGCCACCACCCCTGCATGCTGCAGCTCGGAAAGACAAGCCATTCCCATCTTGGAGTGTAGATCTAATAAGGGTTGTGACCAAGGATGCTGTGAAGGGAGAAGTCAGGAGCCAGCAGCCTGGAAGCCAAAACCAAACTGCCAAGAGAAAGCCGCAGGTGCATCTCTTGTGGGTGGGGACAGGAATTGTAAGTGCGACATGAAAGAACACCCTTGCATCTCTAGGCAAGATGTGAATTTGTTTAGCCCTAGGACAAGCATTGACCAATGCTGCATGAGAGACACTCTGATTGCTGCTTGTTGCAGCTCCAAATGCCAAGCTCCTCCAAGCTTAGAAATTCAATCTACTAAGGATTGTTGTGCAGGATGCTGTGAAGAGAGAATCTGCGAAGCAGTGAAGGAAGCAGCAGAGGAGCCAAGGTCAGAGTGCCATGGAAAAGATGGTGCAGCTTCTCTGGGTGTGGAGGGAAATGAGAAGCAGTGCTGTCAAGCCGGGCCCACTTCGTATGATTGTGTGGGAGGCAATAAATGCTGTGCAACTGCTTGGAAAGATAAAGATGCATGCTGTGGTGGTGGTTCCACTAGTAGTCATGGTAGTAAAGATGTGGGCTGTGCTAGTAATAATGATACCTGTCGTGGTGATAGCTGTGAAAAGGGTGAGGCAGCAACCGATGATAGTGCTTGTGTTGGCGGTGGTTGTTGTCGTAGTAGTAACATAAGACATGAGCTAGAAGCTAAAGATGGAAGCTATGATAGTTCATGCTTTGAGGGTAACACTTCTAAAGAAGAGATTGATTGTTGTCGGAGTGGTGGATGCTGTGGCTCAATTAGCCAAGACATAAAGGCTGGGTCCTGTGGAGCTGTTGCCATGCCATCGATGGAAAAGCCAGGCCAAGCTGAAGGACCTGCCATGTCATCAATCGAAAAGCCAGGCCAAGCTAGAGGATGCCAAGGTAAAGATTGTTGCGCTGTCACTCAGTAAACTCACGAAAAGAGTCTCAGCTGAGGTCTGTAGAAATCTACTTGTCTGCCTACGCTGAAACAGAGTAATTCCAGAGTAATCAATTTCCCCAGTGCTTTATGTCATTTTATATCCCCATTGCCGTCTTACAAAGCTGATGAGAAACAATATGGTCAGGGAGCTGGGCAAGCAGAAGGCATTGAACCTTGGGTCCAACACCATAACATGTGACTTCGCTACTTCCTGGATCTCTTGTCTATCAATAGGACTTTGCTGTGGTAGACCTTTTTCTGCACTATGATGTCTTGTATTGTAGTAGAAAACTTCTCTCCTTCATACCGGTAAGCTTGTTATCGGCCTCGCGCATACCTTTCTTTGTACCATCCACTATCTTCTTGTGTAAAAATATTGGCCTCCATGATCCTTGTAGAGATAGTAATTATTAAATGTTTGTCCAAGTGGTATGACCTAGACCAATCTAAAGTCCCACCAATCACCTATATCTTGGCCCCCCACACATTTCCCATGTCAACTTGCATTTGGGGGCTTGACATTTGCTGCCCCTGTAGGGCAGGATTGCGTGTGCATCAATACTCCTAGCAATAGCCGACTACGTGTGCCAGCTCAAACACGAGAAGTTTTAGATAATTTCCCACCGTAATTCCCACTTTGCCGATTACGA |
| **PLANT CADMIUM RESISTANCE 2 (PCR2):**  GTAAAGGTGGTCTAAAATTTGACAAATAGACTATTAAACAAGCATAACACTACATTACACATAATGACACAAATCATGGCACTGCATAACGTTAGCATGCAGGAGCATTATATGGTACATCACTTTATACTAAGGGGCCAATGATAAATCCATATTGAATGATTGCAAATTATTTTCTCAAACAACACCTAGGATTAATATTACTAAAATAAGAGGGAAAAGTTTGGGATAAAAAAAATTTGAGACAACATTCTTCGAATTCCTTATTTTTGCATGACATGAGGTTGAGGAGCAACATGTGCATATTTCTCAGAGTTAGCGGCCCATCCAAGGCAAGGATCAATGCCCCTAGCTTTGAGCTCACGATATTCTTGGCAGATAGCACATGCATCACAGAAGCAATGCACCACAAAGTCATGGCATGGTGTTGCTGGAAGGCCAAACTTTTGTCGAAGCTTGGTCCTATAATGGGTAGTGTACATACAAGGGCACCCTACACATGTTGCCCCCAAGTAGAATATAGCATTCATAGGACATGATTTGGTGCCCGTATCCAAAATCTCTCCAATTTGCCCAAACGTCACGCACGGCAACCACCATGTAAAACAACAGTTTTTGAAGTCCTTGAAGCAGTCGAAGAGGCCCGAATGCCAGCCTTGTTGGGAGGATGAGCTCGCCATGGCCTCTTTTAGAGAGAGAGAGGGAGTCTCAATAGGTGGTAGTTA |
| **HYPERSENSITIVE TO EXCESS BORON 2 (HEB2):**  GTTGGCTATGAAGTCCAATGCTTGCAACACCAGGCCATAGAGTTATTCTACCTTCCGGCTATATATTTGGATGGAGCCCTGTGTTAAGAGGGCAACATTTTTTCAAAAATTGAGTGATTCCCCCAGAGCCATACACACACACCCAAGGTGCCAAGATTCGACCTTGGCCCATAAATTTCGTGAAAATCATTTGAAAGCTGATCTGAATCACTCAAAGCCCCCTTGTTTGCCAGATTTTCCATGTTTAAGATATTGATAGTGGATATGCTGCTTCACAAAATTATCTGCAGCCAAATGTAGCATATTCAGCATGCAGGAGGTCTTATTGCTGGTGTAATGCAGGAAGATTCTTTCTGCTCATCCACGATGTCAATGTTCATTTCCTCAGGAACATCGTCGAGATTTAGAAAATCTTCCTCTTGATCTTCAATGATGTCTGAATCTTTCCCATTGGCTTGTATGGAGCTGTTTTCCCGTTGGTCTTGTTTTTTATTGGCTATGAAGTCCAATGCCTGCAATACCAGGCCATAAAGGTATTCTACCTTCCGGCTATAGATTTGGATGGAGCCCTGTATTAAGAGGGCAGCTTCTGCAAAATTGAAGGAATCATGGCCGTCTTCATCCGGAAGCGACACCAAAGAGAGCCCTGCTAGGTAGCTTTCAAGCTCCTGCGCCACATCCACAGCCCAATTTGCTGCCAGGTCCCGATTAGGTTGCAGCAGATGCAAATATCGAGGCTCCTCTTCCTTCTCCATATATCCTTTCAATTTGCACTGAATCTCAAGCTCTGATGGTGCACTGAGCTTTCCGTATCCAAAACGAGCAAGGTGGGCATGCTTCTAAAAGACTCTTCATAATCCTTGATCATTCCTGTTACAATTTTCTGCAAAACCCTAGTGTTTCAACCCAGAACCCTGCTTTGTCCTCTCCAAAAAGCCCTTCAGATATCTGCAAATCCCTAGCATGCTCTATAACTTAAAGCTGCAGGGAAATCCTTCTCGTCCGGGATCTCCCTAAACCCTAATTTATCAGCTAAAATTTACAGGGTAAAAGAAACCCCTAATCCCTTGTTGTAGCAGAAACTTGCCGCAAGAACTTGAGAAACTACACAATGCGCTTCTGGTTCCTCCTCTCTGCGTCTGCTTGAAGTAAAATTCAAACAGCTACCTAGTTCCCGCTCGCATCAACGACACAAAACTCTTTGGGTGCTTTCATACTTG |
| **ELONGATOR PROTEIN 6 (ELP6):**  CTACATACTTGCATAAAGGCATCATAAAAACCCCAACCAAGACAGAAAAACATTAAGGGCAAAACTCTATGACCAAACTTCTCTAATGGAAATCTTAATTTCTTGCAAATTCCCATGTTTGGTTGCTGTATACCCACTAAAATGAGTTGAGCACTTGCCCTGCCCACGGCCCGAATAAGGGGCGTAAACCAAGTTTGTTGGAGGGTCAGCTTTAAAGGTGCAAGAGGATGTTCAAGTACAAAAATTTACTTCCTGCTTTCAGATTTGCTTTCCAGGAGCAAAATAAAGGACTGTGTTCTCCATCAGCTTGTAGTGAAGACCATAGCCATTCAAGTCTTGACGCACATTTGAGTGACCACTGGAGTCTGTATTGCAAGGCATCCTATGCTCAACAATCAGCTGCCCATGAACATCATTGGCTTGCCCTGTTGCGAGCGGTTCAACATTGATGACTGTGTCTGCCAAGTGCTCAAGCTGGTAAGCCATCACAGTCTCATTGGCAGTTTCATAAACATCTCGATGAGTAAGAAGTAGCAAAGAACAAACCTCCTCATTGGAAGCAAGTGTCCTGCAATAGTGGAGAAAATCTCGAATATGCGTACTATTGCCTTCAGCCACCACTTCTAGCAATGATATGTCATCAATTATTATCCACACATTCTTTCCTGAAGTCTGCTTCAAAATCTTCACAAAACTCTCGAAGAGCGGAAACAGTTTGTTTTCAACACTTGCAGCAGCCACATTTGGGGACAAGCTGGATAGCTCAAGGATGGATTTTGACAGCAAATCAATGTAGACAAGAGAGCCACGCTCTCGATGCACCACCAAATTGCATCCCTGTTTTCGGGCAATTCTGGTGTAGTGAGAAAAAGGCTCCTTAAGGGCAACGAAGATGACAAGGGAGTCGGGAACAGGCAAGATGTAGGAGAGGAAGGAGAGGAGGAGGAAGGAGGCAGGAGAAGTGAGCGAGTCGTTGATCAACACCACCTTGTTTCGTGGGGGGGCTCCTCCACTCCACCCCAATGCCTCGTGCAGCATGGACATGCTCTAAGGCCCCATTCAACGCTGATTGGTTGAGAGAGCACGTTGTTTTGTTAACTTTGAGTTGGGCGTGCTTGGAAGCCGGAAGGTTTTTTCTCCAAAAGCTGTCGTGACTCAGCAGCCAGATCAGCTTGGATGCTGCCTTGTGTGAACTGGCAAGAAACAGGCAGCTCGGTTGATTGGTCCACGTCAGCCAGATCAGCTTGGCTACCGCCTTGTGTGAACAGCCAATAATCAGGCGTCTCCCTTGATTGGTCCACATCATCATTTTCACCTTGGCTGCCGCCCTGTGTGAACCAGGCCTTATATTGAATGTCACTATTGAAAGGACGACCTTTTTTAGGTTGGATGTATGCATTGTGTAGGATTTCAATTTGTTTATATGTGGTTTTTGCTTTTTTTCTTTGTTGCGAGGGGGGGCAAATAAAAACACAGCTGCTAAAACACGTCACTCCATTACCGCTCCATTAAAAAGTGCATTACAGGAAGTAGTAAATAGGAATAGGTGTGTGGAGACTTTACATTCACTCTGCTTGTATTGTCACTTTGAGAGTTTAAAAGATTATGTTGGTTGCCTCAGCCTTGTCATGAATATCCCCTATCAGATGGAGGGGTAACCCATTGAGTGATGCACTCCATCAAAAAGGCAGGTTATCAATTTTGTGGATTATGGATTATGGGGTGACAAAGTTTGTACACAGTTTGGTGCGTCTTGTACAAATTGTAGACTATAATTATGCAAATCAGCCTGTGTATAGCTTTATCCCTATGGGAGGGAATGACCGTTTTTTCTTAGACCCCCAGTTTTCCAGTTTTTTATGCAGCAAATTAGGAAAAGCCCTTAAAAGTTTACTTAAATCTCAAAAATAGCCTTGAAAAAATACACTAATGGTCGGTTTTTCATGAGCACTAAGTATTTTTAGTGGTCACACTATTATTTCATGGTCACAATATCATTTCAGAAAATGACCGTTGCTGCTAAATTTACATGCATGAGGGAAAAATCCGTAGGCAGCCAACATGTGGAAAGGCCAAAACTGCTATTAGATTTCAATATGTTATTGCCACTGGAAGGACCATTATACTGGGGTAAATTCCAGCATTAAGAGTATTTCAAATTCAAGGAGGTTGTCTTGTTAAAAGCACCAAGCTCGGAAATTGTGTGTGTGACTGGAGTATAGTACTAGCGGCACTCAGCTCCTACGTGATGCTTCTAATCAATCCTATTCTTGTCTATAAATACCGAGAGAGTATTTTGTG |
| **NUCLEOSIDE DIPHOSPHATE KINASE 1 (NDPK1):**  TCCAAAAAGCTTATCATACCTCATTTCACACAATAGCACCATTAAACTACAGAACCAGGTTCTTCGGAAAATATGAATATTTATAGCATTTAGAGCCTAAGACAGAGATGCATGGGCGGCACTGGATGATCTATGAATGCCATCAACATTCAGTCAATAACTAGACCCAAAACTCATACACCGAGTGCACAAGAGAAAAGAGAATCACTGGAACAACCTGGGCAGCTAATGATCAAACCCACATTGCATGATTAGGGTGATACAGAAGCATCGTTGTCATGCCCTAACTAATCGCAGATGGAAATACGAATACTATCTGCTCATTGTCACACATCATGCATCCTCTTAACAACAACTCTGTAGGAATACCTTATGCCATGCTGCTGCAGTTTTCAATAGTTAAATTCCCAAAACAGTGAAACACTCTGCTCCATTTTTGGACAAATCTAATCCTTGAGCAGTCTTTACACATGAGTTTGACATAACCCCTGTTGAAATCAGAGACTTCCTTTTGTTGCATCTCTATCCAGATGTATATGGCAAGTTTCTATGCACGCAGGCATGAGACAAGTGCTTTCATTGCGCCTCTATCCAGATGTCTATGGCAAGTTTCTGTGCGTGCAGTCACGAAGCTAGTAGGCCAATACAAGAAAATTAAACAACTCACTTTCTCGACACCTCCAGGCATGTACACGCCATAAAATATCAGTGGCCAACTTATAGTAAACCTCAGACAACAATCACTATGCGGTGCAATGACATCTTCGCTTATTCTCAAGCTCTTCAACTTGATAAGATCAAATATCATGGTGCCTTCTGAGCATCAAAATTGTGCATCTCAACGTATGGGACACTGATAGCACTGAAGGCACCAAGATTTCCACAAAAAATCCAAAAATGGAGCTCATATGATAACATTTTTTTAAAGAAATCTCACTAAAAGGTTACGTTATTCCCGTAGCCATGGAGTCAACGTTGGCTCCCAATCACATAATTCACTCTCTTTGAACCACAAACCAATTTCACGCTCCCCATTGTCGGGGCTGTCACTTCCATGAATAACATTCCTTCCAGTTTCAATACCCAAATCCCCACGGATTGTTCCCGGCTCAGCTTGTAAGGGATTTGTAGAACCTATGAGTTTCCTAGCAGAAGCCACCACACCTGGTCCTTCCAATGCCAGACACACAACGGGTCCTGATGTGATGTACTTCACTAGCTTTGGAAAAAAAGGTTTAGAGCTCAGGTCTTTATAGTGTTCCTCTGCAAGCTCCGTTGAACAACTGAAAAGCTTGAGTCCTTTTATGACAAAACCTTTTCTTTCGAATCTTGAGATAATCTCTCCCACCAGGCCACGTTGTACTCCATCTGGCTTTATCATGACATATGACCTTTCGGTTGAACCCTGAGATGCGCGGACAATTGGGGAGAGGCGCAAGCAATGGTGGTGGAGAGAGGCAGAATGGGAGAAGCGAAGCTGCGAGCGGAAGAGGCTGTGGGAGCTGGAGAGGGTGACACGAGCAGATCGGGCGAGGCTGGAGCCAAATGGCTGGAGGCCTGAAGAAGATGATGCTGAAGAAGACGTTGAAGAGGAGGAGAGGAGGGCAACAGAGCCTGCCATAGCCACAGAAGTGCAGGCCATGCCTCCTGCCCCTCGACTATTTGCCAGCGAACTCCCTGGGAGGCGGTGCAAGGAGAGCCAGGGCAAGTGTGGTGCCTTGTCAGCAAGCTAATTGGGGGCAAAGGCACCTG |
| **GLYOXYLATE REDUCTASE 2 (GLYR2):**  AAGGGCGAGAGCAACGAGGAGCGAGCCACCTCCACACCTCTGTACGATGGCGTCCCTCCATGTGAAGCTTCCGCTCCTCGTTGCTCTCGCCCTTTGCTGCTCTCATTTGATAGCCTCTCCCACTCTGGCTGGCTACTACGGCGCCGATAGCGCCGGATGGAGCAACGCCCACGCCACTTTCTACGGCGGCAGCGATGCTTCTGGCACCATGGGTGGTGCCTGTGGCTACGGCGACCTCTACTCATCGGGGTATGGCACGAACACGGCAGCGCTGAGCACGGCGCTGTTTAACGCAGGGCTGAGCTGCGGAGCATGCTTTGAGCTGAGGTGCGACGAAGCAGCAGATGGCCAGTGGTGCGTGAAGGGGACATCCGTGATAATAACGGCAACGAATTTCTGTCCGCCAAACAACGCGCAGCCAAGCGATGATGGGGGGTGGTGCAATCCGCCGCGAGAGCATTTCGACATGGCGCAGCCGGCGTTCGAGAAGATAGGGGTGTACAGAGGGGGGATAGTGCCGGTGCAGTATAGGAGAGTGGCGTGCGAGAAGAAGGGGGGAGTGCGGTTTACAATGAACGGGCACGCGTATTTCAATTTGTTGCTGGTGACGAATGTGGGCGGCGCAGGGGACGTGACGGCGGTGTCAGTGAAGGGAGCGGGGACGGGGTGGCAGCAGCTCAGCCGCAATTGGGGCCAAAATTGGCAGAGCAACGCCCATCTGACAGGCCAAAGCCTGTCGTTTCAGATCACAACCAGTGATGGCCGCCTTCTCACCTCCTACAACGTCGCCCCCGCCGACTGGCGCTACGGCCAAACCTTCAGTGGCCTCCAATTCTAGCTGCTCTCCTCCACGTTTCCGCCCTCATTACTCACATACAGAAGTGGCTGCCTGTAGCTCAACCCTAATTCTACTTCAGCCCGCTGCACCTTAATCTCCTTGGCCATATCTATATATATTTTTTATATTTCTCTATTGCTGGATGTAATCTGTAGTGTAAGTTGAAAACAAGTTAAAATGTTTAAATTGCCTTCCTTTTACAACCATTAACCTTAACCAACTGCGCTACTGCCCGTTGATAATTGCTTGCATTCATTGAGTTAAGAGCACGTCTCTTTTGTAAGAGTAACTACCACGGCACAAGCCAATTCCACATAGGCAGATATTTAGAAACAATTATCCCTCTTGGTGTTCTAAATATGGTCATTGTAGCATCAGAACAAAAGGGCTACTCCAGCAAGACCTTCAAAAACCTCAAATAATTGTGTGGCGGTGTGGGTGTGTATAAGTGGACACCCTCAAGGCAAAGAATGCAGCCAGTTCAACAGCAAAGGTGCAGTTTGTGCCTTTACAATCAAGCTATACCCCATTGCATGACTAGGTTTGTTTGATATCATTTTAGTGTAATTGATCACTTCTGCCAAAAGAAGCTTTTAAAGCAAGCTCAAGAAACTGAGCAAAAATGTCAATCCACTAACACTTGTTACTAAAAGCAGCTTTAAAAGAAAACATTTTTGTGTAGGTGGTAGCCGCATTGCAACCTGTTGCGAAACTAATCTTCTTTGATTGCTTCAATTACCGCAGAGAAGTCCTGATCGCCAAAGCCTTTAGACTTGGCCACTTTGTACATTTCATTTACAGATGCTGCAACCGGAACTGGTTGGGACACAGACTCAGCCAGTTGCAATGCGAGTCGTAAATCCTTTTGTTGGTGTTTAAGAGGAAATGACGGGGAGAAGCTCTTCTTGATCATTGAGGGACCCTTCATTGCAAACATTGGCGAGCTTATGGCACCTTGAGATATCACCTCAATTATAGTGCTTGGATCCAATCCAATCTTGTCACCCAAAACAAGACCCTCTGAAAAAGATGCCATCATGCTTCCCATGACCATGTTGACAATCAACTTCATGGCTGCACCATTCCCAACTTCACCAAGGTAAAACTTTGACTTGCCCATTACATCCAGCAGAGGCCCAGCTTGTTCATACAAAACCTTATCTCCGGCAGTAAGGAATATAAGAGTTCCATTTTCAGCAGGTTTCTTCGATCCTGAAACCGGTGCCTCGAGAAATGACGCGCCGGTAGCTTTTACAAGGCCACAAATCACTTTTGAAGTTCCACCATCTACTGTTGACACATCCACGTACCCTTTACCAGGGCCCAAGCCATGAATGACACCATGCTTTCCTGAAGCTACTTCCAACGCAGCAACAGGATCGGCGAGCATGGCAAATGTGATATTGCACTGTGAGGCAACCTCTGCCGGAGAACTACCACAACTGGCTCCTTGGTTGATCAAAGGCTCGCATTTTTTTGCATTCCTATTCCATACTGATACATCATACCCGGCCTTGATGAGATTGGTTGCCATGGCAGTGCCCATAATGCCAAGGCCAAGGAATCCAACGGTGGGTTTTGCAGTATCATCAGAAGAGGGGGAGGAGCTGCAACAGCGCGCCGATGAGGGCTTGCGTGCCAGGGAGAAGAAGGGCGGGCACAATAGATGACGGCGAGAGGGTGCGAAGAAGAGTGAGCTCGAACACGCACACGCTATCTGGTTGAGCTGAGCCATCGCTCGTTTTG |
| **GLUTAMATE RECEPTOR 3.4 (GLR3.4):**  TCTCTCTCTCTCTCTCTAAGCTCTTTTGTTCACAAAAATCTATGGAGTTTGATTGTCTATTCTCACTCCCATCATTTTGATTCTGCTGGCCTTAGTAGTAATTTTATTCAGAGTTATCTTTTTTTTTTTGGCATCTTTGAATTTCCTTGTTTCAATACTTTACACTTGCACTCCTACATCCATCAGCTTCTTAAGTTCTCTAAATTTCTGCAATTCGGCATGTTTCGTGCTACCGGGCAAGCCCACATCGGCTTTTTGATCTTAATAATGAATTATGCTTTAGCTCTCTATAGAAATTTTTAATTCAATCCAATTTTCATTAATTAATCTTCAATTTTGGCAGCTTCTTGTCATAAACCATGATTCAAGCTTCTTTGCATAAGCCCAGGATTCAAGCTTCTTAAGCCTTTGTCCTTCTGTAAGGGCCGGATTTCAAATTGTGGGTTCAAATCCCTATGTTATTCACTTGGTTTCTTTCACAAAAATTGTGTGTTTTGCAGCTTTTGATGCTCTGGTTATACACCTTACTCTGTTAGGAGTAAGTCATGCAATTTTTTCAGATCATTCTGATTTTGCACGGGTTAAGCCGCAATTTGTACTCCCCACGCATGACTGCGCATTGTGCGCATTTGCAATAGGTCATTACATTACTCCAATCATGCTTCAAATTCTCCACTTATATAGCCACTTTTGTATATAGATAGCAGGGTCTATAGGCTATGATGCAATATGTTAAACACTCTGTTAATGATGAGTGCCATGCCCCTTCTACATTTAGCAGCCAGAGTTCTTTTTTTCCTATTCAGTTGATTTTCATTCTACAGCATGCTTTCTTCAAGATGACAAAATGAATCAAGATTGATGTCGCCGTGCGCACTCTCGTTTGCATATGGTTCACATCGAGAACATTGAGAGTATTCCTGTGGGCAAAGCCATCATTGGCATGTAGTAGTAGCCTGCATGATCCACTTAGAGCTGCCAAGCTAATAGCTCAATTTCCTTAGACTAGATTACAGCTTAGTTTTATTCACAACTTTTCACGAAAACTTCGTTTGCGACATCGCTACTTTGCGAGACAATCAAGCAGTCAAGAATGCAATATCCTTTCTCTTTGTGTGTATTGAGTTGTATTCTGCTTACACTCACATGGTGTTGTATGAATGCTTTGGGTGCTTCCAACATTACAAATGGGCCTCCTGCAAGTGTGAAGATAGGGGCTCTGCTTGCTTTCAACTCGACAATTGAACAGTTTGCACGGCGGGCTATACTGCTCGCAGTCCAGGATGTGAACAAAGCAGAAGATGTTCTAAATGGAACACAGCTTATCTTGGAGATGTTTGATAGCGGCTACAACAATCCAATACAAGGAGCTGCTTCTGCTGTGGAGCTTGTGAAGAAAGGCGTTGTGGCTGTTGTAGGTCCTGAATTTACAGCAGTTTCGCAGTTTGCAGCTCATATAGGACAAGAAACAACTATCCCCTTTGTCTCATTTGGTGCAACAGATCCAAACCTCTCAGAGATGCAATACCCTTATTTCATGCGTGTTGCACCTAGTGATGTGCTACAGATGGAAGCAGTTGCAGCCTTCATTGCCAATTATGGATGGAGAGAAGTGGTGGTGTTTCACATGGATGATGACTACGGTACAAATGGAGCCTCTGCCTTAAGCGATTCACTGCAAGTTAGAGGTAGCAAAATTGTGGACAAAGTAGCTTTTGTTCCAGGGATAGATAAGCCTGGAATTGACAAGGAGCTTGCCCGGTTAGCTAATAAGCAGGCACGAATATTTGTCGTGCATACTCATCAAGATGTGGGTTTTCAGATCCTCCTAGAAGCGTACTATTTAAACATGATGGTTTCTGGATATGTATGGATTGTGAATGATTTGATCACCACCCATCTAGGAGATCTGCACCTTGATGTGCGAATACCGCGATATACCAAGGGGGTCATTGGAGTACGGCGGCATGTAATGGACTCTCCACAGTTGGATAGCTTTTTGGTGGAATGGAGGAGGACTTATCCTAATCAGTCACTTGATTTGGAGGCAAGCCAGATCAATGCCTACAGATTATATGCTTATGATGCTGTCTGGGTAGTTGCTCGAGCCATTTCATCTTATTTAAATGACAAACATGAGATTAGCTTCCAGAACCCCCCTAAACTGCCCATTTATTCAGGGGGCGAGTCTGAACTAGCTCAAATGAGGAGATTTGTAGGAGGGGCAGTAATGAGGAATTATGTCTTGACTACAAAGTTTTTGGGTGCGTCTGGACTCGTAAAGTTTGATAAAAAAGGAGACCTTGTAGGTGCCGCTTTTGAGTACATAAACATGGTGAGTAGAAGCCCCCATGTGGTGGGATACTGGTGGGCAAACAACTCTGGTGTCTCTTTAAATCCCCCTCCCAATCATGAGATTTCAAGTCTGAGTAGTGACACTTCTGATGATGAATTTTTCTTATCTCATGATGGCAAACCAGCAAATGCTACCAGAATGGCTATCATCTGGCCAGGCATGGCCACCACGGCACCACGGGGTTGGGTGCTCCCTAAGAATGGCAATCCTCTTAAAATTGGTGTCCCTCGAAAAGCAGGTTACAACGAGCTGGTTAGCATTACTATTGGTGCTGACAATGTGACTGCGTATAGCGGGTTCTGCATACAAGTGTTTGAAGCTGCCCTCAAGAACCTTCGGTACGCTGTGCCTTACAACTATGAAATGCTTGGTGATGGGATTACAACACCAGAATACAACAAGTTGATTCTGAAGCTAGCTGACCAGGAATATGATGCTGTAGTTGGAGATGTAGCAGTCCTCGCGGATCGTCTTAGAATAGTTGATTTTACTCAACCATTCATAGAGTCTGGGCTAGTGGTGCTGGTACCTGTCAAAAACAACAGGGAAAGTAGTCCTTGGGCTTTTCTCAGACCATTTACACTATACTTGTGGTTGACTGTGCTGTTATCATTTGTATTTACTGGCGCAGTCATTTGGATACTTGAGCACAAAGTAAATGAAGATTTTCGAGGTCCTCCAAGGGCACAATTAGTTACTATGCTGACGTTCATCTTCTCCACCTTGTTCTTTGCACATAGGGAAGACACTCGAAGCGTTTTAGGTCGATTTGTTTTGATTGTCTGGCTATTCGTGATCCTAATCATCAATTCCAGCTACACAGCAAACCTTACCTCTATTTTAACAATTGAACAGCTAGCTCCAACCATTCAAGGGCTTGATAGCTTAATCCAAACAAATCTGCCCATAGGGTATCAAACTGGATCATTCGTTCGAGATTACTTAATAGGTCTCCATGTGAATCCAGGAAGACTGAAGGAGCTAAGTTCTCGTGAAATGTATCAGAAAGCATTGGAGGCAGGGCCTTATAATGGGGGTGTTGCGGCAATCGTGGATGAGCTTCCCTATGTGCAGCTGTTTCAAGAGTCTGATTGCAAACAATATATCATTGCTGGCCAGGTGTTCACAAAAAGCGGATGGGGATTTGCGTTTCCACGGGGATCCGATATAACAGCGGACTTGTCGCAAGCCATACTGGAGATGTCACAGTCGGGGGAGCTGCAGCACATAAGGGATTATTGGTTCCGGGATGTAGTATGCGAGGAGGTAGCGAGGAATGCCGCAACGGGATCAGGCAAGCTTGATTTGGGAAGCTTCTGGGGTCTCTTTCTTATATCAGGGGTGGCATCAACAATATGTGTGTGTGTACATTTGCTGGTTTTATTGAAGAAGTACAAGGAGCATAATAGGCTACAACGGAAGGAGGAGAGCATCCTCGTGAAGAATTCAGGTAGATGGGAGCATGTGAGACAATTCATGTCATTTGCAGACAAATCTTCTCATGGGTGCAAGCATGTGAGCAAGTATGATGCCTCCTTCAAAAACCATGCAGCTAGTAGCTCTACATCTTCTTCCACTTGCATGACTAACTCTTGCAGTTAACTATATATATATATATCAAGTTGTGCATTCAAAA |
| **PPHB SUSCEPTIBLE 2 (PBS2):**  CAACATTATATCCAACATTACAAAGCCCGATTCGAACTCACACCATGAGGCTATGATTCCTTTTGGTATCTGTGTGTACTCTAGGTTAGTACGTGCGAGGAGATGACAGAGCGATGCCAGAGAATCGGTTGCAACGCCAGTTTCTCTGCAGATAACAACGCCGACGATAGCTGCCGCTATCACCCCGGGCCTCCTTTGTTTCATGATGGTGGAAAGGAATGGAAATGCTGCAAGCAACGGAGCCATGACTTTTCTCTGTTCATGGATATTCCTGGATGCACAACTGGCAGGCATACTTCTGAAAAACCTCAAACAACATTTACCTCTTCAGCTAAGAAACCTGTCATGGCACCGGTTGGGCACAGAAGTTTTACCACCGTAAGCAGGGAAGCTTGTCCTAGGTGTAGACAGGGGTTCTTCTGCTCTGACCATGGTGCACAACCAGCAGTGAATACGTCTGTGCGAACCCAAGCATCAGCAACAATTGTTGACCCAGTTGTAGAGAGTAGCGAAAATTTACAAGAAAAAGTTAATGAAGAACCGAGAGTGGTGGACTTCAGTGTAGAGCAAACTTGTAAGAACTGCAAGAAAACTTTCACAGAGAAGGAAAACTACGACACAGCATGTGATTATCACCCAGGGCCTCCTGTTTTCCATGACCGATCTCGGGGTTGGGCATGCTGCAACGTTCATGTAAAGGAATTCGAAGAGTTTTTGGAAATTCCTCCCTGCAAAAAAGGATGGCACCGTGGAAACTTGTGAAGCATATTGAGCCTTCCAAGATTCTTCTCTTTTTTACAATGAATTGACTGTACACCATAAAGACAACTGCTTAGCATTAGGAACTTTTTCAAGGTCTTTCTTGCTTTGGAGTTTCCACATGCCTACCAATGATCATCATTGTAAGAACAATTTTGAAGCTGTTGGGCATGATATAGGAGAGCTTAGTTGGAGCTTGTCTCTGTTTTTCTTTTGTGACTATGTCGAAACCTACGGAATTTCTCTCAAGCT |
| **GLUTAMATE-CYSTEINE LIGASE (GSH1):**  GAGGTTAAATGTACACATTTAAGGGAATTTACCAAGAAAGGATTCCAAGGAACAAACCTTTATTTTTATTTGATTGTGAAATTTTTATTTTATCCCAAAAGCCTTGTAGTCCTCTGAACAAAGATTGGTCTACAAGCCTTGGGGTCAATATGTGATCTAGAACCTTAGTAGTTCAAAGGCAGGGTCTACACTTTCCTTCCAACTACCTCTGTACAGCTCCAAAAGTCTCTCTGCTTGAGTCACACCTGTATTGGCAATTTCAACAACTTCGTCAAGAAAATGGGCCTCCCCATACCCTCTTCTTTGCAAACCCTCCTGTGCCAAGTTGACCACATCTTGTGCCACATGTTTTAGAAGAGAGTCTCTAAAAGGAGTCTTCAATCCCAATGTTGGTACCTGGTTTCTCAACAGCAGGTGCTCATCCAAGGTCCAATCCCGTATTAAATCCAATGCTGCTTGAAGTGATATGTCATCATATAGTAGACCAACCTGCACAAACAAGTTTTTCAAAAGGAGATCCCAAAGGATTACAAGTAACAAGTTCATCTATAACTTTTTCTTTTTTATTCATGATTTTAGGTAAGGGCCTAGAAAGTGGATCCCTGAGATTTCCATCCACACACACCCAACCCACGACAGGTTTCAAGCTCAGGACCTCGTCCTTTCCAGCTCTACAACCAAGTCATCACGTGGTGCTCCCATGGACAGGTTCACCTGGAGCTTAAATATGTACCCAAAAAGCTGGCACTGCACATAGTAGACTCCTTGGACCTGCATCAGCTCCTCTAAATTCCAGAAACTTTTTGAGCCTCACCTCTGTATAAATTGTAGCAATATGAGATTCCCAGTCTTTGAAAGTAGCATTCTCACCAGGCAAATTAGGTAAATTTCCCACAATAAAGTCCTTGAAAGACATGCCACAACAGTTGAACAAGTGATTATTGCGATACACCCAATACAAAGGAACATTCAAAGCATATTCAACATATCTCTCGAATCTGTATACTAACAAAAGAAAACACTTAGACCCCATTGACCAACCCCTACTGTGGCTTTTTACATTGTCCTTAAACAGGTATCATGAGGTACTCTTTCAATAACGACGCATCGGCAAACCCTGATTGCAGACGTACTCCTACTTCCCTTGAATATCTTTCCTACTGGTGTTACAAGGGCACATTAATTAAATGGTTAAAATGCCGGTTTGAGAAGCACATAATGTCATTAATCTTTAAGTAAAACCCTGTACTGGCCTAGATACCCTGTACTGGCCTAGATATACAAATTGTGAGAGACCTGATTTCAGCAGAATATGAAAGAAAGGTCAGTGCTGAGCCTTGTACCCCGTCTTTTTAGCTTTAGTATTGTGTTAATAGTAATGCTCTGAAGCTTTTCTGCATTAGCCTGGAAGACGCTATGAAGAGTCTAGCATCTGCTTGAGCTGGCCAATAGAGCAAACACAAGGTTGAAGATTGGAAAATATTTAAATTATCTCTCTTCCATATTTAAAGCAATTTATACATTAAGAACATGTCATTCCTAGTTAGAACACCGCTTGGGCATACTATGGACACAAAGGTTTCGAACCTTGTGTTTGCAGACCAGAAAATAAATCCAAGTAAATACAAGTTCGCGTATGTAAAAGTTAGAACACTTGTGTAAGCATCATAAGACAAATAAATCAATCTTAGAGACTGGAATTCTGGCTGTAGATCAAACTCATCACAAAGCTGTCAATATGAACCAAAGCAAGTCTGCTGTAAGTCATCAACATATAGTAATAGCATGCAAATCAGCTTGAAAGTTATGCTCCTCAAATCACGTTTCTTTTTTTCCGAAAACTCTCACACCCCGGCGCCACATTTGTGGCTTTCTAAAAATCAAGAAAAATAACGCAAATGCACAAAGGATATCACAAAGAATACACCTAAGAAAATGAAGCTCCAGATTTTACCCAAAGTTGTCATCGAAAACAAATGGTAGTATGCCAGTCCGATTGTTATCCAGGTCCTTATATAGTTCACTTCTGTGGCTTAAAAGTCCACTAGGCTTCCCATCTGCGAAAGGAGAGTTTGCAAAAAGAGCTATTGCAATAGGTTGTAGTGCAAGTCCAAGTCGAAATTTGTTCATCATATCTTTCTCAGAACTGAAGTCCAAGTTCACCTGAGTAGAGCATGCTCGAAACAGCACTTCATTTCCAGTAGTACCAACTTTGGGAAAATATTCATGCATCATTCTGCATTTTTTCTTAGGCATCAACGGAAATGCATCAACTGGCCACTTGGGATTAAAACCCATCCCACATAAGCCAAGCCCCAGCTCATCTGCTACAGATTTCACCTCGAAAATATAGGAGTTTATTTCTGCCTGTGATTGATGTAAGGTTTCAAAAGGGGCACCACTAAGTTCAACTTGGCCGCCACCCTCCAAAGTCAAACTCTTTCTGTCCTTTAGTAGTCCAACTAATAATCCATCCTCCTTGATTCTGTCCCAGTTGAAACGCCCGGCCATGCCT |
| **DICER-LIKE 4 (DCL4):**  GAGTAAATTATGAGGAAGAGGAACTAAGGTGATAATAATTATGTACTATGATAAGGACAGCTTTCAGGCTCTATTCATTAATTGGGTTTAAACAGGCATGCCAACTAAGGTGCATGTCTTTTGGATGGCAAAGTGAGATAGGCTTCACAAAAAGCCACAAGAAGCCAGCCAAGTCAGGAGTTCTGCTGCTGCTGAATCCTTGGCTGCCTTAGCGGATTTCCTTGGATCTCCACGACATTCGACGAGTCCCACATCTGGCAGCTGCAGTACGGCATCGTATGTGAAACACTTGTCATGGGGTGGACCCTCCTCGGAGGAGCAAGTGAATGATGGGGGCTCCCATCTTTTTCTTGAGCAAGCTTCATTCAGGGATGCTCTTGCAAGGCCCCTTGATGCTGCTTGCGTTGTCGTGTCGATTGTACATGCAGGGACAGATGCTAAATTGGCAAACTGAAAGTTAGTTTCATTTCTGATACCAACACCATTAGTTTTCATTAGCCATGAATTCTCCATAGACATATTTGAGCGCTGAAAGTAACTTCCTGGCTCCTCTGCTATTAATCTGGATGTACTTTTTTGCTGATCTTTGATTTCATAAAGAAGTCCTTTTCCATTATCTTCAATAAATTTTTTGGCCTCATGATCCATGATTGATGCTGCACATTTCTCTTCCCTAGTCATTCCTACTCTAAATTGTGGCTCATTTACATTGCTTACTCCCAATATTTTTTCAAATCTGTTACTAATAGCACTTATACTGGCTATATGGTCCTGTATATGGTGAGTGCTACTGCCTGCACCAGTTGCTAAAAAGCCAGAGCTCAAAGCAGTTGATGACAATGTGGCAAAGGAGCTCACTCTAAATTGTGGCTCATCTACATTGCTAGCTCCCAATATTGTTTCTGATGGCTCCTTCGCCAATGTGGCAAAGGAGCCCACTCCAGATTGTGGCTCATTTACATTGCTAAATTTCCGTATTTCATCAAATTTCGTGCTGCTGCCGGCACCAGTTGCTAAATATTCCATGTTCAAATCAGTTGGCGACAATGGTGCGCTGGGGATCATGTTTTGGGGGGTGTCACTTATTTTGAACTTGTCTGACGGCTCCTTTGTGGCATCCCTGCTCTTTGCTTTCTGAGAGTCTAGAGTCTCACCCTTGCATGAGTGAACATAACCTAGAACCTTCAGTTCCCTAAGAGCGTTAATTGCAGCTCGTTTCTTTGCAGACTTCTTATCTTTAGATACAGAAGTTCCCTTGACAATATTATTGCTCACATTGATTTCGTAAGTGTATTGGCATTGCCGGCCACCAAGAGACGATGAACTTTTAGTCCACGTCAAGTGATTCTTTTGGCATACTTCTTGCAGCTCACGAACAGGATGAAGACGTACAAGGCGACTTGAGATCAGACGCCCCAATATTGGCTCAAACACTTTCCAGACCAGGCTCAGGTTAAACTCTCCATCCACACATATAGCGCCACTCAAGGACTCCAGAATGTCAGCAAGCACCTTCGAGCACTTGTCTCCTTCCCGGTCTCCAATAAGTTCCTGCTCTGAGGAAGCACAGATGTAGGAAATAAATTCATTTATGCCTTTCCGAAGTTCTGTAGAGTTCTCTATAAGATATGCATAAAGTTTATGCTGAATTGCAATCCTGGAAAAGCTTTCGTTGCTCACTATTGTCGACCTCAAGTCAGTAAGCTCTCCCGGTTTCGAATCCTTGAATTCTCTGTAAAGGTGCTTTGTGATAAGGAAATCCAAAACAGAGTCACCAAGAAACTCCAAGCGCTGATAGCATTTCCCGAGATGATTGGTAAACGAAGCATGTGTGAAGGCTTCTATTAGAAGCCCTTTGTGCATAAACCTATAGGACAGAAGCTTCTCGATTGCTTCAATATCAATTCTTCTGATCAACGAAAGATTGTTCTTGCTTGATGACTGAATCTTGACAATTTGTGAGATGTCTGTGGCTACTTCCAGTCCAATAAATTGCATGAATGATACCGCAGCCTGCTCACCACCATCTTCTAGGTAAGCACCAATCAATGCTTCAATAACATCTGCAACAGTTTTCCTCTGCATCCATCTGTGTCGCTTGTTACATGTCACAGAAGCCTTCCATTCTTTGCTTTCACTGGAGTCTCCGTGTAGGTCCCCAAGTAAACTTTCACCACACACAGCTTTGCTGGGATGGCAAGGTGCTACCCAATGCTTAGGATCAAATAAGGTATCACGAATATATCCAGCAAGACCAAGTTTGCTTCCAAGTGTGAACAGTGAAGAATTGCAGATTCTCTGATTACGTTGCAAACTCAGAAAACCTTCATCCACTTCCTCATGCTCAAGGAATAGACGACGACTGATGGCATACTTCAAGAAAGAGTCTCCAAGCAGCTCCAATCGTTCTAAAGAAAATGAATCTAGGCATTTCTCTGTCGTTAGTGCTTCCAGAATCTTTTTTATGGAGACCTGAGACCCTTGCGGAAACTGTGAGCTGAGAAAATTGCGGAATTGAGTCGCAACAAGAATGCTTTCAAGTTTGTGCAGCAGGGATGGCAGAATAACTGCAGCATTAACCAAATCATCATAAAACCCAGAAAATTCGATTCTGCAGACCTCCGGAGGTAGCTCTACTAATGACTTTTCTGGCTCCACTGACACTTTTTCTGCTTCCTTCAACTTTTCTGGTGTAGCCTTGCTGCCCTGATCTGGTGACAGATTCAAAACCTTTTCTCTTGACCTAGCCAGTAAGAAATTGTGAGCGTCTCTTAGAAACCTAGACTTTACCAAGTGTTGTCCTTTAAGAACTATGTCATGGTCATACATGGTCTTAAAATACTCCACATAAGTTTTGTATCGTGCATTTGGAAAAGAACTCTCTGCTCTCAGATCTGTGAACATCTCAATGACGCAGTACAGATGTCTGCCATGAAGAGTCTTTACTAACAAACCAGGCACATCCTCTATACGTATAACACCGTTGGAGAAATATAAAGCAGCACCATCGACTTTGGGGCAAACATTCTCTGTCACCCCAAAGGTGTAATTTGAATCTTCAAAAGGTTTTAGTTTGGAAATTCTAGTCCAATCAATTTCTGGCTCCTTGTTGGAATTGATCGGTTTGAGTGGCAATAAAAGATACCAGGAACCATCTGATATAGGAGGCTCTTTGGTTTTTGAATGCTCCAAATCACGATCCAGAAGAACAGAGAACAATATTGATTGAAATTGCTCTGCTTGTGCTACCTTTTGTAAGAAAAAGATTCGTCAACTTATCCCTACTTTCATCATAGGATACGCTCCTTATGTTTTTGCAACTGTTTGTCTAGCATATCTCTGTGAAGTCCGTCACCCTCTCGCATATCATTAGCGTACGTCTGTGGAGCCCGTCACCTTGTGCGACCGTGCTGTGGAGCCGCCCTAAGCTCTGTCAGCAGCGCGCGCACTACCTGCAAAGCCTTAGGCTGCTGGTGGTCGCATCCTTGCCTGGTTCACAGTCTGAACAGTGGGCCTTTTTTTTGCCTCGCATTGCTCTCAAGGCCCACCCCCATCTAGATGAAGTGACCTCGCCACAAGCTGCCGCAGCTATCACGCGACCTTGTCACCTGAAAGGGATCAAGAAAGACATTTTTGACAGGTTCAAGCTTGCATAAAACAGCCCTTCCATGACGCAATTGGAGGGTTTCATGGAAGTTAGCAGCCCCTTCGGGTAACGGTGATTGAAGCAGCAAAACAAAGCTTACGTACTCCCTGTCACTAGGAATAGCATCGAAAGATATTCTGTAAGCCTGCAAGCACATTGCACTAGAACTACTTGTGGGTTGCCGAAGCCACACGTCTGGTATCACAGTCTCCTGTAGCTCCTCAGTTGTTGCTTTGACAATGCTTGAATGATGATCTGTTTCAGCATCTTCTTCTACTGTGGTTGTTTGTGGTAAGAGATAATCTGTTAAGGCTCCCTTTGAATGTAGTAATCTGCATGCTTTCAAGCAGGCTGCCTTTTTGGCTGCAGCTTCTGAGTCACAAATTTCTCCTTCAACCAGTCTTAGGCAGGCGTTTGAAGGAAGTGTAATGCTGCATTTGATTCCATCTTGTTCTTTTAAAAAATGAAATGATGGCTTTGGCTGATAAAATTCATCACTTGGAAGCTTGGAGCAATAGCGATGAAGTAATTGAACACTGGAATGAGTGTTGACAATAGCTCCCGTGCTCTCCACTTCATATAAATCAAAATTTTCTCGCTGCCTCTGATTTGTAGATGGTAAATGCCCTTGTGAAATTTTCTCTTTGACAAAGCTTTCGCTGTGGACCAAGGTATCTAATAACGTTGCTTCCGAGATGTTTGTTCTATCCTTAAGAATAACATAATGAGA |
| **CHITINASE A (CHIA):**  GTTTGCTTGAGATGGCGCGGTTCAACACAATTATAAGCTGCACCATCCTGCTCTTGACATTTTGCAGCCTCACCGGCTTCACGGTGGCGGCCGGCAACCTGGTGACCTATTGGGGCCAGGGGGGGGGCACGGATGGCATCGAGGGGACCTTAGCAGAGGCGTGCCAGTCCAACCTCTACAATACCCTCATCATCTCCTTCCTCGACGTCTTCGGCCAGGGCCAGCAGCCAAAGCTCGACCTGGCAAACCACTGCGACCCTGAATCAGGCACCTGCACCAGCCTCTCCAACAACATTTCGACTTGCCAAAGCCTGAGCATAACAGTACTCCTCTCTATCGGCGGCGCGAACGGCACCTACGGCCTCTCTTCTGCTGATGACGCCGCCACTGTTGCTAGCTACATCTGGAACAATTACCTGGGCGGTCAGCAGTCCAGCAGCCGCCCTCTCGGACCGGCTGTCCTGGATGGTGTTGACTTCGACATTGAGACAGGCACCGGTGCCTCTTACTACGGCAGCCT |
| **NECROTIC SPOTTED LESIONS 1 (NSL1):**  CGGGCCGCGAGCTCTCTCTGATTAGTACGGGCGGGCCGGCTTTTTGTCTGCTCCCGCTCCTCACCCTCTTTCACTTTCAAAGTTAAGCATCTTCTCCTCTTCTCTCTTAACAATTCTGTGTATGCTCCTTCTGCACGTCGCCTCACGCTGGCTCAAGCTTAACGAAACGGCTTTGACCCCCTCATGTTCATTTTTTGCAGATTGCACATATTCATATTGGGTTGTTTAGTTTGAGCTTTCGCAGCCCTTCTCCTTGCCTCTTCACATTCCTTGTGTCATGGTGCCATAATTGGCGATTGTACCCCCCCAAAAAGACAATCCTGAATGAATACAGACTGATTTGAAGATCTGTGTAGGGAAAGGATGTGCTAAATGGCTTCCAAGCTTGACATCCCAAATGCAGCTAAAGTCGCTAACACTGCCATCCAGTCTCTAGGTCGAGGTTATGATCTCACATGTGACTTAAGGTTTTCTACTTGCAAAGATATTGATGGCACATCCCTAATTGAGCTGAGAAACAATGAGACGGTTGAGCTTTCGCTTCCGGGGGGCGTGGTTGTCGCAAATGTGCCTGCAATCGTCAAGTGTGACAAGGGTGAACGGACTCGTTTCCGGTCAGACATCCTCACTTTTGATGAGATGTCTCAACAGTTTAATCAGGGCCTCTCACTATCTGGTAAAATTCCATGTGGTCTCTTCAATTACATGTTCAATTTCACCGGGTCATGGCAGAAGGATGCATCAACAACAAGGCACCTTGCTTTAGATGGTTGGTTTTACACATTGTATACTGTGGAGATGCCAAGATCTCAACTTGTCTTGAAGGAAGACATAAAAGCATCAGTTCCAACATCCTGGGAACCAGCTGCTTTGGCCAGGTTTATTGAGACTTTTGGGACCCACATAATTGTAGGAGTCAAAATCGGAGGGAAAGATGTTGTTTACATGAAACAGCATCAGTCATCACCGTCAACATCTGTTGACTTTCAGAAATTACTTGCTGAGGTGTCAGATGAACGATTTTTGCAGACTGAAGGTCGTACCAGTGTGGGCTCGAAGGACAGCCGCATCAACAAAAAGCGTAGCGTAGAGTTTCAGTCATGGTCAGCTCATTTGGACACCTTCAGTCAGATAATCCACAATGATAAACATCATGTGACCATTATACCAAGAAGGAAGGGCGGTTTTGACCATGGTCAAAGCCATTCCGAATGGATTCATACTGTCCCTCTTGCCCCTGACGTGATTTCCGTGAGTTTGATACCCATTACCTTCCTACTCAATGGCGTTGCTGGGAGTGGTTTTTTGAGTCATGCTGTAAACCTCTACTTACGCTATAAACCGCCTATTGAAGAGCTGCGTCAGTTTTTGGAGTTTCAGCTCCCCCGTGAGTGGGCCCCAGTTTTCAGTGAATTGCCTTTAACTTTGTCTAGGAGAGAGCACAGCCCTTCAACTTTGCAGTTCACTTTGATGGGTCCTAAGCTTAAAGTTAGTAAATTGCAGGTGACAGTTGGGAAAAAACCGGTAACAGGAATGCGCTTATTCCTGGAGGGCAAGAGGTGCGACAAACTTGCAATTCATCTTCAGCACCTGTCGGCTATTCCCAGATTTCTACAACCTCTGTGGGAAGATCATCCATTTGCAAGAGAGGTCACATGGCAAGATCCAGATGACTACAGTACAAAATATTTTGAGCCAGTACAGTGGAAGAATTTTTCGCACGTCTGCACAGCACCTGTGGAGAACACGGAGACATGGATAGGGGACCATGCAGGTGCATCTGTTGTTGTTGGAGCGCAGCTTGCAGTGAAGAGCTTTGGGCTGAGAAATGTGCTGTATTTGAGGTTGCTTTACTCAAAGATACCAGAAGCTAAGATTCGGAGGTCCGAGTGGGATCACATGCCAGCTTCTACACAAAAATCTGGAATGTTCTCGAGCTTTTTAAGTACCACTTTCTCATCCACACCGATGCCTACAAAGTATCCAGCTGTGGTTATAAACTCCGGCATATTTCCAGAAGGCCCACCTAAACCTGTACACAATCATAAGCTTTTGAAATTTGTTGATACGACTGAAATGACCAAGGGCCCTCAGGATATGCCTGGGCATTGGCTAGTGACTGGGGCCAAGCTCTACCTGGATAAGAGAAAAATCTCTTTACGAGTCAAATACTCTCTTCTGACTGTTAGCTCAGATTGACTCTAAATCACAGCCTTGTAATTTTCGACTCTATGGGAGTCCTAAGAGGAAGGACTCCCAAATCATAGAAGTGAGGTCAACTAGTCGTTACACATCGGCGCTGCTCTGTACAGTTTTTGCAGAATGCTCAAATAGAAAGTTACAGTGGAGGTTGGGAGCTGAGTAAGCACTTGTGCAGAAGCACCATAAAGTGCCCAACAACAATCAGTGTTGTGACCTAATACTCATCAACGCAGTGGCGGTGAGTGAGGCTTAGAAACATTATCCAATCTTCTGTAAATTTTGTAAAACGCATGTATATGGATGCACATAGTTTTTTACAGTTGTGTGGCTTTTAGAAGTTCAATGTGTGTTAGTCCCAGCTTTGAAGCATCCAGCTTACATGGTGAGTAATGTTCAGGGCAGGATTGATGCTCCGCTCATTTGTACTTAAAAGGCTGGCTACCCTTCAGAGTGTGTGCTTGACGATTCGGGTGGACCAAGAAGTTCAAAGCTTCACAAAATTTCTGATGCATAGTAATGCTGGGGAAGACTAGACCTTAAGCAAGGTGAAATATTGTAGCACAGAGAATTTGAATGCATATCATCATTTTGTTCAGTTCATTGGAGGAGCTTGGGCTCATCTCTTGACATCAGACAAGCGCTGGTCCGAGGTCGTTGAGGCGATGGTCTGAATTCATATCCTCTAGACCTTAACAACTAAGCAAAGTGGCCAGAGAACTACATATTTTTGTGTGAAAATGGTAACCTTTTTTATTTGGCATCGAAAAAACAGTGTACTGCAAAAGAGGAGTACAGAGCATCCAAGCCAACTGCGGCGGAAGAAACACAATTTCTCAGCACGAGCTGGTATGACAAGAAAGGACTAGAGTGTGCTGTGCATCATCCAAAGAAAAACAAAACAAAACAAAACAATCCTTGCCGTTTTATGTAGTTATTTTGCATATTGCAACACATGCCTTTGCAAGGTTGTTCAAGGTGCTCAGTATAGTGGAGGTATTGAAATGTGCAAATGGCTCAAATGAAGCACAAGAAGTTTCAGAAAATTTCCTTTGTGGATTTTGACTACTAGGGAAAAGCAAAGGGAAAGGGGCCTCTTTCTCACAAGGAAAAAATCCAAAACTTCATGTGTATATACCAAATTTACAAGGGGGTGTCATGAAGAGAGAGACACAGAGGACCTCAAGTGATGAGAGCTTAGAAGTTAAGGAAGGAGATGGAGTCCAAGGCTTGAGCGAAGAGTTTGATATGGAATGAGATGATGAGCATGATGGAGGAGGTGGTGAGGATTGAGTTTCAATTTTGATATAGAAGGAGATGAGGGAAAAAAGTATTGCAAGATGCCTGAGGGTCAAACAAGCCCCTACATAAAGAAAAACGAGTTTAAAATGATAAAAAACAAGATGATATTCCACACTCAACTTGCCATTGCGGTCAAATCGTCTTGCAACCAACCGTACGTTACATGGACAAGCTACAACGTGTCTAAAGAACAGTATCTTTGTGGCAACCATCACAAGAAGTTTTGGACATTTTTC |
| **FATTY ACID AMIDE HYDROLASE (FAAH):**  TCTCTCTCTCTCTCTCTCTTTGTGGCCAAGATAAATGTAGCTGGTATAGAAGGGCAAACAAAGGTTTGCTTTCTTTGATAGGCACACGTGCGTGCACATGCATGCGCCTGTGTGCACGCGTGCGCTTGCGCAGCTCTTAAAGGATGCACGCACGTGCGTGCGCGCGCACCCACACCCACACCCACACTCACACAGACAAAAGCATAGCTTCTTCCTTAACCACACAGATTGCCGGGCTTTGAACAAGATTACTATCAAGATCAATTTAGAGCACGGATGGATCGTATCAGATGTGTGTTCATTACAAAGGGTTACTACCAAGGATTGTAGAGGAGCATACAAGGCACTTGGCATGAATGTCTGAAGAGCTAAGCAATGGGTCAGAAGTCTATGCCCCTTTTGGAGGATGTTAAAATTGATGCTGTGAAGTATCACCGTCATCAGTTAGAAGCTCCTCACCTTACTGGTTATGGCCTCAAGATTTCTACGTGGTTGCTGGAGAGTTATGTTGTTGGTGGCTTGATTATTTCAAACACATTACGTGCAAATAAGTACACAGAGAAACTATCCCAAATTGTGATACCTGATGCGCCATTGTTTGCACCTCAATTTCCTGAGCAAGATCTGGAGCCCGCTGTCAAGGCAGTTGAAGAGACAAGTTCACCTGTGAAAAGAGTTGAATTAGCTCTGGAATGTCTCCCTCCTGGCTTCAATAAAAGATCTGATGTTCCATTTCAATATTGGACTATCCGGGATTATGCTCATGCTTATCGCAATGGATCTACTACCCCTTCAGATGTTGCAAGGCGTCTTATTGCGGCCATAGAGGATTCTCAAACTAGAGACCCTCCCATGTCCTTATTTATTTCATTTGATGCCAAAGACATTGAGAAGCAAGCAGCAGAGTCAACACACCGTTTTTCTGAGGGGCGTCCTCTTTCTATATTAGATGGAATTTTTATGGCAGTCAAAGATGAGGTTGATTGCCTTCCGTATGGCTCCCGGGGTGGCACAACTTGGTTTCACAAAGTAAGGGAGGTAAAAAACGATGCCGTTTGTGTTGCAAGGCTGCGTGAATGTGGAGTAATTTTTGTTGGCAAAACCAATCAGCATGAGCTTGGTTCAGGGACAACAGGCAACAATCCTCATTATGGGACGGCTAGAAATCCCCATGATCCAAAAAGATATACAGGAGGCTCTTCTGCTGGATCAGCGGCATTGGTTGCTTGTGGACTGTGTCCAGCAGCTTTAGGAACAGATGGTGGAGGGTCTGTTCGCATTCCCTCTGCATTATGTGGCATTGTTGGCTTGAAAACAACGCATGGGCGAACTTCGACTGAAGGTGTCATCGAGCTTGGATGGACAGTGGAGGTGGTAACACCAATGGCAAGCACTGTTGAAGACTTACTGCTTGTGTATGCAGCCATGCTAGGATCACGGCCTAATGATGTTACAATTTCTAAGCCAGCTTTGCCAAGTTTTCCCATAATGGAAGGGTCTCAACACGATTTGGATGTTACTAAAGTGATAGGCTCCATTACATTTGGGAAATATACTGAGTGGTTCGAAGATGTTGCTAGCTCGGAAGTATTGGATGCCTGCGAAAAGGTTTTGCAGCTTCTTGTAGAGAACTATGGAATCAAGATTAAGGATGTAATTTTGCCAGAGATGGATGAGATGCGCATAGGTCACGTTGTAACCATTGGATCAGAAATTTCTGCTGCTACTAATACTTACTATCTGAACGGGTTGGTTATTGACTCTGGTGCTCTTGCTGTGTCATCTGAGTTTTCTAAGCAATTTTACTTGAATTTACGATCTGTTAATGTGTGAGAGTGCTGCCTGCTTTGTTACGAGTTCACTTTCACTTTATATGCATGTGGCTTGAGTACAGGAGAAGAAAAGATATTTCTTATGAAATCAGAAATACATTAGCTTTATTACGGTCATTTACGTCACGTGATTATGTGGCTGCTCAGCGCCTGCGGAGAAGATCCATGTTCTATCATATGGAAGCATTCAAATCGGTGGACATTATTGTGACACCAACAACACCCATGACTGCACCTGTCATTCCCAGAAGTGCTCTCAAATTTGGAGAGAGCAATCTCGTTGTTGGAGGAGACCTGATGCGATTTGCAGTGGCACCAAATTTTTTGGGTTTTCCAGCCATCACAGTTCCTGTTGGATTCGATAAAGATGGGCTTCCGATTGGTCTACAACTAATAGGACGACCTTGGTCAGAAGCAACTCTACTACGGTTAGCAGCTGTAATTGAGAAGTTGTGCGCTTCAAAAAGAAAACAGCCAGCCGTGTTTTATGATCTTCTTGAAGGCGTGTAATGAAAGATGCCTTACCAATTTTAGATAAGAAAGGTCTTGTGATTGTAGGCAGCCTTCAAATTTTTCATTTTTTCGAAGTTTAATCCAACAAATAATTTTGATGTAGTTGAGTTTATTCAGTGTGTTTCATGCATCAAAAAACTGAGTTATGCAACATGAAGATGGTGGAACCTGTGCACTACACTATTTTTAAAGAGAGTAATATGCAACTTCCACCTACTTCCACTTGTATACTTGACTACCTTGTAGACTTGTCTTTCCAAATTAACTACATGCAGCTTTCACTGTCTTTTTCAAAA |
| **PRIORITY IN SWEET LIFE 4 (PSL4):**  TTTTTTTGAAGGTGGTAAGATGCCTACATATTGAACCTTTAAATTTGCATTCCTTCGGTATACCAGAGTATTCTGCTTGCCATGTCTGTATTAGACTTGGCTGACATAATTAAAGATTGTTAAACAGTACAGATGTCCGTCCTACAAAAAGAATCAGCTTTCGCTTTGCTTGCCATAAAGCGTAGCTTTGGGGATGCCAGTTTCCTCGGCCAAATACTTTCATAGTATTCAGCACTATTTTGAAGAGGAAAAAATACTCCCTGCAAAACTTTTGGGTGCCCTTCTCAAGCACATTAACCACTCATCATCGATATATTGACATGAAAATAAAACATTACAATAGGGGGTGAATGTTGTGCATTGAATTACAGTTCATCGTGGGCAGGAACATCGCCTTGATTAAGCAGCCGCTGTTCCAAAGTCGCTAATCTTTCTTGCAAACACACCGCAGGTGTGGAGAGCTCAGCAACATACTCGCAACGACTTGGCTCATCTACATCCCTCAGCTCTATTTTAGAACCACACCTCAACTTCACCTTCAAGCTCCGATCCGGACCATTCCAGCACTTGTCACCCCGTACAAAATGCATGCTTTTATACTCATCTTTGAAGCCATCCCAATTCCCTAAGGTAGTGGTTGAATGGGCCTCCACCTGCACCGCATGTCTAAAAGGACAAACCTTGTACGTATACTTCTGTAGCCGTAACTCGAAGCAATGGCCATAAAGGCTATAGAACTCTCCATCCTTTCCAAAATCTTGCTCCAGTTTCTTCTCAAGATCCGAAATCTTGGTCTGTATGTCGCTCAACTTTGAGGTTGAATCCTTGTATTCTTTGCGAATGTGCTCAGCTTCAGATTTATCCACTGGTGCTTTCACAAAAATGCCATCTGAAAATTCCTTGATCTTTTCCTTCAACTTAGTCCACCAAGGACCTTCCTTCGAAGTCGCATGACCTGAGGAACCAGATGCCTCATCTGTCTCCTCTGCCTCATCCTGATCATCTATAACATCCTTCTCATCCTCTTCATCATCTTCATCATCTGTAGAGGCCTCATCTTCATGGTCTGCATCATCATCAAACGTGCCTTCGGACTCTGCATCATGATCATCTTCCACTCTTCCTGCCTCTTCTTTCTCAATGTTTGGGTTCTCTCCAGTCCAGCGAGATGCTACAAGACGACCTAACTCTTCTTTTGACAAACTCTCCTGGTTATGTTCTGATGAAATGATGTCTTTATCATCATCCAGTGTCACTTTCTCTTCTTCAAGACCTTCCAGGTGGTCTTCCAAAATCTGATCATCTTTTGCGGGTTCTGTTTTTTCTTCTTCGGTAGCTTCTTCTTTCGCATCTTTCATCTCTGTTTCTTTTCTTGCCTTCTCTTCTTTCTCTCGCTTCAGCTTCTCGTGTTGCTCAGCCTTTTCGATTTCTTCTTTCTTTGCTTTTAACTTCTGAACCTGGTCTTTCAACTTCTTCTCTTCTTGCTTCAACGTTGAGAGATCCACTTTCTCCTTTGCAATCATGTGTTTGGCATCCTCAACATCTCGTTTCCTAACCTCCAACCCCCCTTTGAATGTCGCAATCTTCCTCTCCAACTTCTCCTTCATTACTTTTCCTACTTGAGAGCATGTATTGGAGCACTTTGTCTTCCCATCATACTCATCACTTCCATCACAACAATCACAAATGCCATCATTGACTCTTGAAGAAAATATTTCCACAGGTGTATGCCCTTTATTTCTACAGTGGAACTTCCCATTCGGACAAGCAGATGTCCCAGGCTCATCAGTCCCATCGACGCAATCACAAAAGTCGTCGTTTAATCGATTCGATTGAAAGCTCTTGGACCCATCTTTACACCGGATAGTCGCTGTTTTGTAGTAGGCTTCATCTTGAAGGGCGATGCCTCTGAAGTTGGGGGAGGGCGCAGGAGCTGCAAGAGTGTGAGAGAGAAGAGAAGCTACCAGACAAGCTTGAAAGAGGAGGGCGAGGCCGCCCATCGCTGCTCTGAGATCGGCTCTCCTCGAGCAATGCTCGTAAGCGAACACACAAATCTCACTGTTCCTTAGAGAGAGACAAATAACATGGAGAGAGATGGCATGCATTTTTTTTCTTAAGTGAAAATAAAGGACCCAAAGCCGG |
| **SIRTUIN 2 (SRT2):**  GTCAAACCCGAGGTCGCGGGTTCGAACACTGCTGGCTTCCCCCAAAGAGAGAAAGTCTTTAGAAGCTTTAATACAAATGTAACTCCCAACCAAGTGACAAAGAGAGATGCGACATACATGCATCTCGTTGCTAATTTTTCATAGGATGGTAATTTTCTGATGAATATATAATTTGCTCCCTTATGTTGAAGACAGTCCTTAATGGCAGGAAGGGTTTCTCTCAGGATGATGGCACCATCTTTAGTTTCAGCTTACAAGCTTTGCAGAACTTTTATGCTGTTTGAGGATCAAAAGCATCATCAGAAGGACCTTATCTGTAGCACAGATGTTGCATTGCAGTTGGATTTGCAAAAGAAGCCGATTGTTCCTGAGGCGCCCCCACCGAAACCTGATGAGATTGCTTCTCTTAATCAGTTTATTAAAAACAGCCACAAGCTTGTGGTACTAACTGGAGCAGGCATAAGCACGGAATGTGGAATCCCAGATTATAGAAGCCCACACGGAGCTTATAGTACAGGTTTCAAGCCAATAACTCATCAGGAGTTTATTAATTCTGAACAAAGTCAGAAACGGTATTGGGCGAGAAGCTATGCAGGCTGGAAAAGATTTATCTCTGCTGAGCCAGGAATAACACACATATCCATTGCGAAGATTGAGTCAAGGGGATATATTCAGCATATTATTACACAAAATGGTGACAGGCTAGTCACTACAATACTTGGTTGCACCATTGTGCAGGCAGCAATGCTCTGGAATTGCATGGCAGTACGCACGAAGTTATCTGTTTGAACTGCAGTAATATAACGAGTAGGCATGAGTTCCAAAAGCAAGTGAAAGAGTTAAATCCAGAGTGGGCATCAGCATTGGAGGCTTTGGAAAATGGCAAGGCAGTCTTAAGTTCTGGGGTGCAACAACGCCCAGATGGGGATATAGAAATAGATGAGAGATTTTGGGAGGGGAGGTTCCAAATTCCCACATGTCAAGAGTGTGGGGGCGTCTTAAAACCCAATGTGGTGCTCTTTGGTGCCAATCTTCCAAAATCAAGAACAGAGGAAACACTAGCCATGATACGGAATGCAGATGCAGTTCTTGTCTTGGGATCTTCGCTGATGGTTTTATCTGCATTTCGACTTGCCAAAACAGCGCATGAAATGGGAACACCTTTGGCCATTGTCAACATTGGGGTTACCAGAGCTGATAATTTGGCTTCTTTGAAGATTGAATGCCACACTGGGGAGATTATGTCAAGAATTGTGGACAATGGGTCTTTAGGACAACGTGCGGCCTGATCGAGGAGAGGGATATTTAAACACACAAATACAGATTAAATTAAGCCCAAATAATCATGGGATTATACGCAACTAATCAATGGACTAAATTCAACAAGCTGGATGTTTGGAAGGCTGTGGTCGATAATTCTCATTATGGATGGTTGTTCTACTGCTCTCATTGCATTTAAGCCTCTTGAATGGCATTCCACATAGAGGTGTTTTGTAAATGTATGGGACCTAAGACAACATTCGTTCTCACATAAAATGAAGAGTTTGAAATTAAGCATACCCATTGTCAATTTATTTTTCTTCTTTGCATCAAAATTAAAAAGTGGGCCCAACTGTTTTTGGTTAAACACTCACT |
| **LAZARUS 1 (LAZ1):**  CATAAAAATACTAAAAAGGAATTGTCCATCAAATGACAATTCTTGATTAGAAATGTTCATGATTTTTTTAATGATTAAAGGTAACCTCAAAAACTACTTGTCAATGGAAATCCCAAATTCTTAAGAGACCCAACAGATTTTAGCATGGAATGCCATACGATTAACTATTCTCTGATGCATTTGCAAGGCCAGATTTGTGCACCCACATGTCATGCATTTTTTTGTCTCTTAACTCTGAGGTGTACAAGGCTGCAAAAAGCCTGCACACTTTTTCTCATGATTGGAGGCTGATGCATTTGAGGCATGAGAATCACTTTTGGATTAGTAAACTTCATTTACACGAAGCAACCAAAATGGATGTATATACATCAAGACTCGTTACAAAAAAGATTTTGCTGTTGTCATTGCACAGTCCAAACACCTTTGTTTGGAAGCACGTGTATAAAAGAAGATAAAATACATTGTAAGAGGAAGGCGGGAAGACCCATCTTTTCTTGCTGTTTGTCAACTTGCTCCCATCCCAATTCCAGTCCCTTAATTGTAGTTGGAATTCGAGTACAACTCCATTTCTCTTATGCTACTTATTCTCATCCTGTGTCTTTGAATAATTGTTCTCTGACTTGAATCCTTAAAACTGCAAGAGTACACCCAATTGACGAGGTGACTTGCACCCTAGATAAGATCTGGCTCCATCTGGCTTACAAAAAAGCGCTAAAAAGGAGTGGCAGACAATCATTTTGAGTATCTTTCATTTACTTGAATGTCCAACGGTGACCCGAAGCATGGTAGTCACCCAACCCCTGATCACTACTCTCCCCACTGCTATCTGCTGTCTTATAGGTGTAGGTGTATTTCTTTGTTTTACGCTTCTCAGCTCCACTATCACTCACACTTCCAACCAGCGAAGGGTCATCAATCCCTCTTATGGTTGGAATCCAGCTGTCATCTTTGACTTGCTTCTTGTGGTCCCTATGCATGGTTGCCTTCAAGCGACCAAATCCTTGCCTAACTGGCTCCTGGACTTTTCCTACAGTAAATTTCACATCGTTTATAACATATTCTCCACCTTTGAATACAAGGTCATGCATGCTTTCCTTCAGACTTGTTCCTATTTCTGCAACCTCCGAGTACGGGAGCCGTTTGACAGTAGGCCTTTCAATATCTTTGACCTCTTCGGGATCAGGAGGCACACCAATAGCAGCATAATCTGACATAACAGAAAGGCTTCCACGCTTTTGTGAGCCCATTAGTTGATAGGGTTTTGCTGGCAATACATAGACATGAGCAACAGCAGCAAATCCCATCTCTATGCAAATAAAGAAATCTTGTATTCGAGCTTCTAAAGATTCTTCATTTTCGAACCTGGCTGATTGCATAGCACAGAAAATGGCTATTAAGAGACCCTGCCACCATGTGAGAAAAACAATGGACTTAAAACATAAGAACTTTGATAATGGTTGGATTGGTGCAAGTTCTTCTTTTGTGACGTTGTAGAACTGCATCAAGCAATATAGTGCCCAAGATTGACTCAAATTCATAACCACAGCAACGTAGAGGTACCCCCTGTTCCATTTAAATTCTCCTTCTCCGTAAAGATCCCAGAATTGTAAAATCAATGCAACCATAGCACTGACAGTCTTCATAATCATAAACTGAACAATGGCGAATTTGAATGCTTGATAAAACTGCCTCCCAAGATCCCAAGGCTGCAAAATATAACTGAGTGGAAAGCTATGTTTCACAGCGGCTCTTCCGGCAGTCTGGACAAGAAGTGGAGTTCGAGGGCCTGCAACTGCTTGCCTTTCCATTGATTCCATTGTCTTCTCTTCCCCACCTAGACAGGCAATGAGATAGCGCCCAAAGGAATACAATGCAAAAGCTTCGTAGCAGTCTCGTACGATCTCGAACACGAGAGCTGCATGTGAGCTGCATAACGACACATAAGATTCTATGGCATACAATGGAACTATCAGAACTACTCCTATGATCCATTTTTGCTCCTCTGGATTGTTGTACCAAGAAAGATGTTCGAGTATCAACAAGGTTGAGAGTGCAACGGCAAAGAGTACAAAAATGCCAGTGAGTTTCCTGGCATTAACTGAAATATAGATGCTTTATATCTCTTCCCTGCCAAAAAAAATAGCAGAAGGTCAATCTTATTACTGTTGGAGCACAATCTTCTCAATTAGTCAAGCTACCCATCAATCATAAATGTTTTCTACTGTCACAAACACCTTACTTATAAACTCCGTCTTCTCTTCAGTAGTCAACACAGATAGAACAAAAATACGAAAAACCCTTGAATAATGCGCACGACGGGCCATGTAAAATGGCTTTCTGTGCACGTGTTTACAAATGGACGCATGTGCAATACGTCTGTACAGCAATTCCATGAAACATGTTTGTGAAGTGCGCAATAGTAATGAGGCAGAAACTGAAAAATTTTACCTCCAGTAACTGTAGACAAATTTCTTCTACTAAAAAACCCAACCTGAGAGAGAGAGAGAGAGA |
| **STROMAL CELL-DERIVED FACTOR 2-LIKE PROTEIN PRECURSOR (SDF2):**  ATGCCCTCTGCAGAATCTCATGATGCTATCAGAGAACAAAGGGAAAGGGGCCCCTTTTTCATAGGGAAAGTGTCCATAACTTCTTGGGTGGATTGTAGAGCTTATCTAGCCGCCACAAAGTTGGTAATTAATAGATGATAGACATTGTTTTACAAACCTTTTGCCACTTTAAAAGGTATAAACATGATTGAAACGACTATTGTATTGTATTAATAGAGAGCACACTTCATATTGTTTGAGCTGTTTAAGAAAAGTTTTATGGCCTCACGGTGAATCACCCAAATAATGGACATAGTGGGATGATTCAACCCAAATCTAAGTGGATGTGGAAATGGGCTCAAGATTTTACTTTGTCAGTTTTCATGGACATAGTGGGATGATTTAACCCTTCCGCCGCTTGGACCCCTCCCTGAAGTGGGACCTTAAAACGTTCTACCCAAGATACAATTGCAAGGAGCCCACCGTCACCTCCTTCCACTGCTTTAATGATTCTATGATTGTGTTTTATGATTATGTATCAATGATTCTATGATTATGTTTTATGATTGCACTTGGTCACAAGCTTGTGTGTATATTCACTCAAACTTACATGATGTCAGCACGATGATGTTCAGGTAACCTATGGAAGTGTGTTGAAGTTGATGCATGGGACCACTAAGAGAGGTTTGCATTCCATTGACATATGATACAAAGCAGGGGGCCCATATCATGTTGTTACTGGTTTCAAGGAGTTTGTAACTCCAATAGCTATTGGGTATGGAGCTCTTCCCCTTTCAATCCTAGTTTCACCTAGAATCACTTTATTGTCAAGTAGATCAGCTTTGTTAGGTGGGTTTCTTGTTTTACATCCTCAAATTTTATTTCTTATTTGAGTTACTTGAAGTTATTGACTTGCAATAAAATGGCTTATTGTACACACTATAGCCGGGAGCTTGTGAGTGTAGGAGGTTTACATGCATGTAGCAGCTTAGGAGATGTAGAAAAGCACAACATGTGTCGTAGCCTTGAAAACACACCCTCTCATTGCATTCTGTTGGTAAATTAGACAATCAAGAAGATGGGCCATGTCATTAGATTATTCTTTAGACACCCAAATTTTCTATTCAGTATGTGTCTTTTATGTACCCTTCTCTTTAAATTATTGGTATAAAATACACATGTTGCACCTACATAAGTGGAGTTTCATGCTGCTACCTTTGTTATACATTAGAAAAGTGTTGATGACAATCTGCCGCTTAGCCCTTACAAGAATGAAGGACCTCCCAATGCTTTGCAGGCTGCCCCAATAAAGCATCAAAACTATGATGAACCACTACTTCAGCTTGGAGGAAAATGCAAGAGGAAGGTATTTGCCAAACATGGTAACAATTGTCAGCATTTTCAATGCATGTGCAATCTATTTAGCTCTTCTTTAAGGTGATTAGATACATGCCAAATTGCGATGTTAAAATTTTATTTAGTCCTGCAATCAAATTTATTTTCCCTTCAACACTAGTACTCAAACATAGCTCCTCTCCACTTAATCATATTCCATATGACTTCTAATTCTATAACCATTTTACCTATGTATTGTCTCTTGCAATAGGTTGGCGATGCACATGCTGATCATCAATCCCAAAAGCCTGATAACACCATGCACAACTGTATAGATCAACACTTCTCGGCTCAAAACCAAAGTTGCTAGTGAGACTACTTTTGCTACCCTCGCTTCTTCAAGGGAATCAACATTGATTACTTTGCACTTCTGCTCAAAAGGGCCGTCATGGTACTCATGGCGTCAACAAAGAGAAGGGCAAGGGATGCATCCAGCGGGAAGTTTGCTGGCCCCCAACAGGATCCGCCAAAACACTCTCAAGGACGCCCAAAAGTAAAAAAAATCCCGAGGACAGCAACC |
| **CELL DIVISION CYCLE 5 (CDC5):**  GACCACATTGGTTGTGTGTGTCAAGGACACTGCAGTCATACTAAGATACCTTAAAAAATTTACAAGGATTTATTGATTTTTTTAACACGCTCATTCCCATTGATTTAGCCATTATCGCATACTAATCTTATACTTACATGTGGCACTCTCATGTTTGATTCCCTTATGTATGTGTCGAGGTTACAAGGTTTAAGTATTGAATTACACCATCAAAGTAAACACTTTTTCAAATTTTTAACCCATTGAGATTTCCGAAGCAATGACCATTGAGAGGACCATTTGGGTGGTACTATCTGAACCAAGAAGGCTACCACCTCCCATAGAGCAAGAGAGAAAGGACATCTAGGAAGCTTGGAGCCTGAGGGTATAGAAGAAGAAGCAATAAGGCTTCAGATCCAATTGCAAAATATGATTCCAGTGAGCCAGAATGATGGTGGAGAAAATTAAAGGAAATCCTTAGCATGTAAGTTTCAAGACCCTATGGTGTTGCCTCAACCTCTGCTGCTGAGCGTGCTGCCAGCTCAGCCTCTGCCATCACCTCTGCCTCTGCATCTGTACACGCCACCACTTCCACCTCCTCAACCTCTTGTGCTGCCACCTCCGCCACTGCAGCTGCACTCGTCATCTCTGCCTCTGCCTCTGCAACTGTGCACACCGTCACTTCTGCGGCTCGTGCTGCCACCTCTGCCTCTGCAGCTGCACTCGTCGCCTCTGTGTCTGCAACTGTGCACGCCACCACTTCCGCCTCCTCAACCTCCGCTGCTGCATGTGCTGCCACCTCAGCCTCTGCAGCTGCACACGTCGCGTCTGCATCTGCAGGTGTACACGCCGCCATTTCCACCTCAAACTCCAGTGCGGCTGTCTCAGTCTCTGCAGCTGCACACGTCGCCTCTGCCTCTGCAGCTGTACACACCTCCATTTCCACCTCCGCTGCTGCAAGTGCCACAGCCTCTGCCTCCGCTGCTGCACGTGCCACAGCCTCTGCCTCAGCTGCTGCCTCCGCTTCAGCAGCAGCACGTGCCACTGCAATATCATGTTCTTGAATAAGTTTCCTCCAATTCTCCCGTTCAACAAGCAAGTTTTCATATCTTAGCTGAAGCTTGTGCTCATTCTCATTCTGCTGCTTCACTGCTGCTTGCAAACTCTCCAAGCGAGACGGTGCTGCAAGCTGCTCCTGCTTAAACAGCAAGCGGAAGGACTCCAGTTCTGTTGCTAGAGTGTCAGCATCTTTAAACAATGCCTCAATTTGCCCCCACAAGGTTTCTGCCCTGACCTGATAACCTTGAGTGAGTACTTTCAGCTTTTGCTCTGACCGGACCGCTTTCTTGGTTTCCCCTTCCATGTGTTTCTTAATGATCTCAAATTCATATTGTAAAGCAGATAATCTTTCGTTGTTGCTTGCCACACTTGCAGAGTTATATGCTTGTCGTGAAGGGAAGTACATCATATCCTCAGCATGAGCATCCCTTGCCTCTGAGTAATCATCAATTGTCGCAACATCATGCCCCATTGCCAATCGCAAGAAGTCCGACTCTTCTTCAATCATCATAGCTGCATCTTTCAGCTCCTCATCTTCAAAATCGTCGAGTACAGGTATTTCAACTTGCTTTTTGCCATTGGCCACGTTCTTACTGGCCTTCTTCTTCTCCTTTCCTGATAATTCCTCCAAAGGATATTTCGCATTATCGTGCTCCAAAAGAGCTGCCAATTCCATCTTTATTAACTTATCTGCCTGTTCCGTCAATGTTGTTGCTACACTCTCTTCAGCCTGAGCCAAAGAACTCTTCAACCATTCAACTGACACTTGCGGAGGTCGTGGAAGGTCCCTTTGAAGGACTTTGGACCGCTTTAAAAGAAGGGCAGCTTGCCTTGCTACTTCTTCTGCTCGATCCCTTGCAAGCTTATCAGACATATCTTCTTCCACAGCATCCACAGCCTCTGGTTCGTCTGGTAACAAATCACGCACAACAACTTGGTACTCATATTTAGGCACAGGAAGATCACCCAGGCCAGCCCTAAGGTTCCTTCGTATCTCTGCCTGCCTTGCTTTCTCAGCCTTGACACTTTCATAAGGGGTTTCCAATCCTTCATTGATGTGAAGTTCGTCCCTGATTGGAGTACCCTTGGGAGTAGCATAGATGGAATCCTTGATGGGTGTCATTCTTGCTAAAGGAGTTAAGCCTACCCCACCAGAAGGGGTTTGAGCTGGAGTAGCAATCGGATTTGGAGTTTGTATTTCCCTCTTTTTGGGTGTCACTCCAGAAAAATCCCATGGATGCAAGTCCGGGTTCTCACCTCCAAGAAGAGGAGTCTGCACTTGCCTAAGCCGAGCTAAGTTCTCAGCCTCCATCATGATAGCGTCACCTTTCCCTCCTGGAGTTCTTTGAGGAGTTCTGAGTGGTGTCATGCCAGTTCTAGGAGTTTGCCCATAGTTTGCAAGCAGCATACGAGTAGCCCCACTTCCATCCCCAAGCTCATCATCGCCTGTAAGTAGATCATTAGAATAACCCATCTTTGCTATCTCCTCCAGCTCACGATCTGAAATCTGAGGAGGAGGAAGCATAAGCTTCGACCTCTTACGAACAGCCTCTGGGTCATTTAACCTGTTGATTTGCATTACAGAGGCTGGAGCATCCCTGCGCTCCGCAATCTTGTTGTGAGCGACATCCTGCTTCCTCAACTGCGCCTCTACATCAACTCGCCTCTTCCCTTCCAACTCTTCAATGGTGGTTGGAAATTGAGGCTGTTCTACAACCATATCTTCCTGAGATATGTCAAAGAAACCTGGGGGAGGCTTCTTTTCAAAAGGAATTTCAGCATTGTAGTCAATGCCTCTTTGTTTTCTCCTTCTATGCCTCCCCTCGATGCCTGCTGCTTTAAGCTCGCGGCGCTTTTGCAGAGAAGCGAGTCTCCTAGCTTCTTCAAGCTGCTTCTCCCGAGCTTTCCTCTTGGCCTTCTTTCCGCGAGTATTCGCAAGCCTGGCCCTTGCTTCAGAAAGCATTTCTTTTTCATCTTCATCCATGTCGACAGGGTCAGGCCTAGCGGGTTTGGACTCAGGGTTTGGATCAATTTCACCAGGCCGAAGTTTCCTTGGATCATCTGAAGGCTCGTAATTCTCATCCCGAGCACAAGCTGCATCCAGCAATTTCTCGTACCGTTCTAAGCATTGTGCAGGCGTGCGACCGACAATGGGAGCTATAGTTCGCCACTGAGTAGGCATGAGCTTTGCAAGATGTAAAAGCTTCTCATCTTCTTCCCGAGTCCATTCCGTCTTTTTGATGGATGGATCCAGCCATTCATACCATCGTGCTTTGCACTGCTTTGCCGATTTTCGGACAAGCAGAGATGATATACGAGCCCACTGATTCTTGCCGTACTTCATGACGGCAGCCTTCAGAATCTCATCTTCTGTGTTCTTCCAAACACCGCCCTTTATCATGATCCTCATCTTGCCCTGAAAACCCTGAAAAGGAGCTGTTGTTTAAGAAGCCTCTGTGATACACAGGAAAGATGAGCCTCGCAATGCAGGCAAGACTGCGTAGTTACACGGTTGACCTAAAGAAACTGAAGGCACAAGATAAACTACAGGCACGCCGTTGCTTCGCATACGGCTTCGCAGGTAGTGAGACATTATGAAACCCCCTAAAACCCCCTAATTGAG |
| **APETALA 2 FAMILY PROTEIN INVOLVED IN SA MEDIATED DISEASE DEFENSE 1 (APD1):**  CGAGATTTGACAATTAAAAATGTATGCACATGCATTGAAAAGCTAGACATCCGCCAAAGCGAAACATGAAGTAGACAAGCATGGCACATTAAAGTGAGCTTCTCAATTGCTCTCATACCACAACACACCACCCCTCATATCAATGTGAAAACAAATGAACAGATCTTGCGAGCTTCTTGCTTTCTTCATCTAAGACGCGGACAATGTCCTAATGAACAATAGTATATACTTTTGAAGAACTTGCAATCCTGCTACCTCATTACTACCATACTTTTTACAGCATTTCATACTGCGCCCCTCCGAAATCTGGTCATACCTCGTGTTCCAAATTAAAAGGTGGCATAAGAAATTGCTCCATCTCCTCCTAGAAGCAAAGCAAACAATTAGAGGACAATGAAATCCCAAATCCTTGCTTGCTTTTGGACACTCCCTAGCAGTTTGTTAAGTGATCCATGTGAATGCTCGACCCTCCTTTCTAATAAGGGGGTCTTGAGCTAATAATGGTTTCCAAAACTTGGCAACTTTGTTTGCTAAGAGCTGTGGAACATCTGCTCATCTCTGGAAAACTAGTAGACTTTATAGCTTGCTTCGATTGGGAGTACGCCAATCAATGAACACGTGATATCACTGTAAGGATAATTAAGGATAAGGACTGTTGCCTGCAAAGGCAGAATGCTGGTGAAGAGGAGGCGGCAAATAAGATTCTGACAAGTAGAAAGGTGCTGTCTGGTGAAAAGGCTCAAAGGGTTGTCCTTGCTGCTGCATTAGCTTTAAAGCTGCTGATTGCTCATAAACAGAAGTGGGTGGTGAATGAGCCTGCTGAAAGGATGCCAAAGTTGCGGCTTGGTGAAGAACAGATGCAGTTTCATCAAAAGTTGATGTACGCTGATACGCAGATGAGGGTTCATCAACAGATGCCATTTGATGAAAGGGTGGTACTTGATGGTACACCGATGCAGGTTTGTCATAACATTGAGACTGATGAAAAGACGATAAATCAATTCTTCCATCTTCAAGTAGCTCTGAAGAGCGCCGCCTCCGTACACAACTCTCTACCCTCTTCTGCTTCTTTTTGTTTGCAATAGCTTCTTTTGTTATTTGAAGAAAATCCTCCCACTGAAGTCCCTGTAGCTCTTGCTTCTCCTCCTCAGAGAGTTCAAAATTTGGTACTCTTCCGCAAAGGTAGGCAGCCCTGTCATACAAATGAGCTGCTTCTTCCATAGAAGCAACTGTGCCCAAATGTATTTGCTTTTTGTCCACCTTTATTGCTGCTTGCCACTTCATATTTTTGAAGTACACACCTCGTAGCAAACAAGGGTCCTGATTCTCTGATTGCTTCCTTCTGGAAGCTTTCCTCAGCTTCAGTGCATGATCTTGAAATGCATTGTTTAAGTCCTTCATTGAGTTATCACATACAGAAAAGGAAGGGCTGGGTTCCGCTGCAGGAACCAGGTTTTTCTCCTGTCGTGTCTCCAAGATGCTCGATTCACATTTCTGGTAGGGAATCTCTTCAACCACCTCCTTGCGCTCCATTGCACGTTTTTGCTCATTGAGTCTTGTAAGTTGATTGAGTATTTTATACCTTTCTTGTGCACCGAGGTGGCGCCATTTTCTGATGCTCACCATTTCACAAATGTATTGGCAATTCCAGAAACAGTAGTTCTTTGGAGCCTCCAATTCTCAAGCAACTCTCACAGCTCGGAGTAATGAATGTAGTTACATCTATATATGAGGTGTTTGAGAACTCTTTAGCAACCCCTTTACAAGCTAGTTCTTCCAAATGTTCTGGCTTCCAAGTCACAACCTGCTTGCTAATCTGTGAGAGCTGGTCGGCGTCAAGGCTGCCTACCTTCTGGAGCTGCCGATCTTCGTCCCCCCCGCCAAACAGAAAATGCTCGCCACCTAGAGAGAGTGTACAACGCAAATTTTG |
